# Unlocking Selenium Chemical Space via a Programmable Synthesis Platform Bearing Cannabinoid Receptor Recognition Motifs

**DOI:** 10.1021/jacs.5c16359

**Published:** 2026-04-28

**Authors:** Malliga R. Iyer, Pinaki Bhattacharjee, Subhradeep Dutta, Maloba M. M. Lobe, Paul D. Volesky, Grzegorz Godlewski, Henry L. Puhl, Sergio A. Hassan

**Affiliations:** † Section on Medicinal Chemistry, National Institute on Alcohol Abuse and Alcoholism (NIAAA), 2511 National Institutes of Health (NIH), 5625 Fishers Lane, Rockville, Maryland 20852, United States; ‡ Laboratory of Physiologic Studies, National Institute on Alcohol Abuse and Alcoholism (NIAAA), National Institutes of Health (NIH), 5625 Fishers Lane, Rockville, Maryland 20852, United States; § Laboratory of Biophotonics and Quantum Biology, National Institute on Alcohol Abuse and Alcoholism (NIAAA), National Institutes of Health (NIH), 5625 Fishers Lane, Rockville, Maryland 20852, United States; ∥ Bioinformatics and Computational Biosciences Branch, National Institute of Allergy and Infectious Diseases (NIAID), National Institutes of Health (NIH), Bethesda, Maryland 20892, United States

## Abstract

Developing synthetic methods that allow controllable homologation to quickly access new chemical space is vital yet remains challenging for studying biological pathways. Utilizing underexplored elements as biological probes offers promising platforms to reveal uncharted aspects of G-protein-coupled receptor (GPCR) pharmacology. Here we report the development of an expeditious, sustainable platform for the scalable conversion of chloro-imidoyl­sulfonyl­ureas, to provide one-pot access to synthetically versatile chiral, pro-chiral and achiral selenosulfonyl homologated compounds bearing the cannabinoid receptor-1 (CB_1_R) recognition motifs. The synthetic route was designed using Na_2_SeSO_3_, a benign selenium source under aqueous conditions to enable the target-oriented synthesis of novel seleno-cannabinoid agents labeled as SelenoCanns. This Bunte-reaction-inspired seleno-homologation opens the door for on-demand synthesis of bioactive organoselenium drug-like molecules, covalent-drug conjugates, click handles, and seleno-fluorescent probes, thus opening a vast space of previously inaccessible molecules for expanded structure–function studies on the CB_1_R. Of the new chemotypes discovered and synthesized, many compounds showed nanomolar to sub-nanomolar binding affinity for the CB1 receptor.

## Graphical abstract

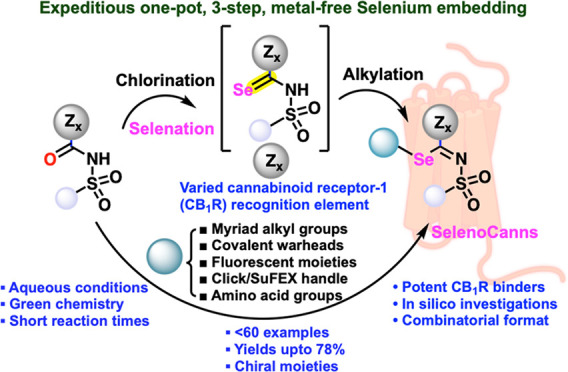

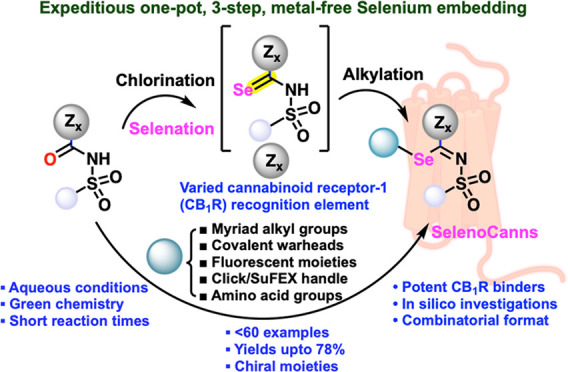

## Introduction

Modulation of G-protein-coupled receptors (GPCRs) by novel ligands enables the exploration of distinct biological pathways that influence physiological responses.^1–3^ The integration of ultralarge virtual libraries for molecular docking, combined with machine learning approaches, is increasingly used to identify novel chemotypes and drug-like entities.^4,5^ Consequently, there is a growing demand for robust synthetic methods capable of generating target-oriented compound libraries and providing datasets for training large language models.^6–8^ If the synthetic methods lean toward sustainable approaches, it further incentivizes the broad application of such methods. Incorporation of the under-utilized chalcogenide element selenium in drug discovery campaigns is increasing rapidly.^9–11^ The discovery of the first organoselenium compound in 1836 laid the foundation for various organoselenium compounds that have been developed as important structural motifs in numerous biologically significant synthetic scaffolds.^10,12,13^ Thus, organoselenium compounds could serve as pivotal contributors in medicinal frameworks.^10,11,14^ The biochemical implication of selenium has undergone significant elevation following the discovery of selenocysteine in numerous mammalian enzymes.^15,16^ Presently, the roster comprises 25 selenoproteins, including notable examples such as glutathione peroxidases, iodothyronine deiodinase, and thioredoxin reductase.^16^ Selenium is a crucial dietary component as well, with several organoselenium agents characterized for their activity in glutathione peroxidases and deiodinase.^17^ Inadequate selenium intake leads to the deactivation of selenoproteins,^18^ thereby inducing oxidative stress and contributing to cognitive decline, which is implicated in the pathogenesis of neurodegenerative conditions like Alzheimer’s disease.^19^ Selenium, when integrated into the catalytic site of glutathione peroxidase, effectively impedes lipid peroxidation induced by ferroptosis.^17^ Intervention with selenium or selenium-based ferroptosis inhibitors has also been shown to significantly improve ethanol-induced liver deterioration.^20^ Within the realms of modern medicinal and environmental applications, chalcogenide elements of the periodic table in their various oxidation states are proving to be vital.^21,22^ Selenium, an essential micronutrient, is now being utilized in various biochemical investigations and can potentially contribute to advancements in disease treatments. Addressing biological challenges implicated in intricate diseases necessitates innovative approaches to developing new chemical probes and drug-like molecules, frequently in quantities that are scalable. Consequently, the development of efficient synthetic methodologies for organoselenium bioactive compounds is in high demand.^23,24^ The sulfonylurea moiety present in synthetic compounds provides a valuable pharmacophoric accessory in diverse applications, including disease treatments and agricultural uses. Sulfonylureas (SUs) commonly act as anti-diabetic agents and exert an influence on various biological pathways,^25–28^ including anti-convulsant properties^29^ and insulin secretion modulatory properties.^30,31^ While selenium-based analogs have been researched considerably, sulfonylseleno urea compounds (SSUs) have had no sightings in the literature, except for a sulfonyl­ferrocenyl-attached seleno compound.^9^

Sulfonylureas are readily synthesized active pharmaceutical ingredients, and their extensive synthesis methods have been well-documented.^32^ In contrast, the lack of robust routes to access the analogous seleno­sulfonyl compounds has limited their applications so far. Seleno analogs have been shown to have novel properties and applications (Figure 1A).^33–35^ An important chemical niche would allow the generation of novel, non-traditional chemotypes with Se-bearing stereodecoys, which are yet to be explored pharmacophores in the biological realm (Figure 1B-C).^36^ Conventional synthetic methods for forging C–Se lack efficient methodology and have been plagued by formidable challenges, e.g., demanding high-temperature reactions, hazardous and toxic reagents, and also costly transition-metal catalysts.^37^ Transition-metal-catalyzed cross-couplings have reshaped modern synthesis and are widely used to forge carbon selenium (C–Se) bonds linkages central to many bioactive scaffolds and intermediates across pharmaceuticals and medicinal chemistry.^37,38^ Despite their utility and industrial track record, these methods often depend on catalysts that are costly, are air- and moisture-sensitive, and require specialized ligands, additives, or cocatalysts. Residual metal impurities also complicate purification and can limit pharmaceutical suitability. These constraints motivate the development of metal-free, sustainable, and cost-effective alternatives for C–Se bond construction that avoid toxic reagents and trace-metal contamination.^39^

**Figure 1:**
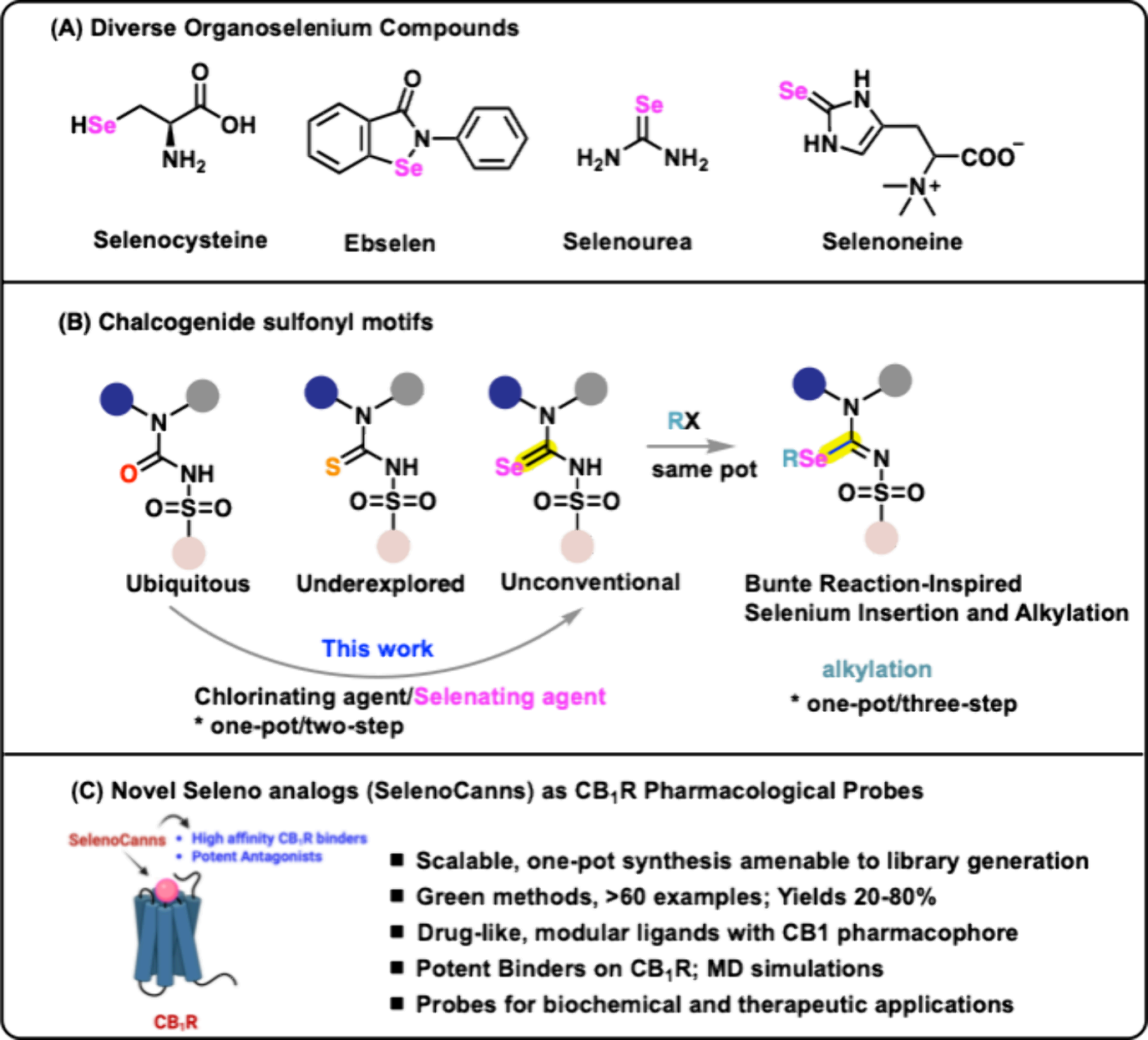
Inspiration for selenation and selenohomologation of sulfonyl­urea motifs. Image generated partly using https://BioRender.com (2026).

Herein we highlight a simple, one-pot sequential approach to generate terminal sulfonyl­seleno­carbamide transient species which, upon its propagation, allows for the generation of sulfonyl­isoseleno­carbamide analogs in a complex scaffold. We now show a robust synthetic approach, utilizing sulfonyl­ureas accessorized with pharmacophores meant to engage cannabinoid CB1 receptor —generating seleno­cannabinoid ligands called SelenoCanns. Through pharmacological assays we show that SelenoCanns generated from this route are potent binders of CB_1_R and act as functional antagonists. CB1 receptor antagonists have roles in ameliorating metabolic and cardiovascular diseases including obesity and diabetes and in modulating alcohol intake and fibrosis.^40–43^ To the best of our knowledge, this is the first report of C–Se bond formation under transition-metal-free conditions involving the Bunte reaction utilizing sulfonyl­urea and a CB1 pharmacophoric scaffold as a key structural framework.

## Results and Discussion

Current methods to convert urea to selenourea-containing derivatives involve the use of malodorous reagents analogous to Lawesson’s reagent, namely Woollins’s reagent or unstable selenocyanates.^44,45^ These routes, however, remain non-starters for the generation of seleno analogs bearing sulfonyl substitutions. Thus, a sustainable process for generation of sulfonyl­seleno­carbamide analogs bearing complex pharmacophores is highly desirable but remains unsolved in organic synthesis.

Organic selenosulfates (RSeSO_3_M, M = Na, K)) are commonly known as Seleno-Bunte salts for their ability to be used as “seleno surrogates”,^46^ named after Hans Bunte, who first reported the sulfur surrogates in 1874.^47,48^ A direct synthesis of Na_2_SeSO_3_, which can be generated in situ by the reaction of Na_2_SO_3_ with Se powder, showed it to be an odorless reagent for the selenation of alkyl halides to produce dialkyl diselenides.^49^ Many methodologies have appeared expanding the use of Bunte salts, including some from our own lab.^46,50,51^ Despite the well-established application of SUs in various biological and environmental settings, there is a paucity of routes for sulfonyl­seleno analog generation. We questioned whether we could integrate a seleno-Bunte salt into a SU motif to selectively deliver isolable seleno­sulfonyl­ureas or in situ which could be propagated to generate organoselenium analogs for diverse applications. On this basis, we hypothesized that a sulfonyl­urea imidoyl halide may serve as an intermediate precursor to a sulfonyl­urea seleno-Bunte salt under the right conditions, which may then collapse to yield seleno­sulfonyl­ureas. This pathway, if successful, would generate sulfonyl-bearing seleno adducts with myriad provisions on the three-clover amidino framework (Figure 1B). A protocol which allows for the generation of seleno analogs based on SUs as well as concurrent augmentation to chiral and pro-chiral handles will provide a platform for programmable target-oriented synthesis (Figure 2). We envisioned that delivering the selenating agent in an aqueous form in the presence of an aprotic solvent would avoid the generation of a malodorous selenol intermediate. The selenation reaction with Na_2_SeSO_3_ will thus be environmentally benign over other limited options. Owing to the cheap and abundant starting materials and selenium reagent, our novel synthetic method will advance practical applications of organoselenium technologies.

**Figure 2:**
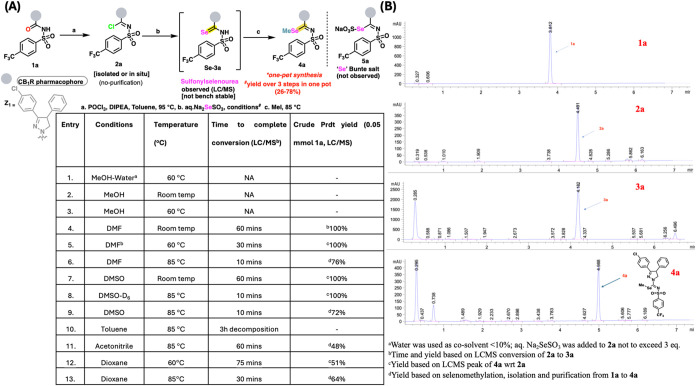
(A) Method optimization. (B) LCMS traces for compounds **1a** to **4a**.

### Optimization of Reaction Conditions (Reaction Development)

Our interest in cannabinoid receptor probes based on pyrazoline sulfonyl­urea precursors has led to potent CB1 receptor blockers, which are currently being explored for ameliorating a range of health conditions.^40^ Two compounds, MRI-1867 (INV-101/zevaquenabant) and MRI-1891 (INV-202/monlunabant), have undergone clinical trials. We sought to explore the generation of pyrazoline sulfonyl­seleno­ureas directly from the sulfonyl­urea intermediate precursors.^52–55^ A privileged 3,4-diarylpyrazoline sulfonyl imidoyl chloride obtained from the corresponding urea **1a** served as an ideal precursor to fine-tune the selenating conditions. In an optimized approach, we now show the successful execution of the reaction as depicted in Figure 2. We present a protocol where an inexpensive, odor-free, and safe-to-obtain aqueous inorganic selenosulfate can displace a SU imidoyl chloride to deliver sulfonyl-containing seleno­ureas. Using **1a** as the model system, imidoyl chloride **2a** could be obtained cleanly from substrate **1a** by treatment with POCl_3_/DIPEA (N,N-diisopropyl­ethyl­amine) at 95 °C.^56,57^ Evaporation of the toluene solution and POCl_3_ followed by treatment of the crude intermediate with aqueous Na_2_SeSO_3_ led to the generation of carbo­seleno­amide analogs in near complete conversion, likely resulting from the putative Bunte intermediate (**3a′** not detected) (Figure 2A). Surprisingly, the selenourea **3a** (carbo­seleno­sulfon­amide) could not be isolated cleanly and, upon purification, reverted to give the sulfonyl­urea compound **1a**.

This one-pot, two-step protocol through the intermediate imidolyl chloride can be carried out with isolation and purification of the imidoyl chloride. The nucleophilic displacement of imidoyl chloride **2a** by the seleno­sulfate ion worked best in DMF or DMSO at 85 °C, and the seleno­urea could be observed on LCMS within 10 min (Figure 2B). Solvents like dioxane were also acceptable. The reaction did not give the seleno products in alcoholic solvents like methanol where the sufonyl­urea starting material was generated. The nucleophilic displacement also worked well in DMSO or DMF at temperatures 60–85 °C, beyond which decomposition could be observed. The reaction was successful at room temperature with extended reaction times in the case of **1a**. In general, we observed that, with substrate **1a**, the reaction performed the best at 80–85 °C for the conversion of imidoyl chloride **2a** to the transiently stable seleno­sulfonyl­urea **3a** via the putative Bunte salt. The presence of the seleno­sulfonyl­urea could be confirmed by its clean footprint in the LCMS (Figure 2B) The presence of the putative seleno-Bunte salt could be clearly acquiesced by subsequent trapping of the seleno nucleophilic center with an electrophilic agent like MeI **4a**, where the seleno methylated product could be obtained in moderate to good yields, as observed from LCMS (Figure 2B).

Under mild conditions, the reaction delivers efficient seleno­methylation of sulfonyl­urea-imidoyl substrates bearing the cannabinoid receptor recognition motif Z_*x*_ underscoring its practicality (Figure 3). As indicated in Figure 3, the CB1 recognition element Z_1_ could be easily varied along with the sulfonyl fixtures, offering myriad combinations to build a diverse library of seleno methylated analogs (**4a**–**4v**). The reaction also proceeded with excellent stereo retention over three steps, as seen in the case of compound **4h**, which was obtained from enantiopure sulfonyl­urea starting materials.^57^ Similarly, cannabinoid recognition elements Z_2_ (an ethyl­pyrazoline core)^58^ and Z_3_ (a tetrahydro­pyridazine core)^59,60^ could be varied with sulfonyl attachments and seleno appendages.

**Figure 3:**
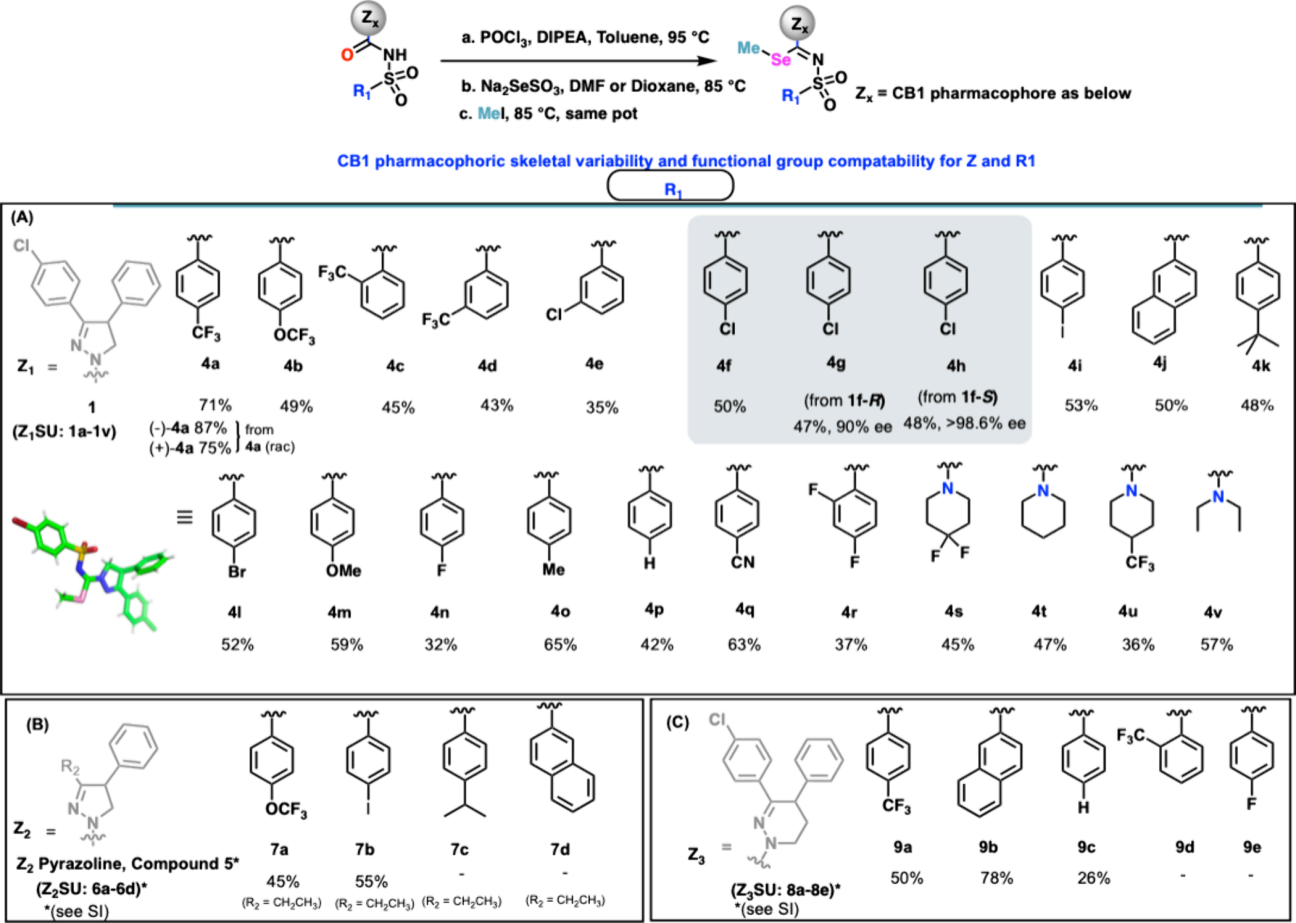
Scope of substrates for selenomethylation.

It was immediately rationalized that the nucleophilic selenium center can be merged with numerous electrophilic agents to yield seleno homologated products (Figure 4A–C). Indeed primary, secondary, and even tertiary alkylating agents could be treated with the seleno-Bunte intermediate to generate a library of selenium-containing compounds. Unsurprisingly, aromatic electrophilic reactants were not amenable to the selenium nucleophile trapping.

**Figure 4:**
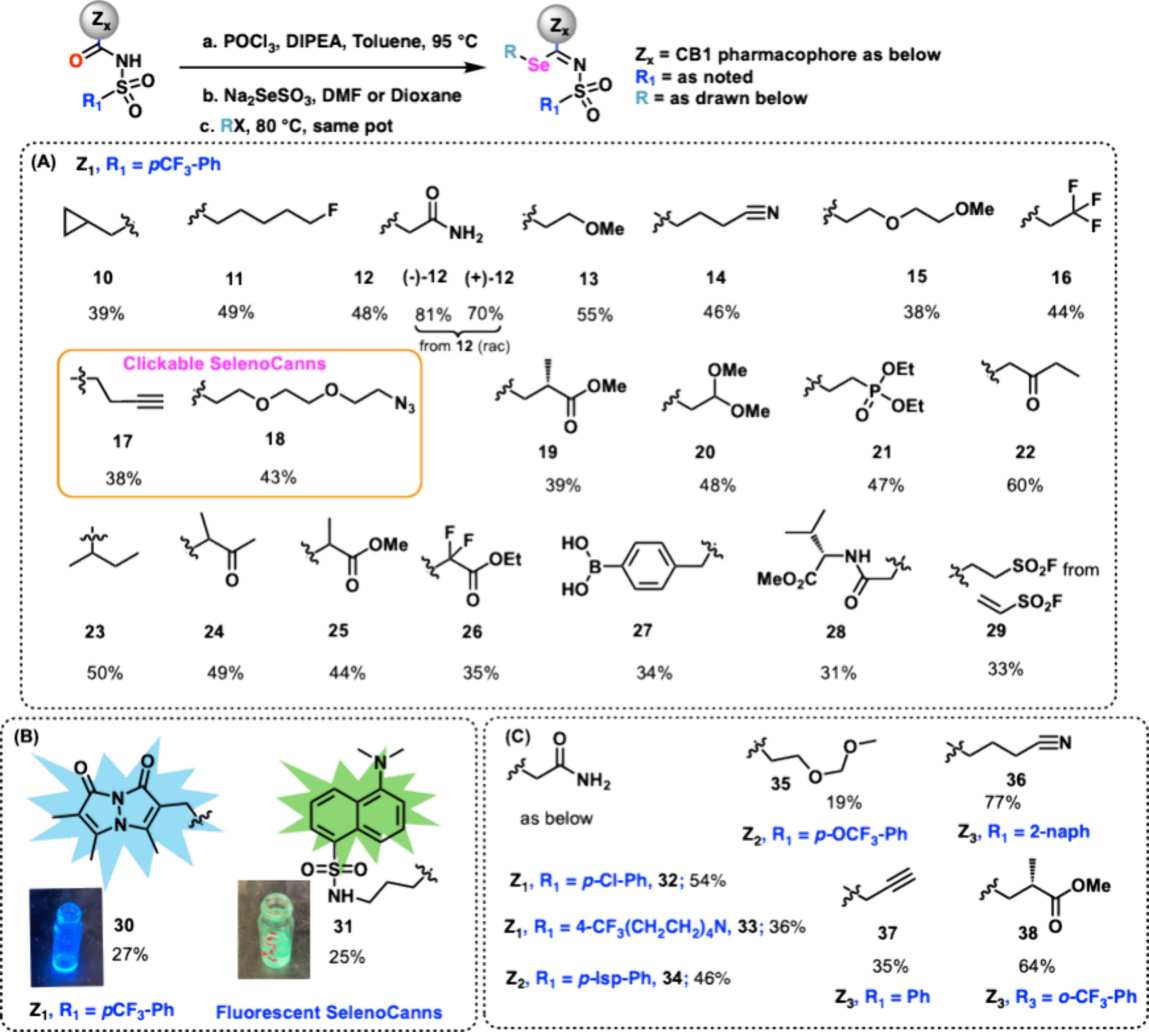
Se augmentation and substrate diversification.

This protocol delivers a versatile set of taggable synthons that support modular downstream derivatization (compounds **10**–**38**). As an illustration, click chemistry is routinely applied in biological settings for labeling, ligation, and cyclization.^61^ We also introduced clickable handles with azide or alkyne end zones for generating click seleno-cannabinoid probes (e.g., **17** and **18**). Electrophilic warheads like a fluoro (compound **11**) or cyano end group (compound **14**) could be installed easily, as also could be a masked aldehyde like compound **20**. Simple esters (**19**, **25**), phosphonate esters (**21**), and ketones (**22**, **24**) can be introduced at the selenium arm via their respective halides. An unprotected bromomethyl phenyl boronic acid was coupled with the seleno nucleophile to yield the putative covalent probe **27**, with boronic acid bearing the seleno-cannabinoid recognition element. In a similar vein, a seleno-cannabinoid conjugated to an amino acid-bearing electrophile was also generated. For example, an amino acid accessory, l-valine acetyl bromide, can be attached to the selenium center in acceptable yields to give the amino acid selenium conjugate **28** (Figure 4A). Treatment of vinyl sulfone with the seleno intermediate delivered the Michael adduct, yielding the sulfonyl fluoride **29**, allowing for potential SuFEx explorations. A unique advantage that could be harnessed from this protocol is the introduction of fluorescent groups using bromo­bimane **30** or a dansyl linker **31** via an electrophilic handle that could be conjugated to selenium to generate seleno-cannabinoid fluorescent probes (Figure 4B). As alluded to previously, the cannabinoid recognition element Z_*x*_ can be varied with various permutations and combinations on the sulfonyl end and the R electrophile (Figure 4C, **32**–**38**), offering numerous opportunities for chemical space expansion and structure–activity relationship refinement.

### Pharmacological Studies

To glean the role of the novel compounds in CB_1_R-mediated molecular interactions, we assessed the binding affinity of a subset of the compounds in radioligand binding assay and GRAB_eCB2.0_ for functional activity.^62–64^ With the availability of a library of drug-like molecules in hand, we conducted radioligand-based CB_1_R binding studies of a select few molecules. To our delight, many of the tested seleno compounds turned out to be high-affinity binders on the CB1 receptor (Table 1). In radioligand binding studies, compounds **4a**, **4q**, **4s**, **4t**, **4u**, and **4v** were shown to be high-affinity binders, with **4s** and **4u** having sub-nanomolar binding affinity for CB1 receptors. The binding affinity for **4h** selenomethyl analog was slightly better than for the corresponding aminomethyl analog ibipinabant.^52^ An important point to note here is that the compounds tested (except **4h**) were racemic, courtesy of their stereocenter at the C4 position of the pyrazoline ring. Separation into component enantiomers further offers the potential to accentuate the binding affinities at the CB1 receptor for all of the compounds presented here. Indeed, this is what we accomplished with racemic compound **4a** and **12**. Chiral preparative HPLC separation of **4a** led to (−)-**4a** and (+)-**4a** enantiomers. Likewise, separation of **12** led to (−)-**12** and (+)-**12** enantiomers. As observed in Table 1, the radioligand binding data showed that CB1 affinity resided with the (−) enantiomer in the cases of (−)-**4a**, **4h**, and (−)-**12**. Additionally, introduction of chirality at the selenium handle offers the potential to resolve the C4a chirality by way of diastereomer formation (e.g., compound **19**). The α,α-difluoro-substituted analog **26** had weaker affinity compared to other tested compounds, possibly pointing to the detrimental effects of the (tertiary) α,α-disubstitution. Gratifyingly, compound **31** showed a CB1 binding affinity of 157 nM. This is particularly exciting as fluorophore tags are known to penalize affinity in some cases. Here, competitive binding experiments demonstrated that the aforementioned CB1 fluorescent ligand preserved high target affinity.^65,66^ Further studies indicated that, as anticipated, these compounds behaved as antagonists of the CB1 receptor, as seen from the GRAB_eCB2.0_ sensor-based assay (Table 1, Figure S1, SI) and the β-arrestin assay (Figure S4, SI).^64^

**Table 1:**
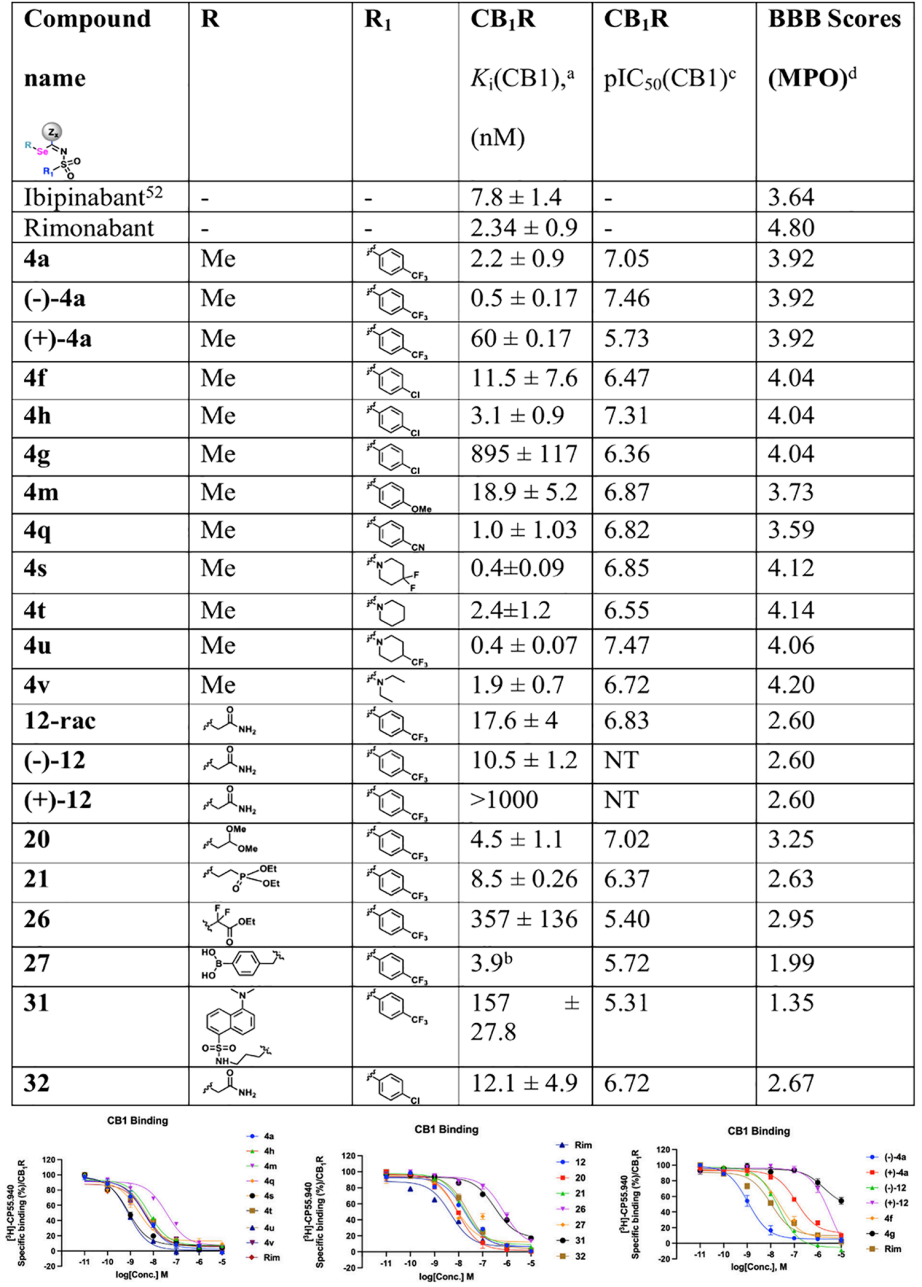
*In Vitro* CB1R Binding Affinity of Select SelenoCanns

a Binding affinity for CB1 was determined from binding displacement isotherms using [^3^H]-CP55940 as the labeled ligand and crude mouse (C57BL/6) brain membranes. K_i_ values were calculated using the Cheng–Prusoff equation and plotted using GraphPad Prism 10. Data represent mean ± SEM from three independent experiments.

b Data generated from a single experiment with technical replicates.

c Apparent functional CB_1_R antagonism was determined using GRAB_eCB2.0_ sensor in HEK293 K cells in the presence of CB1 agonist CP55,940 (at EC_80_ concentration) for select compounds.^64^ pIC_50_ values were calculated and plotted using GraphPad Prism 10. Data represent mean from three independent experiments.

d In silico BBB score calculated using Molsoft LLC. Blood–brain barrier (BBB) scores below 3 are not expected to be BBB penetrant. NT: not tested.

### Computational Study

The hypothesized reaction mechanism (Figure 5A), coupled with the promising pharmacological profiles exhibited by several compounds, prompted us to conduct a more in-depth analysis using computational methods. We examined the energetics of the chalcogen substitutions to gain a better understanding of the reaction conditions required for synthesis and modeled compound **4h** to compare its effect on CB_1_R behavior to that of ibipinabant.

**Figure 5:**
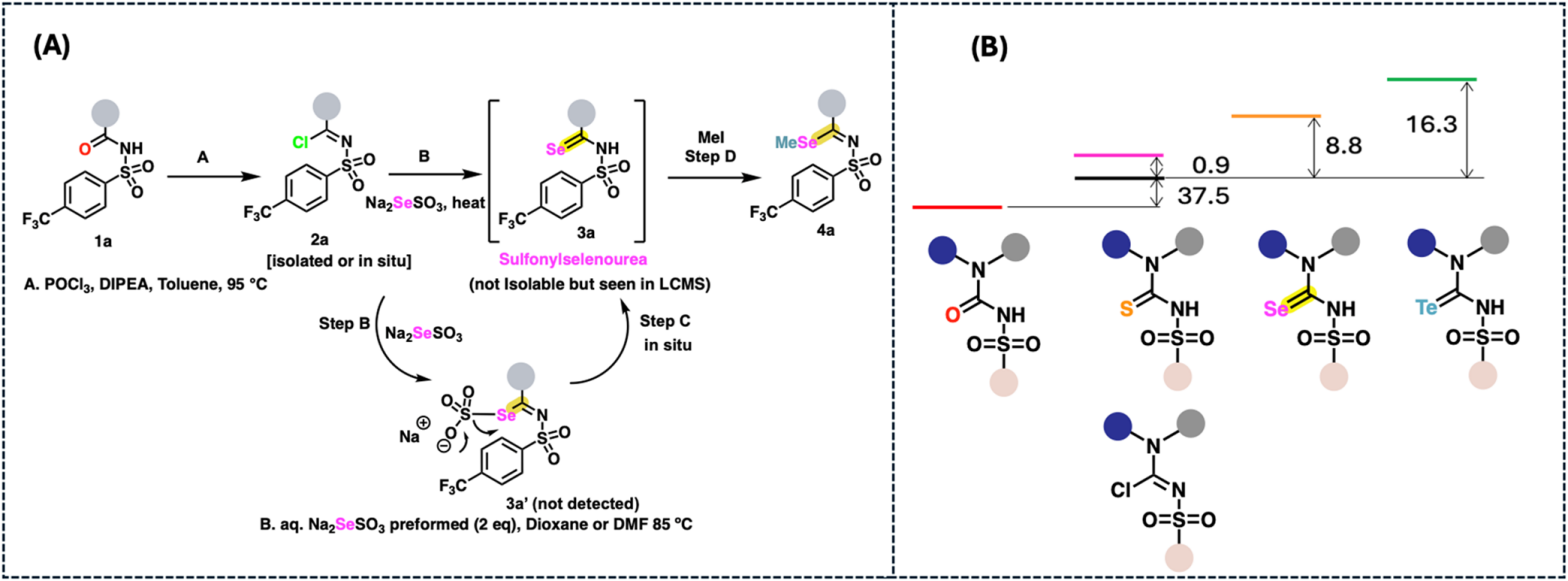
(A) Mechanistic outline of the reaction process. (B) Calculated bond dissociation enthalpies of O, S, Se, and Te (in kcal/mol); see SI.

Importantly, selenium’s large atomic radius coupled with its low electronegativity in comparison to sulfur results in lower bonding energy and greater propensity to oxidation. The calculated homolytic bond dissociation enthalpies of the chalcogen atoms on Arm 4 in the sulfonyl series show the expected trend (see Methods and SI): O (109.2) > S (70.8) > Se (62.9) > Te (55.4) kcal/mol, suggesting that O-to-Se substitution requires an appropriate intermediate species and elevated temperatures for the reaction to proceed (Figure 5B). Step ***a*** in the reaction shown in Figure 5A connects both sides of the process, rendering step ***b*** feasible at lower temperatures and making the formation of the sulfonyl­seleno­urea species likely, although still thermodynamically unstable. It is thus expected that the small population of selenium species present at equilibrium will gradually convert to stable selenomethylated products, yielding increasing amounts over time—consistent with experimental observations (cf. Figure 2A).

To understand the compound’s behavior on CB1 receptors, both ibipinabant and **4h** were initially docked in the orthosteric site of the receptor in a putative mode suggested by our studies of the related four-arm series.^67^ These two compounds differ by the −NH-to-Se substitution in Arm 4 and provide an opportunity to investigate whether such a small change can influence the ligands’ interactions with the receptor and the resulting conformational substates. We then performed molecular dynamics simulations (cf. Methods) and focused on the structural and dynamic differences. The statistical analysis of relevant metrics (cf. Methods) revealed how the −NH-to-Se substitution affects both the structure and dynamics of the receptor, and how these changes propagate to the intracellular side where the effectors bind. In **4h**, the longer C(1)–Se bond in Arm 4 (1.935 Å, compared to 1.486 Å for C(1)–N in ibipinabant), the absence of the proton donor group, and the more hydrophobic character of the arm have important consequences for the interaction of all the arms with the receptor, resulting in a slight repositioning of the molecule in the pocket (Figure 6A,C, Figure S5, and in the SI). Arm 1 shifts downward, making the polar interaction between Cl and W279 much stronger (cf. Methods for definition of relative strength), whereas Arm 3 is redirected toward TMH1, strengthening the Cl polar interaction with S123 (Figure 6B). In ibipinabant, the −NH group in Arm 4 is highly hydrated with extracellular water, contributing to its upward orientation and solvent exposure. By contrast, the −SeCH_3_ group in **4h** causes Arm 4 to interact much more strongly with nonpolar groups in the receptor (Figure 6B), effectively doubling the strength of hydrophobic stabilization. Additionally, despite the lack of solvation of Arm 4 in **4h**, nitrogen atoms N(1) and N(2) in the central five-membered ring become significantly more hydrated due to the repositioning of the ligand in the pocket, which permits greater water access on the side of the plane opposite S383 in TMH7. Overall, there is a significant increase in the interactions of the four arms of **4h** with the receptor, both polar and nonpolar (see Methods), as well as in the hydration of the polar groups (see Figure S5 in the SI and included ). A further role of extracellular water in the affinity of this four-armed series of compounds has been discussed in the Supplementary Information of ref 67. Despite the increased rigidity expected from these stronger interactions with the receptor, the enthalpic reward is likely to far exceed the entropic penalty, thus explaining the higher affinity of **4h** relative to that of ibipinabant. The differences in interactions of the four arms with the receptor induce structural and dynamic changes in the TMH, particularly affecting TMH1 and TMH7, which propagate to the intracellular side, potentially impacting effector binding (not analyzed here; Figure 6D).

**Figure 6:**
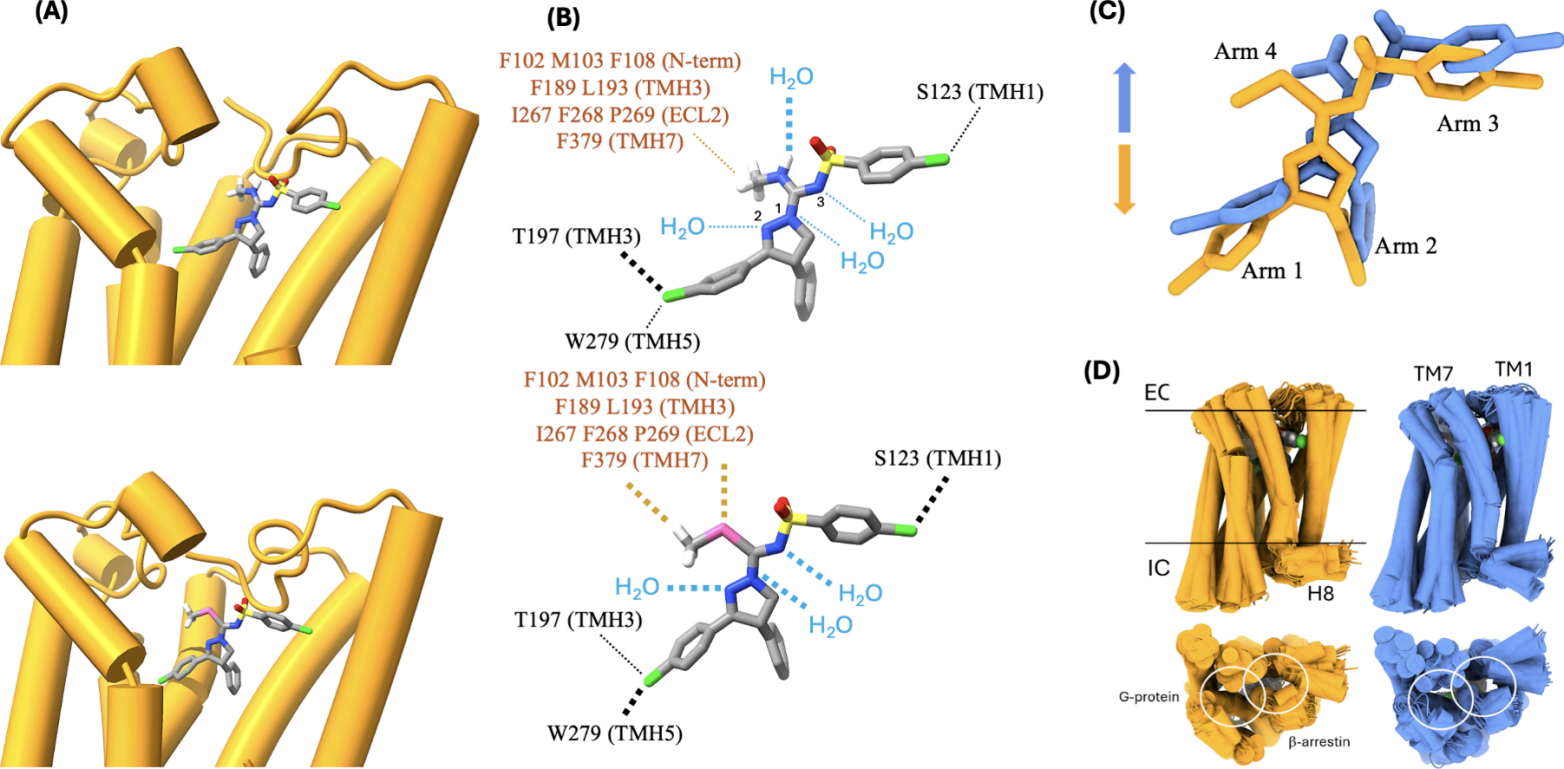
(A) Binding modes of ibipinabant (upper panel) and compound **4h** (lower) in the orthosteric pocket of CB1R from the simulations, with TMH1 on the right; part of TMH7 was removed for visual access to the ligands (Cl: green; C: gray; H: white; N: blue; S: yellow; O: red; Se: pink). (B) Interactions between the ligands and their environments (water and receptor) reveal major differences in strength between ibipinabant and **4h** (cf. Methods for a definition of relative strength). Interactions with minor differences are omitted (see in the SI for a three-dimensional distribution of interacting amino acids with a heatmap representation of interactions). Relative interaction strengths are indicated schematically by thick (stronger) or thin (weaker) dashed lines (cf. Figure S5). (C) The changes shown in (B) reposition the ligands in the pocket, resulting in specific and distinct interactions of each of the four arms with the receptor. (D) Changes in ligand–receptor interactions influence the dynamics of the transmembrane helices; these effects propagate toward the intracellular side, generating distinct conformational substates that can modulate effector binding (see Methods for a definition of conformers and ref 67 for further discussion; white circles indicate the approximate binding sites of effector proteins).

### Combinatorial Screening for Direct-to-Biology (DtB) Adaptability

Recognizing the obviously broad substrate scope and the structurally diverse scaffold products of the current one-pot seleno­alkylation reaction, we wondered whether we could integrate this platform for target-oriented combinatorial synthesis in a small library design. We selected six varied sulfonyl­urea precursors (Figure 7) bearing different CB1 pharmacophores and eight alkylating agents and carried out 6×8 combinatorial LC-vial chemistry using solutions of substrate mixtures. Among the substrates we evaluated, two SU precursors (**6d**, **7e**) and two alkylating agents (**Y3**, **Y5**) had not been explored in our substrate scope studies (Figure 3). At least 40 combinations from this 6×8 combinatorial library were not individually evaluated in Figure 3 (see Figure S2, SI, for full structures of the products). Using the general procedure outlined and DMF as solvent at 85 °C for the selenation/alkylation, the sequence could be carried out using a commercially available combinatorial setup. 81% of the combinations successfully underwent sequential procedures to provide products suitable for further optimization in potential DtB applications. Among these, 71% of the products were formed in crude yields greater than 60% as analyzed by LCMS fingerprint. These results highlight the excellent scope and potential of seleno embedding in plying the vast chemical space for GPCR drug discovery. This protocol thus amplifies the value of target-oriented synthesis, allowing for the construction of stereo-diverse, three-dimensional frameworks to drive translation endeavors in drug discovery.

**Figure 7:**
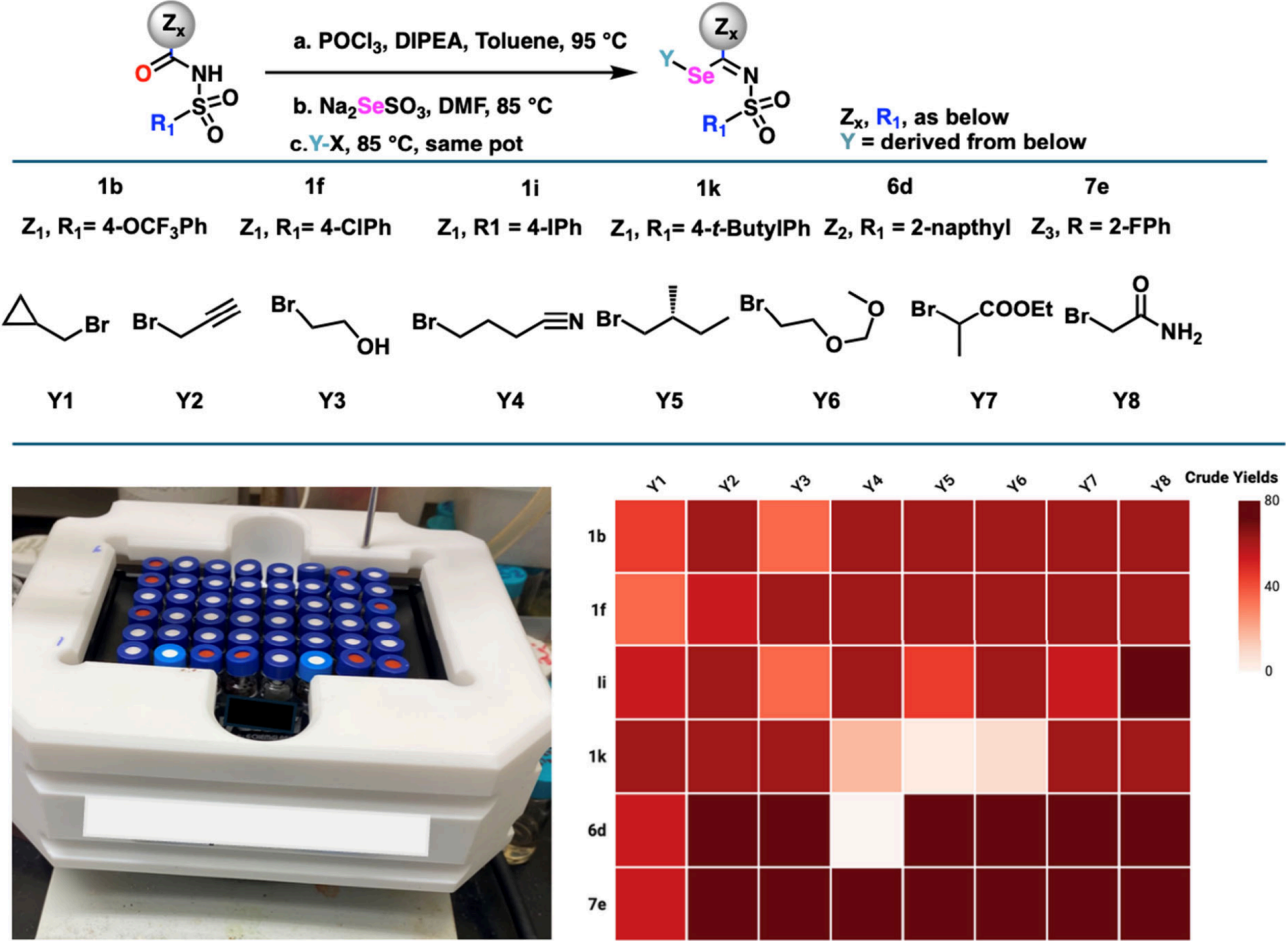
Screening of selenoalkylation substrates in a combinatorial synthesis library format. Image generated partly using https://BioRender.com (2026).

## Conclusions

We have developed the first successful conversion of sulfonyl­urea to seleno­sulfonyl analogs via a chloro-imidoyl­sulfonyl­urea intermediate, foregoing transition metal assistance. This method allows for facile, modular access to stereo-programmed organoselenium analogs in a rapid one pot, three-step approach through the utilization of a simple, aqueous selenium reagent, Na_2_SeSO_3_, obtained from cheap feedstock Na_2_SO_3_. The reactions proceed rapidly with complete configurable control of stereochemistry, and the products are easily purified under simple flash purification conditions. As such, rapid access to these novel seleno-adorned motifs bearing cannabinoid receptor recognition pharmacophores is now enabled. Cannabinoid receptors are an important part of the class A GPCR family, and cannabinoid antagonists have a significant role in ameliorating obesity, metabolic syndrome disorders, and organ fibrosis.^54,56,59^ A subset of racemic compounds tested showed that they have very high affinity toward the CB1 receptor and act as potent functional antagonists. Chiral separation of racemic **4a** and **12** into component enantiomers further reinforced the high CB1 binding affinities of these compounds and confirmed that the CB1 affinity rested with the (−) enantiomers. Similarly, given the evolving scope of the biological functions of seleno derivatives and the seemingly endless possibilities for substrate:​reagent combinations, this synthetic protocol offers a persuasive prospect to explore not only structure–function relationships on CB1 receptors and its secondary signaling pathways but also new therapeutic areas where selenium biology could be used. Additionally, amino substitutions, even a lipophilic and bulky adamantyl amino group at Z_*x*_, work with the three step, one-pot protocol (Figure S3, SI). Similarly myriad primary, secondary, and even tertiary alkyl halides can be used to alkylate the selenium center. Of note, from the perspective of targeting the CB1 receptor antagonism, the seleno fragments can be modulated to yield compounds with physicochemical properties that can limit the compounds to the peripheral tissues, sparing the CNS.^40^ Miniaturization of the reaction for applicability in DtB screening was also accomplished with high purity trends in the one-pot, three-step protocol. Molecular dynamics simulations provided an in-depth analysis of the interactions of the selenium analog **4h** within the CB1 inactive-state binding pocket, demonstrating that −NH-to-Se substitutions in the ligand have the potential to induce distinct receptor functionalities.^67,68^ In summary, the realization of a robust, site-selective selenium embedding to a heretofore inaccessible class of compounds paves the way for its potential application in the areas of translational medicinal chemistry, chemical biology, and biosensor technology.

## Supplementary Material



















## Notes

Experimental procedures, materials and methods, preparation and characterization, chiral HPLC separation chromatograms, combinatorial compounds, pharmacological assays, X-ray crystal structure coordinates, MD simulations data, and full spectroscopic data for all new compounds (PDF)
^1^H and ^13^C NMR spectra for all new compounds (PDF)
Simulations and analysis package (ZIP)
H-bond and hydrophobic frequencies (animation V1, MP4)
Hydration ibipinabant (V2, MP4)
Hydration selenium compound (V3, MP4)
Ligands gas phase optimized (PDB)
Ligands octanol optimized (PDB)
Ligands water optimized (PDB)

## #

Department of Chemistry, University of Buea, Cameroon
