# Supplementary material for: Unlocking Selenium Chemical Space via a Programmable Synthesis Platform Bearing Cannabinoid Receptor Recognition Motifs
Source: J Am Chem Soc. 2026 Apr 28;148(18):18649–59. doi: 10.1021/jacs.5c16359 (PMC13185125; doi:10.1021/jacs.5c16359)
Supplement: Supplementary file 1 [file ja5c16359_si_009.pdf]

Supporting Information for

Unlocking selenium chemical space via a programmable synthesis platform bearing cannabinoid receptor recognition motifs

Malliga R. Iyer[*1], Pinaki Bhattacharjee,[1] Subhradeep Dutta,[1] Maloba M.M. Lobe,[1] Paul D. Volesky,[1] Grzegorz Godlewski,[2] Henry L. Puhl III,[3] Sergio A. Hassan[4]*

[1]Section on Medicinal Chemistry, National Institute on Alcohol Abuse and Alcoholism (NIAAA), National Institutes of Health (NIH), 5625 Fishers Lane, Rockville, MD 20852, USA, [2]Laboratory of Physiologic Studies, National Institute on Alcohol Abuse and Alcoholism, National Institutes of Health, 5625 Fishers Lane, Rockville, MD 20852, USA. [3]Laboratory of Biophotonics and Quantum Biology, National Institute on Alcohol Abuse and Alcoholism, National Institutes of Health, 5625 Fishers Lane, Rockville, MD 20852, USA. [4]Bioinformatics and Computational Biosciences Branch, National Institute of Allergy and Infectious Diseases, National Institutes of Health, Bethesda, MD 20892, USA.

Corresponding author: malliga.iyer@nih.gov

**Table of contents**	**Page no**
General Methods	S3-S5
Compound Characterization data	S5-S46
Chiral HPLC traces of 4a, (-)-4a, (+)-4a, **4f-4h**, 12, (-)-12, (+)-12, **19**	S47-S50
X-Ray Structural data information of	S51
$GRAB_{ecb2.0}$ sensor-based assay	S53
Computational studies and MD simulations	S55
Structures of combinatorial library compounds	S58
LCMS trace for compound **39**	S59
β-Arrestin Assay	S60
CB_1R-ligand and water-ligand interactions	S61
Full Structures from Figure 3 and Figure 4	S62
Bibliography	S64
^{1}H and ^{13}C NMR of compounds	Separate file

Experimental Methods

General Information

Commercially available reagents were procured and used as is. Prohibitively expensive reagents and/or intermediates were synthesized based on reported procedures where indicated. Proton (^{1}H NMR and ^{13}CNMR) spectra were recorded on a Jeol 400 or Bruker 800 MHz spectrometer in $CDCl_3$ or DMSO-d_6 (unless otherwise noted) with the values given in ppm (TMS as internal standard) and J (Hz) assignments of ^{1}H resonance couplings were determined by iNMR or Mnova software. Mass spectra (HRMS) were recorded on a VG 7070E spectrometer or a JEOL SX102a mass spectrometer. Thin layer chromatography (TLC) analyses were carried out on Analtech/Miles Scientific silica gel GHLF 0.25 mm plates using various gradients of EtOAc:*n*-hexanes or $CHCl_3$/MeOH containing 1% NH_4OH. TLC plates were visualized under UV light or by staining in an iodine chamber. Flash column chromatography was performed on Teledyne ISCO Combiflash system. Product yields are un-optimized and reported yields could involve trituration (e.g Isopropyl alcohol-hexane mixture) after purification to give amorphous powder compounds. All compounds tested had ≥ 95% purity based on a combination of TLC, ^{1}H-NMR, ^{13}C-NMR, LC/MS, high-resolution mass spec and X-ray (where indicated) unless specified otherwise. Detection on LC-MS was carried out on Agilent 1200 using either Luna C18 3 mm (3 x 75 mm) where the mobile phase was 4% to 100% acetonitrile (0.05% TFA) standard gradient or Agilent EC-C18 poroshell 2.7 mM (3 x 50 mm) where the mobile phase was 50% to 98% acetonitrile (0.1% formic acid). The LC-MS chromatogram showed the expected molecular (MH^+) ion as well as a single peak at UV (254 nm) which was used to compute purity. Chiral rotation data was

obtained using Anton Paar polarimeter under shown conditions. X-ray facility at Georgetown University was utilized to obtain crystal structure of select compound(s).

Procedure for Preparation of Compounds

General Procedure for the synthesis of Na_2SeSO_3

A vial containing fine selenium powder (0.8 g, 10.1 mmol), sodium sulfite (2.4 eq., 3.024 g, 24 mmol) and distilled water (10.0 mL) was stirred and heated to 90 °C. After 4h at 90 °C, the clean colorless reaction solution was cooled to room temperature, capped tightly and used as needed. If stored under argon, this colorless solution is stable and usable for prolonged periods. The solution must be discarded if pale red/orange color start developing. This leads to incomplete conversion in the subsequent steps possibly due to reagent degradation.

General Procedure A for the synthesis of selenosulfonyl urea and in situ alkylation

In a dry round-bottom flask, compound **1a** (1 eq) was taken in toluene (5 mL), $POCl_3$ (1.5 eq), was added, followed by the addition of *N, N*-diisopropylethylamine (DIPEA) (1.5 eq) and the mixture was heated to 95 °C for 1.0 h under N_2 atmosphere. The reaction mixture was then cooled, and the excess reagents and toluene were evaporated in vacuo. The imidoyl chloride intermediate was dissolved in Dioxane (or DMF/DMSO) (5 ml). To this solution was added dropwise, premade (1.0 M), aqueous Na_2SeSO_3 (3 eq) and the reaction was heated to 85 °C. Upon completion of the reaction as seen from the selenosulfonyl product peak in LCMS, the appropriate alkylating agent (1.5 eq) was added dropwise to the reaction mixture and reaction continued until all the in situ seleno intermediate is consumed as seen by TLC and LCMS. The organic mixture was the extracted into ether, washed with brine and dried over Na_2SO_4. The sticky solid was purified by flash chromatography (35% hexanes in EtOAc) to pure seleno alkylated compound.

Following the general procedure A, in a dry round-bottom flask, compound **1a** (200 mg, 0.39 mmol) was taken in toluene (5 mL), $POCl_3$ (0.05 mL, 0.59 mmol), was added, followed by the addition of *N,N*-diisopropylethylamine (DIPEA) (0.1 mL, 0.59 mmol) and the mixture was heated to 95 °C for 1.5 h under N_2 atmosphere. The reaction mixture was then cooled, and the excess reagents and toluene were evaporated in vacuo. The imidoyl chloride intermediate was dissolved in Dioxane (or DMF/DMSO) (5 ml). To this solution was added dropwise, premade (1.0 M), aqueous Na_2SeSO_3 (2 eq, 0.78 mmol, 0.78 mL) and the reaction was heated to 85 °C. Upon completion of the reaction as seen from the selenosulfonyl product peak in LCMS, then MeI (1.5 eq, 0.59 mmol, 0.04 mL) was added dropwise to the reaction mixture and reaction continued until all the in situ seleno intermediate is consumed as seen by TLC and LCMS. The organic mixture was the extracted into ether, washed with brine and dried over Na_2SO_4. The sticky solid was purified by flash chromatography (35% hexanes in EtOAc) to pure seleno alkylated compound (126 mg, 54.7%) over three continuous steps.

The same reaction was repeated in DMF to give (161 mg, 71%) of pure product **4a.**

General Procedure B for the synthesis of sulfonyl ureas.

The sulfonyl urea intermediates were synthesized by treatment of the respective central core substituted pyrazoline/pyridazine/amine with the sulfonyl carbamates as indicated. To a solution of amine (1 eq) in toluene was added sulfonyl carbamate compound **1** (1.2 eq) and the resulting slurry was refluxed for 3h. After cooling the reaction to room temperature, the toluene solution was removed in vacuo and the slurry was purified using flash chromatography.

Sulfonylureas (SU) (1a-1v, 6a-6d, 8a-8e) were synthesized according to General Procedure B and as reported in the literature.

3-(4-chlorophenyl)-4-phenyl-*N*-((4-(trifluoromethyl)phenyl)sulfonyl)-4,5-dihydro-1*H*-pyrazole-1-carboxamide (1a)[1,2]

3-(4-chlorophenyl)-4-phenyl-*N*-((4-(trifluoromethoxy)phenyl)sulfonyl)-4,5-dihydro-1*H*-pyrazole-1-carboxamide (1b)[1]

3-(4-chlorophenyl)-4-phenyl-*N*-((2-(trifluoromethyl)phenyl)sulfonyl)-4,5-dihydro-1*H*-pyrazole-1-carboxamide (1c)[3]

3-(4-chlorophenyl)-4-phenyl-*N*-((3-(trifluoromethyl)phenyl)sulfonyl)-4,5-dihydro-1*H*-pyrazole-1-carboxamide (1d)[2]

3-(4-chlorophenyl)-*N*-((3-chlorophenyl)sulfonyl)-4-phenyl-4,5-dihydro-1*H*-pyrazole-1-carboxamide (1e)[4]

3-(4-chlorophenyl)-*N*-((4-chlorophenyl)sulfonyl)-4-phenyl-4,5-dihydro-1*H*-pyrazole-1-carboxamide (1f)[1,2]

***R*-3-(4-chlorophenyl)-*N*-((4-chlorophenyl)sulfonyl)-4-phenyl-4,5-dihydro-1*H*-pyrazole-1-carboxamide (1g)**[3]

***S*-3-(4-chlorophenyl)-*N*-((4-chlorophenyl)sulfonyl)-4-phenyl-4,5-dihydro-1*H*-pyrazole-1-carboxamide (1h)**[3]

3-(4-chlorophenyl)-*N*-((4-iodophenyl)sulfonyl)-4-phenyl-4,5-dihydro-1*H*-pyrazole-1-carboxamide (1i)[1]

3-(4-chlorophenyl)-*N*-(naphthalen-2-ylsulfonyl)-4-phenyl-4,5-dihydro-1*H*-pyrazole-1-carboxamide (1j)[1]

N-((4-(tert-butyl)phenyl)sulfonyl)-3-(4-chlorophenyl)-4-phenyl-4,5-dihydro-1*H*-pyrazole-1-carboxamide (1k)[1]

***N*-((4-bromophenyl)sulfonyl)-3-(4-chlorophenyl)-4-phenyl-4,5-dihydro-1*H*-pyrazole-1-carboxamide (1l)**[1]

3-(4-chlorophenyl)-*N*-((4-methoxyphenyl)sulfonyl)-4-phenyl-4,5-dihydro-1*H*-pyrazole-1-carboxamide (1m)[1]

3-(4-chlorophenyl)-*N*-((4-fluorophenyl)sulfonyl)-4-phenyl-4,5-dihydro-1*H*-pyrazole-1-carboxamide (1n)[2,5]

3-(4-chlorophenyl)-4-phenyl-*N*-tosyl-4,5-dihydro-1*H*-pyrazole-1-carboxamide (1o)[1,2]

3-(4-chlorophenyl)-*N*-(phenyl)sulfonyl)-4-phenyl-4,5-dihydro-1*H*-pyrazole-1-carboxamide (1p)[5]

3-(4-chlorophenyl)-*N*-((4-cyanophenyl)sulfonyl)-4-phenyl-4,5-dihydro-1*H*-pyrazole-1-carboxamide (1q)[5]

3-(4-chlorophenyl)-*N*-((2,4-difluorophenyl)sulfonyl)-4-phenyl-4,5-dihydro-1*H*-pyrazole-1-carboxamide (1r)[4]

3-(4-chlorophenyl)-*N*-((4,4-difluoropiperidin-1-yl)sulfonyl)-4-phenyl-4,5-dihydro-1*H*-pyrazole-1-carboxamide (1s)

3-(4-chlorophenyl)-4-phenyl-*N*-(piperidin-1-ylsulfonyl)-4,5-dihydro-1*H*-pyrazole-1-carboxamide (1t)[6]

3-(4-chlorophenyl)-4-phenyl-*N*-((4-(trifluoromethyl)piperidin-1-yl)sulfonyl)-4,5-dihydro-1*H*-pyrazole-1-carboxamide (1u)[6]

3-(4-chlorophenyl)-*N*-(*N*,*N*-diethylsulfamoyl)-4-phenyl-4,5-dihydro-1*H*-pyrazole-1-carboxamide (1v)[6]

3-Ethyl-4-phenyl-4,5-dihydro-1*H*-pyrazole (5)

To a solution of 1-phenyl-2-butanone (3.0 g, 20.0 mmol) in MeOH (33 mL) was added piperidine (0.33 mL) followed by acetic acid (0.33 mL). To this was added (37 wt% in H_2O) formaldehyde solution (5.5 mL, 74.0 mmol), and the resulting mixture was refluxed for 24 h. The reaction mixture was then cooled to room temperature, concentrated and taken up in a mixture of ethyl acetate and water. The organic layer was collected, dried over anhydrous Na_2SO_4, filtered and concentrated to give crude 2-phenylpent-1-en-3-one as an oil. This was used directly for the next step without further purification.

The crude 2-phenylpent-1-en-3-one was taken up in EtOH (24 mL), and hydrazine hydrate (9.7 mL, 200.0 mmol) was added to it. The resulting mixture was refluxed for 4 h. The reaction mixture was then cooled to room temperature, concentrated and taken up in a mixture of ethyl acetate and water. The organic layer was collected, dried over anhydrous Na_2SO_4, filtered and concentrated to give crude **5** as a viscous oil. This was used directly for the next step without further purification.

^1H NMR (400 MHz, $CDCl_3$): δ 7.30–7.24 (m, 2H), 7.24–7.15 (m, 4H), 3.97 (t, *J* = 9.7 Hz, 1H), 3.80–3.70 (m, 1H), 3.34–3.27 (m, 1H), 2.25–2.11 (m, 1H), 2.09–1.96 (m, 1H), 1.04–0.97 (m, 3H). LRMS: 175.1.

3-Ethyl-4-phenyl-*N*-((4-(trifluoromethoxy)phenyl)sulfonyl)-4,5-dihydro-1*H*-pyrazole-1-carboxamide (6a)

To a solution of 3-ethyl-4-phenyl-4,5-dihydro-1*H*-pyrazole (**5**) (0.52 g, 3.0 mmol) in toluene (5 mL), compound methyl ((4-(trifluoromethoxy)phenyl)sulfonyl)carbamate[2,5] (1.1 g, 3.6 mmol), was added, and the resulting mixture was refluxed for 3 h. The reaction mixture was then cooled to room temperature, and toluene was evaporated in vacuo. The residue was extracted into dichloromethane, washed with brine water and purified by flash chromatography (30-40% EtOAc in hexanes) to yield compound **6a** (0.64 g, 48%) as a colorless viscous oil. ^{1}H NMR (400 MHz, $CDCl_3$): δ 8.65 (s, 1H), 8.20 (d, J = 8.2 Hz, 2H), 7.40–7.23 (m, 5H), 7.08 (d, J = 7.2 Hz, 2H), 4.24–4.13 (m, 2H), 3.85–3.73 (m, 1H), 2.23–2.01 (m, 2H), 1.06 (t, J = 7.4 Hz, 3H).

^{13}C NMR (100 MHz, $CDCl_3$): δ 163.9, 152.8, 147.7, 138.3, 137.4, 130.7, 129.3, 128.1, 127.4, 120.5, 54.0, 52.3, 21.8, 10.3. LRMS: 442.1. HRMS (ESI) m/z: $[M+H]^+$ Calculated for ($C_{19}H_{19}F_3N_3O_4S$) 442.1048; Found 442.1047.

3-Ethyl-*N*-((4-iodophenyl)sulfonyl)-4-phenyl-4,5-dihydro-1*H*-pyrazole-1-carboxamide (6b)

To a solution of compound **5** (0.52 g, 3.0 mmol) in toluene (5 mL), methyl ((4-(iodo)phenyl)sulfonyl)carbamate[2,5] (1.2 g, 3.6 mmol), was added, and the resulting mixture was refluxed for 3 h. The reaction mixture was then cooled to room temperature and toluene was evaporated in vacuo. The residue was extracted into dichloromethane, washed with brine water and purified by flash chromatography (30-40% EtOAc in hexanes) to yield compound **6b** (0.68 g, 49%) as a colorless viscous oil.

^{1}H NMR (400 MHz, $CDCl_3$): δ 8.64 (s, 1H), 7.94–7.80 (m, 4H), 7.38–7.23 (m, 3H), 7.11–7.04 (m, 2H), 4.23–4.12 (m, 2H), 3.84–3.72 (m, 1H), 2.22–2.00 (m, 2H), 1.10–1.00 (m, 3H).

^{13}C NMR (100 MHz, $CDCl_3$): δ 163.8, 147.7, 138.9, 138.3, 138.1, 129.8, 129.3, 128.1, 127.4, 101.5, 54.0, 52.2, 21.8, 10.3. LRMS: 484.0.

HRMS (ESI) m/z: $[M+H]^+$ Calculated for ($C_{18}H_{19}IN_3O_3S$) 484.0192; Found 484.0185.

3-Ethyl-*N*-((4-isopropylphenyl)sulfonyl)-4-phenyl-4,5-dihydro-1*H*-pyrazole-1-carboxamide (6c)

To a solution of compound 3-ethyl-4-phenyl-4,5-dihydro-1*H*-pyrazole **5** (0.52 g, 3.0 mmol) in toluene (5 mL), compound methyl ((4-(isopropyl)phenyl)sulfonyl)carbamate[2,5] (0.93 g, 3.6 mmol), was added, and the resulting mixture was refluxed for 3 h. The reaction mixture was then cooled to room temperature and toluene was evaporated in vacuo. The residue was extracted into dichloromethane, washed with brine water and purified by flash chromatography (30-40% EtOAc in hexanes) to yield compound **6c** (0.73 g, 61%) as a colorless viscous oil.

^{1}H NMR (400 MHz, $CDCl_3$): δ 8.63 (s, 1H), 8.04 (d, *J* = 8.2 Hz, 2H), 7.41–7.24 (m, 5H), 7.07 (d, *J* = 7.3 Hz, 2H), 4.22–4.11 (m, 2H), 3.83–3.73 (m, 1H), 2.97 (hept, *J* = 6.9 Hz, 1H), 2.20–2.00 (m, 2H), 1.25 (d, *J* = 6.9 Hz, 6H), 1.04 (t, *J* = 7.5 Hz, 3H). ^{13}C NMR (100 MHz, $CDCl_3$): δ 163.4, 155.0, 147.9, 138.4, 136.5, 129.2, 128.4, 128.0, 127.4, 126.9, 53.9, 52.2, 34.2, 23.6, 21.8, 10.3. LRMS: 400.1, HRMS (ESI) m/z: $[M+H]^+$ Calculated for ($C_{21}H_{26}N_3O_3S$) 400.1695; Found 400.1692.

3-Ethyl-*N*-(naphthalen-2-ylsulfonyl)-4-phenyl-4,5-dihydro-1*H*-pyrazole-1-carboxamide (6d)

To a solution of compound **5** (0.52 g, 3.0 mmol) in toluene (5 mL), methyl ((2-(naphthyl)sulfonyl)carbamate[2,5] (0.96 g, 3.6 mmol), was added, and the resulting mixture was refluxed for 3 h. The reaction mixture was then cooled to room temperature and toluene was evaporated in vacuo. The residue was extracted into dichloromethane, washed with brine water

and purified by flash chromatography (25-35% EtOAc in hexanes) to yield compound **6d** (0.66 g, 55%) as a colorless viscous oil.

^{1}H NMR (400 MHz, $CDCl_3$): δ 8.77–8.69 (m, 2H), 8.13–8.07 (m, 1H), 8.03–7.95 (m, 2H), 7.90 (d, *J* = 8.1 Hz, 1H), 7.68–7.55 (m, 2H), 7.34–7.23 (m, 3H), 7.10–7.03 (m, 2H), 4.20–4.10 (m, 2H), 3.81–3.71 (m, 1H), 2.21–2.01 (m, 2H), 1.06 (t, *J* = 7.4 Hz, 3H). ^{13}C NMR (100 MHz, $CDCl_3$): δ 163.5, 147.8, 138.4, 136.0, 135.3, 131.9, 130.3, 129.6, 129.3, 129.1, 129.0, 128.0, 127.8, 127.4(2), 127.3(9), 123.1, 54.0, 52.2, 21.8, 10.3. LRMS: 408.1

HRMS (ESI) m/z: $[M+H]^+$ Calculated for ($C_{22}H_{22}N_3O_3S$) 408.1382; Found 408.1385.

3-(4-Chlorophenyl)-*N*-((4-trifluoromethylphenyl)sulfonyl)-4-phenyl-5,6-dihydropyridazine-1(4*H*)-carboxamide (8a)[7,8]

3-(4-Chlorophenyl)-*N*-(naphthalen-2-ylsulfonyl)-4-phenyl-5,6-dihydropyridazine-1(4*H*)-carboxamide (8b)[3]

3-(4-chlorophenyl)-4-phenyl-N-(phenylsulfonyl)-5,6-dihydropyridazine-1(4H)-carboxamide (8c)

Using general procedure B, amino component (3-(4-chlorophenyl)-4-phenyl-1,4,5,6-tetrahydropyridazine)[7] (0.4 g, 3.69 mmol) and methyl (phenylsulfonyl)-carbamate[2,3] (1.2 eq) afforded **8c** as a white solid (0.54 g, 79%). ^{1}H NMR (400 MHz, $CDCl_3$) δ 9.27 (s, 1H), 8.19 (d, *J* = 7.7 Hz, 2H), 7.66 (t, *J* = 7.4 Hz, 1H), 7.61 – 7.50 (m, 4H), 7.35 – 7.27 (m, 4H), 7.24 (d, *J* = 7.1 Hz, 1H), 7.07 (d, *J* = 7.1 Hz, 2H), 4.17 (d, *J* = 6.9 Hz, 2H), 3.03 (td, *J* = 12.8, 4.9 Hz, 1H), 2.19 – 2.03 (m, 2H). ^{13}C NMR (101 MHz, $CDCl_3$) δ 149.67, 147.36, 140.43, 139.15, 135.88, 134.21, 133.88, 129.33, 129.03, 129.01, 128.64, 127.96, 127.66, 127.49, 38.25, 35.13, 25.82. LRMS 454.1, HRMS (ESI) m/z: [M+H]+ ($C_{23}H_{21}ClN_3O_3S$) 454.0992; Found 454.0988.

3-(4-chlorophenyl)-4-phenyl-N-((2-(trifluoromethyl)phenyl)sulfonyl)-5,6-dihydropyridazine-1(4H)-carboxamide (8d)

Using general procedure B, amino component (3-(4-chlorophenyl)-4-phenyl-1,4,5,6-tetrahydropyridazine)[7] (0.4 g, 3.69 mmol) and methyl ((2-trifluoromethylphenylsulfonyl)-carbamate[2,3] (1.2 eq) afforded **8d** as a white solid (0.66 g, 85%). ^{1}H NMR (400 MHz, $CDCl_3$) δ 9.49 (s, 1H), 8.59 (d, *J* = 7.2 Hz, 1H), 7.92 (d, *J* = 6.9 Hz, 1H), 7.83 – 7.75 (m, 2H), 7.52 (d, *J* = 8.7 Hz, 2H), 7.36 – 7.28 (m, 4H), 7.25 (d, *J* = 3.9 Hz, 1H), 7.07 (d, *J* = 7.7 Hz, 2H), 4.22 – 4.10

(m, 2H), 3.01 (td, *J* = 13.0, 5.4 Hz, 1H), 2.06 (d, *J* = 12.1 Hz, 2H). ^{13}C NMR (101 MHz, $CDCl_3$) δ 149.29, 147.72, 140.32, 137.35, 136.00, 134.17, 134.13, 133.93, 132.56, 129.36, 127.94, 127.68, 127.42, 38.21, 35.16, 25.81. LRMS 522.1, HRMS (ESI) m/z: [M+H]+ ($C_{24}H_{20}ClF_3N_3O_3S$) 522.0866; Found 522.0863.

3-(4-chlorophenyl)-*N*-((4-fluorophenyl)sulfonyl)-4-phenyl-5,6-dihydropyridazine-1(4*H*)-carboxamide (8e)

Following procedure B, obtained carbamate **8e** (0.73 g, 90%) as a white solid. ^{1}H NMR (400 MHz, $CDCl_3$) δ 9.26 (s, 1H), 8.20 (td, J = 7.1, 3.7 Hz, 2H), 7.53 (dd, J = 8.7, 2.3 Hz, 2H), 7.35 – 7.27 (m, 4H), 7.26 – 7.19 (m, 3H), 7.07 (d, J = 7.3 Hz, 2H), 4.22 – 4.14 (m, 2H), 3.04 (td, J = 12.8, 5.8 Hz, 1H), 2.16 – 2.02 (m, 2H). ^{13}C NMR (101 MHz, $CDCl_3$) δ 167.18, 164.62, 149.66, 147.60, 140.37, 135.94, 135.09, 135.06, 134.16, 131.63, 131.60, 129.35, 129.01, 127.95, 127.69, 127.50, 116.20, 38.25, 35.15, 25.80. LRMS 472.1, HRMS (ESI) m/z: [M + H] + Calcd for ($C_{23}H_{20}N_3O_3FSCl$) 472.0898; Found 472.0891.

Methyl 3-(4-chlorophenyl)-4-phenyl-*N*-((4-(trifluoromethyl)phenyl)sulfonyl)-4,5-dihydro-1*H*-pyrazole-1-carbimidoselenoate (4a)

^{1}H-NMR (800 MHz, $CDCl_3$): δ 8.07 (d, *J* = 7.9 Hz, 2H), 7.72 (d, *J* = 7.9 Hz, 2H), 7.57 (d, *J* = 8.0 Hz, 2H), 7.31 (d, *J* = 7.1 Hz, 2H), 7.26 (t, *J* = 7.9 Hz, 4H), 7.13 (d, *J* = 7.5 Hz, 2H), 4.91-4.89 (m, 1H), 4.82 (dd, *J* = 11.0, 5.2 Hz, 1H), 4.48-4.47 (m, 1H), 2.25 (s, 3H). ^{13}C NMR (200 MHz; $CDCl_3$): δ 160.7, 160.3, 147.1, 138.7, 137.5, 133.7, 133.5, 129.9, 129.8, 129.7, 129.2, 128.5, 127.9, 127.6, 127.5, 126.9, 125.9, 124.3, 122.9, 60.0, 52.6, 10.0. LRMS 586.0, HRMS (ESI) m/z: $[M+H]^+$ ($C_{24}H_{20}ClF_3N_3O_2SSe$) calcd. 586.0082; Found 586.0076.

A 25 mg batch of racemic compound **4a** was separated using chiral prep HPLC (R,R-WhelkO1-25 cm x 21.1mm) to afford approximately 10.9 mg and 9.4 mg each of the enantiomers (87% and 75% respectively).

(-)-4a

^{1}H-NMR (400 MHz, $CDCl_3$): δ 8.08 (d, *J* = 7.7 Hz, 2H), 7.73 (d, *J* = 7.7 Hz, 2H), 7.58 (d, *J* = 7.7 Hz, 2H), 7.32-7.27 (m, 5H), 7.14 (d, *J* = 6.8 Hz, 2H), 4.92 (t, *J* = 11.3 Hz, 1H), 4.83 (dd, *J* = 11.3, 2.9 Hz, 1H), 4.48 (d, *J* = 9.4 Hz, 1H), 2.25 (s, 3H). ^{13}C-NMR (100 MHz, $CDCl_3$): δ 160.68, 160.27, 146.89, 138.64, 137.41, 133.67, 133.34, 129.79, 129.25, 129.19, 128.42, 127.82, 127.40, 126.91, 125.88, 125.84, 125.81, 125.77, 124.89, 122.17, 59.95, 52.53, 10.02. LRMS 586.0, HRMS (ESI) m/z: $[M+H]^+$ ($C_{24}H_{20}ClF_3N_3O_2SSe$) calcd. 586.0082; Found 586.0076.

(-)-4a: $[\alpha_D^{25}]$= -92.3°, c= 0.13 $CHCl_3$.

(+)-4a: $[\alpha_D^{25}]$= +73.2°, c= 0.4 $CHCl_3$.

Methyl 3-(4-chlorophenyl)-4-phenyl-*N*-((4-(trifluoromethoxy)phenyl)sulfonyl)-4,5-dihydro-1*H*-pyrazole-1-carbimidoselenoate (4b)

Using the general procedure, compound **1b** (50 mg, 0.09 mmol) and MeI (0.09 mL, 0.14 mmol) gave 28 mg (48.8%) of **4b.**

^{1}H-NMR (400 MHz, $CDCl_3$): δ 8.00 (d, *J* = 8.5 Hz, 2H), 7.57 (d, *J* = 8.3 Hz, 2H), 7.32-7.29 (m, 7H), 7.14 (d, *J* = 7.2 Hz, 2H), 4.91 (t, *J* = 11.6 Hz, 1H), 4.82 (dd, *J* = 11.3, 4.6 Hz, 1H), 4.50-4.47 (m, 1H), 2.26 (s, 3H).^{13}C-NMR (100 MHz, $CDCl_3$): δ 160.03, 151.48, 141.95, 138.70, 137.34, 129.77, 129.21, 129.18, 128.50, 128.39, 127.89, 127.42, 120.61, 59.94, 52.50, 10.02. LRMS 602.0, HRMS (ESI) m/z: $[M+H]^+$ ($C_{24}H_{20}ClF_3N_3O_3SSe$) 602.0031; Found 602.0042.

Methyl 3-(4-chlorophenyl)-4-phenyl-*N*-((2-(trifluoromethyl)phenyl)sulfonyl)-4,5-dihydro-1*H*-pyrazole-1-carbimidoselenoate (4c)

Using the general procedure, compound **1c** (130 mg, 0.25 mmol) and MeI (0.02 mL, 0.38 mmol) gave 67 mg (44.7%) of **4c.**

^{1}H-NMR (800 MHz, $CDCl_3$): δ 7.98 (t, *J* = 7.2 Hz, 1H), 7.59 (d, *J* = 8.1 Hz, 2H), 7.32 (t, *J* = 7.4 Hz, 2H), 7.28 (d, *J* = 7.1 Hz, 3H), 7.15 (d, *J* = 7.5 Hz, 2H), 6.96-6.91 (m, 2H), 4.93 (s, 1H), 4.84 (dd, *J* = 11.2, 5.4 Hz, 1H), 4.49 (d, *J* = 7.2 Hz, 1H), 2.28 (s, 3H).

^{13}C-NMR (201 MHz, $CDCl_3$): δ 165.94, 164.65, 160.72, 160.41, 160.23, 159.06, 138.70, 137.35, 130.58, 129.77, 129.26, 129.18, 128.38, 127.91, 127.47, 111.15, 111.04, 105.61, 105.47, 105.36, 59.95, 52.57, 9.82. LRMS 586.0, HRMS (ESI) m/z: $[M+H]^+$ ($C_{24}H_{20}ClF_3N_3O_2SSe$) 586.0082; Found 586.0080.

Methyl 3-(4-chlorophenyl)-4-phenyl-*N*-((3-(trifluoromethyl)phenyl)sulfonyl)-4,5-dihydro-1*H*-pyrazole-1-carbimidoselenoate (4d)

Using the general procedure, compound **1d** (95 mg, 0.18 mmol) gave 47 mg (43 %) of **4d.**

^{1}H-NMR (800 MHz, $CDCl_3$): δ 8.27 (s, 1H), 8.16 (d, *J* = 7.7 Hz, 1H), 7.78 (d, *J* = 7.5 Hz, 1H), 7.63-7.59 (m, 3H), 7.34 (t, *J* = 7.5 Hz, 2H), 7.28 (t, *J* = 8.2 Hz, 3H), 7.16 (d, *J* = 7.3 Hz, 2H), 4.93 (t, *J* = 10.9 Hz, 1H), 4.86 (dd, *J* = 11.2, 5.3 Hz, 1H), 4.52 (d, *J* = 6.9 Hz, 1H), 2.26 (s, 3H).

^{13}C-NMR (201 MHz, $CDCl_3$): δ 160.74, 160.34, 144.71, 138.69, 137.46, 131.19, 129.84, 129.66, 129.41, 129.30, 129.22, 128.46, 127.91, 127.44, 124.23, 123.75, 122.88, 60.03, 52.61, 9.97. LRMS 586.0, HRMS (ESI) m/z: $[M+H]^+$ ($C_{24}H_{20}ClF_3N_3O_2SSe$) 586.0082; Found 586.0076.

Methyl-3-(4-chlorophenyl)-*N*-((3-chlorophenyl)sulfonyl)-4-phenyl-4,5-dihydro-1*H*-pyrazole-1-carbimidoselenoate (4e)

Using the general procedure, compound **1e** (77 mg, 0.16 mmol) and MeI (15 μL, 0.24 mmol) gave 31 mg (35%) of **4e.**

^{1}H-NMR (400 MHz, $CDCl_3$): δ 7.95 (s, 1H), 7.84 (d, *J* = 7.7 Hz, 1H), 7.58 (d, *J* = 8.2 Hz, 2H), 7.47 (d, *J* = 7.9 Hz, 1H), 7.40 (t, *J* = 7.9 Hz, 1H), 7.31 (dd, *J* = 17.1, 9.9 Hz, 5H), 7.14 (d, *J* = 7.3 Hz, 2H), 4.94-4.81 (m, 2H), 4.48 (dd, *J* = 11.4, 3.8 Hz, 1H), 2.26 (s, 3H).

^{13}C-NMR (100 MHz, $CDCl_3$): δ 160.1, 145.2, 138.7, 137.3, 134.7, 131.9, 129.96, 129.77, 129.23, 129.17, 128.4, 127.9, 127.4, 126.7, 124.5, 59.9, 52.5, 10.0 LRMS 552.0, HRMS (ESI) m/z: $[M+H]^+$ ($C_{23}H_{20}Cl_2N_3O_2SSe$) 551.9819; Found 551.9814.

Methyl-3-(4-chlorophenyl)-*N*-((4-chlorophenyl)sulfonyl)-4-phenyl-4,5-dihydro-1*H*-pyrazole-1-carbimidoselenoate (4f)

Using the general procedure, compound **1f** (75 mg, 0.16mmol) and MeI (15 μL, 0.24 mmol) gave 42 mg (50 %) of **4f.**

^{1}H-NMR (800 MHz, $CDCl_3$): δ 7.88 (d, *J* = 8.4 Hz, 2H), 7.57 (d, *J* = 8.4 Hz, 2H), 7.42 (d, *J* = 8.5 Hz, 2H), 7.31 (t, *J* = 7.4 Hz, 2H), 7.26-7.24 (m, 3H), 7.13 (d, *J* = 7.2 Hz, 2H), 4.90 (t, *J* = 11.1 Hz, 1H), 4.82 (dd, *J* = 11.2, 5.3 Hz, 1H), 4.47 (d, *J* = 6.8 Hz, 1H), 2.24 (s, 3H). ^{13}C-NMR (100 MHz, $CDCl_3$): δ 160.30, 160.01, 142.18, 138.77, 138.15, 137.35, 129.81, 129.25, 128.93, 128.42, 127.95, 127.47, 60.00, 52.55, 10.05. LRMS 552.0, HRMS (ESI) m/z: $[M+H]^+$ ($C_{23}H_{20}Cl_2N_3O_2SSe$) 551.9819; Found 551.9811.

Methyl -(*R*)-3-(4-chlorophenyl)-*N*-((4-chlorophenyl)sulfonyl)-4-phenyl-4,5-dihydro-1*H*-pyrazole-1-carbimidoselenoate (4g)

Using the general procedure, compound **1c** (60 mg, 0.13 mmol) and MeI (12 µL, 0.19 mmol) gave 33 mg (47%) of **4g.**

^{1}H-NMR (400 MHz, $CDCl_3$): δ 7.89 (d, *J* = 8.3 Hz, 2H), 7.57 (d, *J* = 8.3 Hz, 2H), 7.43 (d, *J* = 8.3 Hz, 2H), 7.31 (d, *J* = 7.2 Hz, 3H), 7.27 (s, 2H), 7.14 (d, *J* = 7.0 Hz, 2H), 4.91 (t, *J* = 11.5 Hz, 1H), 4.82 (dd, *J* = 11.1, 4.5 Hz, 1H), 4.48 (dd, *J* = 11.1, 3.4 Hz, 1H), 2.24 (s, 3H).

^{13}C-NMR (100 MHz, $CDCl_3$): δ 159.9, 156.2, 142.1, 138.7, 137.3, 129.8, 129.20, 129.16, 128.9, 128.4, 127.9, 127.4, 59.9, 52.5, 10.0. LRMS 552.0, HRMS (ESI) m/z: $[M+H]^+$ ($C_{23}H_{20}Cl_2N_3O_2SSe$) 551.9819; Found 551.9810. [α_D^{25}]= -106.4°, c= 0.11 $CHCl_3$.

Methyl -(*S*)-3-(4-chlorophenyl)-*N*-((4-chlorophenyl)sulfonyl)-4-phenyl-4,5-dihydro-1*H*-pyrazole-1-carbimidoselenoate (4h) (70 mg gave 38 mg)

Using the general procedure, compound **1h** (70 mg, 0.15 mmol) and MeI (14 µL, 0.22 mmol) gave 39 mg (48%) of **4h.**

^{1}H-NMR (400 MHz, $CDCl_3$): δ 7.89 (d, *J* = 8.3 Hz, 2H), 7.57 (d, *J* = 8.3 Hz, 2H), 7.43 (d, *J* = 8.3 Hz, 2H), 7.31 (d, *J* = 7.2 Hz, 3H), 7.27 (s, 2H), 7.14 (d, *J* = 7.0 Hz, 2H), 4.91 (t, *J* = 11.5 Hz, 1H), 4.82 (dd, *J* = 11.1, 4.5 Hz, 1H), 4.48 (dd, *J* = 11.1, 3.4 Hz, 1H), 2.24 (s, 3H).

^{13}C-NMR (100 MHz, $CDCl_3$): δ 159.9, 156.2, 142.1, 138.7, 137.3, 129.8, 129.20, 129.16, 128.9, 128.4, 127.9, 127.4, 59.9, 52.5, 10.0. LRMS 552.0, HRMS (ESI) m/z: $[M+H]^+$ ($C_{23}H_{20}Cl_2N_3O_2SSe$) 551.9819; Found 551.9810.

LRMS 552.0, HRMS (ESI) m/z: $[M+H]^+$ ($C_{23}H_{20}Cl_2N_3O_2SSe$) 551.9819; Found 551.9810.

[α_D^{25}]= -115.3°, c= 0.11 $CHCl_3$.

Methyl 3-(4-chlorophenyl)-*N*-((4-iodophenyl)sulfonyl)-4-phenyl-4,5-dihydro-1*H*-pyrazole-1-carbimidoselenoate (4i)

Using the general procedure, compound **1c** (75 mg, 0.13 mmol) and MeI (13 μL, 0.20 mmol) gave 48 mg (53 %) of **4i.**

^{1}H-NMR (400 MHz, $CDCl_3$): δ 7.81 (d, *J* = 8.0 Hz, 2H), 7.67 (d, *J* = 8.1 Hz, 2H), 7.57 (d, *J* = 8.0 Hz, 2H), 7.32 (t, *J* = 7.1 Hz, 3H), 7.27 (s, 3H), 7.13 (d, *J* = 7.4 Hz, 2H), 4.93-4.87 (m, 1H), 4.84-4.80 (m, 1H), 4.49-4.45 (m, 1H), 2.25 (d, *J* = 5.5 Hz, 3H).

^{13}C-NMR (100 MHz, $CDCl_3$): δ 160.0, 143.3, 138.7, 137.8, 137.3, 129.8, 129.21, 129.17, 128.4, 127.96, 127.90, 127.4, 59.9, 52.5, 10.0. LRMS 643.9, HRMS (ESI) m/z: $[M+H]^+$ ($C_{23}H_{20}ClIN_3O_2SSe$) 643.9175; Found 643.9165.

Methyl -3-(4-chlorophenyl)-*N*-(naphthalen-2-ylsulfonyl)-4-phenyl-4,5-dihydro-1*H*-pyrazole-1-carbimidoselenoate (4j)

Using the general procedure, compound **1c** (95 mg, 0.19 mmol) and MeI (18 μL, 0.29 mmol) gave 55 mg (50 %) of **4j.**

^{1}H-NMR (400 MHz, $CDCl_3$): δ 8.49 (s, 1H), 7.92 (td, *J* = 16.7, 8.7 Hz, 4H), 7.58 (q, *J* = 9.0 Hz, 4H), 7.31 (t, *J* = 7.2 Hz, 2H), 7.27 (s, 2H), 7.15 (d, *J* = 7.3 Hz, 2H), 4.96 (t, *J* = 11.7 Hz, 1H), 4.83 (dd, *J* = 11.2, 5.1 Hz, 1H), 4.55 (dd, *J* = 12.0, 4.7 Hz, 1H), 2.23 (s, 3H).

^{13}C-NMR (100 MHz, $CDCl_3$): δ 160.0, 159.7, 140.5, 138.8, 137.2, 134.5, 132.1, 129.7, 129.4, 129.19, 129.14, 128.9, 128.36, 128.32, 128.01, 127.90, 127.44, 127.25, 126.8, 122.7, 60.0, 52.5, 10.0. LRMS 568.0, HRMS (ESI) m/z: $[M+H]^+$ ($C_{27}H_{23}ClN_3O_2SSe$) 568.0365; Found 568.0363.

Methyl *N*-((4-(*tert*-butyl)phenyl)sulfonyl)-3-(4-chlorophenyl)-4-phenyl-4,5-dihydro-1*H*-pyrazole-1-carbimidoselenoate (4k)

Using the general procedure, compound **1c** (85 mg, 0.17 mmol) and MeI (0.02 mL, 0.26 mmol) gave 47 mg (48 %) of **4k.**

^{1}H-NMR (400 MHz, $CDCl_3$): δ 7.87 (d, J = 8.2 Hz, 2H), 7.57 (d, J = 8.4 Hz, 2H), 7.46 (d, J = 8.2 Hz, 2H), 7.31 (t, J = 7.1 Hz, 3H), 7.26 (s, 5H), 7.14 (d, J = 7.5 Hz, 2H), 4.94 (t, J = 11.7 Hz, 1H), 4.81 (dd, J = 11.3, 5.3 Hz, 1H), 4.51 (dd, J = 12.1, 5.2 Hz, 1H), 2.28 (s, 3H), 1.32 (s, 11H).

^{13}C-NMR (100 MHz, $CDCl_3$): δ 159.7, 159.4, 155.4, 140.6, 138.9, 137.1, 129.7, 129.16, 129.12, 128.99, 128.35, 128.29, 128.1, 127.45, 127.27, 126.8, 126.18, 125.98, 125.95, 125.6, 60.0, 52.5, 35.1, 31.24, 31.20, 31.18, 31.13, 10.0. LRMS 574.1, HRMS (ESI) m/z: $[M+H]^+$ ($C_{27}H_{29}ClN_3O_2SSe$) 574.0834; Found 574.0834.

Methyl *N*-((4-bromophenyl)sulfonyl)-3-(4-chlorophenyl)-4-phenyl-4,5-dihydro-1*H*-pyrazole-1-carbimidoselenoate (4l)

Using the general procedure, compound **1l** (150 mg, 0.29 mmol) and MeI (27 μL, 0.43 mmol) gave 88 mg (52 %) of **4l.** ^{1}H-NMR (400 MHz, $CDCl_3$): δ 7.82 (d, J = 8.6 Hz, 2H), 7.58 (t, J = 8.1 Hz, 4H), 7.34-7.27 (m, 6H), 7.15-7.13 (m, 2H), 4.90 (t, J = 11.5 Hz, 1H), 4.82 (dd, J = 11.2, 4.9 Hz, 1H), 4.48 (dd, J = 11.8, 4.7 Hz, 1H), 2.25 (s, 3H).

^{13}C-NMR (100 MHz, $CDCl_3$): δ 160.0, 157.5, 138.7, 137.9, 137.3, 136.5, 132.2, 131.91, 131.84, 129.84, 129.76, 129.58, 129.21, 129.16, 128.8, 128.42, 128.37, 128.05, 127.91, 127.4, 126.6,

124.8, 116.75, 116.67, 59.9, 52.5, 10.0. LRMS 595.9, HRMS (ESI) m/z: $[M+H]^+$ ($C_{23}H_{20}BrClN_3O_2SSe$) 595.9313; Found 595.9312. The crystal structure has been deposited with CCDC. (deposition number 2483863).

Methyl -3-(4-chlorophenyl)-*N*-((4-methoxyphenyl)sulfonyl)-4-phenyl-4,5-dihydro-1*H*-pyrazole-1-carbimidoselenoate (4m)

Using the general procedure, compound **1c** (100 mg, 0.21 mmol) and MeI (20 µL, 0.32 mmol) gave 69 mg (59 %) of **4m.**

^{1}H-NMR (400 MHz, $CDCl_3$): δ 7.89 (d, *J* = 8.8 Hz, 2H), 7.58 (d, *J* = 8.5 Hz, 2H), 7.32 (t, *J* = 7.2 Hz, 3H), 7.27 (d, *J* = 5.8 Hz, 3H), 7.26 (s, 1H), 7.15 (s, 2H), 6.94 (d, *J* = 8.8 Hz, 2H), 4.93 (t, *J* = 11.7 Hz, 1H), 4.81 (dd, *J* = 11.3, 5.2 Hz, 1H), 4.51 (dd, *J* = 12.1, 5.1 Hz, 1H), 3.85 (s, 3H), 2.26 (s, 3H). ^{13}C-NMR (100 MHz, $CDCl_3$): δ 162.2, 159.4, 138.9, 137.1, 135.7, 129.7, 129.13, 129.11, 128.44, 128.27, 128.10, 127.4, 113.7, 60.0, 55.6, 52.4, 10.0. LRMS 548.0, HRMS (ESI) m/z: $[M+H]^+$ ($C_{24}H_{23}ClN_3O_3SSe$) 548.0323; Found 548.0323.

Methyl -3-(4-chlorophenyl)-*N*-((4-fluorophenyl)sulfonyl)-4-phenyl-4,5-dihydro-1*H*-pyrazole-1-carbimidoselenoate (4n)

Using the general procedure, compound **1c** (97 mg, 0.21 mmol) and MeI (20 µL, 0.31 mmol) gave 43 mg (32 %) of **4n.**

^{1}H-NMR (800 MHz, $CDCl_3$): δ 7.97 (dd, *J* = 7.6, 5.8 Hz, 2H), 7.58 (d, *J* = 8.3 Hz, 2H), 7.32 (d, *J* = 7.5 Hz, 2H), 7.27-7.25 (m, 3H), 7.15-7.12 (m, 4H), 4.92 (t, *J* = 11.3 Hz, 1H), 4.83 (dd, *J* = 11.1, 5.3 Hz, 1H), 4.49 (d, *J* = 7.4 Hz, 1H), 2.25 (s, 3H).

^{13}C-NMR (201 MHz, $CDCl_3$): δ 165.26, 164.02, 160.13, 159.90, 139.79, 138.81, 137.31, 129.81, 129.22, 129.05, 128.40, 128.02, 127.48, 115.82, 115.71, 60.00, 52.57, 10.01. LRMS 536.0, HRMS (ESI) m/z: $[M+H]^+$ ($C_{23}H_{20}ClFN_3O_2SSe$) 536.0114; Found 536.0114.

Methyl -3-(4-chlorophenyl)-4-phenyl-*N*-tosyl-4,5-dihydro-1*H*-pyrazole-1-carbimidoselenoate (4o)

Using the general procedure, compound **1o** (120 mg, 0.26 mmol) and MeI (25 μL, 0.39 mmol) gave 91 mg (65 %) of **4o.**

^{1}H-NMR (400 MHz, $CDCl_3$): δ 7.83 (d, *J* = 8.1 Hz, 2H), 7.57 (d, *J* = 8.5 Hz, 2H), 7.31 (t, *J* = 7.2 Hz, 3H), 7.26 (s, 2H), 7.14 (d, *J* = 7.2 Hz, 2H), 4.93 (t, *J* = 11.7 Hz, 1H), 4.81 (dd, *J* = 11.2, 5.2 Hz, 1H), 4.51 (dd, *J* = 12.1, 5.2 Hz, 1H), 2.40 (s, 3H), 2.25 (s, 3H).

^{13}C-NMR (100 MHz, $CDCl_3$): δ 159.7, 159.5, 142.4, 140.8, 138.8, 137.1, 129.7, 129.21, 129.15, 129.11, 128.3, 128.1, 127.4, 126.4, 60.0, 52.5, 21.6, 9.9. LRMS 532.0, HRMS (ESI) m/z: $[M+H]^+$ ($C_{24}H_{23}ClN_3O_2SSe$) 532.0365; Found 532.0369.

Methyl -3-(4-chlorophenyl)-4-phenyl-*N*-(phenylsulfonyl)-4,5-dihydro-1*H*-pyrazole-1-carbimidoselenoate (4p)

Using the general procedure, compound **1p** (98 mg, 0.22 mmol) and MeI (21 μL, 0.33 mmol) gave 52 mg (45%) of **4p.**

^{1}H-NMR (400 MHz, $CDCl_3$): δ 7.96 (d, *J* = 7.4 Hz, 2H), 7.57 (d, *J* = 7.8 Hz, 3H), 7.48 (dt, *J* = 13.1, 6.7 Hz, 4H), 7.32 (t, *J* = 7.2 Hz, 3H), 7.27 (s, 3H), 7.14 (d, *J* = 7.2 Hz, 3H), 4.95 (t, *J* = 11.6 Hz, 1H), 4.82 (dd, *J* = 11.1, 5.1 Hz, 1H), 4.52 (dd, *J* = 11.9, 4.8 Hz, 1H), 2.24 (s, 4H). ^{13}C-NMR (100 MHz, $CDCl_3$): δ 160.01, 159.68, 143.60, 138.85, 137.24, 131.87, 129.78, 129.23, 129.19, 128.67, 128.37, 128.05, 127.49, 126.41, 60.04, 52.59, 9.97.

LRMS 518.0, HRMS (ESI) m/z: $[M+H]^+$ ($C_{23}H_{21}N_3O_2SClSe$) 518.0208; Found 518.0198.

Methyl -3-(4-chlorophenyl)-*N*-((4-cyanophenyl)sulfonyl)-4-phenyl-4,5-dihydro-1*H*-pyrazole-1-carbimidoselenoate (4q)

Using the general procedure, compound **1q** (150 mg, 0.32 mmol) and MeI (30 μL, 0.48 mmol) gave 109 mg (62.7%) of **4q.**

^{1}H-NMR (400 MHz, $CDCl_3$): δ 8.06 (d, J = 7.8 Hz, 2H), 7.76 (d, J = 7.8 Hz, 2H), 7.58 (d, J = 7.8 Hz, 2H), 7.32 (d, J = 6.8 Hz, 3H), 7.28 (s, 3H), 7.14 (d, J = 7.2 Hz, 2H), 4.90-4.82 (m, 2H), 4.47-4.45 (m, 1H), 2.24 (s, 3H). ^{13}C-NMR (100 MHz, $CDCl_3$): δ 160.6, 138.6, 137.5, 132.6, 129.8, 129.28, 129.22, 128.5, 127.7, 127.4, 127.1, 117.8, 115.5, 59.9, 52.5, 10.0. LRMS 543.0, HRMS (ESI) m/z: $[M+H]^+$ ($C_{24}H_{20}ClN_4O_2SSe$) 543.0161; Found 543.0156.

Methyl -3-(4-chlorophenyl)-*N*-((2,4-difluorophenyl)sulfonyl)-4-phenyl-4,5-dihydro-1*H*-pyrazole-1-carbimidoselenoate (4r)

Using the general procedure, compound **1r** (80 mg, 0.17 mmol) and MeI (16 μL, 0.25 mmol) gave 34 mg (37%) of **4r.**

^{1}H-NMR (400 MHz, $CDCl_3$): δ 7.96 (q, *J* = 7.5 Hz, 1H), 7.58 (d, *J* = 8.1 Hz, 2H), 7.32 (t, *J* = 7.1 Hz, 2H), 7.27 (s, 3H), 7.14 (d, *J* = 7.3 Hz, 2H), 6.92 (q, *J* = 8.9 Hz, 2H), 4.93 (t, *J* = 11.6 Hz, 1H), 4.83 (dd, *J* = 11.1, 4.9 Hz, 1H), 4.47 (dd, *J* = 11.3, 3.6 Hz, 1H), 2.26 (s, 3H).

^{13}C-NMR (100 MHz, $CDCl_3$): δ 166.5, 164.07, 163.96, 161.07, 160.95, 160.7, 160.2, 158.50, 158.38, 138.7, 137.3, 130.62, 130.52, 129.8, 129.26, 129.17, 128.4, 127.9, 127.4, 111.23, 111.19, 111.01, 110.97, 105.7, 105.5, 105.2, 59.9, 52.5, 9.8. LRMS 554.0, HRMS (ESI) m/z: $[M+H]^+$ ($C_{23}H_{18}ClF_2N_3O_2SSe$) 554.0020; Found 554.0019.

Methyl -3-(4-chlorophenyl)-*N*-((4,4-difluoropiperidin-1-yl)sulfonyl)-4-phenyl-4,5-dihydro-1*H*-pyrazole-1-carbimidoselenoate (4s)

Using the general procedure, compound **1s** (150 mg, 0.31 mmol) and MeI (30 μL, 0.47 mmol) gave 79 mg (45 %) of **4s.**

^{1}H-NMR (400 MHz, $CDCl_3$): δ 7.56 (d, *J* = 8.4 Hz, 2H), 7.31 (q, *J* = 7.4 Hz, 3H), 7.26 (s, 2H), 7.13 (d, *J* = 7.2 Hz, 2H), 4.88-4.78 (m, 2H), 4.40 (dd, *J* = 10.7, 3.8 Hz, 1H), 3.37 (t, *J* = 5.4 Hz, 4H), 2.39 (s, 3H), 2.10 (tt, *J* = 13.0, 6.3 Hz, 4H).

^{13}C-NMR (100 MHz, $CDCl_3$): δ 159.7, 158.9, 138.8, 137.1, 129.7, 129.1, 128.3, 128.0, 127.4, 123.7, 121.3, 118.9, 59.4, 52.7, 44.65, 44.59, 44.53, 33.7, 33.4, 33.2, 9.7. LRMS 561.0, HRMS (ESI) m/z: $[M+H]^+$ ($C_{22}H_{24}ClF_2N_4O_2SSe$)561.0442; Found 561.0452.

Methyl-3-(4-chlorophenyl)-4-phenyl-*N*-(piperidin-1-ylsulfonyl)-4,5-dihydro-1*H*-pyrazole-1-carbimidoselenoate (4t)

Using the general procedure, compound **1t** (118 mg, 0.26 mmol) and MeI (25 μL, 0.39 mmol) gave 65 mg (47%) of **4t.**

^{1}H-NMR (400 MHz, $CDCl_3$): δ 7.55 (d, *J* = 8.4 Hz, 2H), 7.30 (t, *J* = 7.1 Hz, 3H), 7.23 (s, 2H), 7.13 (d, *J* = 7.5 Hz, 2H), 4.90 (t, *J* = 11.7 Hz, 1H), 4.78 (dd, *J* = 11.4, 5.2 Hz, 1H), 4.45 (dd, *J* = 11.9, 5.2 Hz, 1H), 3.17 (s, 5H), 2.38 (s, 3H), 1.66 (d, *J* = 4.7 Hz, 4H), 1.50 (d, *J* = 4.9 Hz, 2H).

^{13}C-NMR (100 MHz, $CDCl_3$): δ 159.0, 158.2, 139.0, 136.9, 129.7, 129.18, 129.06, 128.90, 128.23, 128.18, 127.48, 127.32, 59.5, 52.7, 48.1, 25.2, 23.9, 9.5. LRMS 525.1, HRMS (ESI) m/z: $[M+H]^+$ ($C_{22}H_{26}ClN_4O_2SSe$) 525.0630; Found 525.0634.

Methyl -3-(4-chlorophenyl)-4-phenyl-*N*-((4-(trifluoromethyl)piperidin-1-yl)sulfonyl)-4,5-dihydro-1*H*-pyrazole-1-carbimidoselenoate (4u)

Using the general procedure, compound **1u** (75 mg, 0.15 mmol) and MeI (14 μL, 0.22 mmol) gave 31 mg (36%) of **4u.**

^{1}H-NMR (400 MHz, $CDCl_3$): δ 7.55 (d, *J* = 7.8 Hz, 2H), 7.30 (d, *J* = 7.0 Hz, 5H), 7.13 (d, *J* = 7.1 Hz, 2H), 4.90-4.78 (m, 2H), 4.43 (t, *J* = 5.6 Hz, 1H), 3.85 (d, *J* = 11.2 Hz, 2H), 2.69 (t, *J* = 12.0 Hz, 2H), 2.37 (s, 3H), 2.10-2.08 (m, 1H), 1.94 (d, *J* = 12.9 Hz, 2H), 1.78-1.69 (m, 2H).

^{13}C-NMR (100 MHz, $CDCl_3$): δ 159.52, 158.72, 138.85, 137.09, 129.73, 129.11, 128.32, 128.00, 127.44, 100.00, 59.42, 52.72, 46.32, 24.02, 24.00, 9.59. LRMS 593.0, HRMS (ESI) m/z: $[M+H]^+$ ($C_{23}H_{25}ClF_3N_4O_2SSe$) 593.0504; Found 593.0511.

Methyl 3-(4-chlorophenyl)-*N*-(*N*,*N*-diethylsulfamoyl)-4-phenyl-4,5-dihydro-1*H*-pyrazole-1-carbimidoselenoate (4v)

Using the general procedure, compound **1v** (55 mg, 0.13 mmol) and MeI (12 μL, 0.19 mmol) gave 37 mg (57%) of **4v.**

^{1}H-NMR (400 MHz, CDCl$_3$): δ 7.55 (d, *J* = 8.1 Hz, 2H), 7.30 (d, *J* = 7.2 Hz, 2H), 7.24 (d, *J* = 7.5 Hz, 3H), 7.13 (d, *J* = 7.2 Hz, 2H), 4.87 (t, *J* = 11.6 Hz, 1H), 4.77 (dd, *J* = 11.3, 4.9 Hz, 1H), 4.44 (dd, *J* = 11.7, 4.9 Hz, 1H), 3.26 (q, *J* = 7.1 Hz, 4H), 2.32 (s, 3H), 1.21 (t, *J* = 12.6 Hz, 7H).

^{13}C-NMR (100 MHz, CDCl$_3$): δ 158.7, 158.0, 139.1, 136.8, 129.7, 129.05, 129.03, 128.24, 128.18, 127.5, 59.5, 52.6, 43.2, 13.8, 9.4. LRMS 513.1, HRMS (ESI) m/z: [M+H]$^+$ (($C_{21}H_{26}ClN_4O_2SSe$)) 513.0630; Found 513.0628.

Methyl -3-ethyl-4-phenyl-*N*-((4-(trifluoromethoxy)phenyl)sulfonyl)-4,5-dihydro-1*H*-pyrazole-1-carbimidoselenoate (7a)

Using the general procedure, compound **7a** (90 mg, 0.20 mmol) and MeI (19 μL, 0.31 mmol) gave 45 mg (43%) of **7a.**

^{1}H-NMR (400 MHz, CDCl$_3$): δ 8.00 (d, *J* = 7.8 Hz, 2H), 7.39–7.24 (m, 5H), 7.12 (d, *J* = 7.6 Hz, 2H), 4.85 (t, *J* = 11.6 Hz, 1H), 4.44–4.29 (m, 2H), 2.37–2.11 (m, 5H), 1.11 (t, *J* = 7.2 Hz, 3H).

^{13}C-NMR (100 MHz, CDCl$_3$): δ 168.2, 159.8, 151.2, 142.2, 137.7, 129.4, 128.3, 128.2, 127.6, 120.5, 58.4, 55.0, 22.1, 10.2, 9.6. HRMS (ESI) m/z: [M+H]$^+$ Calculated for ($C_{20}H_{21}F_3N_3O_3SSe$) 520.0421; Found 520.0413.

Methyl -3-ethyl-*N*-((4-iodophenyl)sulfonyl)-4-phenyl-4,5-dihydro-1*H*-pyrazole-1-carbimidoselenoate (7b).

Using the general procedure, compound **6b** (75 mg, 0.16 mmol) and MeI (15 μL, 0.23 mmol) gave 48 mg (55%) of **7b.**

^{1}H-NMR (400 MHz, $CDCl_3$): δ 7.80 (d, *J* = 8.2 Hz, 2H), 7.67 (d, *J* = 8.2 Hz, 2H), 7.34 (t, *J* = 7.3 Hz, 3H), 7.11 (d, *J* = 6.9 Hz, 2H), 4.83 (t, *J* = 11.6 Hz, 1H), 4.36 (dd, *J* = 20.9, 11.4 Hz, 2H), 2.26 (td, *J* = 15.6, 7.3 Hz, 1H), 2.21-2.16 (m, 4H), 1.11 (t, *J* = 7.3 Hz, 3H). ^{13}C-NMR (100 MHz, $CDCl_3$): δ 168.31, 159.88, 143.61, 137.82, 137.75, 129.54, 128.34, 127.92, 127.67, 98.68, 58.55, 55.10, 22.20, 10.34, 9.69. LRMS 562.0, HRMS (ESI) m/z: $[M+H]^+$ ($C_{19}H_{21}IN_3O_2SSe$) 561.9564; Found 561.9568.

Methyl -3-(4-chlorophenyl)-4-phenyl-*N*-((4-(trifluoromethyl)phenyl)sulfonyl)-5,6-dihydropyridazine-1(4*H*)-carbimidoselenoate (9a)

Using the general procedure, compound **8a** (100 mg, 0.19 mmol) and MeI (18 μL, 0.28 mmol) gave 61 mg (53%) of **9a.**

^{1}H-NMR (400 MHz, $CDCl_3$): δ 8.09 (d, J = 8.1 Hz, 2H), 7.73 (d, J = 7.3 Hz, 2H), 7.68 (d, J = 8.9 Hz, 2H), 7.32 (dd, J = 15.6, 9.6 Hz, 4H), 7.26 (s, 1H), 7.11 (d, J = 7.2 Hz, 2H), 4.51 (d, J = 12.6 Hz, 1H), 4.27 (d, J = 5.6 Hz, 1H), 3.44 (td, J = 14.1, 4.2 Hz, 1H), 2.35 (s, 3H), 2.29 – 2.10 (m, 2H). ^{13}C-NMR (100 MHz, $CDCl_3$): δ 167.51, 150.97, 147.36, 139.40, 136.34, 133.70, 129.34, 128.91, 127.84, 127.81, 127.70, 126.55, 125.80, 125.76, 41.56, 37.78, 26.25, 10.22. LRMS 600.0, HRMS (ESI) m/z: $[M+H]^+$ ($C_{25}H_{22}ClF_3N_3O_2SSe$) 600.0239; Found 600.0235.

Methyl -3-(4-chlorophenyl)-*N*-(naphthalen-2-ylsulfonyl)-4-phenyl-5,6-dihydropyridazine-1(4*H*)-carbimidoselenoate (9b)

Using the general procedure, compound **8b** (100 mg, 0.2 mmol) and MeI (19 μL, 0.30 mmol) gave 90 mg (78%) of **9b.**

^{1}H-NMR (400 MHz, CDCl$_3$): δ 8.51 (s, 1H), 7.99 – 7.87 (m, 4H), 7.67 (d, *J* = 10.8 Hz, 2H), 7.59 (t, *J* = 7.0 Hz, 2H), 7.36 – 7.28 (m, 4H), 7.24 (s, 1H), 7.12 (d, *J* = 5.5 Hz, 2H), 4.58 (d, *J* = 13.2 Hz, 1H), 4.25 (d, *J* = 5.3 Hz, 1H), 3.48 (td, *J* = 13.9, 4.7 Hz, 1H), 2.33 (s, 3H), 2.29 – 2.06 (m, 2H). ^{13}C-NMR (100 MHz, CDCl$_3$): δ 166.82, 150.46, 141.06, 139.72, 136.26, 134.50, 133.99, 132.18, 129.40, 128.98, 128.35, 128.00, 127.91, 127.74, 127.29, 126.46, 122.57, 41.75, 37.91, 26.43, 10.30. HRMS (ESI) m/z: [M+H]+ ($C_{28}H_{25}ClN_3O2SSe$) 582.0521; Found 582.0523.

Methyl -3-(4-chlorophenyl)-4-phenyl-*N*-(phenylsulfonyl)-5,6-dihydropyridazine-1(4*H*)-carbimidoselenoate (9c)

Using the general procedure, compound **8c** (100 mg, 0.22 mmol) and MeI (21 μL, 0.33 mmol) gave 30 mg (26%) of **9c.**

^{1}H-NMR (400 MHz, CDCl$_3$): δ 7.97 (d, *J* = 8.4 Hz, 2H), 7.67 (d, *J* = 6.9 Hz, 2H), 7.48 (td, *J* = 9.8, 6.7 Hz, 3H), 7.36 – 7.28 (m, 4H), 7.25 (s, 1H), 7.12 (d, *J* = 8.1 Hz, 2H), 4.57 (d, *J* = 13.6 Hz, 1H), 4.25 (d, *J* = 5.6 Hz, 1H), 3.46 (td, *J* = 13.3, 4.0 Hz, 1H), 2.33 (s, 3H), 2.28 – 2.08 (m, 2H). ^{13}C-NMR (100 MHz, CDCl$_3$): δ 166.79, 150.38, 144.15, 139.75, 134.02, 131.78, 129.44, 129.00, 128.70, 127.99, 127.92, 127.76, 126.15, 41.70, 37.93, 26.45, 10.28. HRMS (ESI) m/z: [M+H]+ ($C_{24}H_{23}ClN_3O_2SSe$) 532.0365; Found 532.0366.

Cyclopropylmethyl -3-(4-chlorophenyl)-4-phenyl-*N*-((4-(trifluoromethyl)phenyl)sulfonyl)-4,5-dihydro-1*H*-pyrazole-1-carbimidoselenoate (10)

Using the general procedure, compound **1a** (100 mg, 0.2 mmol) was converted in a three-step, one-pot protocol using cyclopropyl methylbromide (1.5 eq) as the alkylating agent to yield title compound **10** (48 mg, 39%).

^{1}H-NMR (800 MHz, $CDCl_3$): δ 8.09 (d, *J* = 8.1 Hz, 2H), 7.74 (d, *J* = 8.2 Hz, 2H), 7.58 (d, *J* = 8.2 Hz, 2H), 7.33 (t, *J* = 7.5 Hz, 3H), 7.28 (t, *J* = 9.3 Hz, 4H), 7.15 (d, *J* = 7.3 Hz, 2H), 4.87-4.82 (m, 2H), 4.45-4.43 (m, 1H), 2.96 (tt, *J* = 13.3, 7.1 Hz, 2H), 0.99-0.95 (m, 1H), 0.55 (d, *J* = 7.5 Hz, 2H), 0.16-0.14 (m, 2H). ^{13}C-NMR (200 MHz; $CDCl_3$): δ 160.3, 146.9, 138.7, 137.4, 133.8, 133.5, 133.2, 129.8, 129.3, 128.5, 128.0, 127.5, 127.1, 125.8, 59.8, 52.3, 36.1, 10.8, 7.1. LRMS 626.1, HRMS (ESI) m/z: $[M+H]^+$ ($C_{27}H_{24}ClN_3O_2F_3SSe$) $[M+H]^+$ 626.0395; found 626.0397.

5-Fluoropentyl -3-(4-chlorophenyl)-4-phenyl-*N*-((4-(trifluoromethyl)phenyl)sulfonyl)-4,5-dihydro-1*H*-pyrazole-1-carbimidoselenoate (11)

Using the general procedure, compound **1a** (100 mg, 0.2 mmol) was converted in a three-step, one-pot protocol using 1-bromo-5-fluoropentane (1.5 eq) as the alkylating agent to yield title compound **11** (64 mg, 49%).

^{1}H-NMR (400 MHz, $CDCl_3$): δ 8.07 (d, *J* = 8.0 Hz, 2H), 7.74 (d, *J* = 8.0 Hz, 2H), 7.58 (d, *J* = 8.0 Hz, 2H), 7.33-7.27 (m, 5H), 7.14 (d, *J* = 7.2 Hz, 2H), 4.95 (t, *J* = 11.3 Hz, 1H), 4.85 (dd, *J* = 11.0, 4.8 Hz, 1H), 4.52 (d, *J* = 8.8 Hz, 1H), 4.39 (t, *J* = 5.8 Hz, 1H), 4.27 (t, *J* = 5.8 Hz, 1H), 2.85 (t, *J* = 7.4 Hz, 2H), 1.61 (t, *J* = 7.6 Hz, 2H), 1.48 (t, *J* = 6.9 Hz, 2H), 1.26 (t, *J* = 7.0 Hz, 2H).

^{13}C-NMR (100 MHz, $CDCl_3$): δ 160.35, 146.98, 138.68, 137.48, 129.85, 129.31, 129.25, 128.48, 127.86, 127.47, 127.00, 125.88, 125.85, 84.60, 82.96, 60.05, 52.62, 30.03, 29.80, 29.22, 25.83. LRMS 660.1, HRMS (ESI) m/z: $[M+H]^+$ ($C_{28}H_{27}ClF_4N_3O_2SSe$) 660.0614; Found 660.0620.

2-Amino-2-oxoethyl -3-(4-chlorophenyl)-4-phenyl-*N*-((4-(trifluoromethyl)phenyl)sulfonyl)-4,5-dihydro-1*H*-pyrazole-1-carbimidoselenoate (12)

Using the general procedure, compound **1a** (150 mg, 0.3 mmol) was converted in a three-step, one-pot protocol using 2-bromoacetamide (1.5 eq) as the alkylating agent to yield title compound **12** (89 mg, 48%).

^{1}H-NMR (800 MHz, $CDCl_3$): δ 8.08 (d, *J* = 8.0 Hz, 2H), 7.78 (d, *J* = 8.1 Hz, 2H), 7.58 (d, *J* = 8.1 Hz, 2H), 7.35 (t, *J* = 7.4 Hz, 3H), 7.30 (dd, *J* = 12.3, 8.0 Hz, 4H), 7.16 (d, *J* = 7.5 Hz, 2H), 6.23 (s, 1H), 5.18 (s, 1H), 4.99 (t, *J* = 11.7 Hz, 1H), 4.91 (dt, *J* = 8.2, 4.0 Hz, 1H), 4.56-4.54 (m, 1H), 3.51 (s, 2H). ^{13}C-NMR (100 MHz, $CDCl_3$): δ 171.47, 161.49, 138.50, 138.14, 130.13, 129.65, 129.54, 128.82, 127.65, 127.54, 127.17, 126.41, 60.43, 53.29, 30.44. LRMS 629.0, HRMS (ESI) m/z: $[M+H]^+$ ($C_{25}H_{21}ClF_3N_4O_3SSe$) 629.0152; Found 629.0512.

A 20 mg batch of racemic compound **12** was separated using chiral prep HPLC (R,R-WhelkO1-25 cm x 21.1mm) to afford approximately 8.1 mg and 7.0 mg each of the enantiomers (81% and 70% respectively)

(-)-12

^{1}H-NMR (500 MHz, $CDCl_3$): δ 8.07 (d, *J* = 7.8 Hz, 2H), 7.76 (d, *J* = 7.8 Hz, 2H), 7.57 (d, *J* = 7.7 Hz, 2H), 7.33 (d, *J* = 6.9 Hz, 3H), 7.26 (d, *J* = 6.5 Hz, 2H), 7.15 (d, *J* = 6.9 Hz, 2H), 6.22 (s, 1H), 5.31 (s, 1H), 4.98 (t, *J* = 11.3 Hz, 1H), 4.90 (d, *J* = 13.1 Hz, 1H), 4.53 (d, *J* = 11.3 Hz, 1H), 3.50 (s, 2H). ^{13}C-NMR (100 MHz, $CDCl_3$): δ 171.35, 161.27, 158.59, 146.14, 138.27, 137.91, 134.30, 133.97, 129.89, 129.44, 129.30, 128.59, 127.41, 127.29, 126.93, 126.23, 126.20, 126.16, 126.13, 124.71, 122.00, 60.19, 53.05, 30.24; LRMS 629.0, HRMS (ESI) m/z: $[M+H]^+$ ($C_{25}H_{21}ClF_3N_4O_3SSe$) 629.0152; Found 629.0512.

(-)-12: $[\alpha_D^{25}]$= -121°, c= 0.1 $CHCl_3$.

(+)-12: [α_D^{25}]= +68.5°, c= 0.07 $CHCl_3$.

2-Methoxyethyl -3-(4-chlorophenyl)-4-phenyl-*N*-((4-(trifluoromethyl)phenyl)sulfonyl)-4,5-dihydro-1*H*-pyrazole-1-carbimidoselenoate (13)

Using the general procedure, compound **1a** (100 mg, 0.2 mmol) was converted in a three-step, one-pot protocol using 1-bromo-2-methoxyethane (1.5 eq) as the alkylating agent to yield title compound **13** (68 mg, 55%).

^{1}H-NMR (400 MHz, $CDCl_3$): δ 8.08 (d, *J* = 8.1 Hz, 2H), 7.74 (d, *J* = 8.2 Hz, 2H), 7.58 (d, *J* = 8.4 Hz, 2H), 7.32 (q, *J* = 7.3 Hz, 4H), 7.28 (d, *J* = 7.2 Hz, 3H), 7.15 (d, *J* = 7.2 Hz, 2H), 4.97 (dd, *J* = 15.4, 7.9 Hz, 1H), 4.85 (dd, *J* = 11.1, 5.1 Hz, 1H), 4.55-4.51 (m, 1H), 3.50 (t, *J* = 6.4 Hz, 2H), 3.19 (s, 3H), 3.04 (q, *J* = 5.3 Hz, 2H).

^{13}C-NMR (100 MHz, $CDCl_3$): δ 160.3, 138.6, 137.5, 129.8, 129.30, 129.19, 128.4, 127.8, 127.4, 127.0, 125.85, 125.81, 125.78, 71.4, 60.1, 58.6, 52.7, 28.5. LRMS 630.0, HRMS (ESI) m/z: $[M+H]^+$ ($C_{26}H_{24}ClF_3N_3O_3SSe$) 630.0344; Found 630.0341.

2-Cyanopropyl -3-(4-chlorophenyl)-4-phenyl-*N*-((4-(trifluoromethyl)phenyl)sulfonyl)-4,5-dihydro-1*H*-pyrazole-1-carbimidoselenoate (14)

Using the general procedure, compound **1a** (125 mg, 0.25 mmol) was converted in a three-step, one-pot protocol using 4-bromobutanenitrile (1.5 eq) as the alkylating agent to yield title compound **14** (72 mg, 46%).

^{1}H-NMR (400 MHz, $CDCl_3$): δ 8.07 (d, *J* = 8.0 Hz, 2H), 8.07 (d, *J* = 8.0 Hz, 2H), 7.76 (d, *J* = 8.0 Hz, 2H), 7.76 (d, *J* = 8.0 Hz, 2H), 7.57 (d, *J* = 8.2 Hz, 2H), 7.57 (d, *J* = 8.2 Hz, 2H), 7.33-7.28 (m, 5H), 7.33-7.28 (m, 5H), 7.15 (d, *J* = 7.3 Hz, 2H), 7.15 (d, *J* = 7.3 Hz, 2H), 4.97 (t, *J* = 11.4 Hz,

1H), 4.97 (t, J = 11.4 Hz, 1H), 4.88 (dd, J = 10.7, 5.0 Hz, 1H), 4.88 (dd, J = 10.7, 5.0 Hz, 1H), 4.53 (d, J = 8.2 Hz, 1H), 4.53 (d, J = 8.2 Hz, 1H), 2.92 (t, J = 7.1 Hz, 2H), 2.92 (t, J = 7.1 Hz, 2H), 2.21 (t, J = 6.9 Hz, 2H), 2.21 (t, J = 6.9 Hz, 2H), 1.91 (t, J = 6.9 Hz, 2H), 1.91 (t, J = 6.9 Hz, 2H).
^{13}C-NMR (100 MHz, $CDCl_3$): δ 160.7, 158.5, 146.7, 138.5, 137.6, 134.0, 133.6, 129.8, 129.33, 129.24, 128.5, 127.62, 127.50, 127.43, 126.9, 126.08, 126.04, 126.01, 125.97, 124.8, 122.1, 118.7, 60.0, 52.8, 27.2, 25.7, 17.1. LRMS 639.0, HRMS (ESI) m/z: $[M+H]^+$ (($C_{27}H_{23}ClF_3N_4O_2SSe$)) 639.0348; Found 639.0359.

2-(2-Methoxyethoxy)ethyl -3-(4-chlorophenyl)-4-phenyl-*N*-((4-(trifluoromethyl)phenyl)sulfonyl)-4,5-dihydro-1*H*-pyrazole-1-carbimidoselenoate (15)

Using the general procedure, compound **1a** (150 mg, 0.3 mmol) was converted in a three-step, one-pot protocol using 1-bromo-2-(2-methoxyethoxy)ethane (1.5 eq) as the alkylating agent to yield title compound **15** (76 mg, 38%).

^{1}H-NMR (400 MHz, $CDCl_3$): δ 8.08 (d, J = 7.4 Hz, 2H), 7.74 (d, J = 7.2 Hz, 2H), 7.57 (d, J = 6.8 Hz, 2H), 7.30 (dd, J = 14.9, 5.9 Hz, 5H), 7.15 (d, J = 6.6 Hz, 2H), 4.97 (t, J = 11.1 Hz, 1H), 4.85 (d, J = 11.2 Hz, 1H), 4.54 (d, J = 9.3 Hz, 1H), 3.59 (d, J = 6.4 Hz, 2H), 3.42 (s, 2H), 3.39 (s, 2H), 3.34 (d, J = 2.3 Hz, 3H), 3.06 (t, J = 5.4 Hz, 2H).

^{13}C-NMR (100 MHz, $CDCl_3$): δ 160.3, 159.8, 146.8, 138.6, 137.5, 134.1, 133.7, 133.4, 133.1, 129.8, 129.31, 129.18, 128.4, 127.8, 127.4, 127.0, 125.93, 125.89, 125.86, 125.82, 124.9, 122.2, 71.8, 70.26, 70.10, 60.1, 59.1, 52.7, 28.3, 25.4. LRMS 674.1, HRMS (ESI) m/z: $[M+H]^+$ ($C_{28}H_{28}ClF_3N_3O_4SSe$) 674.0606; Found 674.0618.

2,2,2-Trifluoroethyl -3-(4-chlorophenyl)-4-phenyl-*N*-((4-(trifluoromethyl)phenyl)sulfonyl)-4,5-dihydro-1*H*-pyrazole-1-carbimidoselenoate (16)

Using the general procedure, compound **1a** (75 mg, 0.15 mmol) was converted in a three-step, one-pot protocol using 1,1,1-trifluoro-2-iodoethane (1.5 eq) as the alkylating agent to yield title compound **16** (42 mg, 44%).

^{1}H-NMR (400 MHz, $CDCl_3$): δ 8.06 (d, *J* = 8.2 Hz, 2H), 7.76 (d, *J* = 8.2 Hz, 2H), 7.58 (d, *J* = 8.5 Hz, 2H), 7.32 (dt, *J* = 21.9, 7.3 Hz, 5H), 7.17 (d, *J* = 7.2 Hz, 2H), 5.12 (t, *J* = 11.7 Hz, 1H), 4.94 (dd, *J* = 11.2, 5.6 Hz, 1H), 4.66 (dd, *J* = 12.2, 5.5 Hz, 1H), 3.37 (dd, *J* = 10.6, 4.1 Hz, 2H).

^{13}C-NMR (100 MHz, $CDCl_3$): δ 160.8, 156.4, 146.1, 138.3, 137.9, 129.9, 129.50, 129.43, 129.34, 129.29, 129.23, 128.6, 127.45, 127.28, 126.9, 125.96, 125.92, 125.88, 125.6, 77.3, 60.4, 53.4. LRMS 653.9, HRMS (ESI) m/z: $[M+H]^+$ (LRMS 524.1, HRMS (ESI) m/z: $[M+H]^+$ ($C_{25}H_{19}ClF_6N_3O_2SSe$) 653.9956; Found 653.9959.

But-3-yn-1-yl)-3-(4-chlorophenyl)-4-phenyl-*N*-((4-(trifluoromethyl)phenyl)sulfonyl)-4,5-dihydro-1*H*-pyrazole-1-carbimidoselenoate (17)

Following General procedure A, Compound (urea) **1a** (100 mg, 0.20 mmol) was converted in a three-step, one-pot protocol using 4-bromo-1-butyne (1.5 eq) as the alkylating agent to title compound **17** (46 mg, 38% yield).

^{1}H-NMR (800 MHz, $CDCl_3$): δ 8.07 (d, *J* = 7.7 Hz, 2H), 7.74 (d, *J* = 7.9 Hz, 2H), 7.57 (d, *J* = 7.6 Hz, 3H), 7.32 (s, 3H), 7.27 (s, 4H), 7.15 (d, *J* = 6.8 Hz, 3H), 5.01 (s, 1H), 4.88 (d, *J* = 5.0 Hz, 1H), 4.58 (d, *J* = 0.5 Hz, 1H), 4.10 (d, *J* = 7.0 Hz, 1H), 2.92 (d, *J* = 3.9 Hz, 2H), 2.45 (s, 2H), 2.02 (s, 1H), 1.91 (s, 1H), 1.28-1.18 (m, 2H). ^{13}C-NMR (200 MHz; $CDCl_3$): δ 160.5, 159.4, 146.7, 138.6, 137.6, 129.9, 129.41, 129.26, 128.5, 127.7, 127.5, 126.9, 126.1, 82.7, 69.6, 60.2, 53.0, 27.1, 19.8. LRMS 624.1, HRMS ($C_{27}H_{22}ClN_3O_2F_3SSe$) $[M+H]^+$ found m/z 624.0239, calcd 624.0239.

2-(2-(2-Azidoethoxy)ethoxy)ethyl -3-(4-chlorophenyl)-4-phenyl-*N*-((4-(trifluoromethyl)phenyl)sulfonyl)-4,5-dihydro-1*H*-pyrazole-1-carbimidoselenoate (18)

Using the general procedure, compound **1a** (50 mg, 0.1 mmol) was converted in a three-step, one-pot protocol using 1-azido-2-(2-bromoethoxy)ethane (1.5 eq) as the alkylating agent to yield title compound **18** (29 mg, 43%).

^{1}H-NMR (400 MHz, $CDCl_3$): δ 8.09 (d, *J* = 7.9 Hz, 2H), 7.76 (d, *J* = 7.8 Hz, 2H), 7.59 (d, *J* = 8.1 Hz, 2H), 7.33 (d, *J* = 6.9 Hz, 3H), 7.29 (s, 2H), 7.16 (d, *J* = 6.9 Hz, 2H), 5.02-4.96 (m, 1H), 4.87 (dt, *J* = 11.2, 2.1 Hz, 1H), 4.57-4.53 (m, 1H), 3.62 (t, *J* = 6.1 Hz, 2H), 3.43 (s, 2H), 3.29 (s, 2H), 3.08-3.05 (m, 2H). ^{13}C-NMR (100 MHz, $CDCl_3$): δ 160.40, 159.76, 146.84, 138.57, 137.51, 133.77, 133.44, 130.24, 130.21, 130.05, 129.99, 129.75, 129.69, 129.64, 129.61, 129.59, 129.56, 129.37, 129.29, 128.93, 128.85, 128.76, 127.99, 127.97, 127.94, 127.92, 127.89, 127.85, 127.71, 127.03, 126.92, 125.89, 125.87, 124.85, 122.14, 70.17, 69.61, 69.60, 50.68, 28.24. LRMS 685.1, HRMS (ESI) m/z: $[M+H]^+$ ($C_{27}H_{25}ClF_3N_6O_3SSe$) 685.0515; Found 685.0523.

Methyl (2*R*)-3-((3-(4-chlorophenyl)-4-phenyl-4,5-dihydro-1*H*-pyrazol-1yl)-(4(trifluoro-methyl)phenyl)sulfonyl)imino)methyl)selanyl)-2-methylpropanoate (19)

Using the general procedure, compound **1a** (100 mg, 0.2 mmol) was converted in a three-step, one-pot protocol using methyl (*R*)-3-bromo-2-methylpropanoate (1.5 eq) as the alkylating agent to yield title compound **19** (51 mg, 39%).

^{1}H-NMR (400 MHz, $CDCl_3$): δ 8.08 (d, *J* = 8.1 Hz, 2H), 7.75 (d, *J* = 8.2 Hz, 2H), 7.57 (d, *J* = 8.3 Hz, 2H), 7.33-7.27 (m, 5H), 7.16 (d, *J* = 7.0 Hz, 2H), 5.02 (t, *J* = 8.1 Hz, 1H), 4.87 (dq, *J* = 8.5,

2.6 Hz, 1H), 4.59 (t, J = 5.7 Hz, 1H), 3.61 (d, J = 4.9 Hz, 4H), 3.03-2.97 (m, 1H), 2.82 (dd, J = 12.8, 5.7 Hz, 1H), 2.65 (t, J = 6.9 Hz, 1H), 0.96 (dd, J = 7.0, 3.7 Hz, 3H).

^{13}C-NMR (100 MHz, $CDCl_3$): δ 175.6, 160.33, 160.30, 159.96, 159.94, 146.90, 146.88, 138.6, 137.5, 133.8, 133.5, 129.8, 129.35, 129.17, 128.4, 127.7, 127.48, 127.44, 126.9, 125.93, 125.89, 125.86, 125.82, 122.1, 100.0, 60.2, 52.9, 52.0, 40.05, 39.99, 31.17, 31.12, 17.93, 17.89. LRMS 672.1, HRMS (ESI) m/z: $[M+H]^+$ ($C_{28}H_{26}ClF_3N_3O_4SSe$) 672.0450; Found 672.0463.

2,2-dimethoxyethyl (*Z*)-3-(4-chlorophenyl)-4-phenyl-*N*-((4-(trifluoromethyl)phenyl)sulfonyl)-4,5-dihydro-1*H*-pyrazole-1-carbimidoselenoate (20)

Using the general procedure, compound **1a** (100 mg, 0.2 mmol) was converted in a three-step, one-pot protocol using 2-bromo-1,1-dimethoxyethane (1.5 eq) as the alkylating agent to yield title compound **20** (62 mg, 48%).

^{1}H-NMR (400 MHz, $CDCl_3$): δ 8.09 (d, J = 7.9 Hz, 2H), 7.74 (d, J = 7.8 Hz, 2H), 7.58 (d, J = 7.6 Hz, 2H), 7.31 (dt, J = 14.9, 7.4 Hz, 6H), 7.16 (d, J = 7.1 Hz, 2H), 4.99 (t, J = 11.0 Hz, 1H), 4.86 (dd, J = 11.0, 4.3 Hz, 1H), 4.57-4.53 (m, 1H), 4.39 (t, J = 4.9 Hz, 1H), 3.16 (s, 6H), 3.02 (t, J = 5.0 Hz, 2H). ^{13}C-NMR (100 MHz, $CDCl_3$): δ 160.4, 138.6, 137.5, 129.8, 129.34, 129.24, 129.20, 128.5, 127.7, 127.4, 127.0, 125.91, 125.87, 125.84, 103.6, 60.1, 54.03, 53.93, 52.8, 31.0. LRMS 660.0, HRMS (ESI) m/z: $[M+H]^+$ ($C_{27}H_{26}ClF_3N_3O_4SSe$) 660.0450; Found 660.0456.

2-(Diethoxyphosphoryl)ethyl3-(4-chlorophenyl)-4-phenyl-*N*-((4-(trifluoromethyl)phenyl)-sulfonyl)-4,5-dihydro-1*H*-pyrazole-1-carbimidoselenoate (21)

Using the general procedure, compound **1a** (50 mg, 0.1 mmol) was converted in a three-step, one-pot protocol using diethyl (2-bromoethyl)phosphonate (1.5 eq) as the alkylating agent to yield title compound **21** (34 mg, 47%).

^{1}H-NMR (400 MHz, $CDCl_3$): δ 8.08 (d, *J* = 8.0 Hz, 2H), 7.75 (d, *J* = 8.0 Hz, 2H), 7.57 (d, *J* = 8.0 Hz, 2H), 7.31 (dt, *J* = 14.6, 7.2 Hz, 5H), 7.14 (d, *J* = 7.2 Hz, 2H), 4.98 (t, *J* = 11.5 Hz, 1H), 4.86 (dd, *J* = 11.0, 4.9 Hz, 1H), 4.55 (t, *J* = 5.6 Hz, 1H), 3.96 (quintet, *J* = 7.3 Hz, 4H), 3.03 (dd, *J* = 9.2, 6.3 Hz, 2H), 2.07 (dt, *J* = 17.6, 8.6 Hz, 2H), 1.21 (t, *J* = 7.0 Hz, 7H).

^{13}C-NMR (100 MHz, $CDCl_3$): δ 160.5, 138.5, 137.6, 129.8, 129.32, 129.21, 128.5, 127.6, 127.4, 127.0, 125.97, 125.94, 61.87, 61.80, 60.1, 52.8, 27.6, 26.2, 16.52, 16.46. LRMS 736.1.1, HRMS (ESI) m/z: $[M+H]^+$ ($C_{29}H_{31}ClF_3N_3O_5PSSe$) 736.0528; Found 736.0518.

.

2-Oxobutyl-3-(4-chlorophenyl)-4-phenyl-*N*-((4-(trifluoromethyl)phenyl)sulfonyl)-4,5-dihydro-1*H*-pyrazole-1-carbimidoselenoate (22)

Using the general procedure, compound **1a** (50 mg, 0.1 mmol) was converted in a three-step, one-pot protocol using 1-bromobutan-2-one (1.5 eq) as the alkylating agent to yield title compound **22** (38 mg, 60%).

^{1}H-NMR (400 MHz, $CDCl_3$): δ 8.07 (d, *J* = 8.1 Hz, 2H), 7.76 (d, *J* = 8.2 Hz, 2H), 7.57 (d, *J* = 8.5 Hz, 2H), 7.33 (q, *J* = 7.4 Hz, 3H), 7.27 (d, *J* = 8.4 Hz, 3H), 7.16 (d, *J* = 7.2 Hz, 2H), 5.00 (t, *J* = 11.6 Hz, 1H), 4.88 (dd, *J* = 11.2, 5.3 Hz, 1H), 4.55 (dd, *J* = 12.1, 5.0 Hz, 1H), 3.66 (s, 2H), 2.21 (q, *J* = 7.3 Hz, 2H), 0.89 (t, *J* = 7.3 Hz, 3H).

^{13}C-NMR (100 MHz, $CDCl_3$): δ 205.4, 160.6, 146.5, 138.4, 137.7, 129.8, 129.36, 129.24, 128.5, 127.53, 127.46, 127.1, 125.93, 125.89, 125.85, 60.2, 53.0, 37.2, 34.9, 25.5, 7.9. LRMS 642.0, HRMS (ESI) m/z: $[M+H]^+$ ($C_{27}H_{24}ClF_3N_3O_3SSe$) 642.0344; Found 642.0349.

***sec*-Butyl 3-(4-chlorophenyl)-4-phenyl-*N*-((4-(trifluoromethyl)phenyl)sulfonyl)-4,5-dihydro-1*H*-pyrazole-1-carbimidoselenoate (23)**

Using the general procedure, compound **1a** (100 mg, 0.2 mmol) was converted in a three-step, one-pot protocol using 2-bromobutane (1.5 eq) as the alkylating agent to yield title compound **23** (62 mg, 50%).

^{1}H-NMR (400 MHz, $CDCl_3$): δ 8.09 (d, *J* = 7.6 Hz, 2H), 7.73 (d, *J* = 7.8 Hz, 2H), 7.58 (d, *J* = 7.6 Hz, 2H), 7.33-7.28 (m, 5H), 7.15 (d, *J* = 5.6 Hz, 2H), 4.90-4.82 (m, 2H), 4.48-4.45 (m, 1H), 3.60 (d, *J* = 6.3 Hz, 1H), 1.66 (d, *J* = 5.6 Hz, 2H), 1.34 (s, 3H), 0.83 (dd, *J* = 13.6, 6.7 Hz, 3H). ^{13}C-NMR (100 MHz, $CDCl_3$): δ 160.40, 160.35, 160.33, 160.25, 160.22, 146.84, 146.78, 146.77, 146.75, 138.7, 137.3, 133.7, 133.3, 129.65, 129.59, 129.34, 129.33, 129.29, 129.23, 129.14, 128.8, 128.24, 128.15, 128.04, 127.96, 127.88, 127.82, 127.61, 127.49, 127.45, 127.42, 127.40, 127.38, 127.34, 127.27, 127.14, 126.90, 126.85, 125.84, 125.74, 125.71, 124.9, 122.2, 52.79, 52.76, 52.74, 51.6, 30.3, 20.8, 20.4, 12.19, 12.10. LRMS 628.1, HRMS (ESI) m/z: $[M+H]^+$ ($C_{27}H_{25}ClF_3N_3O_2SSe$) 628.0552; Found 628.0560.

3-Oxobutan-2-yl (*Z*)-3-(4-chlorophenyl)-4-phenyl-*N*-((4-(trifluoromethyl)phenyl)sulfonyl)-4,5-dihydro-1*H*-pyrazole-1-carbimidoselenoate (24)

Using the general procedure, compound **1a** (50 mg, 0.1 mmol) was converted in a three-step, one-pot protocol using 3-bromobutan-2-one (1.5 eq) as the alkylating agent to yield title compound **24** (31 mg, 49%).

^{1}H-NMR (400 MHz, $CDCl_3$): δ 8.08 (d, *J* = 8.0 Hz, 2H), 7.77 (d, *J* = 8.2 Hz, 2H), 7.56 (d, *J* = 8.2 Hz, 2H), 7.32 (t, *J* = 7.7 Hz, 3H), 7.29 (d, *J* = 9.6 Hz, 3H), 7.17 (t, *J* = 6.0 Hz, 2H), 5.07-5.02 (m, 1H), 4.91 (dd, *J* = 10.4, 5.4 Hz, 1H), 4.62-4.59 (m, 1H), 4.16 (t, *J* = 8.9 Hz, 1H), 2.07 (d, *J* = 10.5

Hz, 3H), 1.46 (t, *J* = 7.9 Hz, 3H). ^{13}C-NMR (100 MHz, $CDCl_3$): ^{13}C-NMR (100 MHz, $CDCl_3$): δ 205.64, 160.68, 146.301, 138.42, 138.37, 137.74, , 129.86, 129.86, 129.38, 129.26, 128.54, 127.46, 127.07, 127.04, 126.00, 125.96, 60.30, 53.06, 47.71, 27.47, 27.22, 16.16. LRMS 642.0, HRMS (ESI) m/z: $[M+H]^+$ ($C_{27}H_{24}ClF_3N_3O_3SSe$) 642.0340; Found 642.0344.

Ethyl 2-(((4-(4-chlorophenyl)-3-phenyl-2,3-dihydro-1*H*-pyrrol-1-yl)(((4-(trifluoromethyl)-phenyl)sulfonyl)imino)methyl)selanyl)butanoate (25)

Using the general procedure, compound **1a** (150 mg, 0.30 mmol) was converted in a three-step, one-pot protocol using methyl 2-bromopropanoate (1.5 eq) as the alkylating agent to yield title compound **25** as mixture of isomers (90 mg, 44%).

^{1}H NMR (400 MHz, $CDCl_3$): δ 8.10 (d, *J* = 8.0 Hz, 2H), 7.74 (d, *J* = 8.0 Hz, 2H), 7.56 (d, *J* = 8.1 Hz, 2H), 7.30 (d, *J* = 7.5 Hz, 2H), 7.25 (d, *J* = 6.6 Hz, 3H), 7.15 (d, *J* = 7.3 Hz, 2H), 5.00 (q, *J* = 11.7 Hz, 1H), 4.88 (dt, *J* = 11.8, 3.3 Hz, 1H), 4.61 – 4.50 (m, 1H), 4.14 – 3.95 (m, 3H), 1.93 (dq, *J* = 17.1, 8.3 Hz, 1H), 1.77 (ddt, *J* = 20.8, 14.6, 7.1 Hz, 1H), 1.26 – 1.10 (m, 4H), 0.78 (dt, *J* = 23.9, 7.5 Hz, 3H).

^{13}C-NMR (100 MHz, $CDCl_3$) δ 172.17, 160.56, 158.77, 146.40, 138.62, 137.59, 133.86, 133.54, 130.22, 129.66, 129.36, 128.77, 128.01, 127.63, 127.19, 125.84, 124.88, 122.16, 61.44, 47.38, 14.29, 14.16, 12.63. HRMS (ESI) m/z: $[M+H]^+$ ($C_{29}H_{27}ClF_3N_3O_4SSe$) 686.0606; Found, 686.0594.

Methyl 3-(((3-(4-chlorophenyl)-4-phenyl-4,5-dihydro-1*H*-pyrazol-1-yl)(((4-(trifluoromethyl)phenyl)sulfonyl)imino)methyl)selanyl)-2,2-difluoropropanoate (26)

Using the general procedure, compound **1a** (75 mg, 0.15 mmol) was converted in a three-step, one-pot protocol using ethyl 2-bromo-2,2-difluoroacetate 1.5 eq) as the alkylating agent to yield title compound **26** (36 mg, 35%).

^{1}H-NMR (400 MHz, $CDCl_3$): δ 8.04 (d, *J* = 8.1 Hz, 2H), 7.76 (d, *J* = 8.2 Hz, 2H), 7.55 (d, *J* = 8.2 Hz, 2H), 7.34 (t, *J* = 7.1 Hz, 2H), 7.29 (t, *J* = 7.7 Hz, 3H), 7.15 (d, *J* = 7.3 Hz, 2H), 5.00-4.89 (m, 2H), 4.53 (t, *J* = 5.8 Hz, 1H), 4.02 (q, *J* = 7.0 Hz, 2H), 1.15 (t, *J* = 7.1 Hz, 3H).

^{13}C-NMR (100 MHz, $CDCl_3$): δ 165.60, 161.01, 145.08, 138.28, 138.02, 129.95, 129.54, 129.34, 128.67, 127.62, 127.45, 127.19, 125.82, 63.82, 59.91, 53.32, 13.85. LRMS 694.0, HRMS (ESI) m/z: $[M+H]^+$ ($C_{27}H_{22}ClF_5N_3O_4SSe$) 694.0105; Found 694.0115.

4-((((3-(4-chlorophenyl)-4-phenyl-4,5-dihydro-1*H*-pyrazol-1-yl)(((4-(trifluoromethyl)phenyl)sulfonyl)imino)methyl)selanyl)methyl)phenyl)boronic acid (27)

Using the general procedure, compound **1a** (75 mg, 0.15 mmol) was converted in a three-step, one-pot protocol using (4-(bromomethyl)phenyl)boronic acid (1.5 eq) as the alkylating agent to yield title compound **27** (35 mg, 34%).

^{1}H-NMR (400 MHz, $CDCl_3$): δ 8.54 (d, *J* = 8.4 Hz, 1H), 8.21 (t, *J* = 9.0 Hz, 2H), 8.02 (d, *J* = 7.9 Hz, 3H), 7.69 (d, *J* = 8.0 Hz, 3H), 7.57-7.49 (m, 6H), 7.31 (dd, *J* = 14.0, 6.8 Hz, 27H), 7.15 (dd, *J* = 17.6, 7.3 Hz, 4H), 4.90-4.82 (m, 2H), 4.79-4.76 (m, 1H), 4.45-4.41 (m, 1H), 2.88 (s, 9H), 2.83 (dd, *J* = 7.8, 4.8 Hz, 3H), 1.73 (dd, *J* = 8.2, 4.3 Hz, 2H). ^{13}C-NMR (100 MHz, $CDCl_3$): δ 160.38, 146.48, 140.06, 138.62, 138.59, 137.53, 137.50, 136.07, 134.00, 133.48, 129.81, 129.23, 127.42, 127.26, 59.87, 52.42, 33.48. LRMS 720.0, HRMS (ESI) m/z: $[M+H]^+$ ($C_{31}H_{27}BClF_3N_3O_4SSe$) 720.0621; Found 720.0609.

Methyl (2-(-(3-(4-chlorophenyl)-4-phenyl-4,5-dihydro-1*H*-pyrazol-1-yl)(((4-(trifluoromethyl)phenyl)sulfonyl)imino)methyl)selanyl)acetyl)-*L*-valinate (28)

Using the general procedure, compound **1a** (50 mg, 0.1 mmol) was converted in a three-step, one-pot protocol using methyl (2-bromoacetyl)-L-valinate (1.5 eq) as the alkylating agent to yield title compound **28** (23 mg, 31%).

^{1}H-NMR (400 MHz, $CDCl_3$): δ 8.16-8.05 (m, 2H), 7.78 (t, *J* = 5.4 Hz, 2H), 7.59 (d, *J* = 8.1 Hz, 2H), 7.32 (dt, *J* = 15.7, 7.8 Hz, 6H), 7.16 (t, *J* = 8.4 Hz, 2H), 6.74 (dd, *J* = 23.7, 8.7 Hz, 1H), 5.08-4.96 (m, 1H), 4.93-4.87 (m, 1H), 4.64-4.57 (m, 1H), 4.43 (dt, *J* = 8.4, 4.3 Hz, 1H), 3.70 (d, *J* = 12.3 Hz, 3H), 3.58-3.44 (m, 2H), 2.10-2.05 (m, 1H), 0.87-0.80 (m, 7H). ^{13}C-NMR (100 MHz, $CDCl_3$): 172.08, 172.01, 169.44, 169.17, 161.41, 161.16, 146.22, 138.34, 138.30, 137.91, 129.88, 129.84, 129.48, 129.46, 129.29, 128.58, 128.53, 127.45, 127.35, 127.30, 127.16, 127.09, 127.07, 126.24, 126.20, 126.16, 126.13, 60.62, 60.36, 57.55, 57.43, 53.09, 52.23, 52.19, 31.34, 31.24, 30.63, 30.39, 19.05, 19.03, 17.93, 17.87. LRMS 743.1, HRMS (ESI) m/z: $[M+H]^+$ ($C_{31}H_{31}ClF_3N_4O_5SSe$) 743.0802; Found 743.0820.

2-(Fluorosulfonyl)ethyl-3-(4-chlorophenyl)-4-phenyl-*N*-((4-(trifluoromethyl)phenyl)sulfonyl)-4,5-dihydro-1*H*-pyrazole-1-carbimidoselenoate (29)

Using the general procedure, compound **1a** (50 mg, 0.1 mmol) was converted in a three-step, one-pot protocol using DMF and vinylulfonylfluoride (1.5 eq) as the alkylating agent to yield title compound **29** (22 mg, 33%).

^{1}H-NMR (400 MHz, $CDCl_3$): δ δ 8.09-8.04 (m, 2H), 7.80-7.76 (m, 2H), 7.58-7.56 (m, 2H), 7.37-7.32 (m, 5H), 7.17 (ddd, *J* = 3.8, 2.0, 1.0 Hz, 3H), 5.14-5.10 (m, 1H), 4.97 (dq, *J* = 4.5, 1.6 Hz, 1H), 4.72-4.69 (m, 1H), 3.60-3.54 (m, 2H), 3.06-3.03 (m, 2H).^{13}C-NMR (100 MHz, $CDCl_3$): δ 160.91, 157.48, 145.91, 138.23, 137.93, 134.77, 134.45, 134.12, 130.39, 130.32, 130.28, 130.22,

130.14, 130.07, 130.03, 130.00, 129.97, 129.90, 129.85, 129.78, 129.70, 129.64, 129.49, 129.39, 129.32, 129.27, 129.16, 129.07, 128.96, 128.88, 128.81, 128.26, 128.21, 128.19, 128.17, 127.94, 127.90, 127.88, 127.50, 127.45, 127.42, 127.39, 127.36, 127.29, 127.22, 126.99, 126.93, 126.87, 126.84, 126.80, 126.68, 126.39, 124.73, 122.02, 53.89, 53.23, 50.86, 19.09. LRMS 682.0, HRMS (ESI) m/z: $[M+H]^+$ ($C_{25}H_{21}ClF_4N_3O_4S_2Se$) 681.9763; Found 681.9774.

(2,5,6-trimethyl-1,7-dioxo-1*H*,7*H*-pyrazolo[1,2-*a*]pyrazol-3-yl)methyl (*Z*)-3-(4-chlorophenyl)-4-phenyl-*N*-((4-(trifluoromethyl)phenyl)sulfonyl)-4,5-dihydro-1*H*-pyrazole-1-carbimidoselenoate (30)

Using the general procedure, compound **1a** (50 mg, 0.1 mmol) was converted in a three-step, one-pot protocol using dioxane and bimane monobromide (1.5 eq) as the alkylating agent to yield title compound **30** (20 mg, 27%).

^{1}H-NMR (400 MHz, $CDCl_3$): δ 8.07 (d, *J* = 8.1 Hz, 2H), 7.78 (d, *J* = 8.0 Hz, 2H), 7.55 (d, *J* = 8.3 Hz, 2H), 7.32 (dd, *J* = 17.1, 11.5 Hz, 5H), 7.15 (d, *J* = 7.1 Hz, 2H), 4.99-4.93 (m, 2H), 4.55-4.51 (m, 1H), 4.07 (s, 2H), 2.30 (s, 4H), 1.80 (d, *J* = 11.2 Hz, 7H). ^{13}C-NMR (100 MHz, $CDCl_3$): 161.18, 160.73, 160.17, 157.35, 145.90, 144.45, 138.17, 129.94, 129.40, 129.35, 127.39, 126.90, 115.03, 113.43, 60.03, 53.12, 20.74, 11.67, 7.13, 6.98. LRMS 762.0, HRMS (ESI) m/z: $[M+H]^+$ ($C_{33}H_{28}ClF_3N_5O_4SSe$) 762.0668; Found 762.0667.

3-((5-(dimethylamino)naphthalene)-1-sulfonamido)propyl (*Z*)-3-(4-chlorophenyl)-4-phenyl-*N*-((4-(trifluoromethyl)phenyl)sulfonyl)-4,5-dihydro-1*H*-pyrazole-1-carbimidoselenoate (31)

Using the general procedure, compound **1a** (50 mg, 0.1 mmol) was converted in a three-step, one-pot protocol using N-(3-bromopropyl)-5-(dimethylamino)naphthalene-1-sulfonamide (1.5 eq) as the alkylating agent to yield title compound **31** (21 mg, 25%).

^{1}H-NMR (400 MHz, $CDCl_3$): δ 8.54 (d, *J* = 8.6 Hz, 1H), 8.21 (t, *J* = 9.6 Hz, 2H), 8.01 (d, *J* = 7.8 Hz, 3H), 7.74 (dd, *J* = 37.1, 7.9 Hz, 3H), 7.54 (dd, *J* = 16.7, 7.6 Hz, 5H), 7.30 (dt, *J* = 14.4, 7.2 Hz, 8H), 7.15 (dd, *J* = 17.0, 7.3 Hz, 4H), 4.87 (dd, *J* = 29.8, 13.1 Hz, 4H), 4.44-4.41 (m, 1H), 2.87 (s, 8H), 2.83 (d, *J* = 6.3 Hz, 3H), 1.73 (t, *J* = 6.2 Hz, 2H).

^{13}C-NMR (100 MHz, $CDCl_3$): δ 160.66, 159.13, 152.20, 146.69, 138.63, 137.54, 134.51, 130.68, 130.02, 129.85, 129.62, 129.34, 129.26, 128.67, 128.47, 127.80, 127.48, 127.21, 126.95, 126.46, 125.97, 125.93, 125.90, 123.34, 122.15, 118.66, 115.38, 59.90, 52.54, 45.56, 42.64, 29.94, 26.35.

LRMS 862.1, HRMS (ESI) m/z: $[M+H]^+$ ($C_{38}H_{36}ClF_3N_5O_4S_2Se$) Calc. 862.1015; Found 862.1007.

2-amino-2-oxoethyl (*Z*)-3-(4-chlorophenyl)-*N*-((4-chlorophenyl)sulfonyl)-4-phenyl-4,5-dihydro-1*H*-pyrazole-1-carbimidoselenoate (32)

Using the general procedure, compound **1f** (100 mg, 0.2 mmol) was converted in a three-step, one-pot protocol using bromoacetamide (1.5 eq) as the alkylating agent to yield title compound **32** (68 mg, 54%).

^{1}H-NMR (400 MHz, $CDCl_3$): δ 7.88 (d, *J* = 8.4 Hz, 2H), 7.58 (d, *J* = 8.5 Hz, 2H), 7.48 (d, *J* = 8.5 Hz, 2H), 7.35 (t, *J* = 7.4 Hz, 2H), 7.29 (dd, *J* = 12.6, 8.0 Hz, 4H), 7.16 (d, *J* = 7.2 Hz, 3H), 6.23 (s, 1H), 5.25 (s, 1H), 5.00 (dd, *J* = 15.3, 7.9 Hz, 1H), 4.90 (dd, *J* = 10.9, 5.3 Hz, 1H), 4.57-4.55 (m, 1H), 3.48 (s, 2H). ^{13}C-NMR (100 MHz, $CDCl_3$): δ 171.7, 161.0, 158.4, 141.4, 139.0, 138.4, 137.9, 129.9, 129.45, 129.33, 128.6, 127.9, 127.5, 60.3, 53.2, 30.2. LRMS 595.0.0, HRMS (ESI) m/z: $[M+H]^+$ ($C_{24}H_{21}Cl_2N_4O_3SSe$) 594.9877; Found 594.9871.

2-Amino-2-oxoethyl (*E*)-3-(4-chlorophenyl)-4-phenyl-*N*-((4-(trifluoromethyl)piperidin-1-yl)sulfonyl)-4,5-dihydro-1*H*-pyrazole-1-carbimidoselenoate (33)

Using the general procedure, compound **1u** (100 mg, 0.19 mmol) was converted in a three-step, one-pot protocol using bromoacetamide (1.5 eq) as the alkylating agent to yield title compound **33** as a pale solid (45 mg, 36%).

^{1}H-NMR (400 MHz, $CDCl_3$): δ 7.56 (d, *J* = 8.4 Hz, 2H), 7.33 (d, *J* = 7.5 Hz, 3H), 7.28 (t, *J* = 8.7 Hz, 4H), 7.15 (d, *J* = 7.2 Hz, 2H), 6.71 (s, 1H), 5.57 (s, 1H), 4.94 (d, *J* = 11.8 Hz, 1H), 4.87 (dd, *J* = 11.2, 5.5 Hz, 1H), 4.49 (dd, *J* = 12.0, 5.3 Hz, 1H), 3.81 (s, 2H), 3.66 (d, *J* = 4.4 Hz, 2H), 2.78 (t, *J* = 12.0 Hz, 2H), 2.20 (s, 1H), 1.97 (d, *J* = 12.6 Hz, 2H), 1.71-1.69 (m, 3H). ^{13}C-NMR (100 MHz, $CDCl_3$): δ 171.35, 159.58, 157.10, 138.59, 137.60, 130.38, 129.85, 129.29, 128.53, 127.95, 127.61, 127.51, 127.34, 126.57, 59.63, 53.26, 45.86, 39.78, 39.67, 39.53, 30.09, 24.17, 23.78. LRMS 636.1.

2-Amino-2-oxoethyl (*Z*)-3-ethyl-*N*-((4-isopropylphenyl)sulfonyl)-4-phenyl-4,5-dihydro-1*H*-pyrazole-1-carbimidoselenoate (34)

Using the general procedure, compound **6c** (100 mg, 0.25 mmol) was converted in a three-step, one-pot protocol using cyclopropyl methylbromide (1.5 eq) as the alkylating agent to yield title compound **34** as pale-yellow viscous oil (60 mg, 46 % yield).

^{1}H-NMR (400 MHz, $CDCl_3$): δ 7.87 (d, *J* = 8.1 Hz, 2H), 7.41–7.29 (m, 5H), 7.14 (d, *J* = 7.3 Hz, 2H), 6.16 (s, 1H), 5.08–4.92 (m, 2H), 4.56–4.46 (m, 1H), 4.44–4.36 (m, 1H), 3.42–3.32 (m, 2H), 3.04–2.91 (m, 1H), 2.36–2.11 (m, 2H), 1.26 (d, *J* = 6.9 Hz, 6H), 1.12 (t, *J* = 7.5 Hz, 3H).

^{13}C-NMR (100 MHz, $CDCl_3$): δ 172.4, 168.8, 157.9, 153.8, 140.3, 137.4, 129.5, 128.3, 127.6, 127.0, 126.3, 58.8, 55.7, 34.1, 29.5, 23.7, 22.0, 10.1. LRMS: 521.1, HRMS (ESI) m/z: $[M+H]^+$ ($C_{23}H_{29}N_4O_3SSe$) 521.1126; Found 521.1124.

2-(Methoxymethoxy)ethyl (*Z*)-3-ethyl-4-phenyl-*N*-((4-(trifluoromethoxy)phenyl)sulfonyl)-4,5-dihydro-1*H*-pyrazole-1-carbimidoselenoate (35)

Using the general procedure, compound **6a** (100 mg, 0.22 mmol) was converted in a three-step, one-pot protocol using 2-(Methoxymethoxy)ethyl bromide (1.5 eq) as the alkylating agent to yield title compound **35** as pale viscous oil (25 mg, 19 % yield).

^{1}H NMR (400 MHz, $CDCl_3$): δ 8.01 (d, *J* = 7.9 Hz, 2H), 7.40–7.27 (m, 5H), 7.14 (d, *J* = 7.2 Hz, 2H), 4.91 (t, *J* = 11.8 Hz, 1H), 4.52–4.42 (m, 3H), 4.40–4.33 (m, 1H), 3.65 (t, *J* = 6.8 Hz, 2H), 3.29 (s, 3H), 3.01 (t, *J* = 6.9 Hz, 2H), 2.35–2.12 (m, 2H), 1.12 (t, *J* = 7.4 Hz, 3H). ^{13}C NMR (100 MHz, $CDCl_3$): δ 168.4, 158.8, 151.3, 142.0, 137.6, 129.5, 128.3(4), 128.2(6), 127.6, 120.5, 96.3, 66.8, 58.6, 55.3, 55.2, 28.2, 22.1, 10.2. LRMS: 594.1, HRMS (ESI) m/z: $[M+H]^+$ ($C_{23}H_{27}F_3N_3O_5SSe$) 594.0789; Found 594.0785.

3-Cyanopropyl (E)-3-(4-chlorophenyl)-N-(naphthalen-2-ylsulfonyl)-4-phenyl-5,6-dihydropyridazine-1(4H)-carbimidoselenoate (36)

Using the general procedure, compound **8b** (100 mg, 0.2 mmol) was converted in a three-step, one-pot protocol using 4-bromobutryonitrile (1.5 eq) as the alkylating agent to yield title compound 34 as yellow viscous oil (98 mg, 77.9% Yield). ^{1}H NMR (400 MHz, $CDCl_3$) δ 8.50 (s, 1H), 8.08 – 7.85 (m, 5H), 7.69 – 7.58 (m, 4H), 7.35 (t, *J* = 7.3 Hz, 2H), 7.29 (d, *J* = 6.6 Hz, 3H), 7.14 (d, *J* = 7.6 Hz, 2H), 4.76 (d, *J* = 12.6 Hz, 1H), 4.29 (s, 1H), 3.76 – 3.63 (m, 1H), 3.02 – 2.97 (m, 2H), 2.74 – 2.67 (m, 2H), 2.31 – 2.18 (m, 2H), 2.13 (td, *J* = 6.9, 1.6 Hz, 2H). ^{13}C NMR (101 MHz, $CDCl_3$) δ 164.70, 151.41, 139.55, 136.52, 134.51, 133.79, 132.13, 129.48, 129.36, 129.19, 129.06, 128.70, 127.97, 127.83, 127.64, 126.55, 122.30, 42.69, 37.81, 27.05, 22.30, 17.27, 16.99. LRMS 635.1, HRMS (ESI) m/z: [M+H]+ ($C_{31}H_{28}ClN_4O_2SSe$) 635.0787; Found 635.0789.

Prop-2-yn-1-yl (E)-3-(4-chlorophenyl)-4-phenyl-N-(phenylsulfonyl)-5,6-dihydropyridazine-1(4H)-carbimidoselenoate (37)

Using the general procedure, compound **8c** (100 mg, 0.2 mmol) was converted in a three-step, one-pot protocol using bromopropyne (1.5 eq) as the alkylating agent to yield title compound 35 as pale solid (43 mg, 35 % yield). ^{1}H NMR (400 MHz, $CDCl_3$) δ 7.99 (d, *J* = 6.3 Hz, 2H), 7.65 (d, *J* = 6.8 Hz, 2H), 7.56 – 7.47 (m, 3H), 7.38 – 7.28 (m, 4H), 7.26 (s, 1H), 7.13 (d, *J* = 7.3 Hz, 2H), 4.75 (d, *J* = 13.3 Hz, 1H), 4.27 (d, *J* = 5.7 Hz, 1H), 3.63 (td, *J* = 13.3, 4.2 Hz, 1H), 3.46 (s, 2H), 2.34 – 2.17 (m, 2H), 2.16 (s, 1H). ^{13}C NMR (101 MHz, $CDCl_3$) δ 164.49, 143.50, 139.45, 136.51, 133.64, 132.02, 128.73, 126.29, 80.02, 71.65, 42.42, 37.79, 26.41, 14.18. LRMS 556, HRMS (ESI) m/z: $[M+H]^+$ ($C_{26}H_{23}ClN_3O_2SSe$) 556.0365; Found 556.0370.

Methyl (E)-3-(((3-(4-chlorophenyl)-4-phenyl-5,6-dihydropyridazin-1(4H)-yl)(((2-(trifluoromethyl)phenyl)sulfonyl)imino)methyl)selanyl)-2-methylpropanoate (38)

Using the general procedure, compound **8d** (100 mg, 0.2 mmol) was converted in a three-step, one-pot protocol using *(R)*-3-bromo-2-methylpropanoate (1.5 eq) as the alkylating agent to yield title compound 36 as pale viscous oil (85 mg, 64 % Yield). ^{1}H NMR (400 MHz, $CDCl_3$) δ 8.33 (d, *J* = 7.8 Hz, 2H), 7.86 (d, *J* = 7.6 Hz, 2H), 7.69 (dd, *J* = 22.7, 8.0 Hz, 8H), 7.37 – 7.27 (m, 9H), 7.13 (d, *J* = 7.2 Hz, 4H), 4.70 (d, *J* = 12.9 Hz, 2H), 4.25 (d, *J* = 5.4 Hz, 2H), 3.63 (d, *J* = 4.5 Hz, 8H), 2.91 (dd, *J* = 13.1, 8.4 Hz, 2H), 2.81 (dd, *J* = 12.9, 5.7 Hz, 1H), 2.77 – 2.64 (m, 2H), 2.63 – 2.49 (m, 1H), 2.21 (dd, *J* = 44.0, 13.5 Hz, 4H), 0.90 (d, *J* = 6.9 Hz, 3H), 0.86 (d, *J* = 7.2 Hz, 3H). ^{13}C NMR (101 MHz, $CDCl_3$) δ 175.92, 139.63, 136.48, 133.78, 132.38, 132.00, 130.08, 129.48, 129.01, 128.12, 128.00, 127.79, 51.98, 42.60, 40.06, 39.98, 37.83, 30.91, 26.53, 18.34, 18.11. LRMS 686.1, HRMS (ESI) m/z: $[M+H]^+$ ($C_{29}H_{28}ClF_3N_3O_4SSe$) 686.0606; Found 686.0596.

Methyl *N*-((3*s*,5*s*,7*s*)-adamantan-1-yl)-*N'*-((4-chlorophenyl)sulfonyl)carbamimidoselenoate (39)

Using the general procedure A, *N*-(((3*s*,5*s*,7*s*)-adamantan-1-yl)carbamoyl)-4-chlorobenzenesulfonamide[9] (50 mg, 0.13 mmol) was converted in a three-step, one-pot protocol

using methyl iodide (1.5 eq) as the alkylating agent to yield title compound **39**. LCMS fingerprint MH^+ 447.0.

Experimental procedure for combinatorial method. A stock solution of urea precursors (0.01M) in toluene (**1b**, **1f**, **1i**, **1k**,**6d, 7e**) was converted to their respective imidoylchlorides. Following the general experimental procedure **A**, the combinatorial library reactions were carried out using 8 distinct alkylating agents **Y** (a total of 8 alkylating agents (1.5 eq based on SU precursors in degassed DMF/85 °C) were prepared. Upon the completion of these combinatorial library reactions, the reaction mixture was then analyzed by LC-MS. The yield was determined by LC-MS using the presence of precursor urea or imidoyl chloride. Crude yields were estimated based on LC signal integration with MS fingerprint and comparison with the precursor compounds evaluated in the substrate scope study.

Chiral HPLC traces for compound **4a, (-)-4a) and (+)-4a)**

Chiral HPLC traces for **4a, (-)-4a) and (+)-4a)**. Analytical HPLC conditions, 20 μl injection, EtOH (1.5mL/min), R-WhelkO, (5μ, 250 x 10 mm)

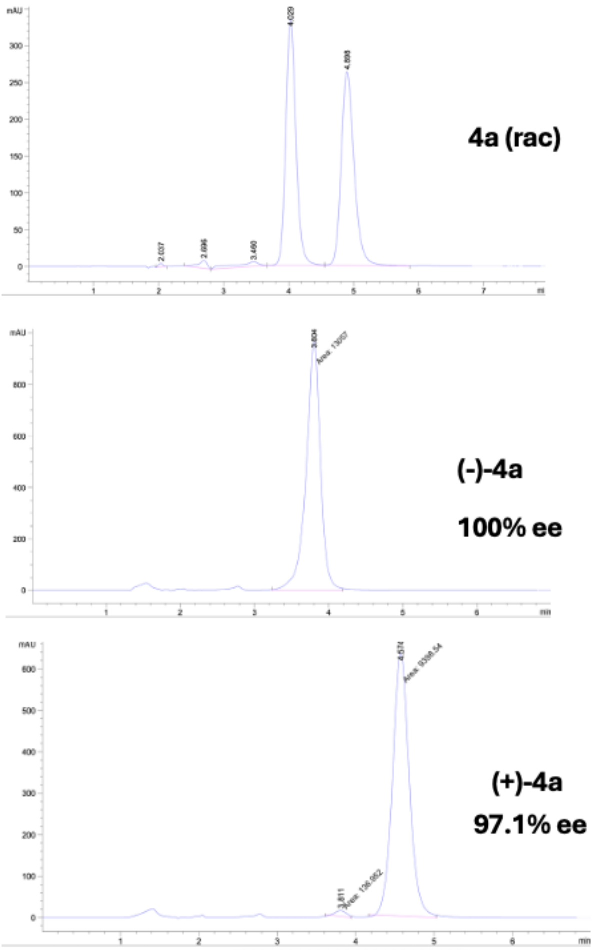

Chiral HPLC traces for compound **4f**, **4g** and **4h**

Analytical HPLC conditions, 20 µl injection, EtOH (1.5mL/min), R-WhelkO, (5µ, 250 x 10 mm)

4f

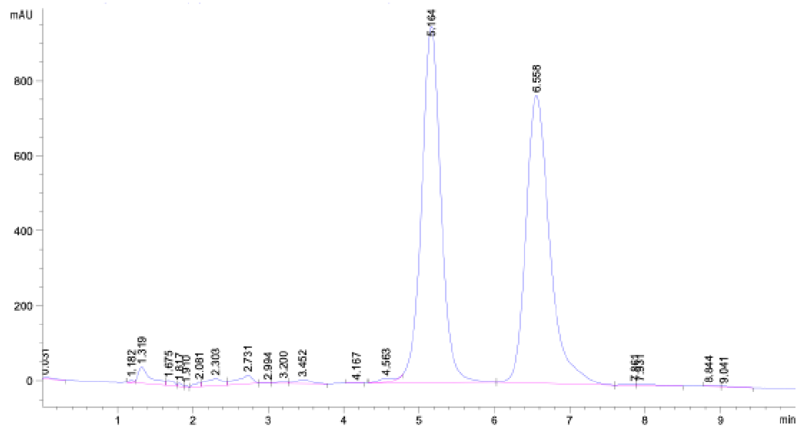

4g

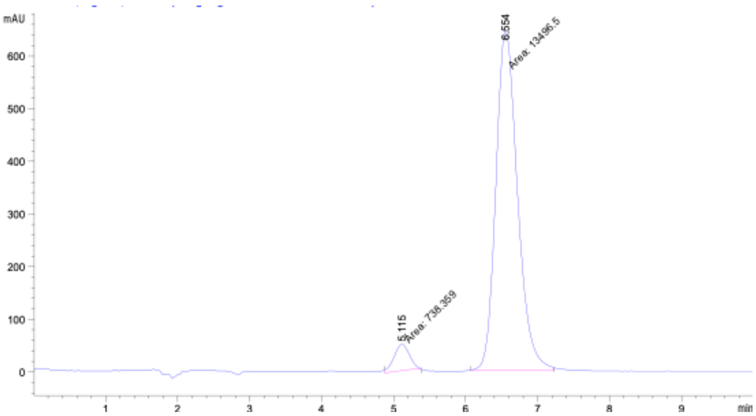

4h

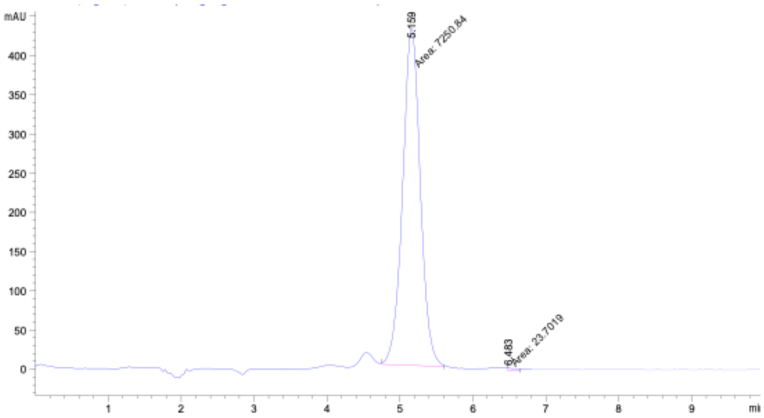

Chiral HPLC traces for compound **12, (-)-(12), (+)-(12)**

Analytical HPLC conditions, 20 μl injection, EtOH (1.5mL/min), R-WhelkO, (5μ, 250 x 10 mm)

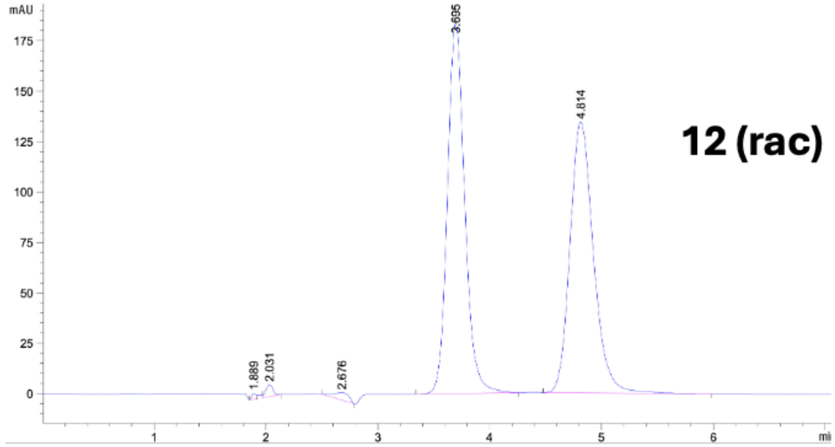

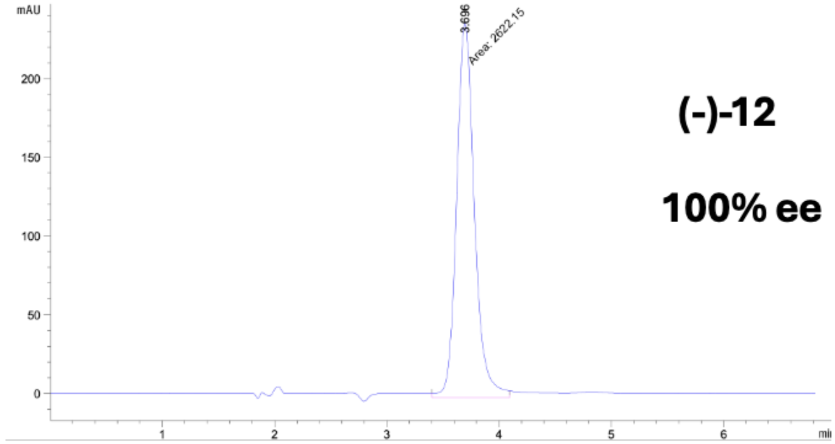

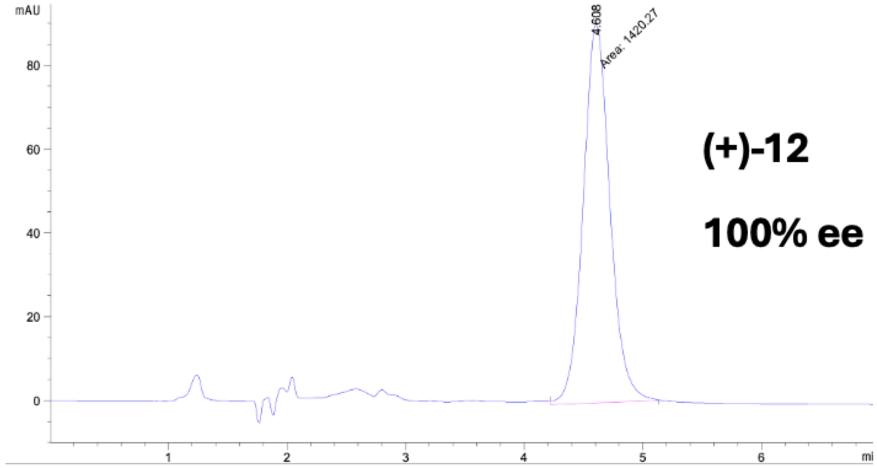

Chiral HPLC traces for compound **19**

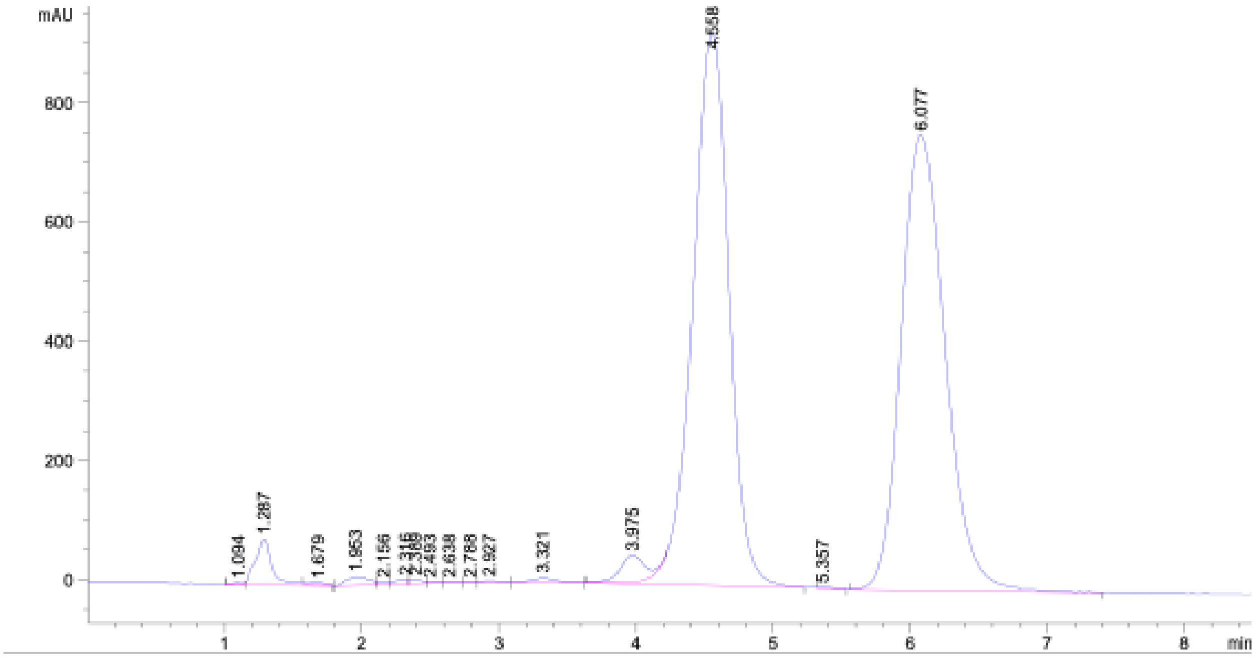

Analytical HPLC conditions, 20 μl injection, EtOH (1.5mL/min), R-WhelkO, (5μ, 250 x 10 mm)

X-Ray structure of Compound **4m**

Datablock 23068_a - ellipsoid plot

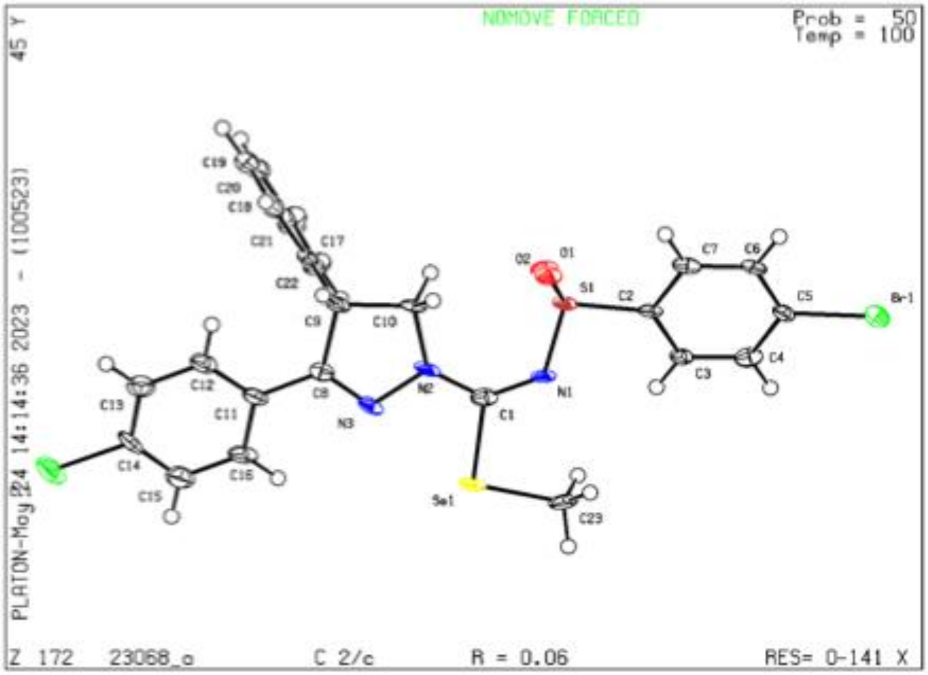

checkCIF/PLATON report

Structure factors have been supplied for datablock(s) 23068_a

THIS REPORT IS FOR GUIDANCE ONLY. IF USED AS PART OF A REVIEW PROCEDURE FOR PUBLICATION, IT SHOULD NOT REPLACE THE EXPERTISE OF AN EXPERIENCED CRYSTALLOGRAPHIC REFEREE.

No syntax errors found. CIF dictionary Interpreting this report

Datablock: 23068_a

```
Bond precision:   C-C = 0.0091 A          Wavelength=0.71073

Cell:              a=11.550(3)      b=17.326(5)         c=23.190(6)
                   alpha=90         beta=94.991(4)      gamma=90
Temperature:       100 K

                 Calculated                   Reported
Volume           4623(2)                      4623(2)
Space group      C 2/c                        C 2/c
Hall group       -C 2yc                       -C 2yc
Moiety formula   C23 H19 Br Cl N3 O2 S Se     C23 H19 Br Cl N3 O2 S Se
Sum formula      C23 H19 Br Cl N3 O2 S Se     C23 H19 Br Cl N3 O2 S Se
Mr               595.78                       595.79
Dx,g cm-3        1.712                        1.712
Z                8                            8
Mu (mm-1)        3.586                        3.585
F000             2368.0                       2368.0
F000'            2367.95
h,k,lmax         13,20,27                     13,20,27
Nref             4227                         4217
Tmin,Tmax        0.753,0.904                  0.517,0.924
Tmin'            0.391

Correction method= # Reported T Limits: Tmin=0.517 Tmax=0.924
AbsCorr = MULTI-SCAN

Data completeness= 0.998           Theta(max)= 25.337

R(reflections)= 0.0591( 3091)                          wR2(reflections)=
                                                       0.1252( 4217)
S = 1.078                  Npar= 290
```

GRAB$_{eCB2.0}$ Sensor Assay Protocol[10]

Briefly, the assay was conducted as previously reported. The GRAB$_{eCB2.0}$ generated in HEK cells (for reference: Plasmid #164604) was used. concentration of cells as adjusted to 0.7–1.0 million cells/mL with DMEM complete media and 100 µL was added to the desired wells of a clear bottom black plastic tissue culture treated 96-well plate. The plate was incubated at 37 °C and 8% CO_2 for a 24–48 h period. After incubation, the 100 µL DMEM buffer was removed and replaced with 100 µL of DPBS buffer containing calcium and magnesium. After the addition of the DPBS buffer, a baseline reading of the cells was taken using the PHERAstar FSX microplate reader (BMG/Labtech, Cary, NC, USA) to establish the F_0 or initial fluorescence. For presumed antagonists, the compounds can be assessed for fluorescence alterations in the presence and absence of an agonist. When running the assay for an antagonist in the absence of agonist, 25 µL of the compound and 25 µL of additional DPBS buffer was added to the wells. A concentration curve was made for the modulator with the final concentrations in the wells holding the cells ranging from 1 pM to 10 µM with 1% DMSO and 0.5 mg/mL BSA in a total volume of 150 µL. When running the assay in the presence of agonist, a total of 25 µL of the compound and 25 µL of additional DPBS buffer or 25 µL of a standardized agonist (CP55940, EC_{80} concentration) were used to assess fluorescence alterations. A concentration curve was made for the modulator with the final concentration in the wells holding the cells ranging from 1 pM to 10 µM, and the final concentration of the EC_{80} CP55940 in the well along with 1% DMSO and 0.5 mg/mL BSA in a total volume of 150 µL. In either assay, the cannabinoid ligands were added, followed by incubation of the compound on the cells at 37 °C for 5 min before taking a fluorescence reading using the PHERAstar FSX microplate reader equipped with a GFP filter cube module (Ex. 485 nm/Em. 520 nm). The recorded values were the F or final fluorescence. Data was plotted and

curves generated using and pIC_{50} calculated from independent experiments using GraphPad Prism 10. Datapoints represent the mean ± SD for each condition in experiments performed in triplicate.

**Compound name**	**CB_1R $pIC_{50}(CB_1)^a$**
**4a**	7.05
**(-)-4a**	7.46
**(+)-4a**	5.73
**4f**	6.47
**4h**	7.31
**4g**	6.36
**4m**	6.87
**4q**	6.82
**4s**	6.85
**4t**	6.55
**4u**	7.47
**4v**	6.72
**7a**	6.72
**9a**	7.09
**9b**	5.63
**9c**	6.06
**12-rac**	6.83
**(-)-12**	nt
**(+)-12**	nt
**20**	7.02
**21**	6.37
**26**	5.40
**27**	5.72
**31**	5.31
**32**	6.72
**34**	6.02
**36**	5.85
**37**	6.53
**38**	5.68

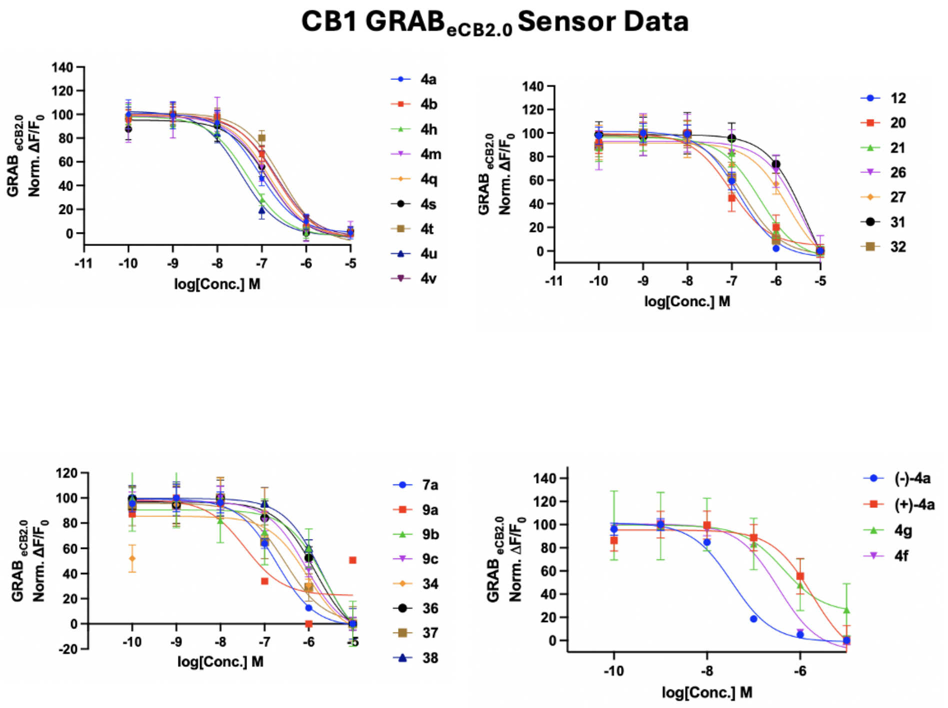

Figure S1. [a]Apparent functional CB_1R antagonism was determined using $GRAB_{eCB2.0}$ sensor in HEK293K cells in the presence of CB1 agonist CP55,940 (at EC_{80} concentration) for select compounds.[10] pIC_{50} values were calculated and plotted using GraphPad Prism 10. Data represent mean from three independent experiments. Nt: not tested.

MD simulation and enthalpy calculation methods

Dynamics simulations followed protocols established in previous studies.[11,12] Briefly, five independent simulations were conducted for each complex (CB_1R bound to either compound **4h** or ibipinabant), with data collected into single ensembles for analysis. Each complex was embedded in a lipid bilayer, solvated, and simulated at 37 °C, 1 atm, and ~120 mM Na^+/K^+, with ionizable side chains protonated for neutral pH. Systems were neutralized with an appropriate number of counterions. All simulations were performed using NAMD and the CHARMM force field (version c4621). Ligand parameters were derived by analogy to related compounds, with APT charges computed from geometry optimizations at the DFT level (B3LYP/6-31G**) in the gas phase using Gaussian (version 16). Each simulation was extended to 100 ns following thermal equilibration, with data analyzed over the final 50 ns, during which structural convergence was observed. Conformational clustering and statistical analysis of ligand–receptor and ligand–water interactions were performed as previously described[11,12] and summarized below. A folder containing the configuration files for the NAMD simulations, the initial coordinates of all system components, and the relevant CHARMM topology and parameter files has been uploaded as Supplementary Data. Bond dissociation enthalpies (X–R, where X = O, S, Se, Te, or Cl; R = shared 3-arm scaffold; Fig. 5 and SI) were calculated at the DFT level with the B3LYP and DGDZVP basis set as the enthalpy difference between bonded and dissociated states.

Metrics include polar and nonpolar interaction networks, as well as receptor conformers and occupancies, described in a previous study[12] and reproduced below for completeness.

Conformational clustering identifies the most populated conformations sampled by the receptor during a simulation. Regions of configurational space, defined here by the C_α positions, are sampled with varying frequency, and local density peaks correspond to clusters whose representative structures are termed conformers. Occupancies (the percentage of conformations in each cluster) are obtained using a density-based algorithm, as follows: The conformation nearest the point of highest local density, computed as the number of neighbors within a fixed radius δ (here, δ = 1.5 Å) is designated as conformer c_1. Similarity

between conformations is evaluated by C_α-RMSD after optimal superposition (first and last four residues excluded). All conformations within δ of c_1 are grouped into its family and removed from further consideration; c_1 is the main conformer with occupancy (or population) p_1. The procedure is then iterated to identify subsequent peaks (c_2, c_3, …) until all conformations are assigned to one of M families. The receptor's dynamics are thus represented by conformer-occupancy pairs $\{(c_l, p_l)\}$, with $\sum p_l = 1$.

Polar and nonpolar interactions can be computed between the receptor and ligand, receptor and water, and ligand and water. Among polar contacts, hydrogen bonds and ionic interactions dominate, with dipole–ion, dipole–dipole, and cation–π interactions make relevant contributions. The calculation does not distinguish between these, as all polar contacts are treated under a single distance-based definition: heavy atoms (excluding carbon) bonded to hydrogen are generically designated donors (D), and heavy atoms capable of polar interaction with donors are acceptors (A). A group K (e.g., a residue in the receptor or an atom in the ligand) is considered to interact with group L at time t if the acceptor-donor distance (δ_{AD}) for at least one $A - D$ pair between the groups satisfies $\delta_{AD} < 3$ Å. The total number of interactions between K and L over the entire trajectory, $f_{K,L}$, is the sum of all such interactions normalized to the maximum value of f within the system; for comparative analysis between ligands, the maximum of f across all systems is used. This metric quantifies the relevance of each group in polar stabilization, accounting for both the frequency of the interactions through time and the number of participating atoms at each time. Thus, to compare the effects of ligands l_1 and l_2 on the interaction between K and L, the ratio $f_{K,L}(l_1)/f_{K,L}(l_2)$ serves as a proxy for the relative strength of such interaction.

Nonpolar interactions are computed analogously. Dispersion, π–π, CH–π, aromatic–aliphatic, and hydrophobic contacts are grouped under a carbon–carbon distance criterion of $\delta_{CC} < 4.8$ Å. The normalized metric $g_{K,L}$ quantifies the strength of these interactions, whether buried or solvent-exposed, and includes contributions from carbon-containing moieties of polar or charged residues.

Relative values of $f_{K,L}$ and $g_{K,L}$ for the most relevant interactions with ibipinabant and the Se-containing compound are indicated schematically in Fig. 6 by the thickness of the connecting dashed lines.

Figure S5 reports the corresponding numerical values and expands $g_{K,L}$ to a per-residue representation for Arm 4, normalized between ligands. All output data from which these quantities are derived are provided in the simulation package. The total interaction strength of a given residue K is defined as the sum of $f_{K,L}$ or $g_{K,L}$ over all interacting groups L. The values normalized within each ligand are reported in the B-factor column of the PDB coordinate files, ChimeraX sessions, and animation V1. These values are represented as heat maps colored from blue (low strength) to yellow (maximum strength).

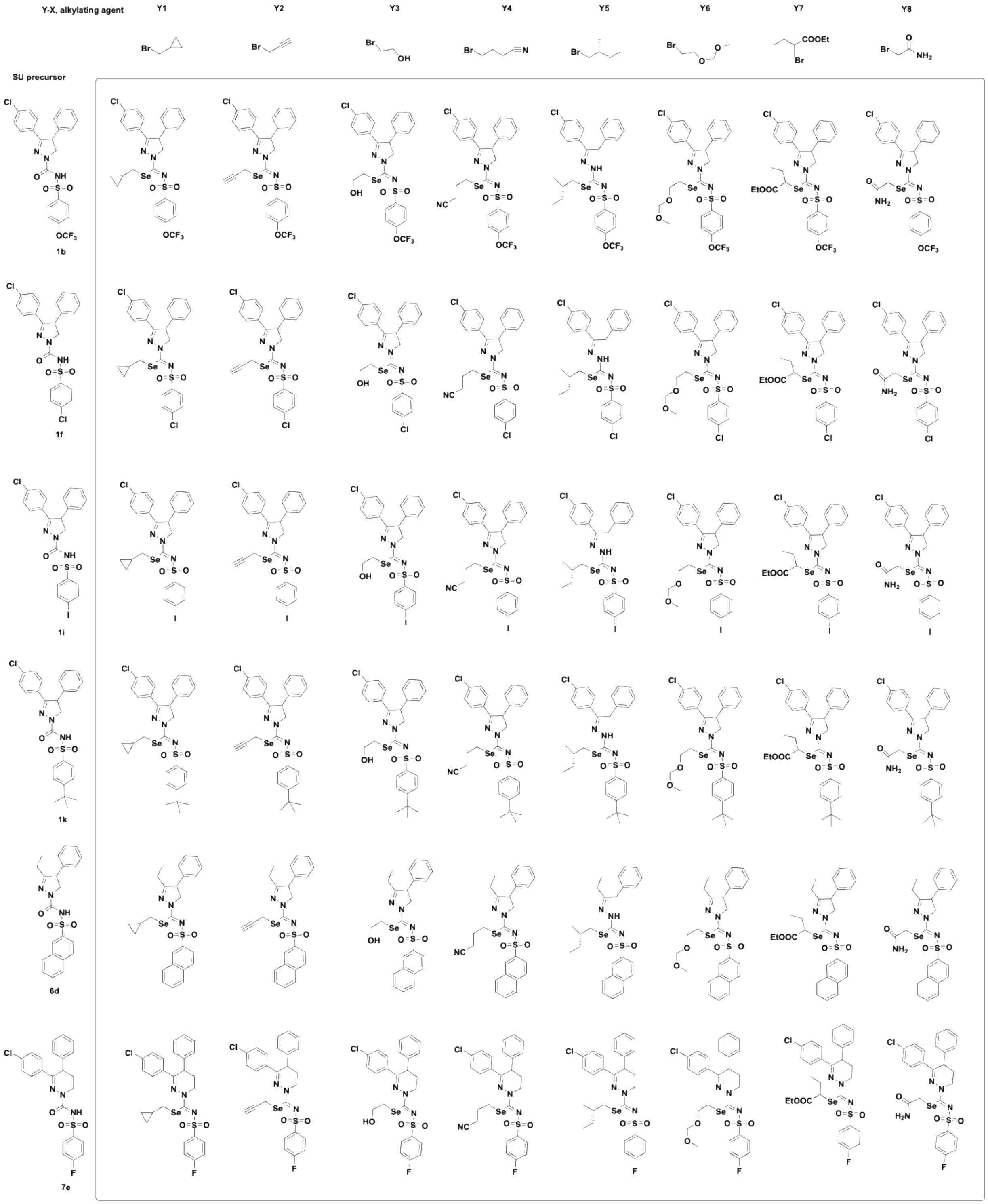

Figure S2. Structures of all the 48 combinatorial reaction products along with their precursors and alkylating agents

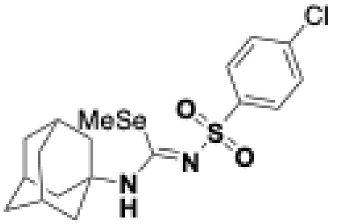

Compond **39**

Chemical Formula: $C_{18}H_{23}ClN_2O_2SSe$

Exact Mass: 446.03

Molecular Weight: 445.88

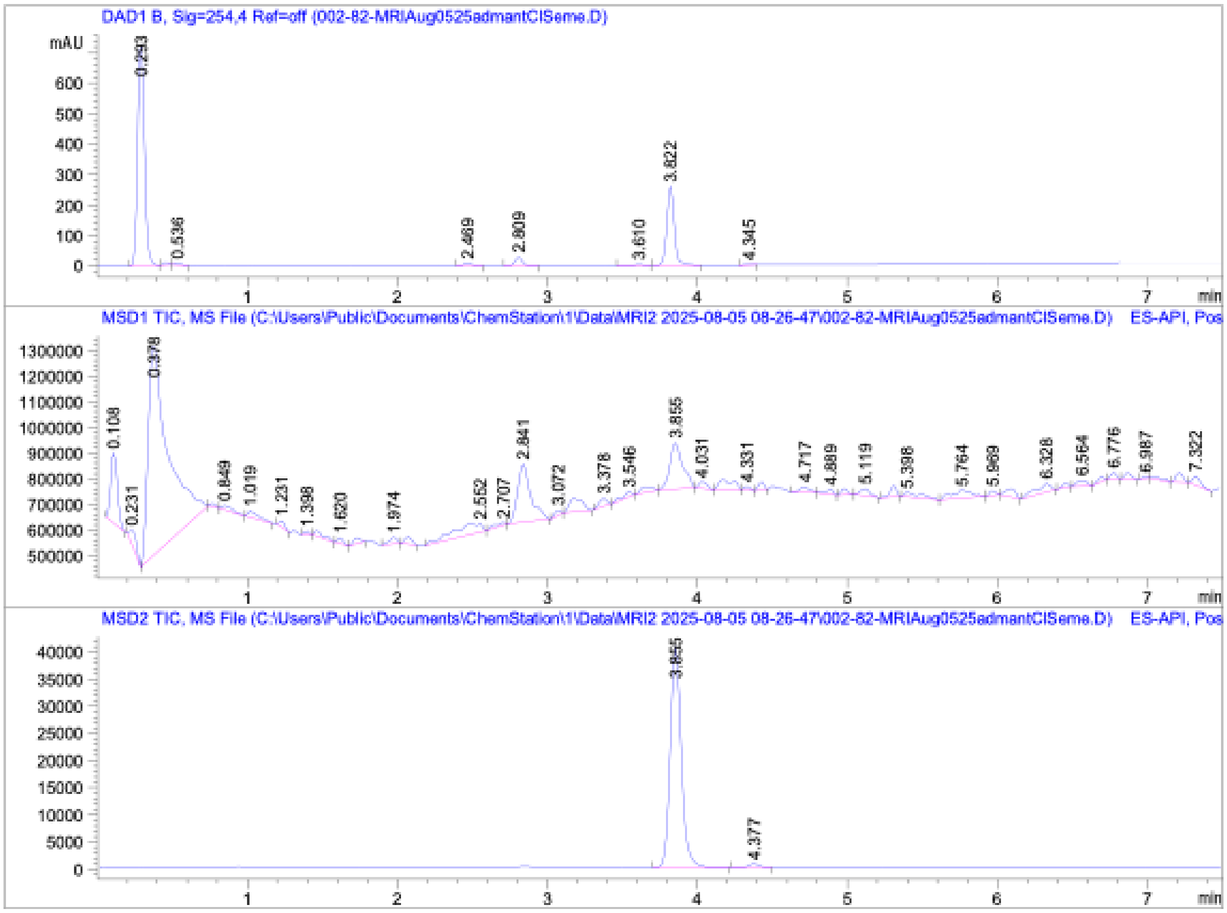

Figure S3. LCMS traces for compound **39**

β-Arrestin Assay Protocol[13]

Validated MPXNomad-CB1 cell line (Innoprot) was used to assay compounds for internalization mediated by β-Arrestin. Briefly MPXNomad-CB1 HEK293 cell line was thawed (3x106 cells per T25) and cells were maintained in DMEM supplemented with 10% FBS at 37 °C in a humidified 5% CO2 atmosphere. Cells were plated at a concentration of 30,000 cells/plate in a 96-well plate and maintained in DMEM medium supplemented with 10% FBS during 24h at 37 °C in a humidified 5% CO2. Cells were incubated with the test compounds overnight diluted in OptiMEM and CP55940 (EC_{80} concentration). Compounds were tested in triplicate at each concentration. The medium was replaced with 100 µl of PBS to perform the fluorescence intensity acquisition. For excitation and emission peaks were measured at 482 nm and 502 nm, respectively. Data was plotted and curves generated using and pIC_{50} calculated using GraphPad Prism 10. Datapoints represent the mean ± SD for each condition in experiments performed in triplicate.

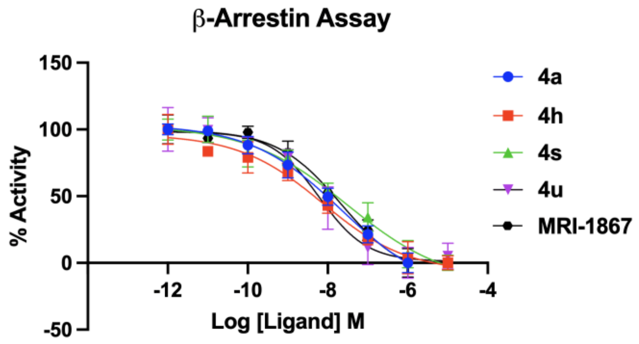

Figure S4. Functional CB_1R antagonism was determined for select compounds using β-Arrestin assay with CB1 Nomad cell line (Innoprot) in HEK293K cells in the presence of CB1 agonist CP55,940 (at EC_{80} concentration). Data was plotted and curves generated using and pIC50 calculated using GraphPad Prism 10. Datapoints represent the mean ± SD for each condition in experiments performed in triplicate.

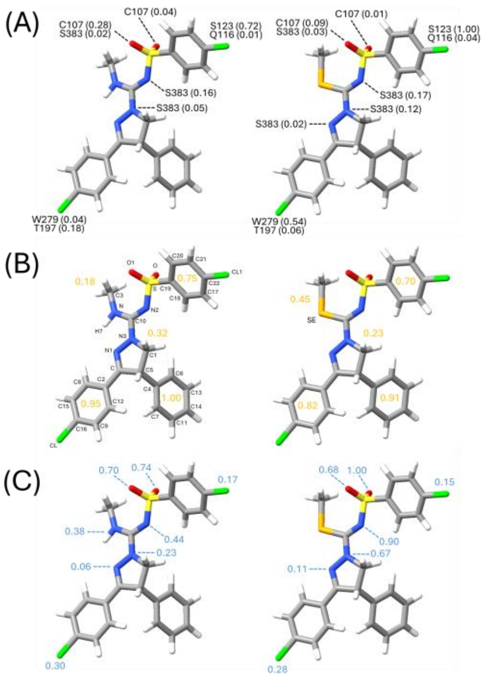

	-NH-	-Se-	
F102	0.26	0.47	N-ter
M103	0.18	0.26	
F108	0.48	1.00	
F189	0.01	0.11	TMH3
L193	0.03	0.13	
I267	0.08	0.14	ECL2
F268	0.59	0.65	
P269	0.01	0.10	
F379	0.36	0.75	TMH7

Figure S5. Ligand interactions with CB_1R and water observed in the simulations (cf. Methods). (A) Polar ligand–receptor interactions, with relative strengths (in parentheses) normalized to the highest value (Cl of Arm 3 vs. S123 in TMH1; see animation V1 for the location of residues in the three-dimensional structural context. (B) Total nonpolar interactions of each ligand arm and the central ring with the receptor, calculated as the sum over the corresponding carbon atoms and normalized to the highest value; a per-residue decomposition for Arm 4 is provided in the accompanying table (see animation V1). (C) Main stabilizing interactions with water, with differences in hydration occurring primarily around Arm 4 and the -SO_2 group; See the three-dimensional water distributions in animations V2 and V3, showing an overlay of hydration water from snapshots taken at equally spaced time points. The thickness of the interaction lines in Fig. 6 reflects the relative strengths shown here. Quantitative data are derived from the simulation trajectories using the provided scripts, and the uploaded data files enable additional analyses beyond the scope of this study.

Full structures of all compounds from selenomethylation (Manuscript Figure 3) and Seleno augmentation (Manuscript Figure 4)

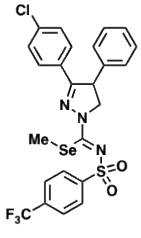
4a
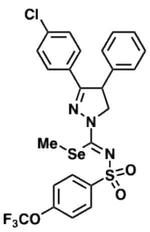
4b
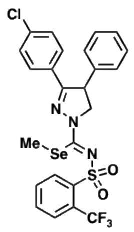
4c
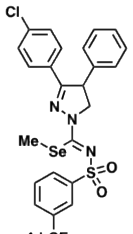
4d
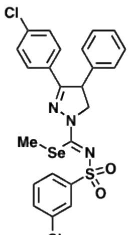
4e
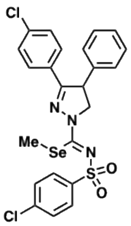
4f
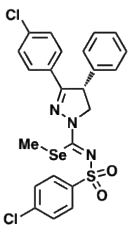
4g
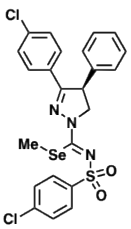
4h
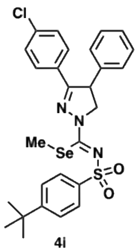
4i
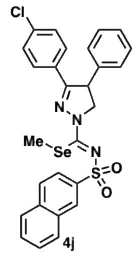
4j
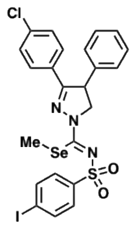
4k
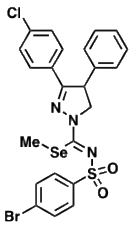
4l
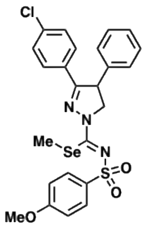
4m
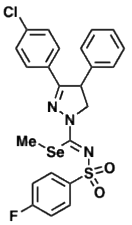
4n
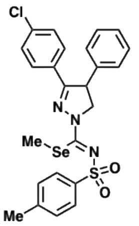
4o
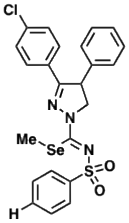
4p
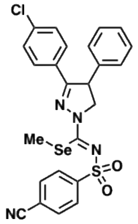
4q
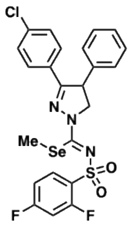
4r
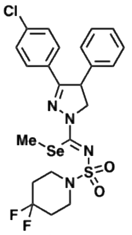
4s
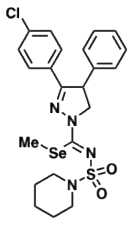
4t
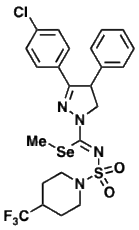
4u
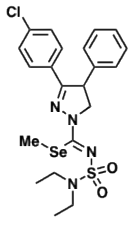
4v
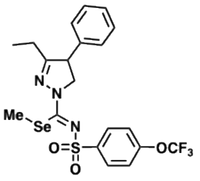
7a
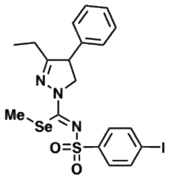
7b
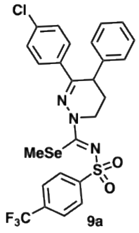
9a
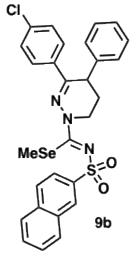
9b
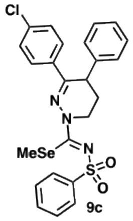
9c

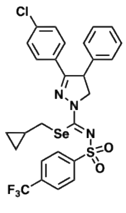
10
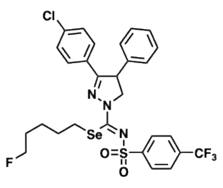
11
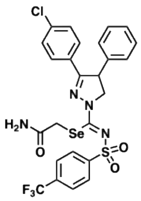
12
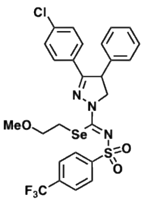
13
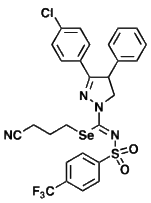
14
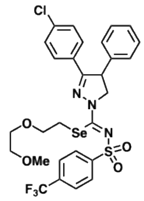
15
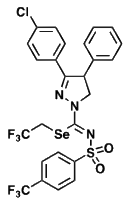
16
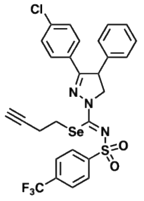
17
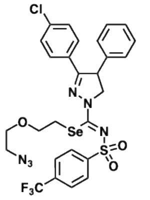
18
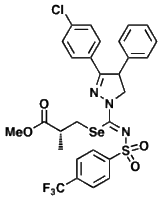
19
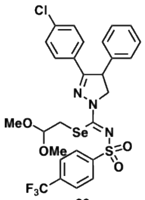
20
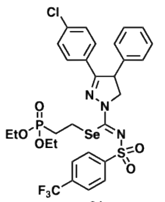
21
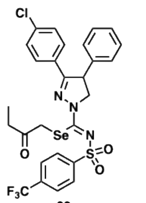
22
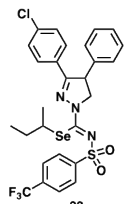
23
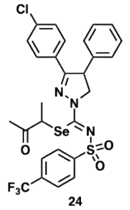
24
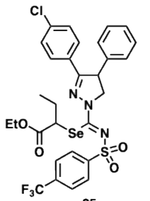
25
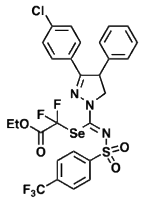
26
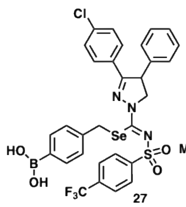
27
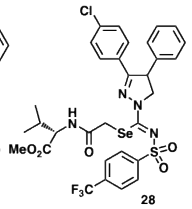
28
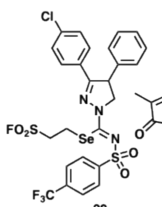
29
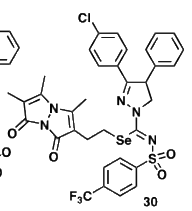
30
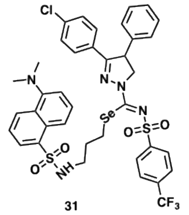
31
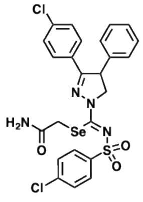
32
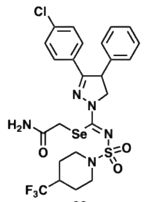
33
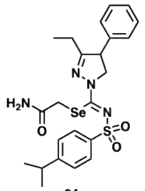
34
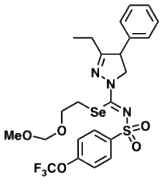
35
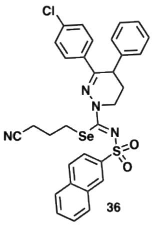
36
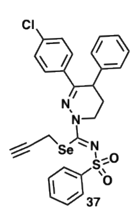
37
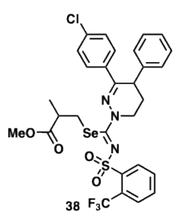
38

Bibliography

(1) Iyer, M. R.; Cinar, R.; Coffey, N. J.; Chorvat, R. J.; Kunos, G. Synthesis of S-2-((S)-3-(4-Chlorophenyl)-N'-((4-Chlorophenyl)Sulfonyl)-4-Phenyl-4,5-Dihydro-1H-Pyrazole-1-Carboximidamido)-3-(Methyl-D3)Butanamide-D5 , Octadeuterated JD5037. *J. Labelled Comp. Radiopharm.* **2017**, *60*, 460–465.

(2) Lange, J. H. M.; Coolen, H. K. A. C.; van Stuivenberg, H. H.; Dijksman, J. A. R.; Herremans, A. H. J.; Ronken, E.; Keizer, H. G.; Tipker, K.; McCreary, A. C.; Veerman, W.; Wals, H. C.; Stork, B.; Verveer, P. C.; den Hartog, A. P.; de Jong, N. M. J.; Adolfs, T. J. P.; Hoogendoorn, J.; Kruse, C. G. Synthesis, Biological Properties, and Molecular Modeling Investigations of Novel 3,4-Diarylpyrazolines as Potent and Selective CB(1) Cannabinoid Receptor Antagonists. *J. Med. Chem.* **2004**, *47*, 627–643.

(3) Iyer, M. R.; Bhattacharjee, P.; Kundu, B.; Rutland, N.; Wood, C. M. One-Pot Synthesis of Thio-Augmented Sulfonylureas via a Modified Bunte's Reaction. *ACS Omega* **2022**.

(4) Solvay Pharmaceuticals B.V. 4,5-Dihydro-LH-Pyrazole Derivatives having CB1 -Antagonistic Activity. WO 02/076949 A L, October 3, 2002.

(5) Iyer, M. R.; Cinar, R.; Katz, A.; Gao, M.; Erdelyi, K.; Jourdan, T.; Coffey, N. J.; Pacher, P.; Kunos, G. Design, Synthesis, and Biological Evaluation of Novel, Non-Brain-Penetrant, Hybrid Cannabinoid CB1R Inverse Agonist/Inducible Nitric Oxide Synthase (INOS) Inhibitors for the Treatment of Liver Fibrosis. *J. Med. Chem.* **2017**, *60*, 1126–1141.

(6) Iyer, M. R.; Cinar, R.; Wood, C. M.; Zawatsky, C. N.; Coffey, N. J.; Kim, K. A.; Liu, Z.; Katz, A.; Abdalla, J.; Hassan, S. A.; Lee, Y.-S. Synthesis, Biological Evaluation, and Molecular Modeling Studies of 3,4-Diarylpyrazoline Series of Compounds as Potent, Nonbrain Penetrant Antagonists of Cannabinoid-1 (CB1R) Receptor with Reduced Lipophilicity. *J. Med. Chem.* **2022**, *65*, 2374–2387.

(7) Lange, J. H. M.; den Hartog, A. P.; van der Neut, M. A. W.; van Vliet, B. J.; Kruse, C. G. Synthesis and SAR of 1,4,5,6-Tetrahydropyridazines as Potent Cannabinoid CB1 Receptor Antagonists. *Bioorg. Med. Chem. Lett.* **2009**, *19*, 5675–5678.

(8) Bhattacharjee, P.; Dvorácskó, S.; Pointeau, O.; Kundu, B.; Rutland, N.; Puhl, H.; Liu, J.; Godlewski, G.; Hassan, S. A.; Jourdan, T.; Cinar, R.; Iyer, M. R. Evaluation of Tetrahydropyridazine-Based Peripherally Restricted Dual Inhibitors of CB1R and Inducible Nitric Oxide Synthase (iNOS) for Treating Metabolic Syndrome Disorders. *Metab. Clin. Exp.* **2025**, *170*, 156291.

(9) Kundu, B.; Dvorácskó, S.; Basu, A.; Pommerolle, L.; Kim, K. A.; Wood, C. M.; Gibbs, E.; Behee, M.; Tarasova, N. I.; Cinar, R.; Iyer, M. R. Evaluation of the Therapeutic Potential of Sulfonyl Urea Derivatives as Soluble Epoxide Hydrolase (Seh) Inhibitors. *Molecules* **2024**, *29*, 3036.

(10) Shivshankar, S.; Nimely, J.; Puhl, H.; Iyer, M. R. Pharmacological Evaluation of Cannabinoid Receptor Modulators Using Grabecb2.0 Sensor. *Int. J. Mol. Sci.* **2024**, *25*.

(11) Liu, Z.; Iyer, M. R.; Godlewski, G.; Jourdan, T.; Liu, J.; Coffey, N. J.; Zawatsky, C. N.; Puhl, H. L.; Wess, J.; Meister, J.; Liow, J.-S.; Innis, R. B.; Hassan, S. A.; Lee, Y. S.; Kunos, G.; Cinar, R. Functional Selectivity of a Biased Cannabinoid-1 Receptor (CB1R) Antagonist. *ACS Pharmacol. Transl. Sci.* **2021**, *4*, 1175–1187.

(12) Kumari, P.; Dvorácskó, S.; Enos, M. D.; Ramesh, K.; Lim, D.; Hassan, S. A.; Kunos, G.; Cinar, R.; Iyer, M. R.; Rosenbaum, D. M. Structural Mechanism of CB1R Binding to Peripheral and Biased Inverse Agonists. *Nat. Commun.* **2024**, *15*, 10694.

(13) Mella, R. M.; Kortazar, D.; Roura-Ferrer, M.; Salado, C.; Valcárcel, M.; Castilla, A.; Villacé, P. Nomad Biosensors: A New Multiplexed Technology for the Screening of GPCR Ligands. *SLAS Technol.* **2018**, *23*, 207–216.
