# Supplementary material for: Unlocking Selenium Chemical Space via a Programmable Synthesis Platform Bearing Cannabinoid Receptor Recognition Motifs
Source: J Am Chem Soc. 2026 Apr 28;148(18):18649–59. doi: 10.1021/jacs.5c16359 (PMC13185125; doi:10.1021/jacs.5c16359)

^{1}HNMR and ^{13}CNMR files for

Unlocking selenium chemical space via a programmable synthesis platform bearing cannabinoid receptor recognition motifs

Malliga R. Iyer,[1] Pinaki Bhattacharjee,[1] Subhradeep Dutta,[1] Maloba M.M. Lobe,[1#] Paul D.Volesky,[1] Grzegorz Godlewski,[2] Henry L. Puhl III,[3] Sergio A Hassan[4]*

[1]Section on Medicinal Chemistry, National Institute on Alcohol Abuse and Alcoholism (NIAAA), National Institutes of Health (NIH), 5625 Fishers Lane, Rockville, MD 20852, USA, [2]Laboratory of Physiologic Studies, National Institute on Alcohol Abuse and Alcoholism, National Institutes of Health, 5625 Fishers Lane, Rockville, MD 20852, USA. [3]Laboratory of Biophotonics and Quantum Biology, National Institute on Alcohol Abuse and Alcoholism, National Institutes of Health, 5625 Fishers Lane, Rockville, MD 20852, USA. [4]Bioinformatics and Computational Biosciences Branch, National Institute of Allergy and Infectious Diseases, National Institutes of Health, Bethesda, MD 20892, USA.

Corresponding author: malliga.iyer@nih.gov [#] Current affiliation: Department of Chemistry, University of Buea, Cameroon

^{1}HNMR and ^{13}CNMR of **4a**

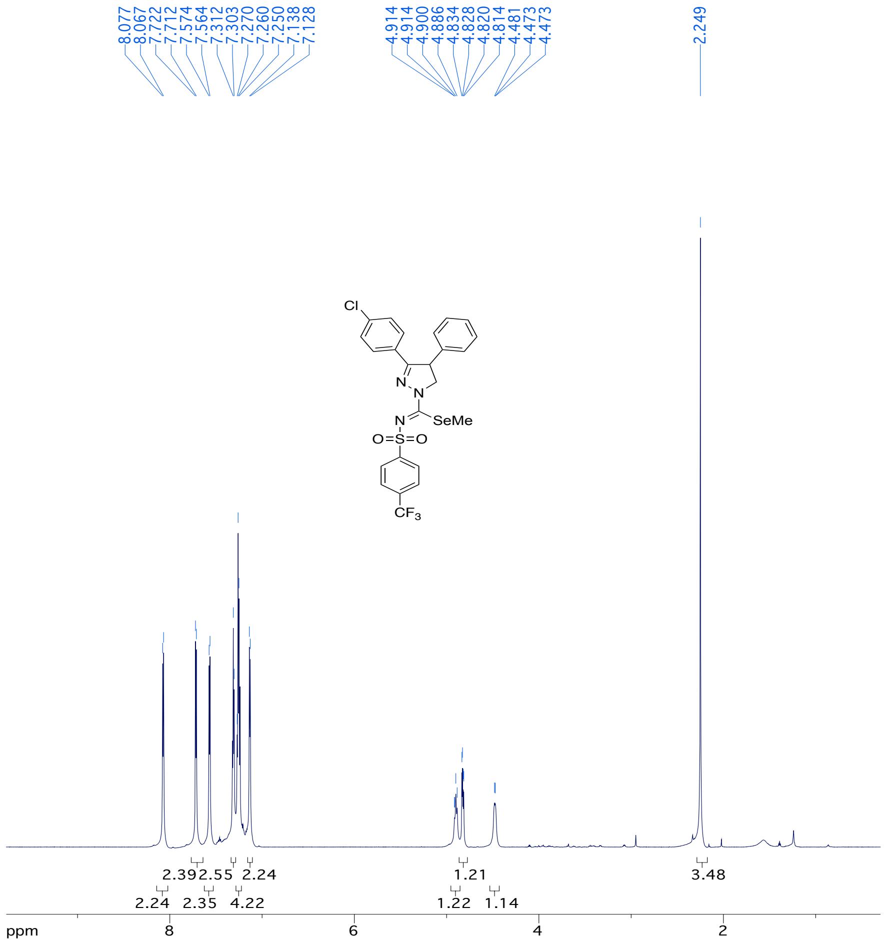

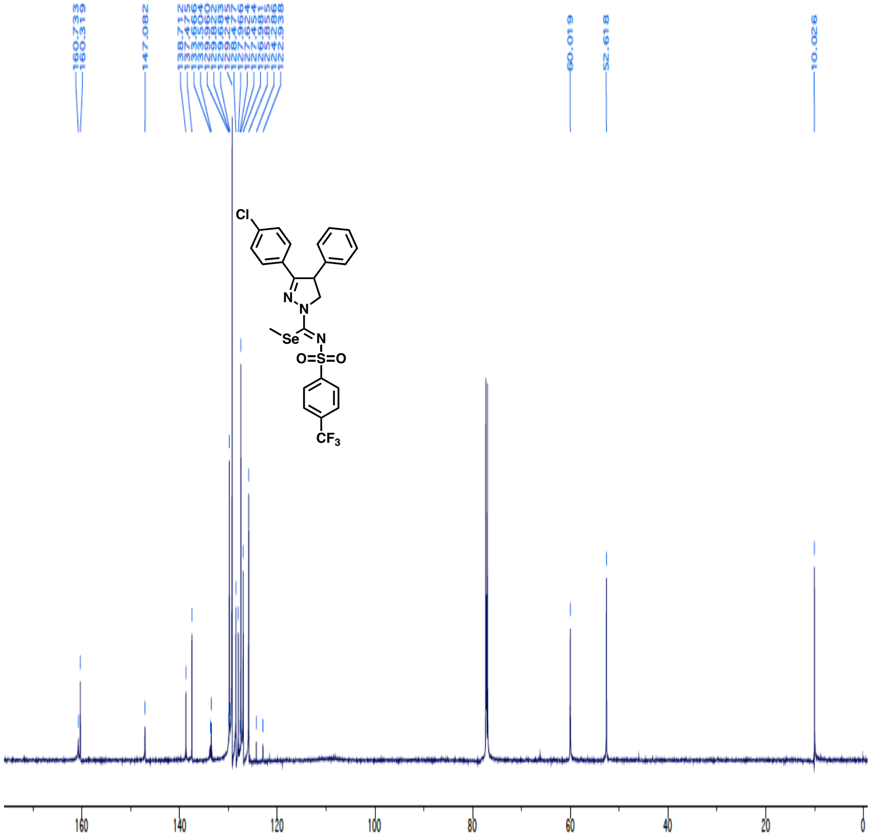

^{1}HNMR and ^{13}CNMR of **4b**

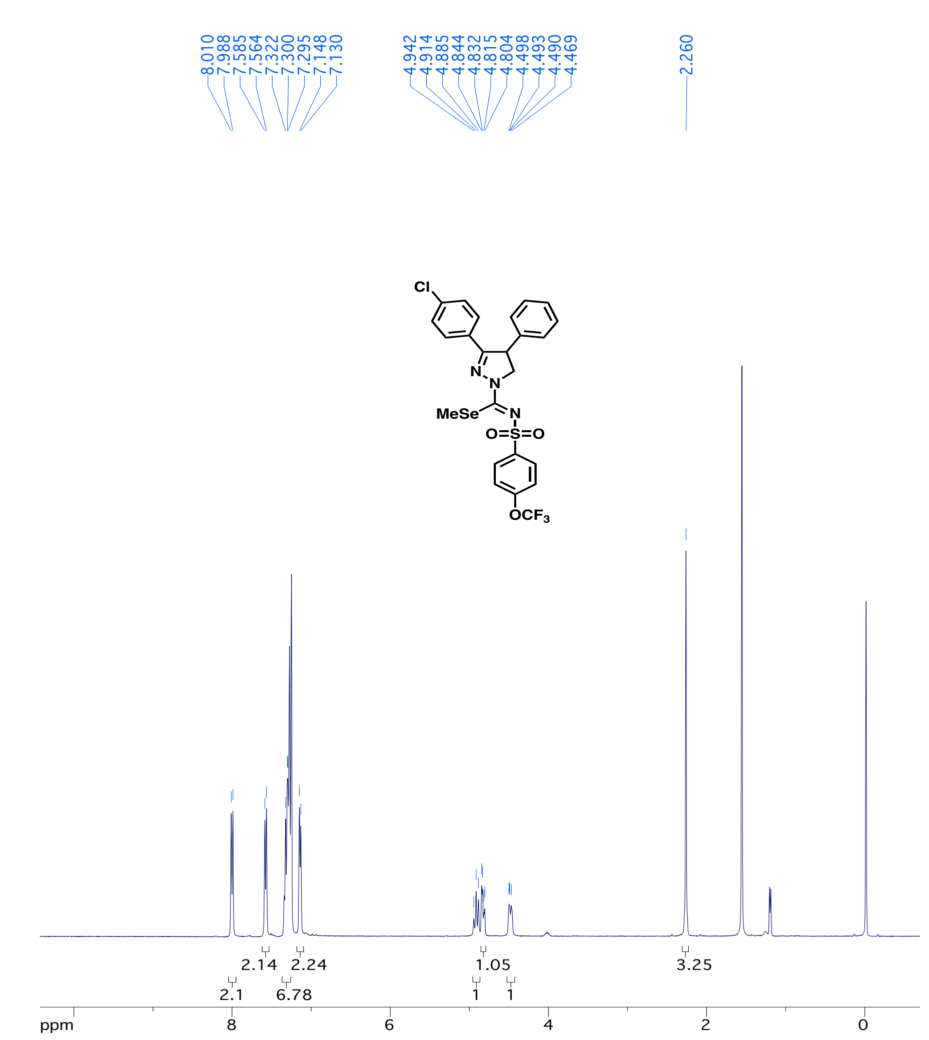

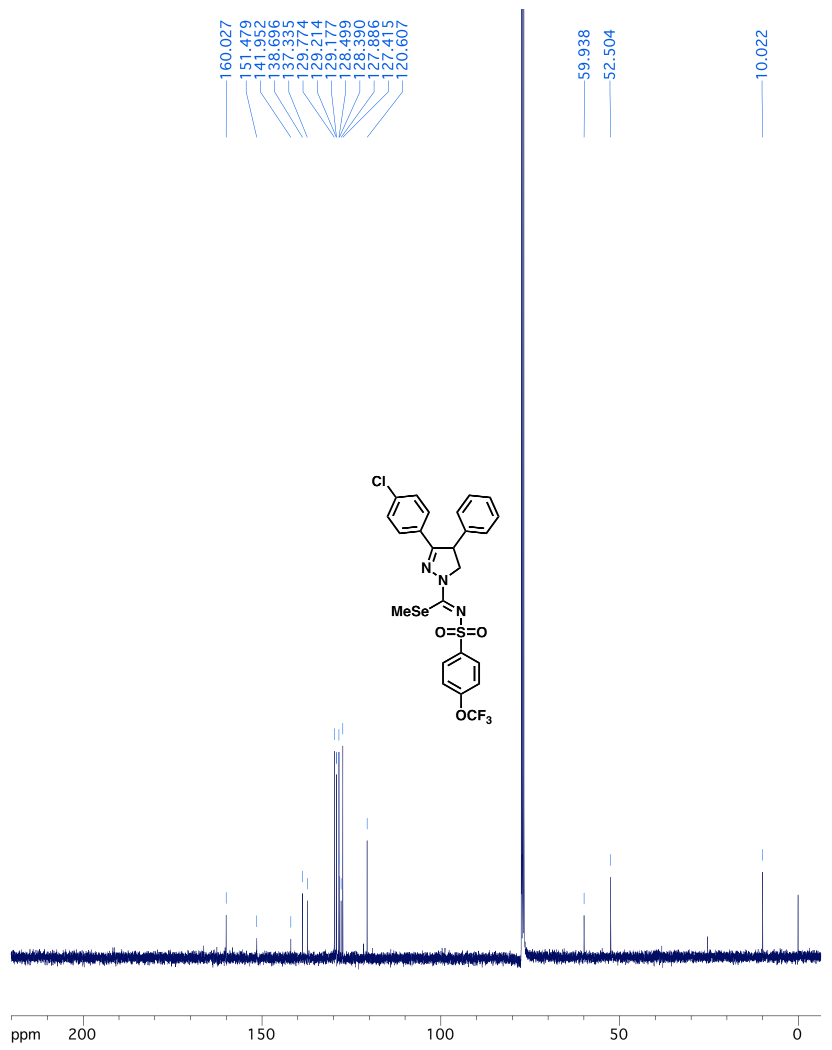

^{1}HNMR and ^{13}CNMR of **4c**

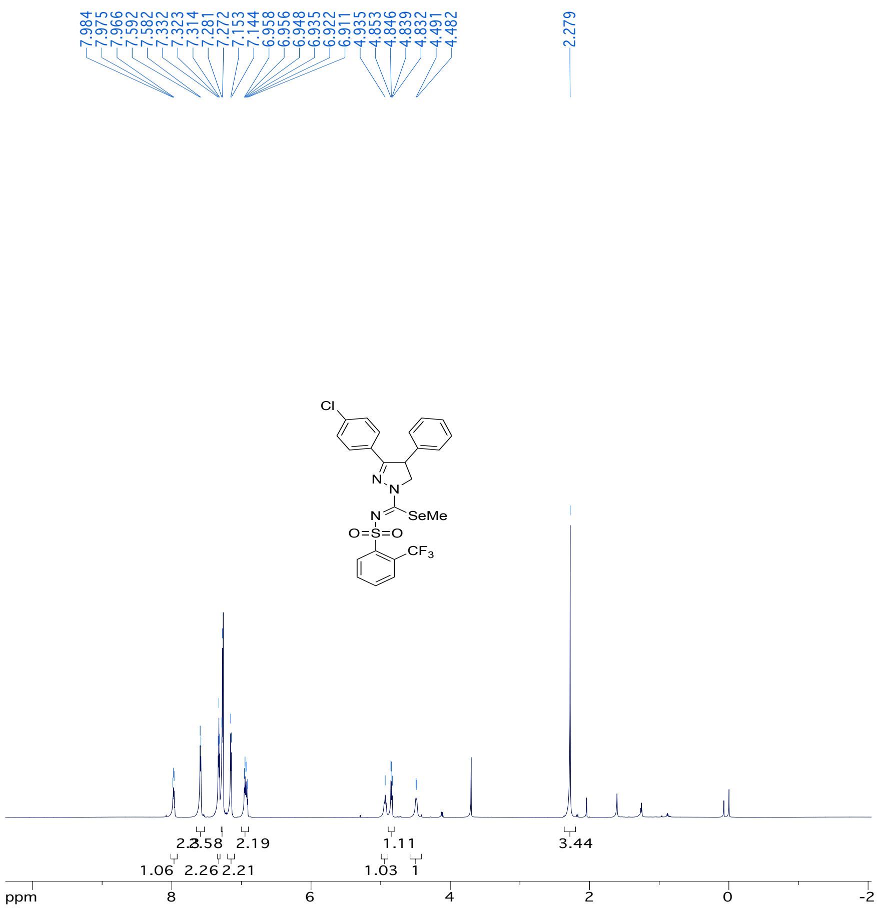

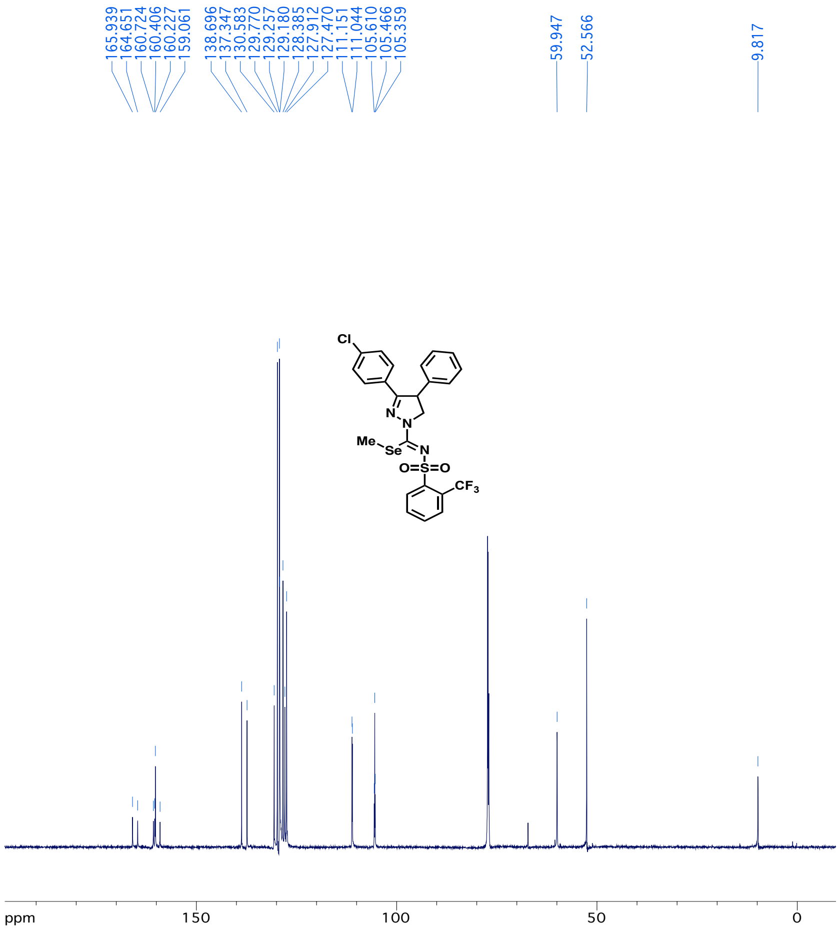

^{1}HNMR and ^{13}CNMR of **4d**

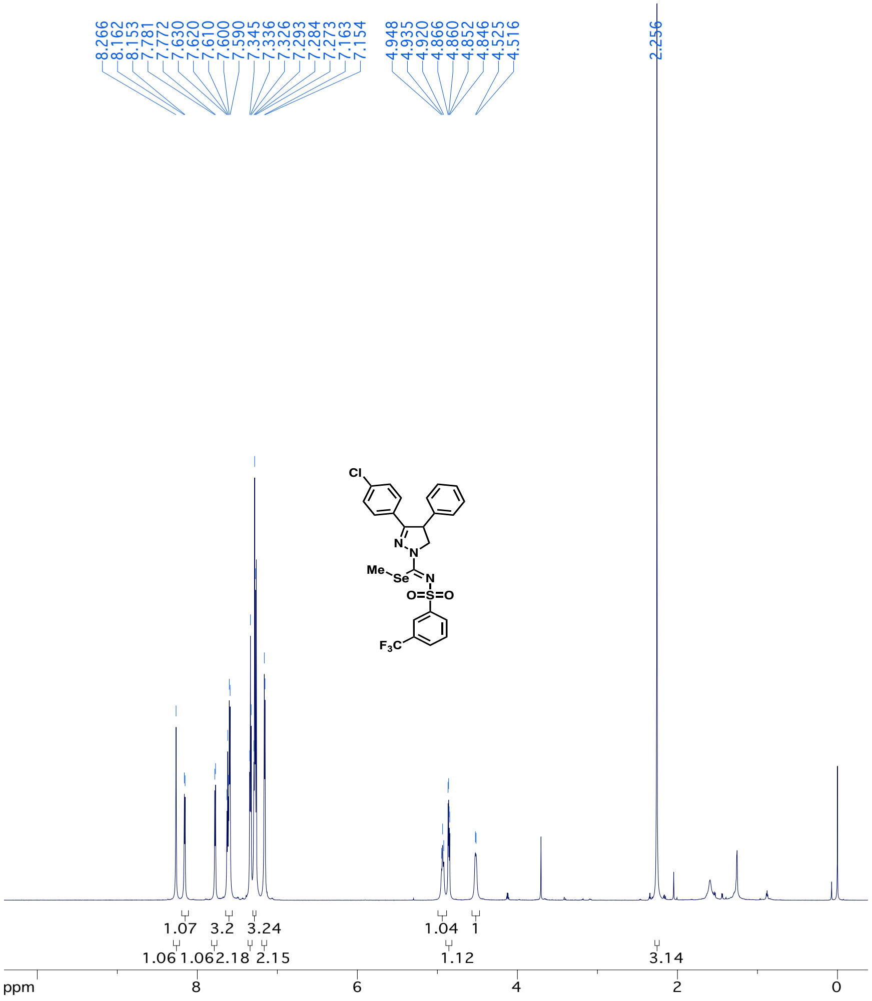

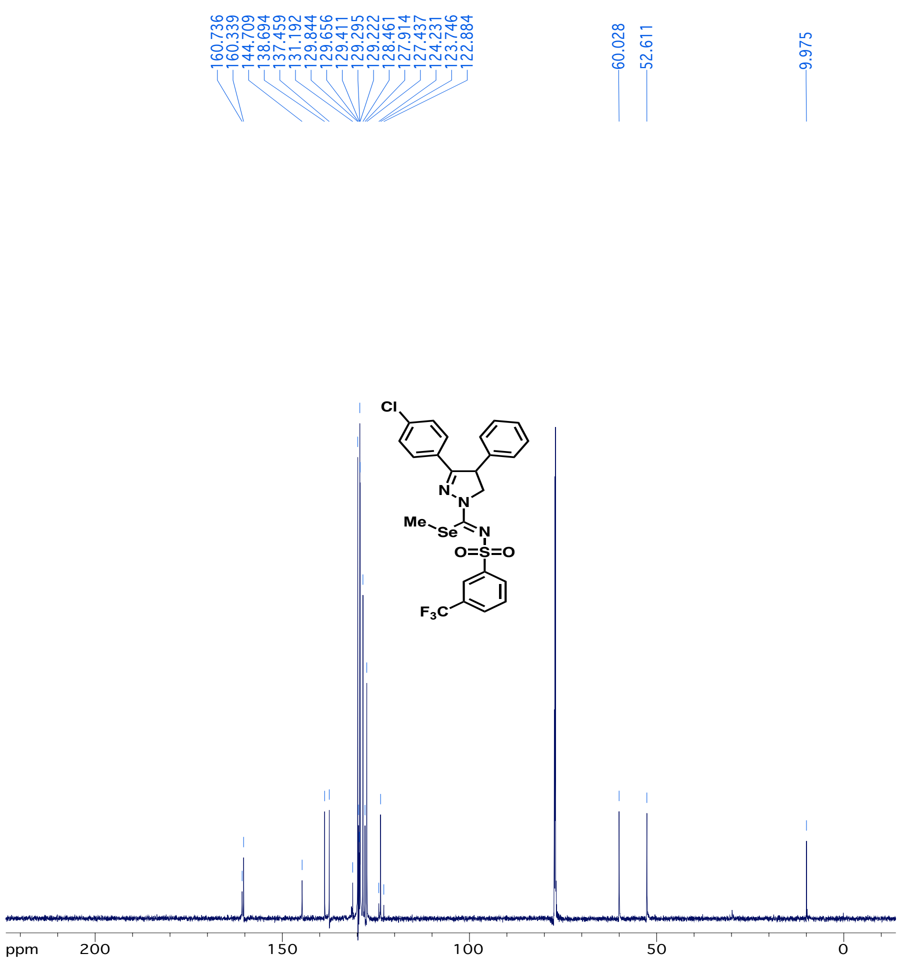

^{1}HNMR and ^{13}CNMR of **4e**

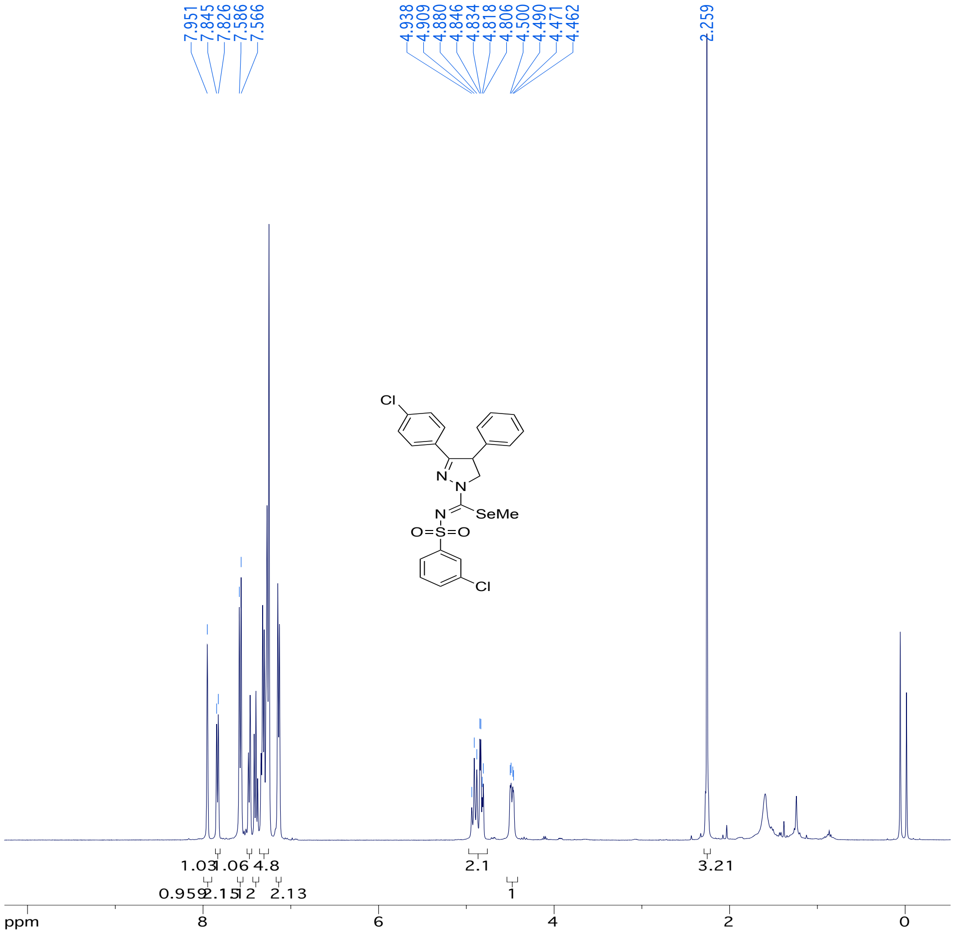

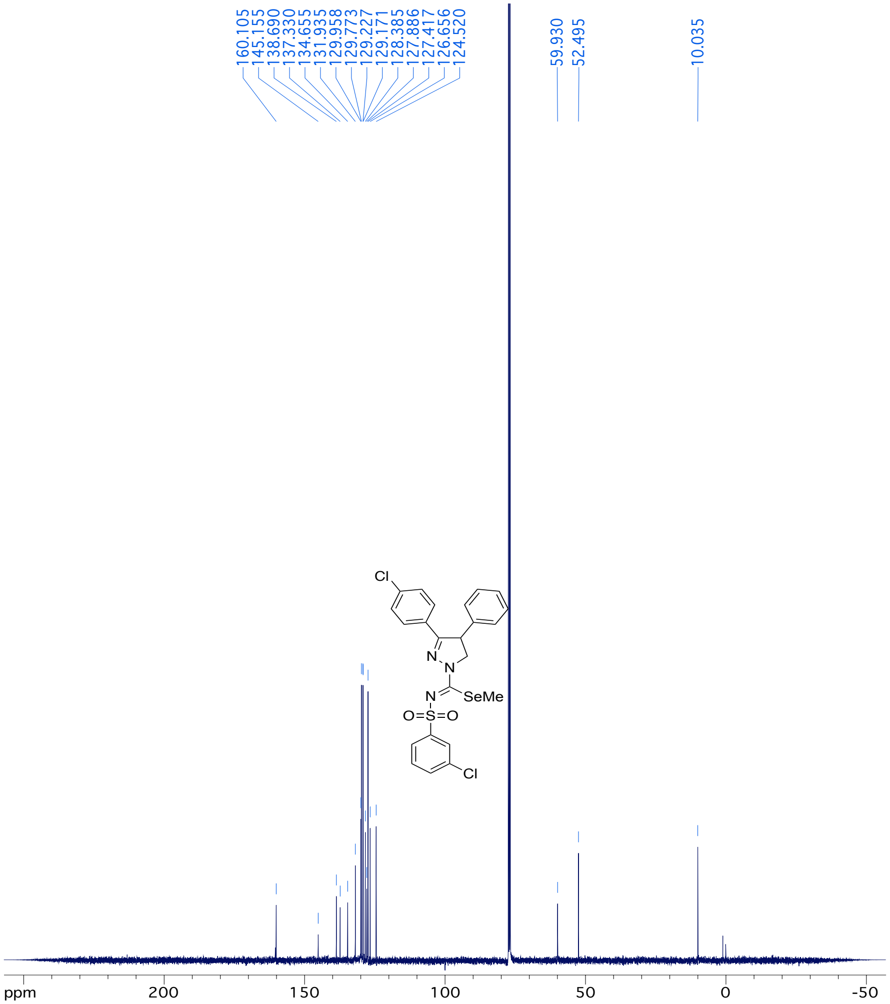

^{1}HNMR and ^{13}CNMR of **4f**

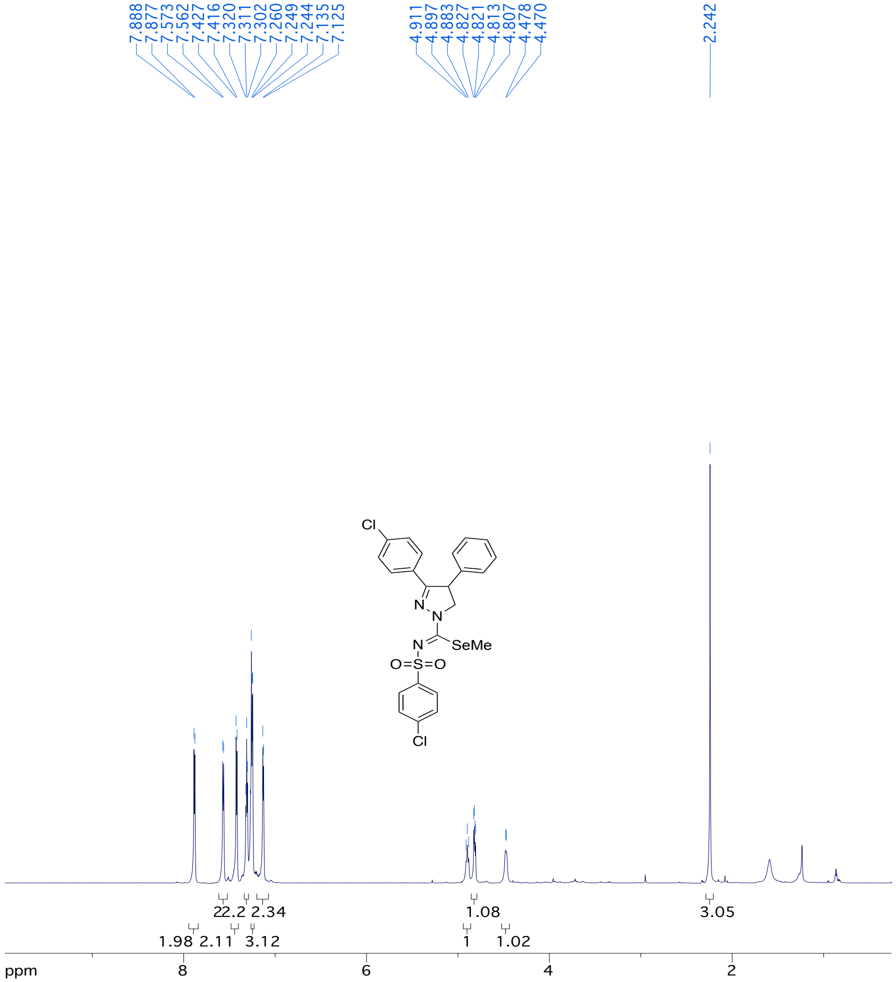

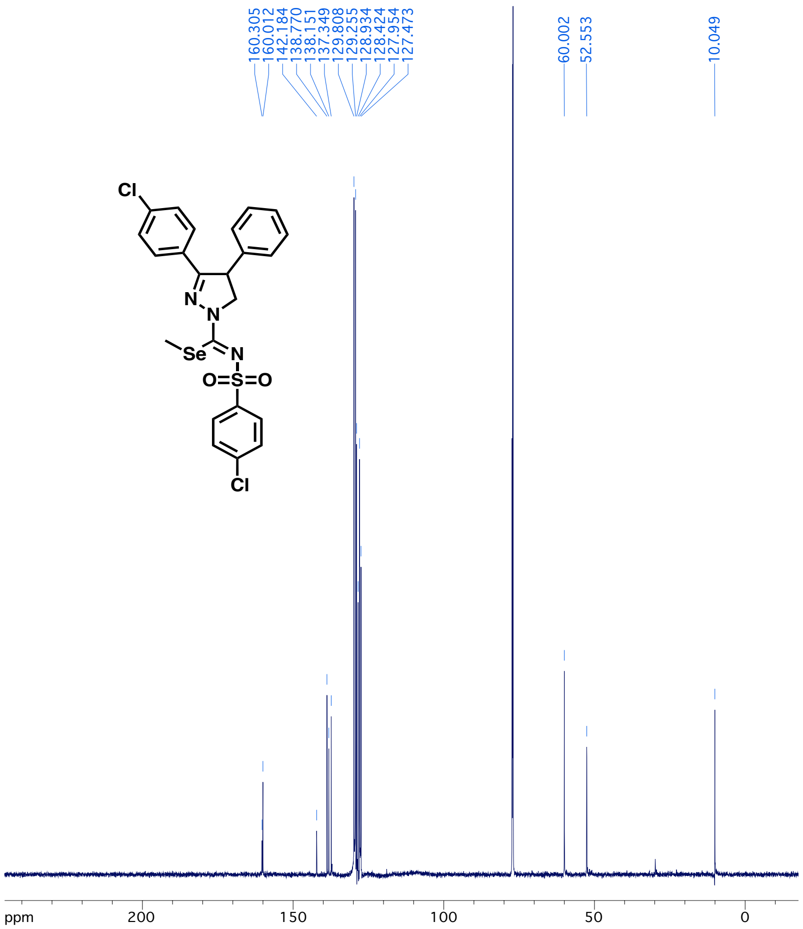

^{1}HNMR and ^{13}CNMR of **4g**

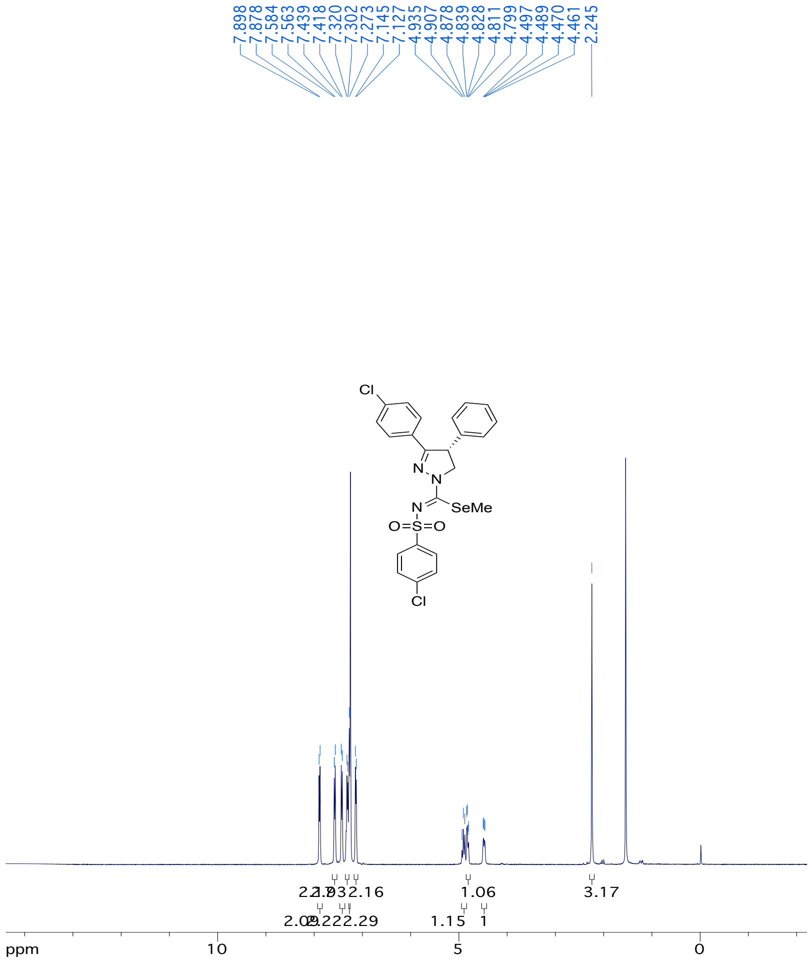

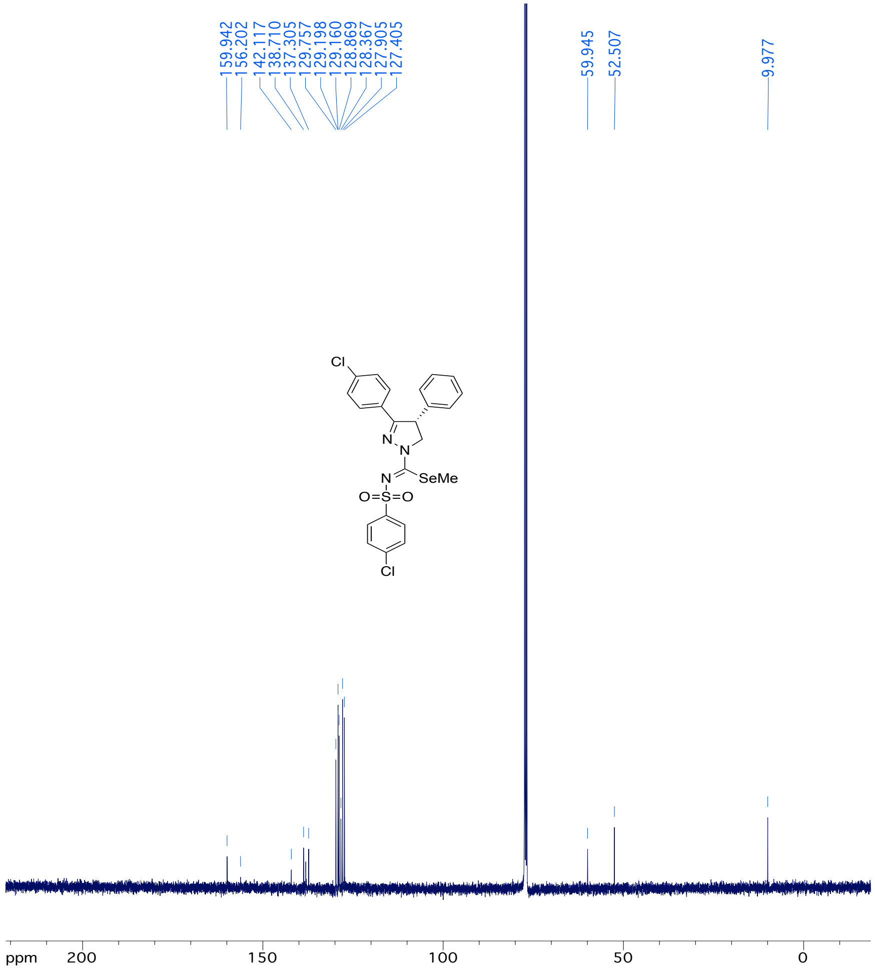

^{1}HNMR and ^{13}CNMR of **4i**

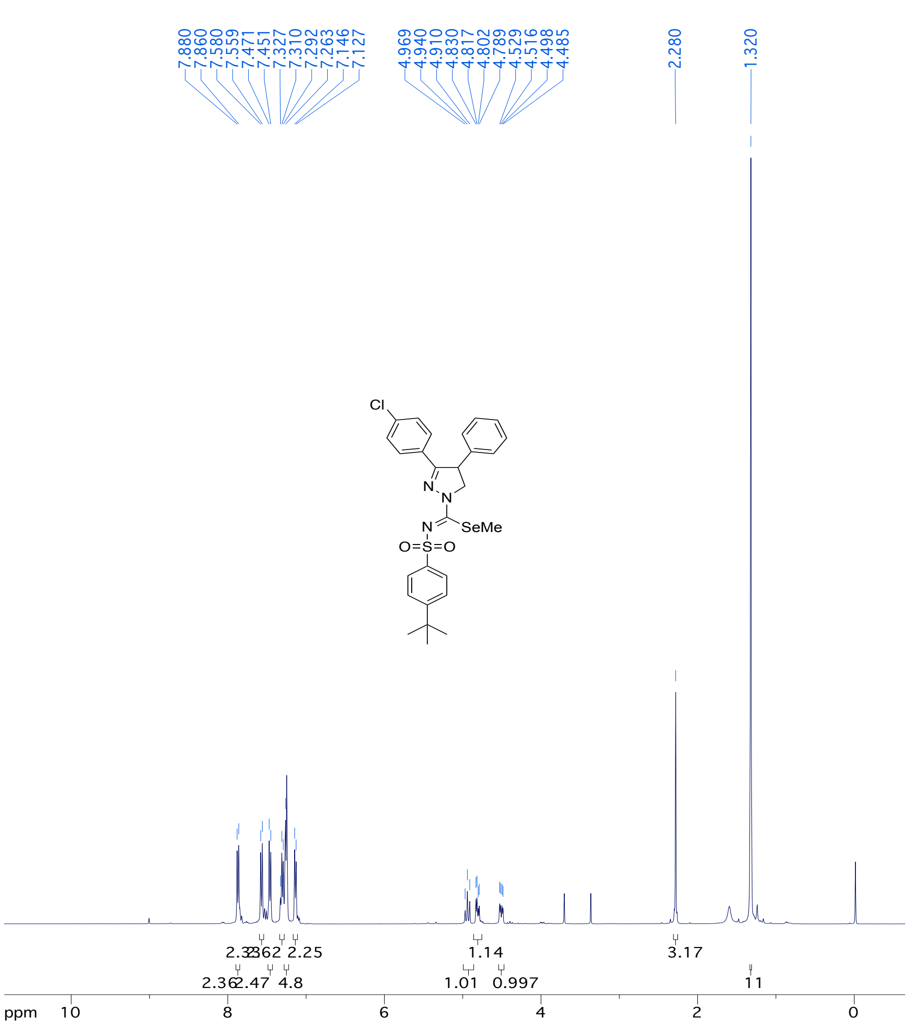

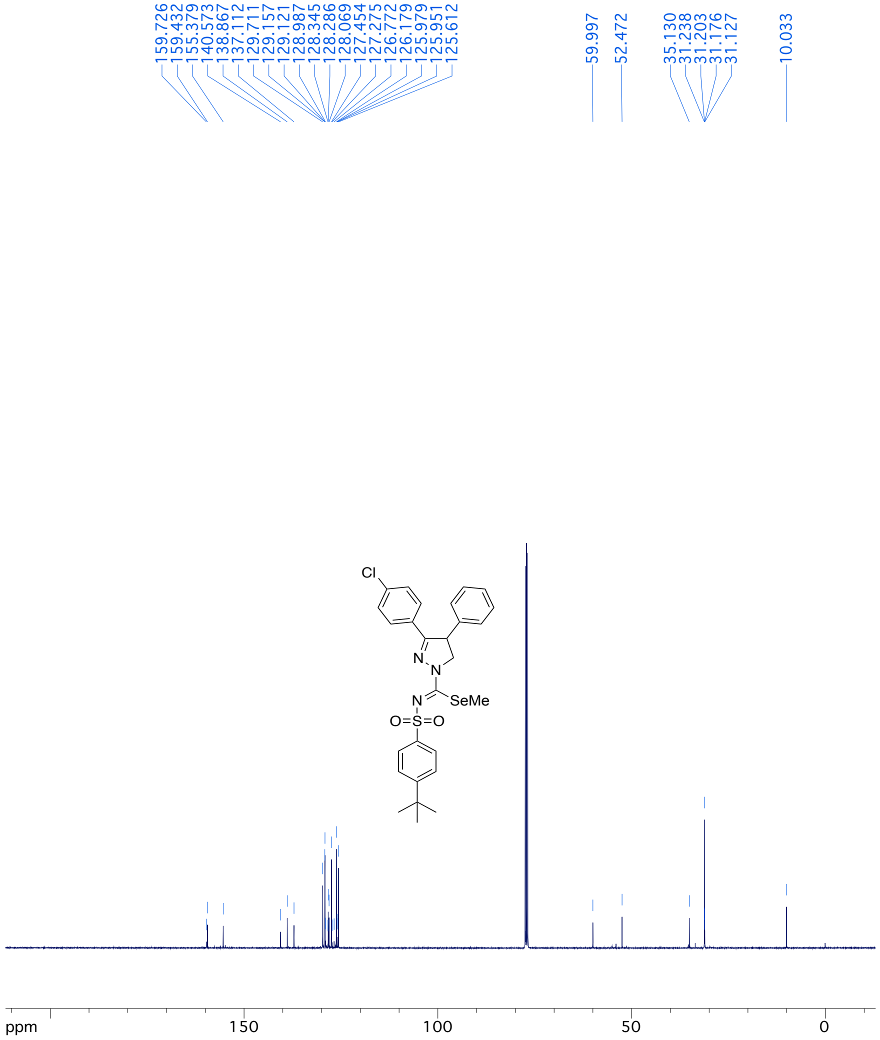

^{1}HNMR and ^{13}CNMR of **4j**

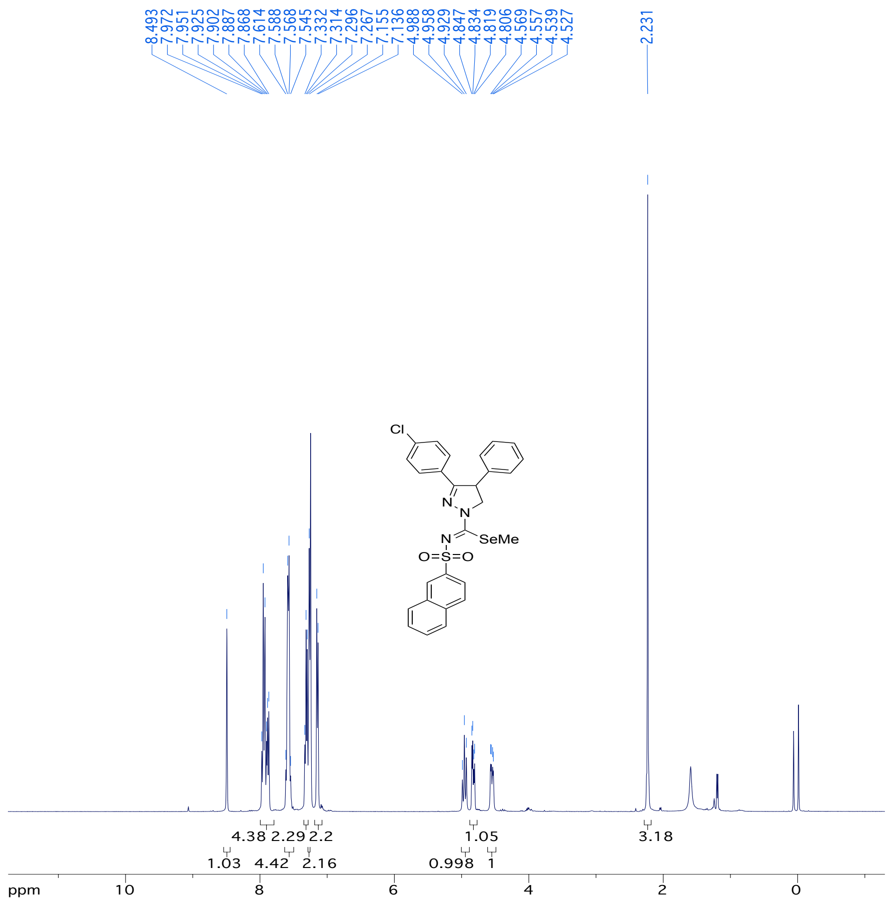

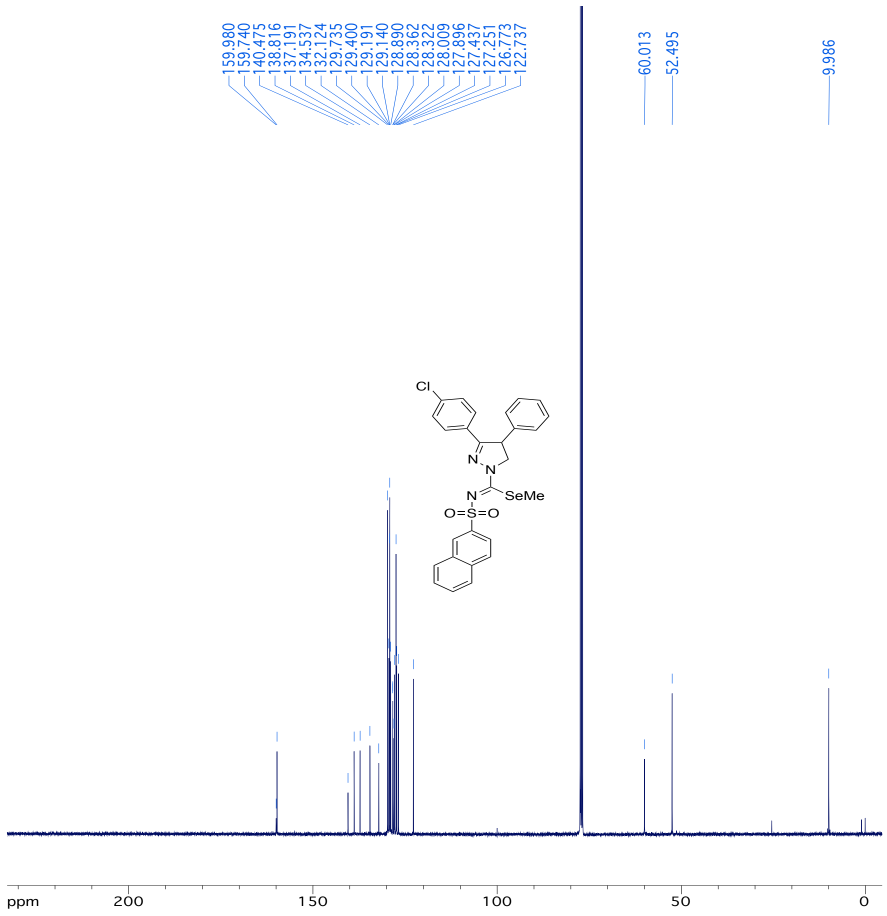

^{1}HNMR and ^{13}CNMR of **4k**

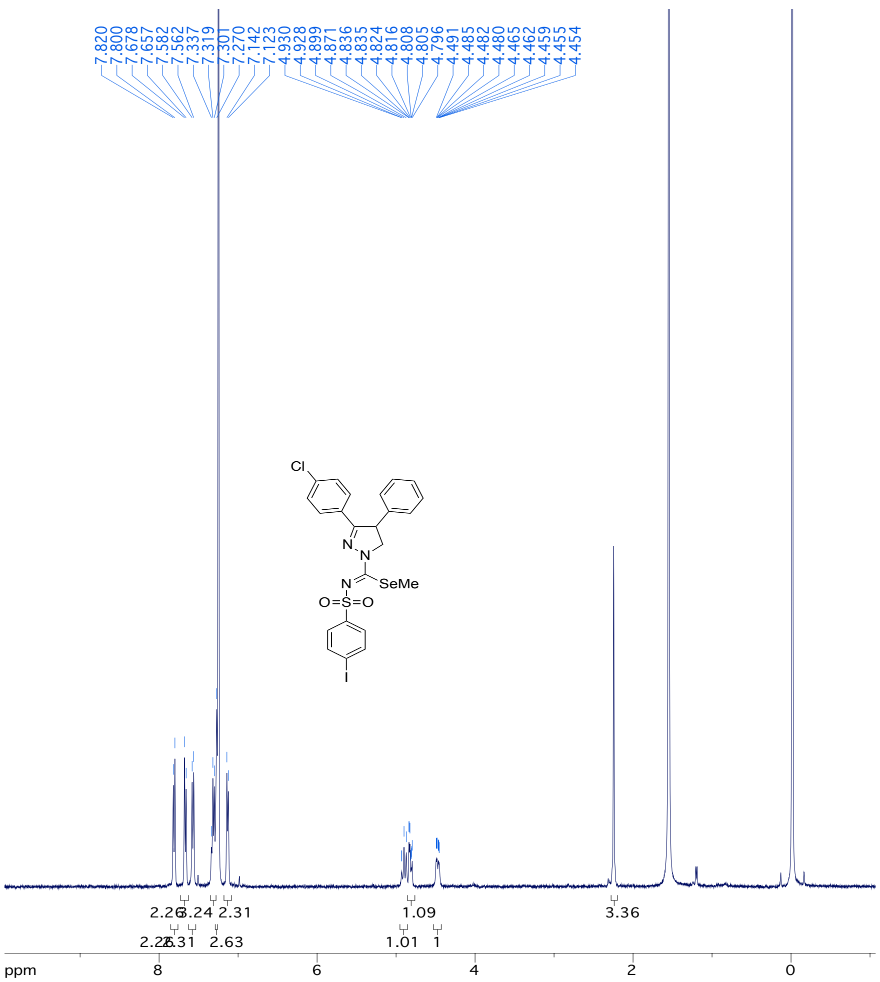

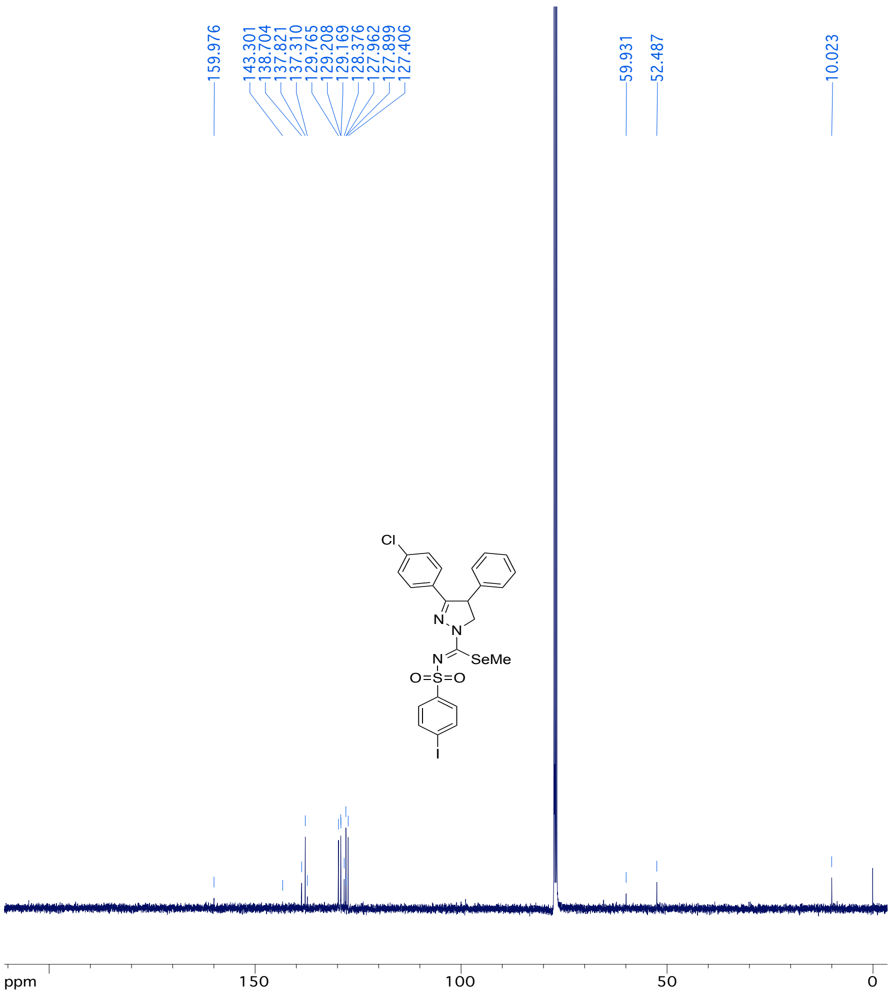

^{1}HNMR and ^{13}CNMR of **4l**

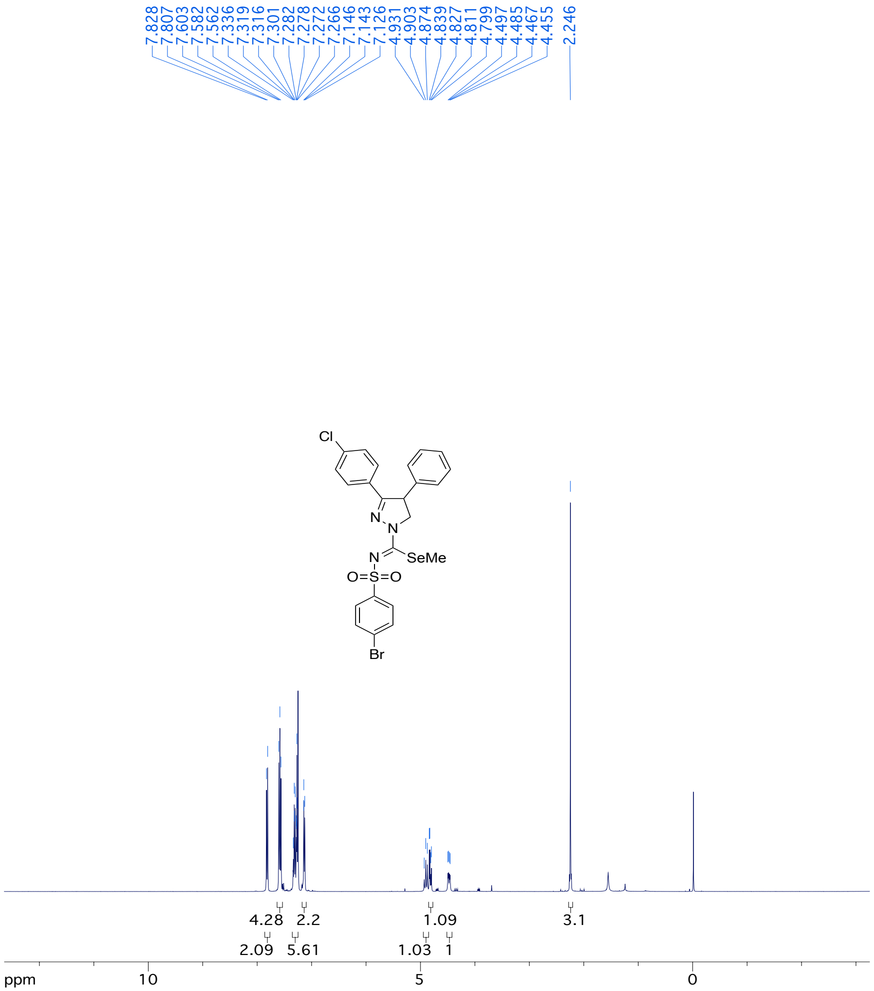

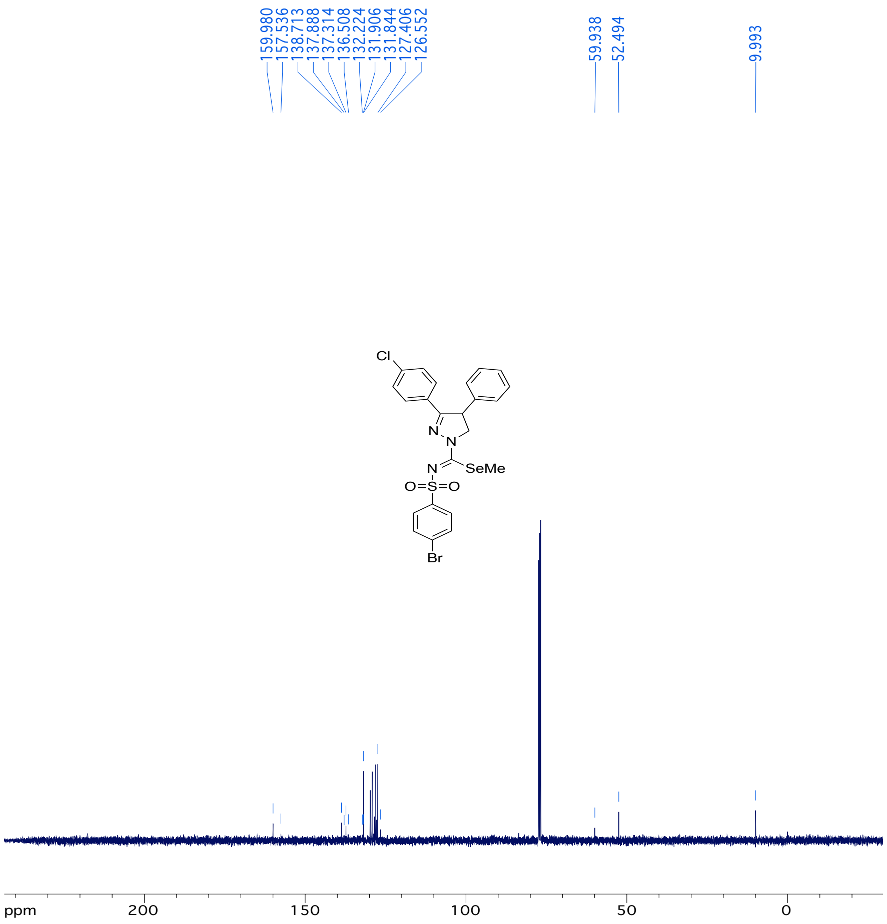

^{1}HNMR and ^{13}CNMR of **4m**

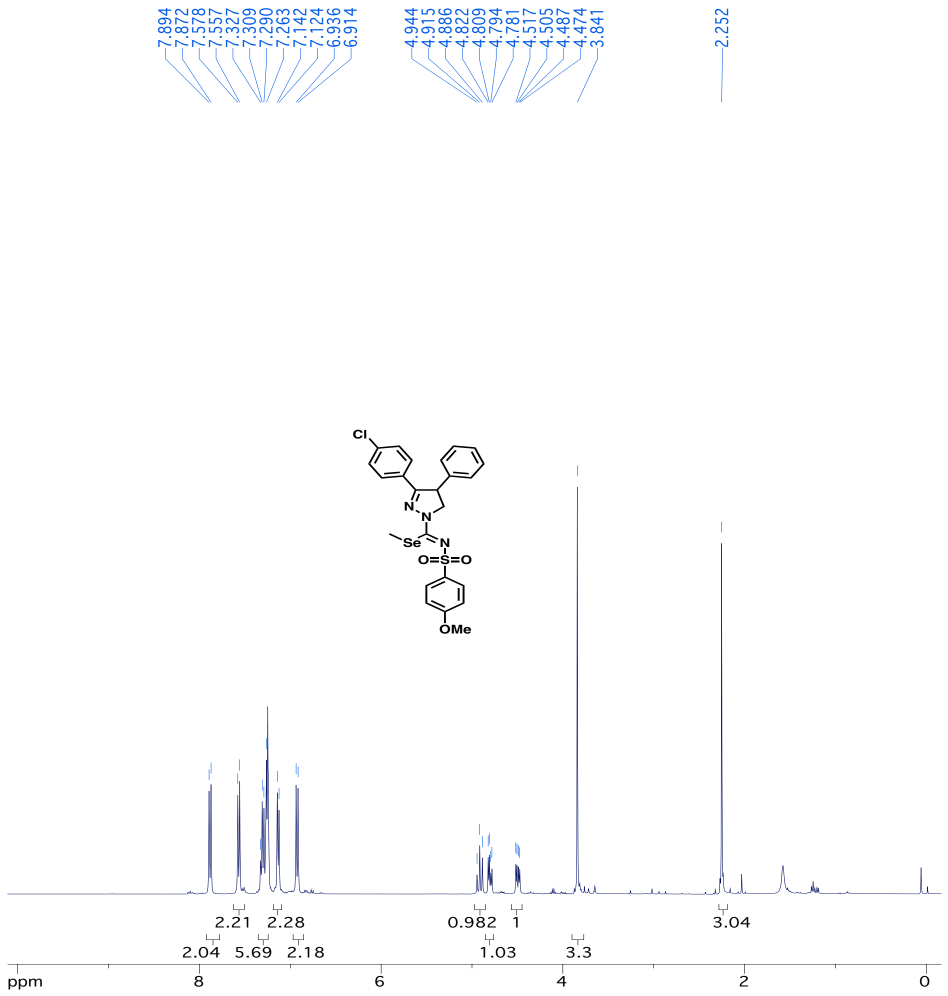

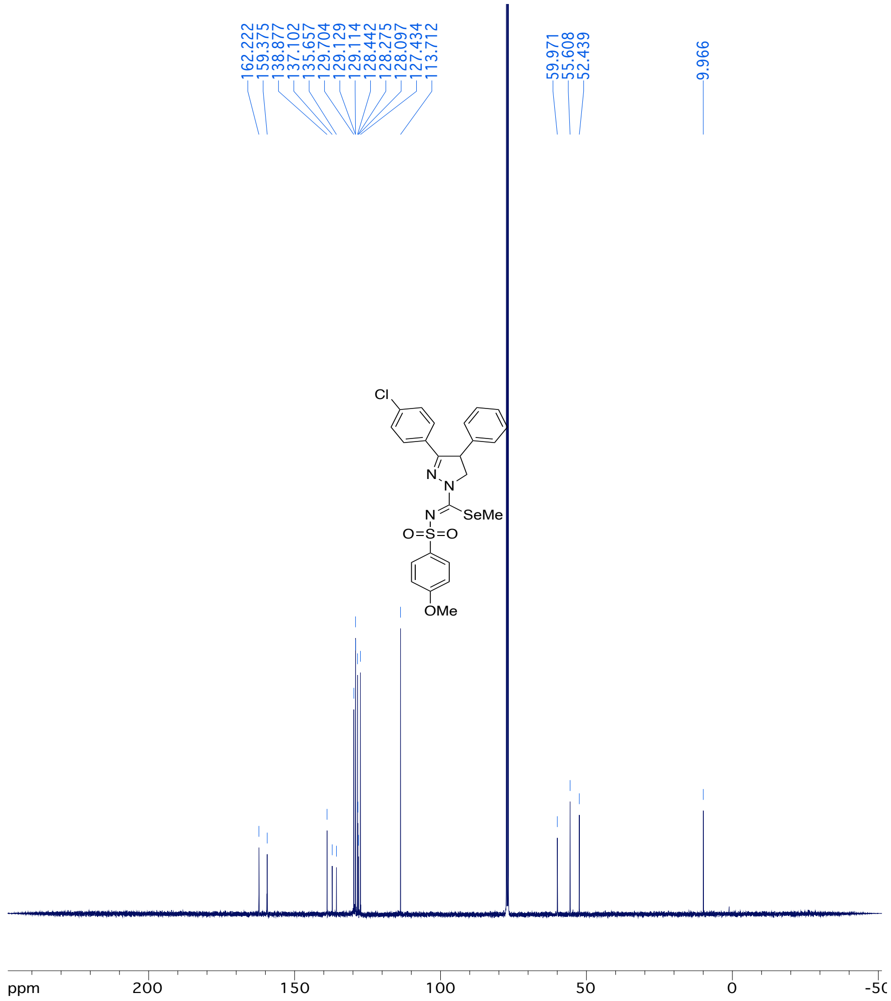

^{1}HNMR and ^{13}CNMR of **4n**

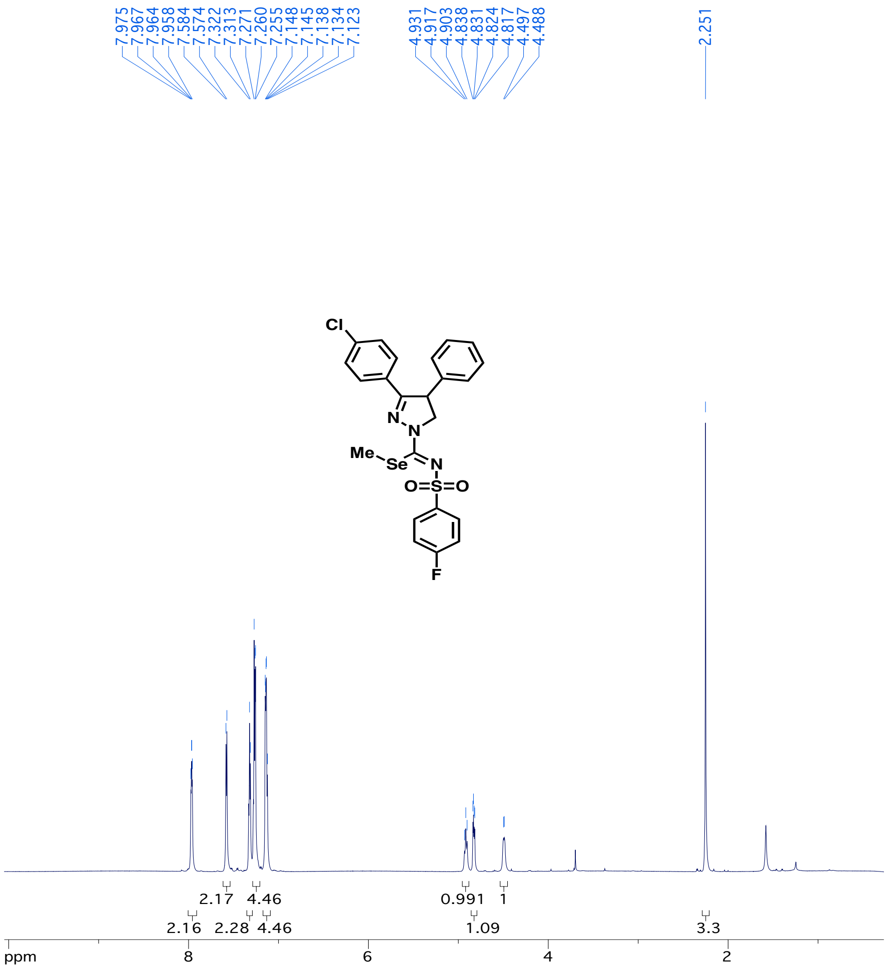

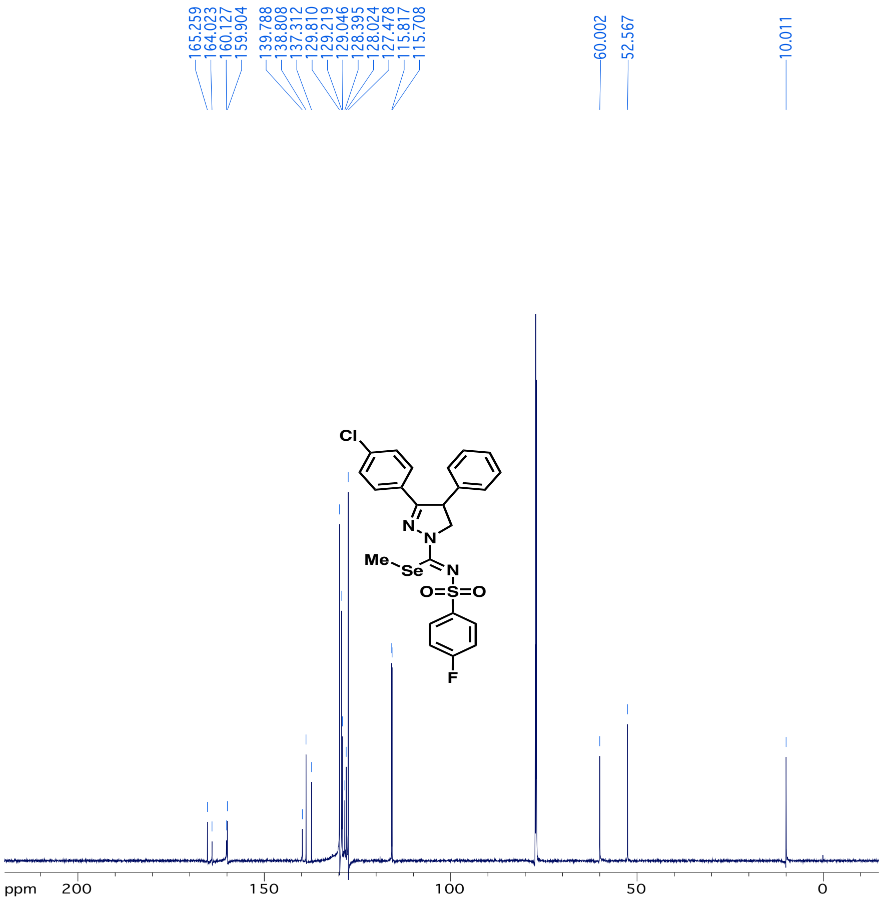

^{1}HNMR and ^{13}CNMR of **4o**

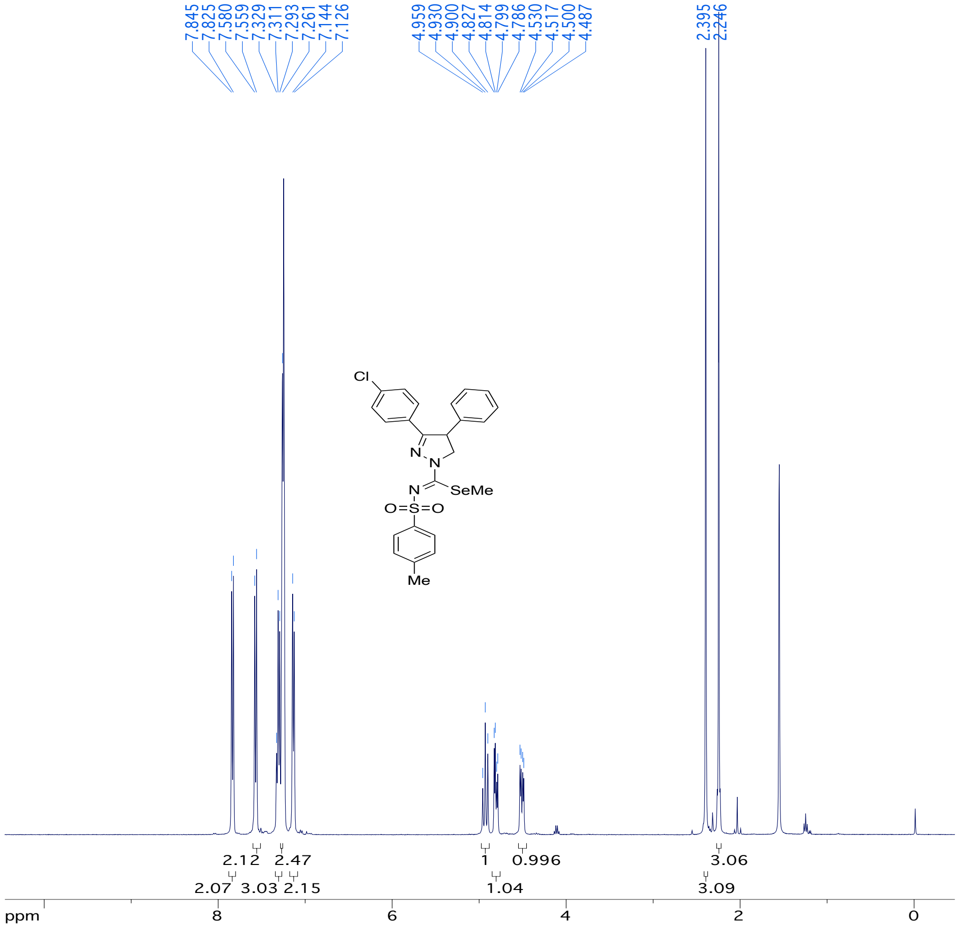

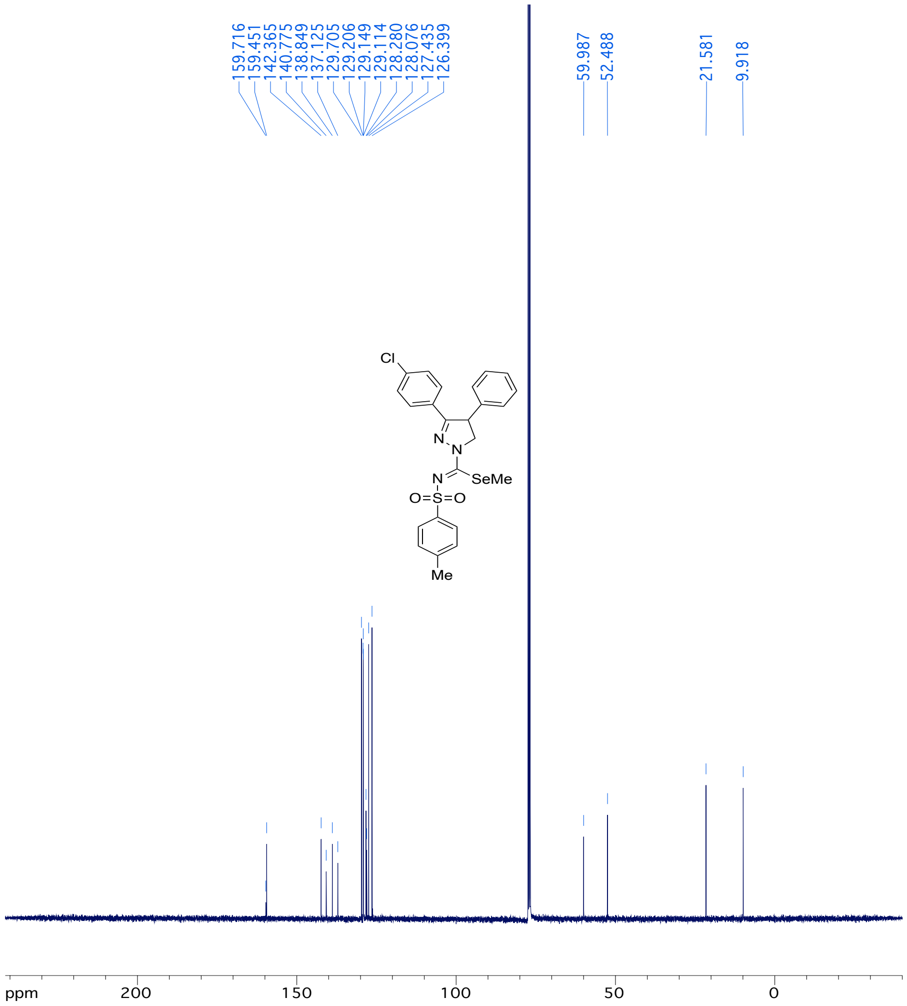

^{1}HNMR and ^{13}CNMR of **4p**

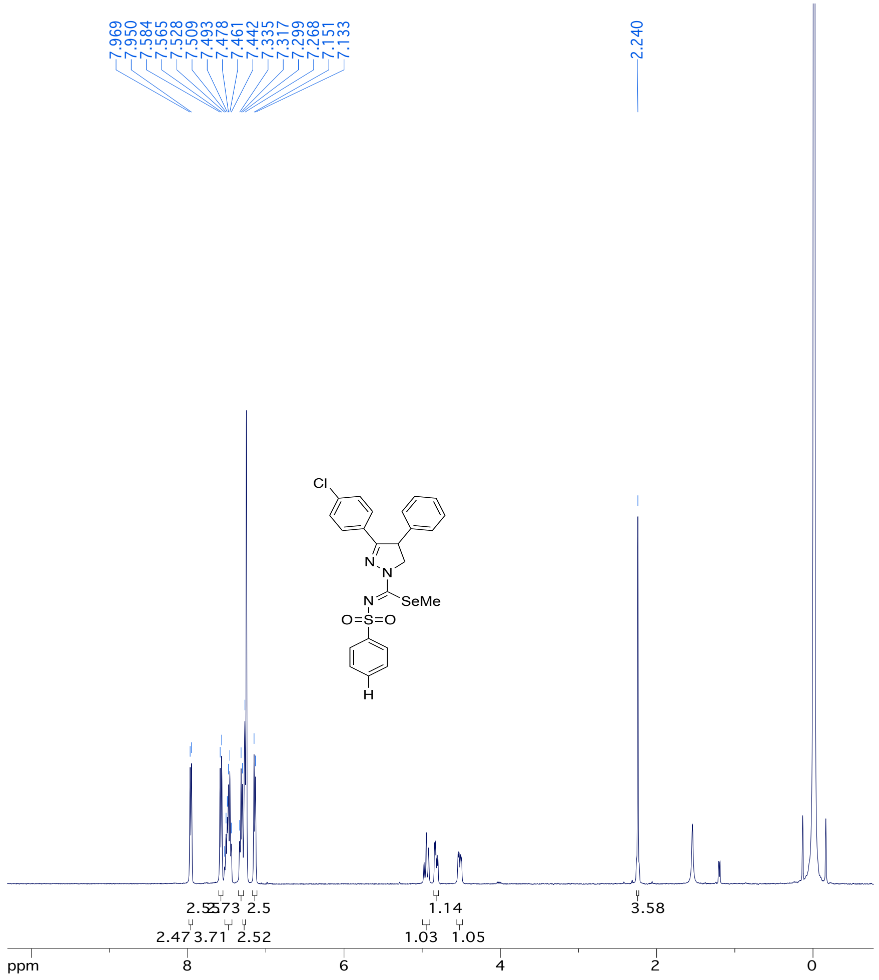

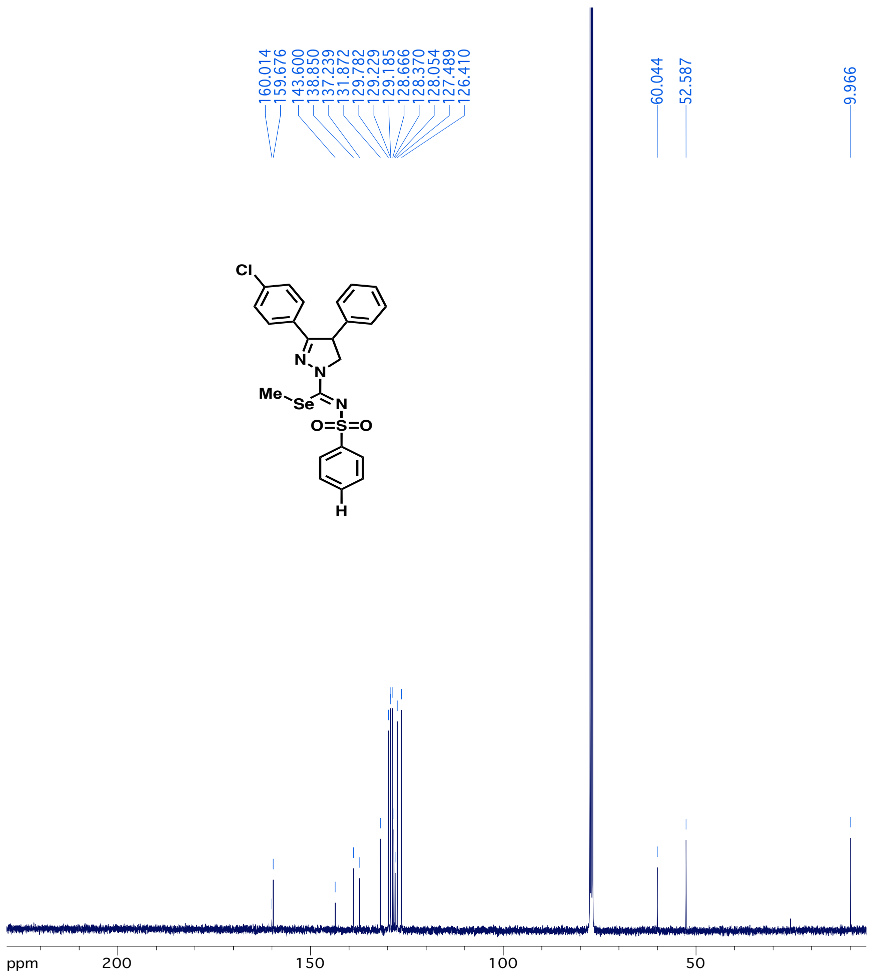

^{1}HNMR and ^{13}CNMR of **4q**

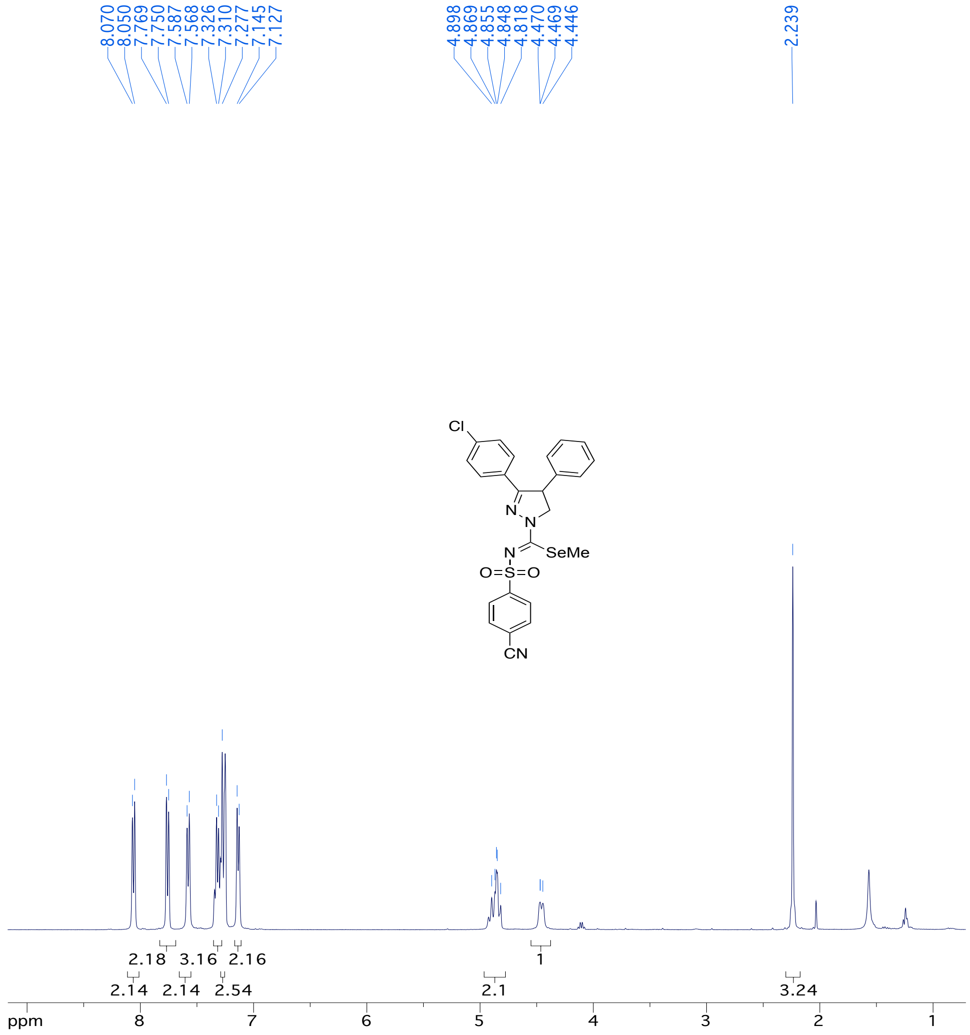

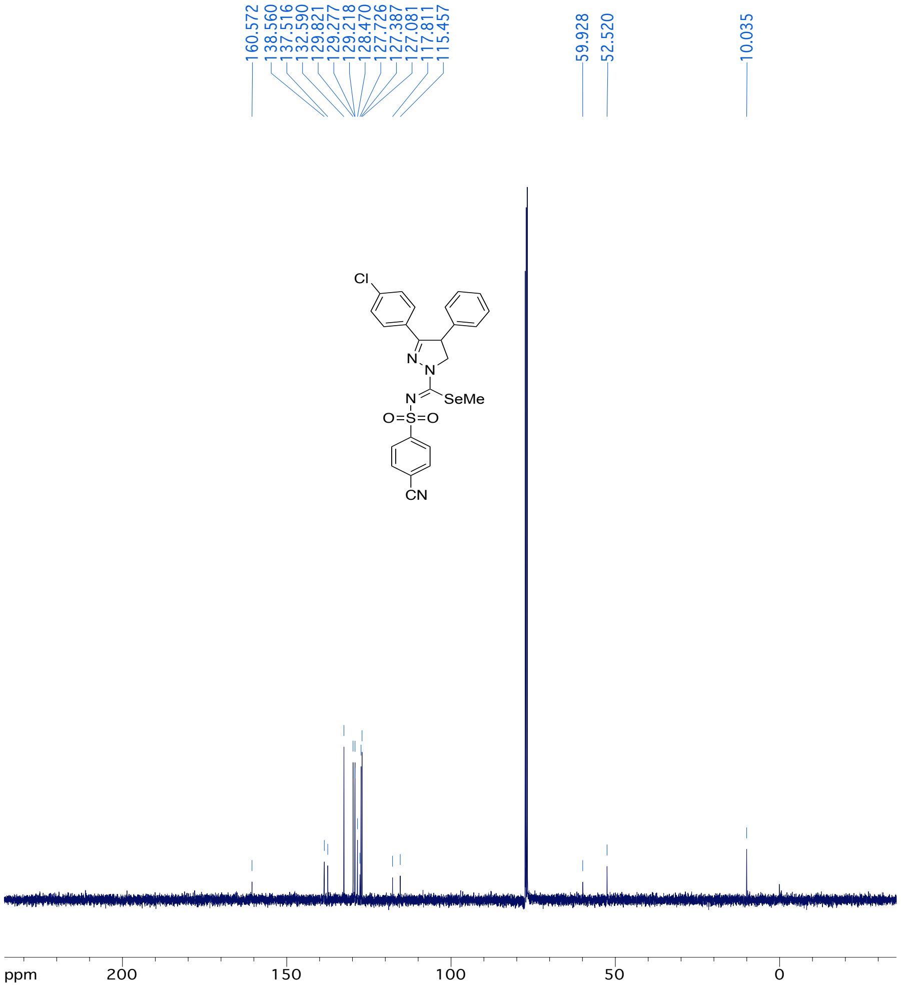

^{1}HNMR and ^{13}CNMR of **4r**

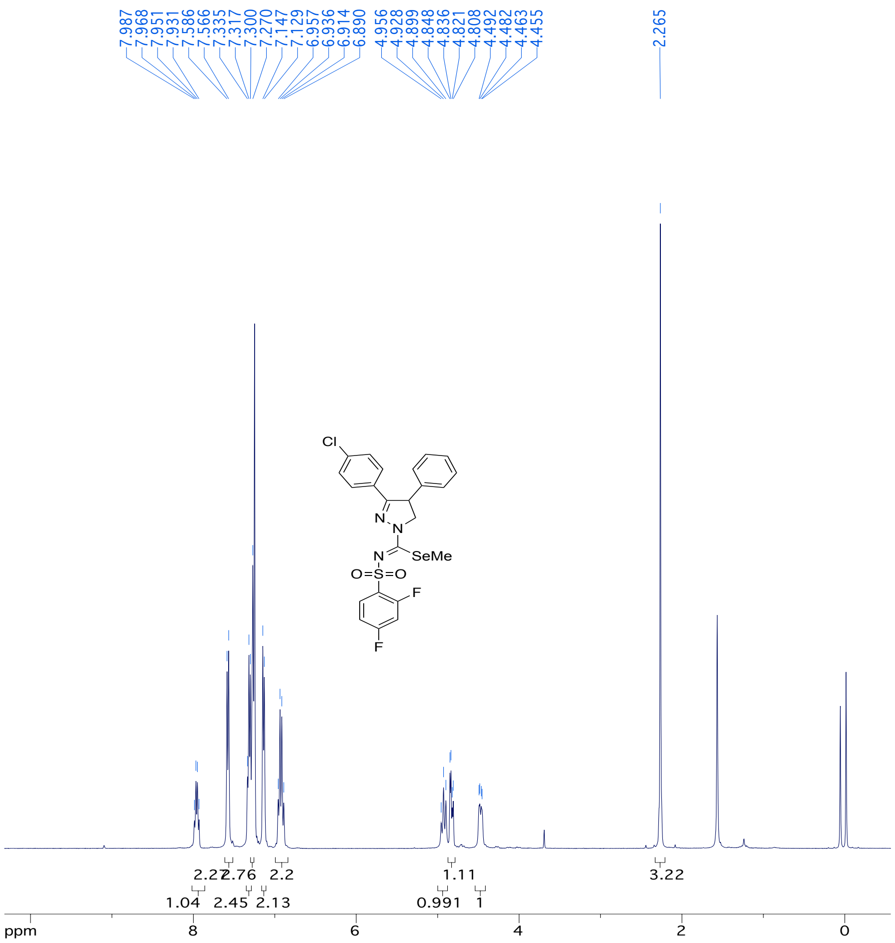

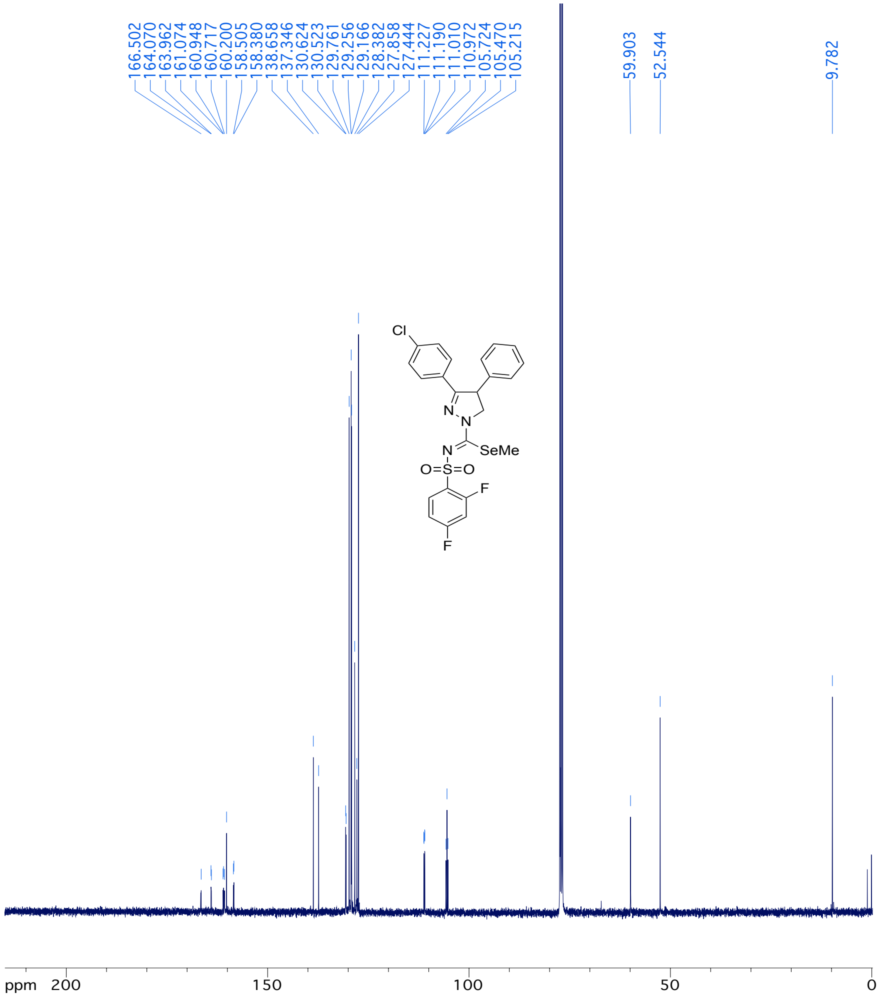

^{1}HNMR and ^{13}CNMR of **4s**

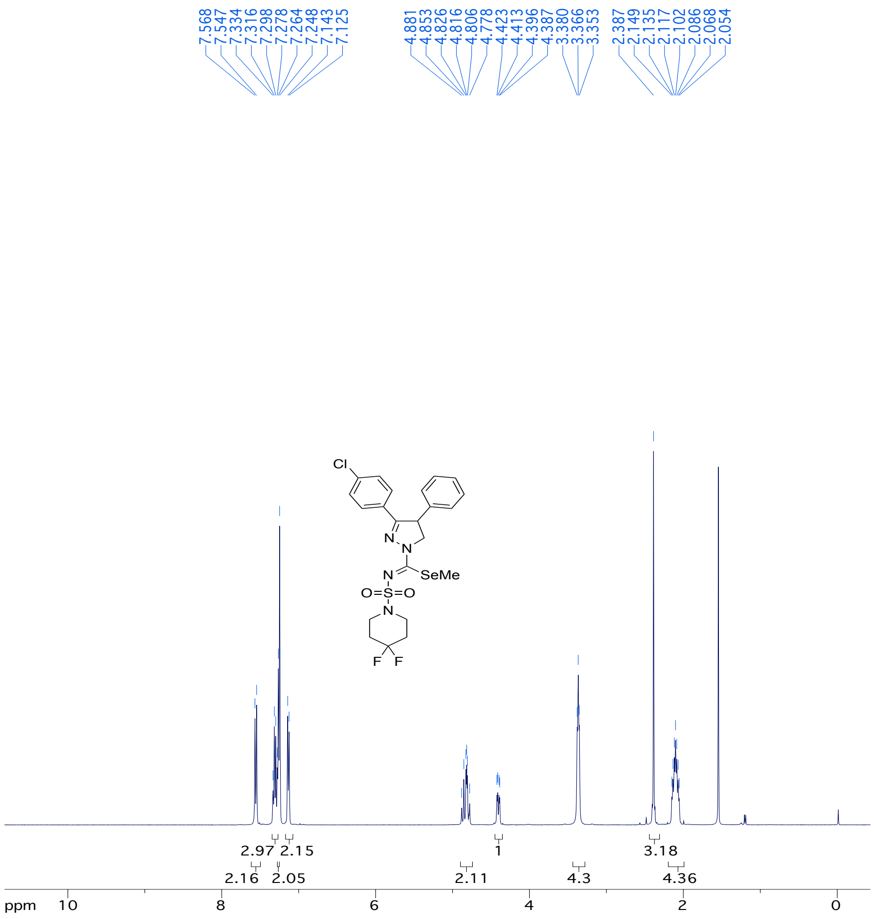

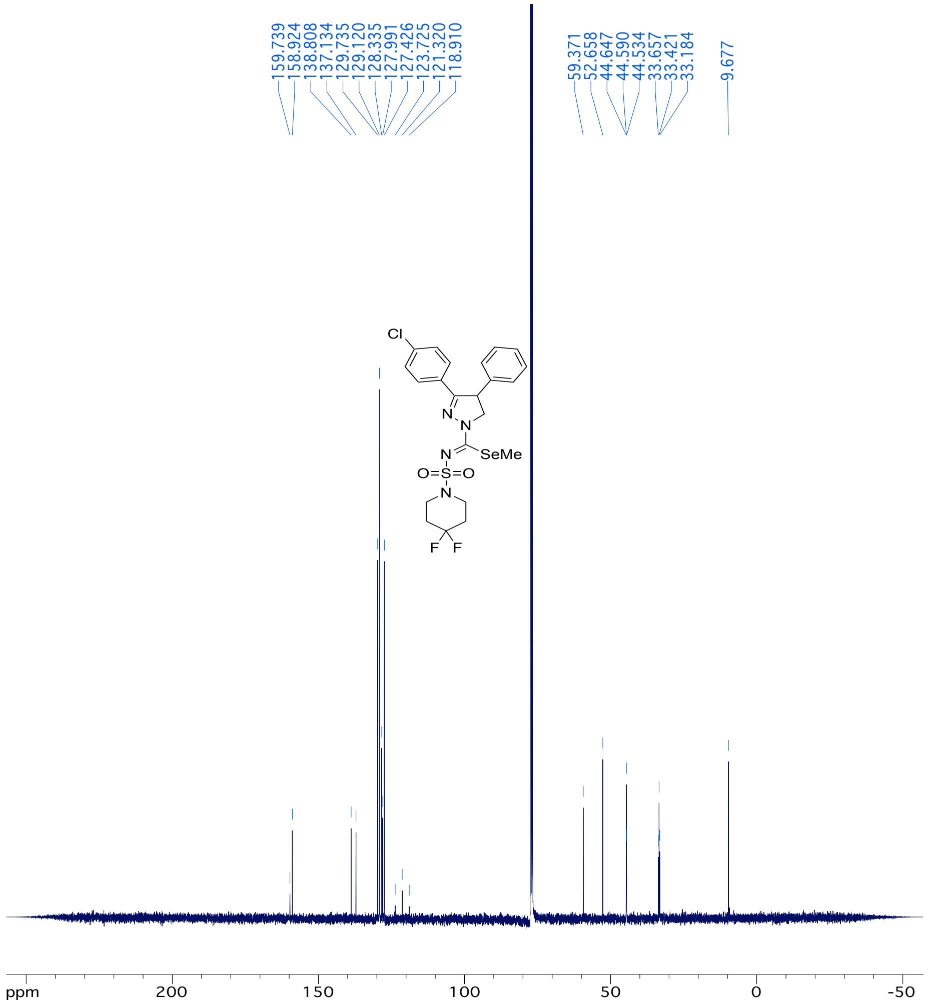

^{1}HNMR and ^{13}CNMR of **4t**

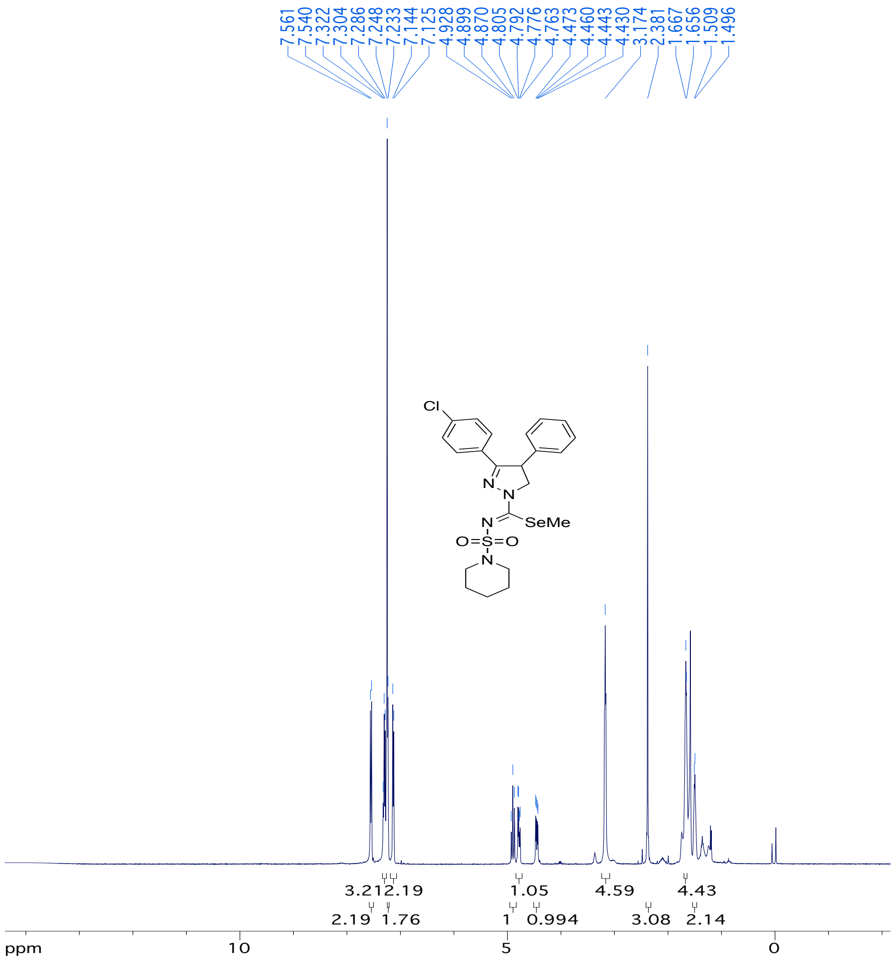

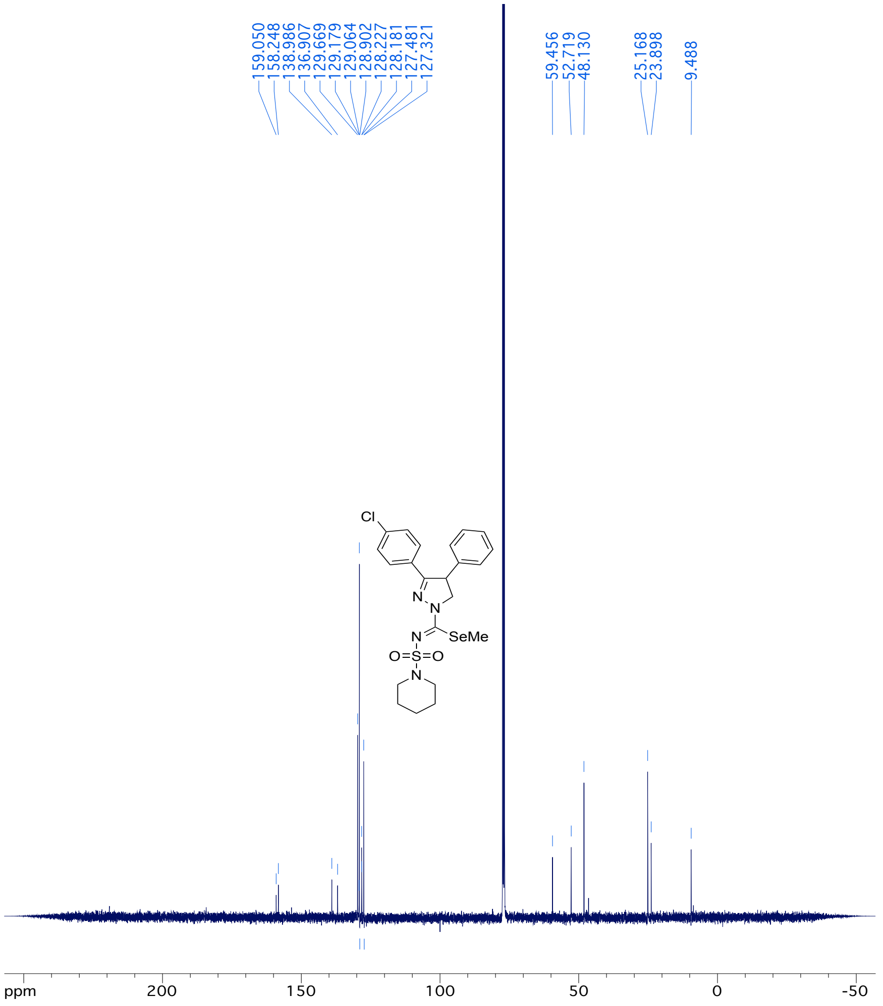

^{1}HNMR and ^{13}CNMR of **4u**

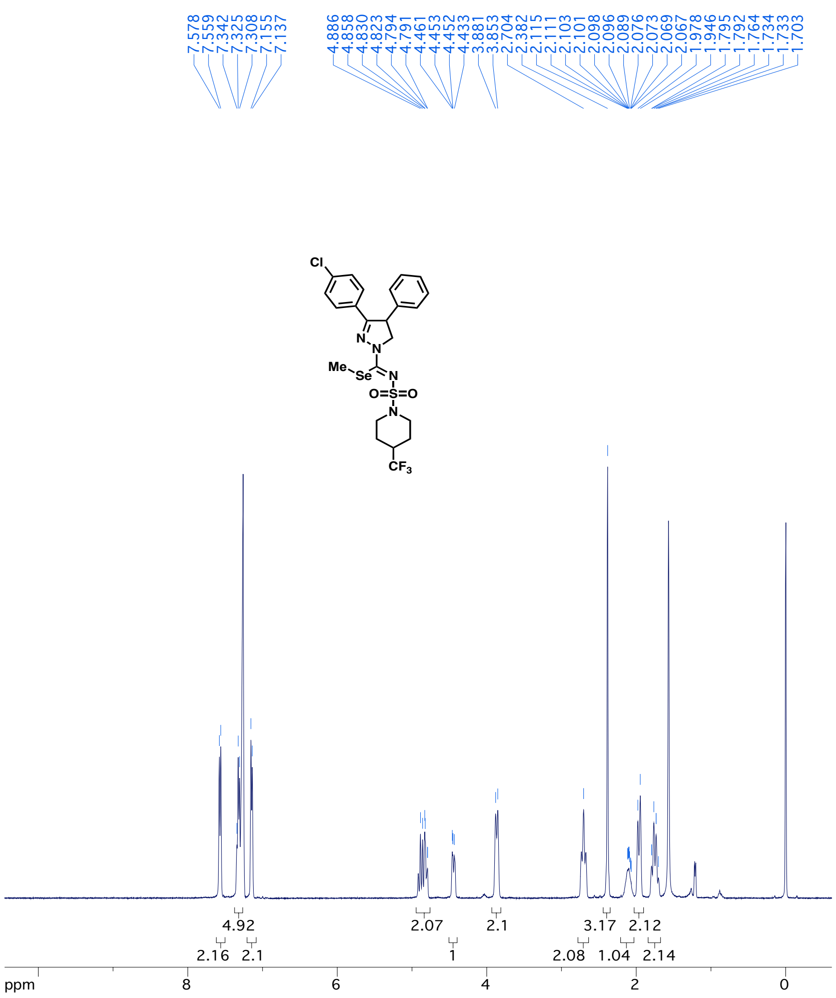

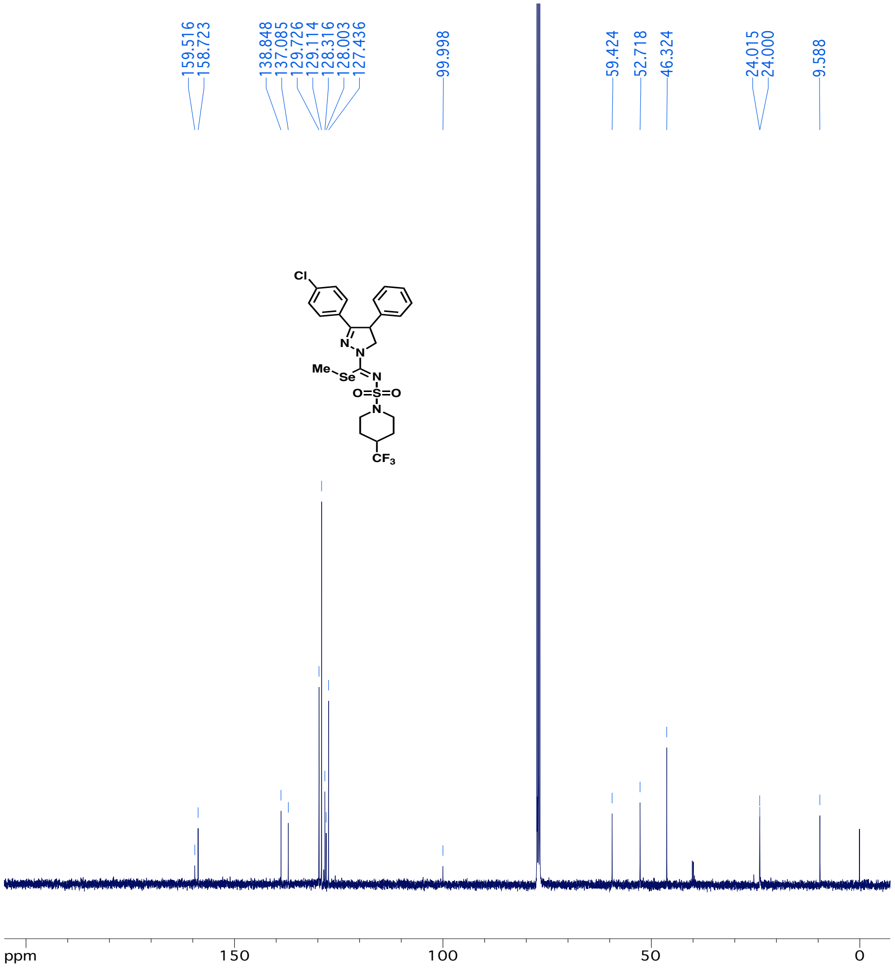

^{1}HNMR and ^{13}CNMR of **4v**

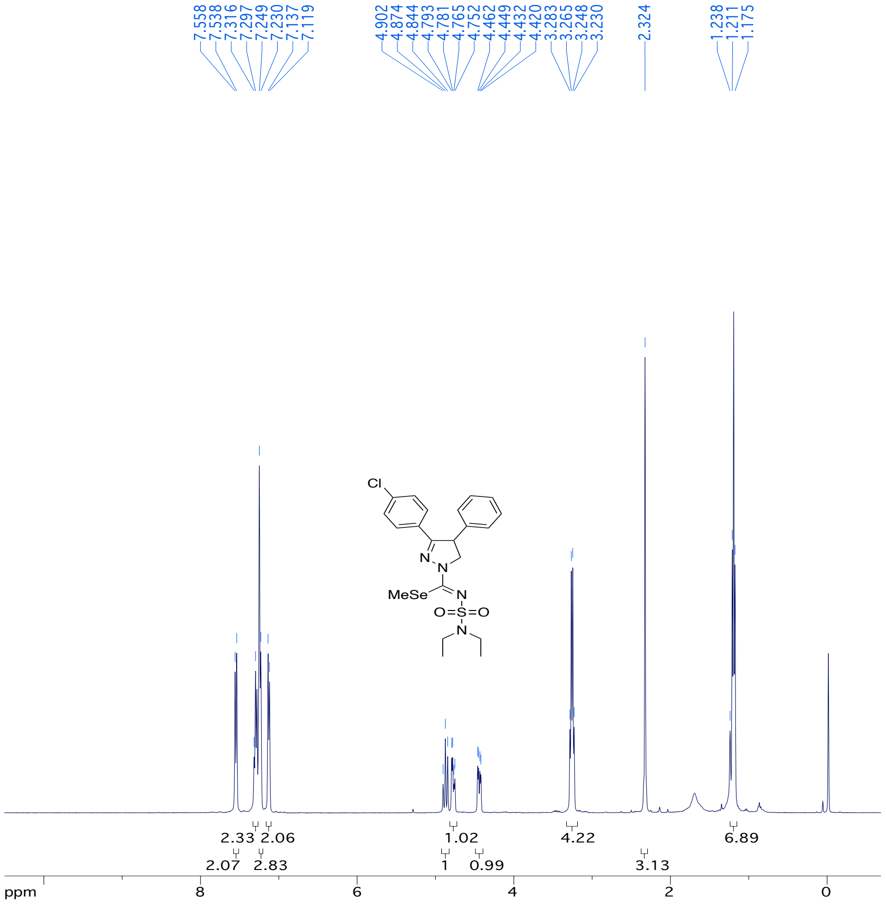

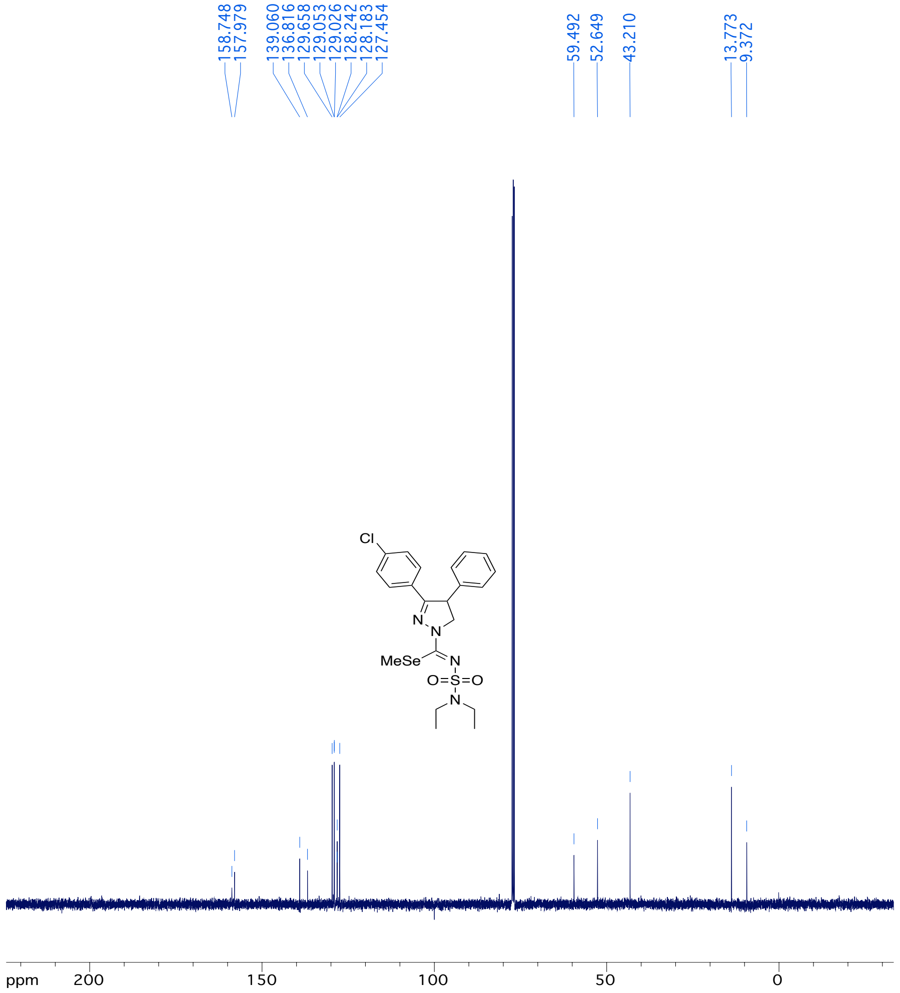

^{1}HNMR of **5**

Used immediately after synthesis

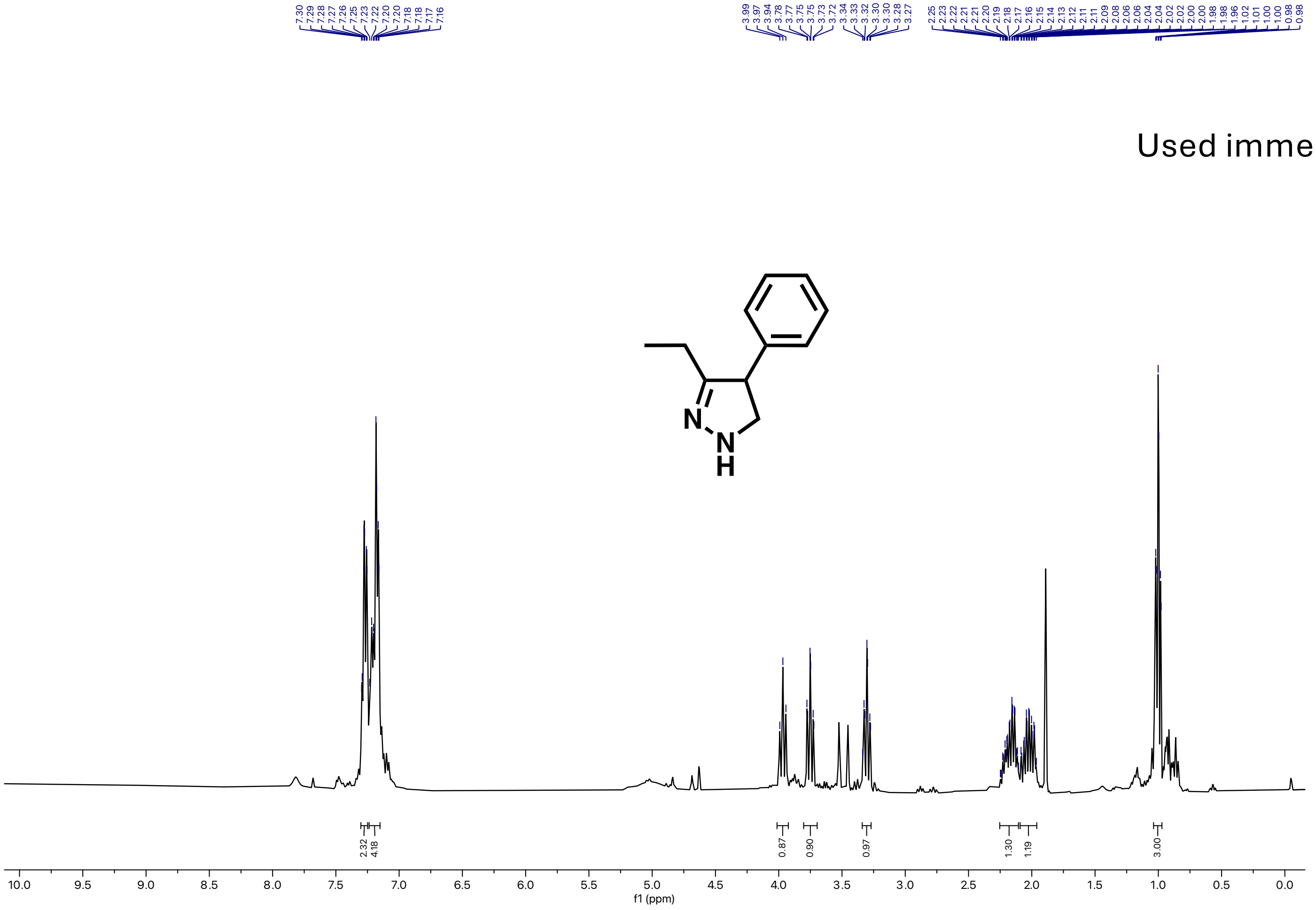

^{1}HNMR and ^{13}CNMR of **6a**

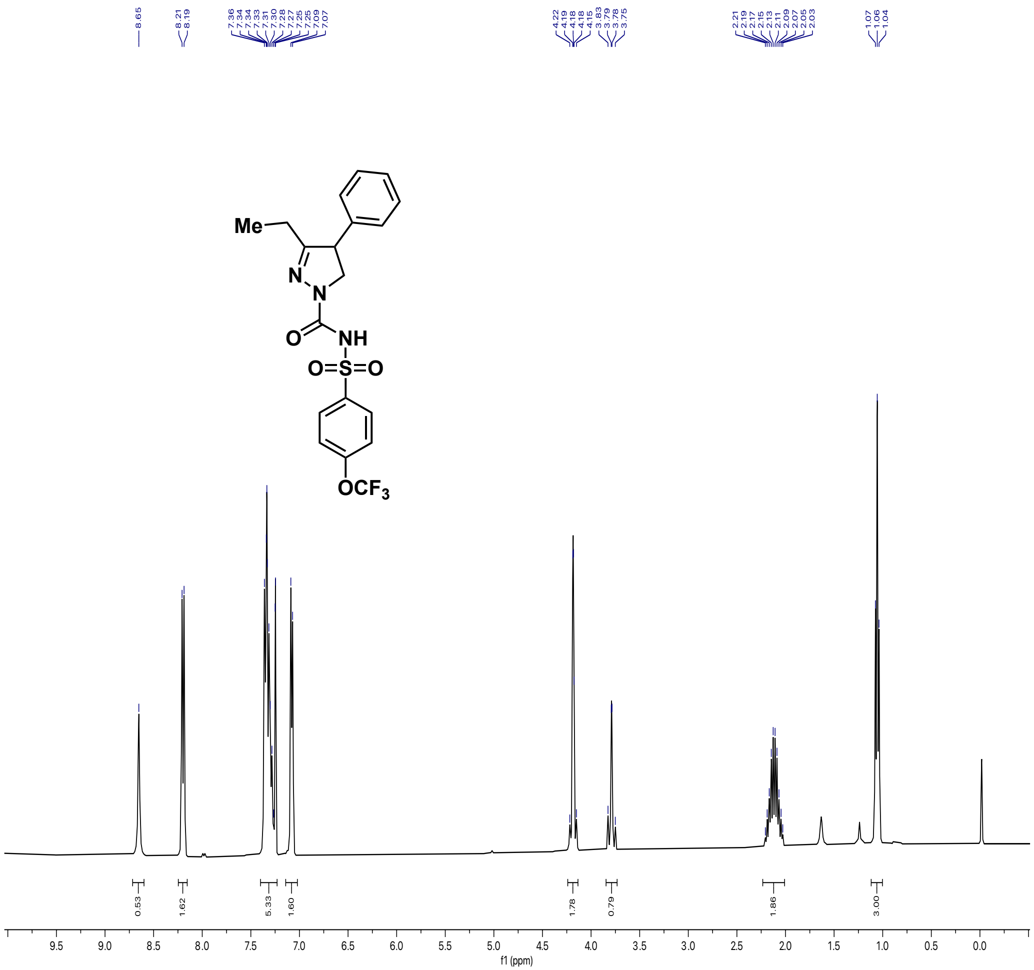

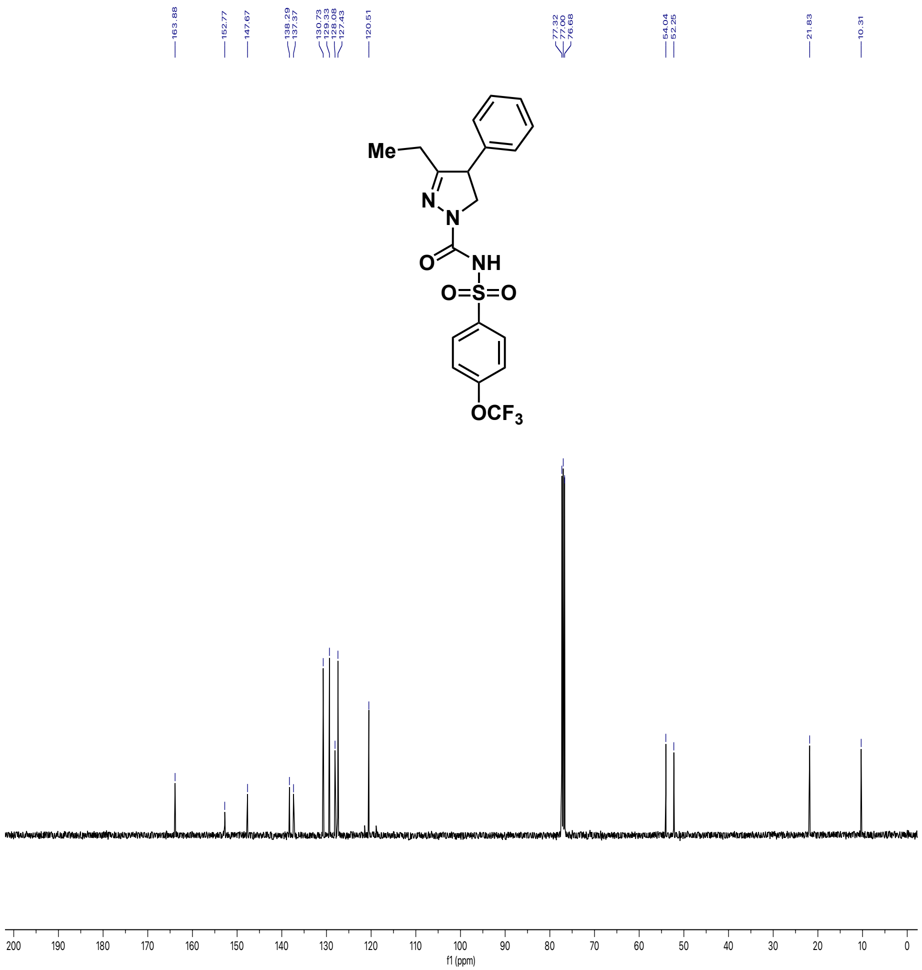

^{1}HNMR and ^{13}CNMR of **6b**

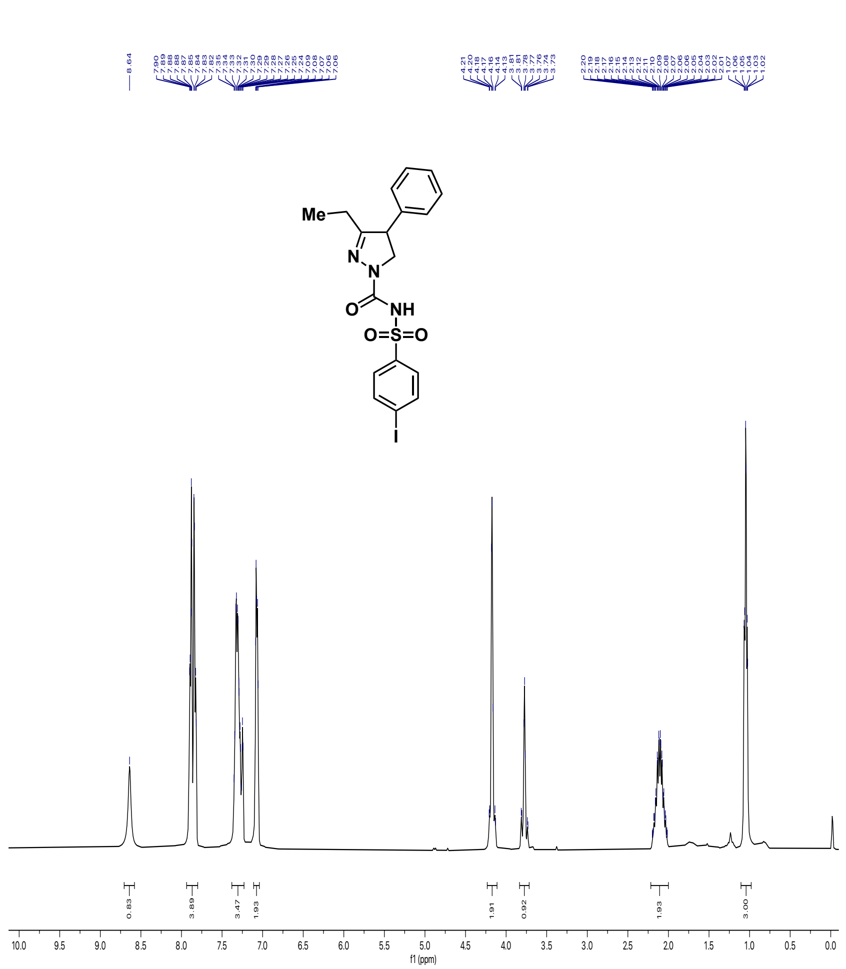

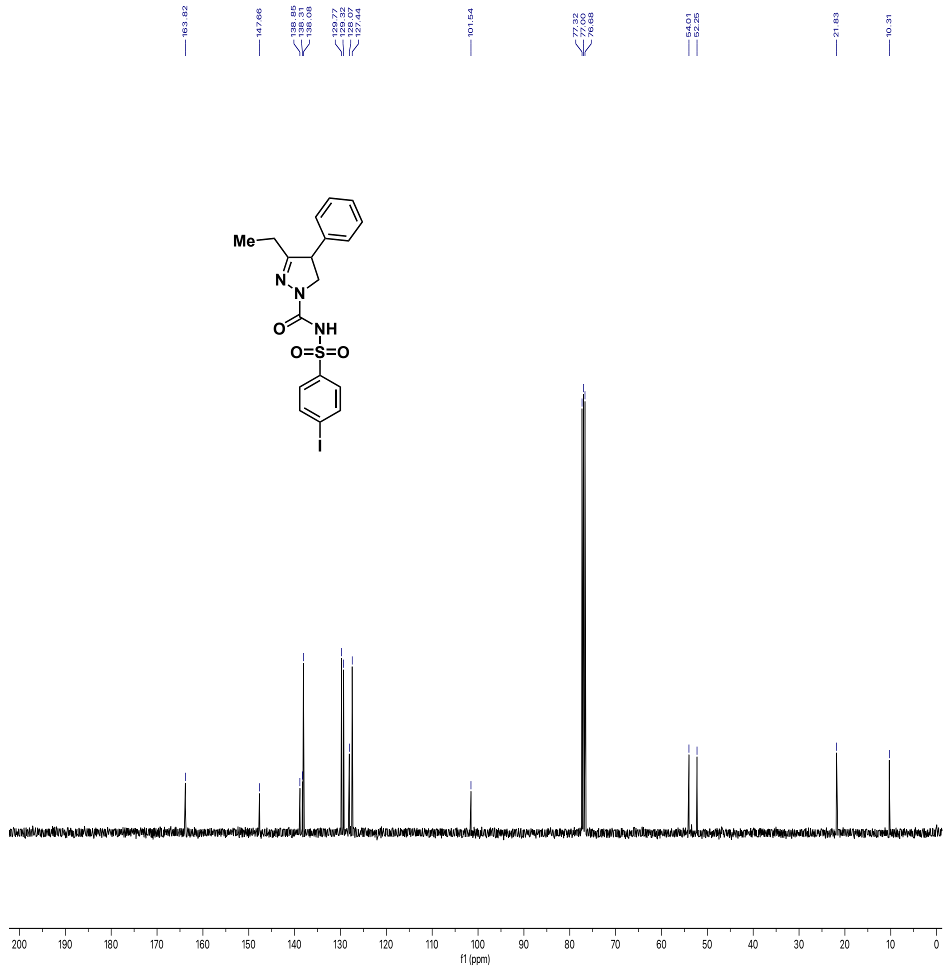

^{1}HNMR and ^{13}CNMR of **6c**

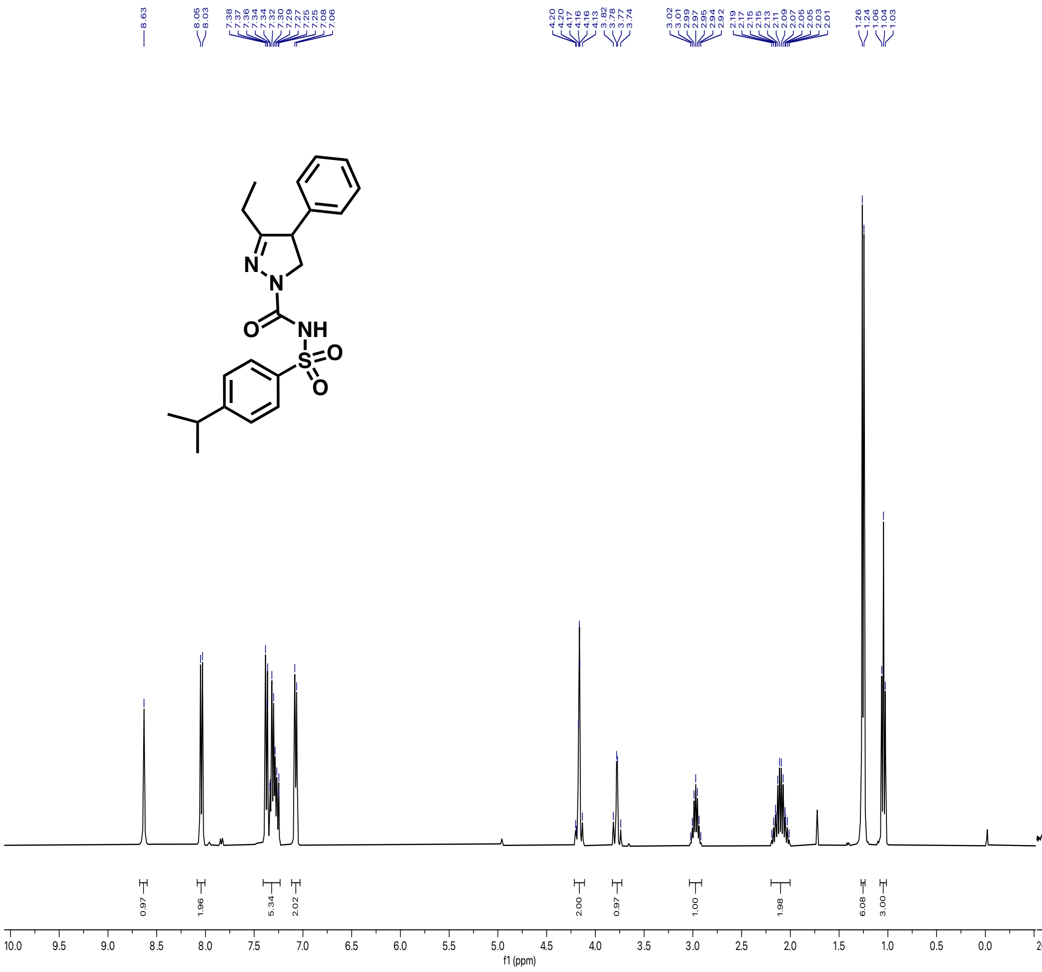

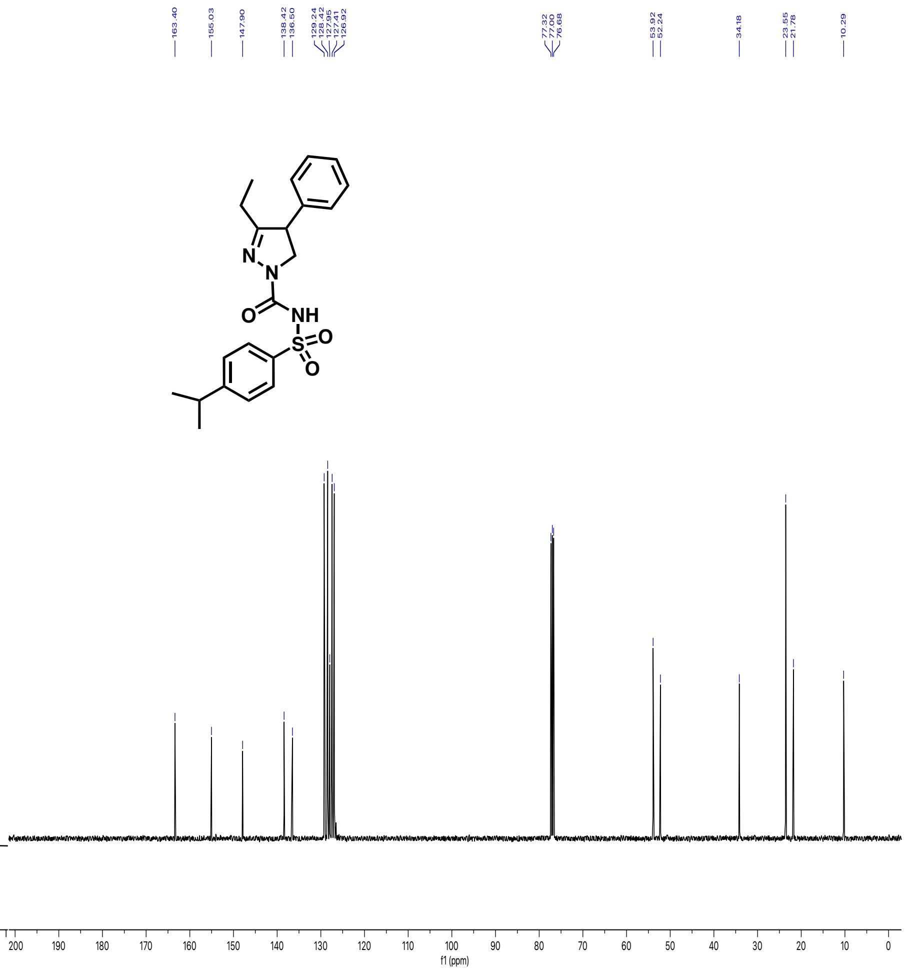

^{1}HNMR and ^{13}CNMR of **6d**

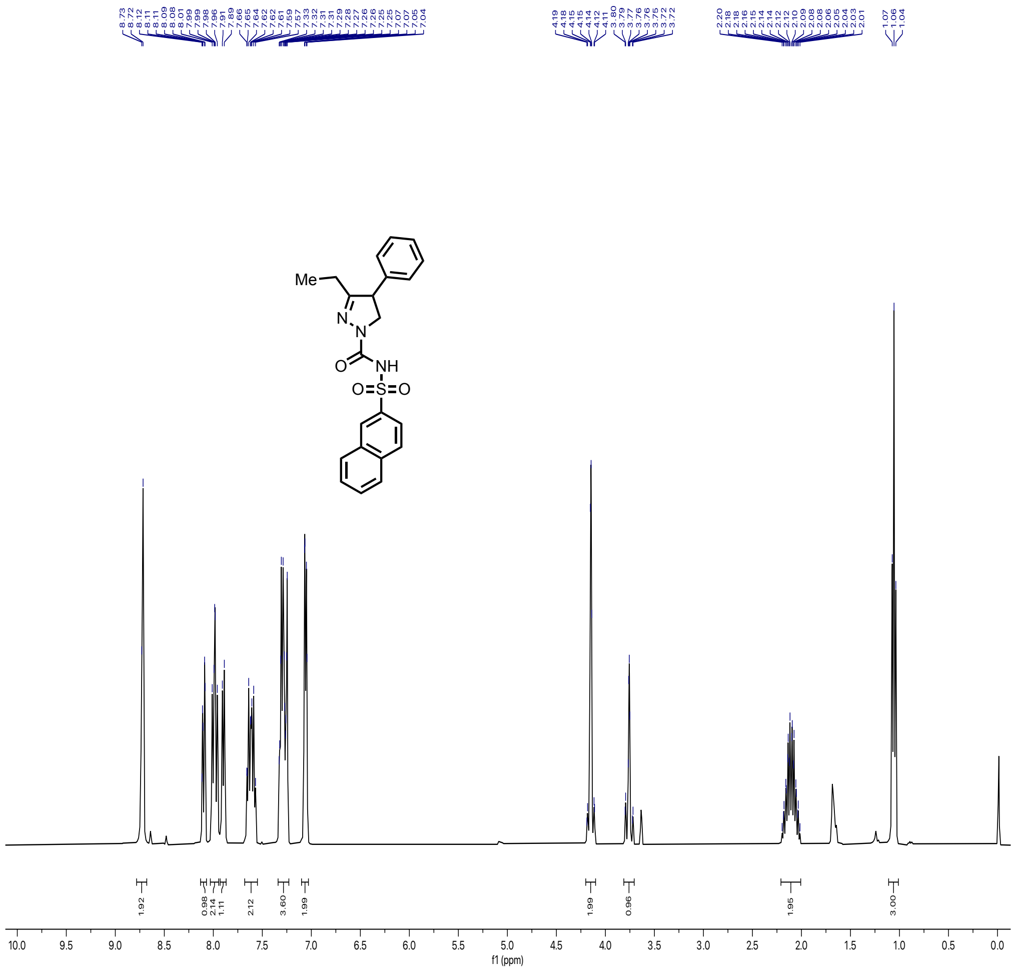

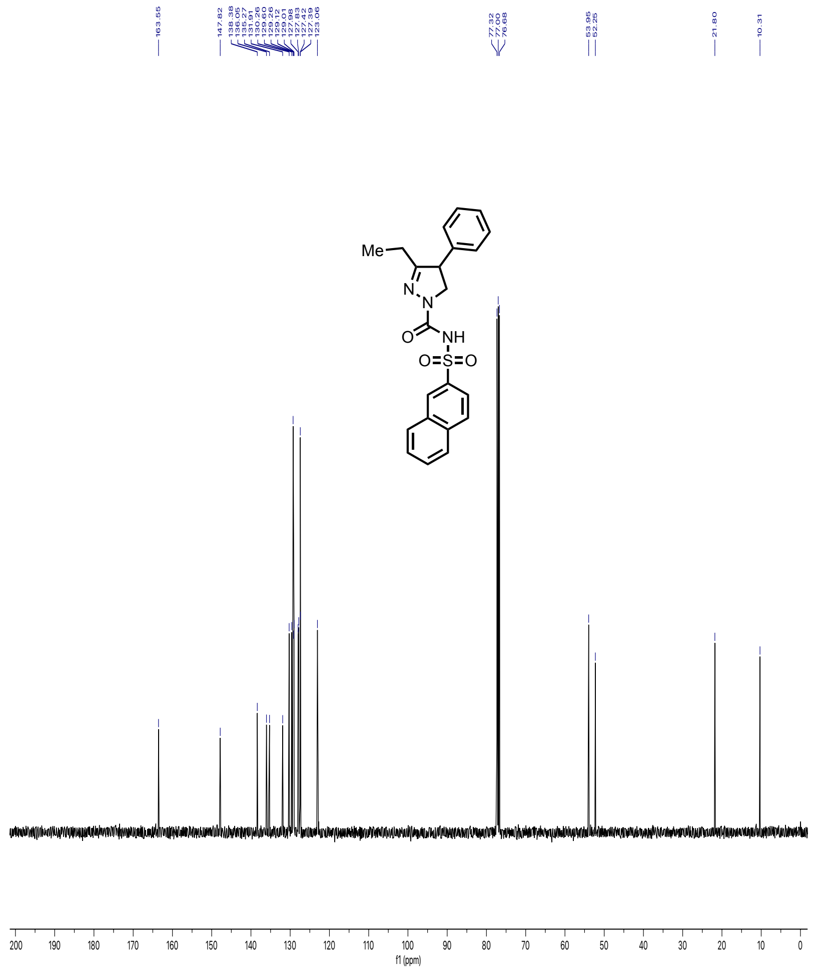

^{1}HNMR and ^{13}CNMR of **7a**

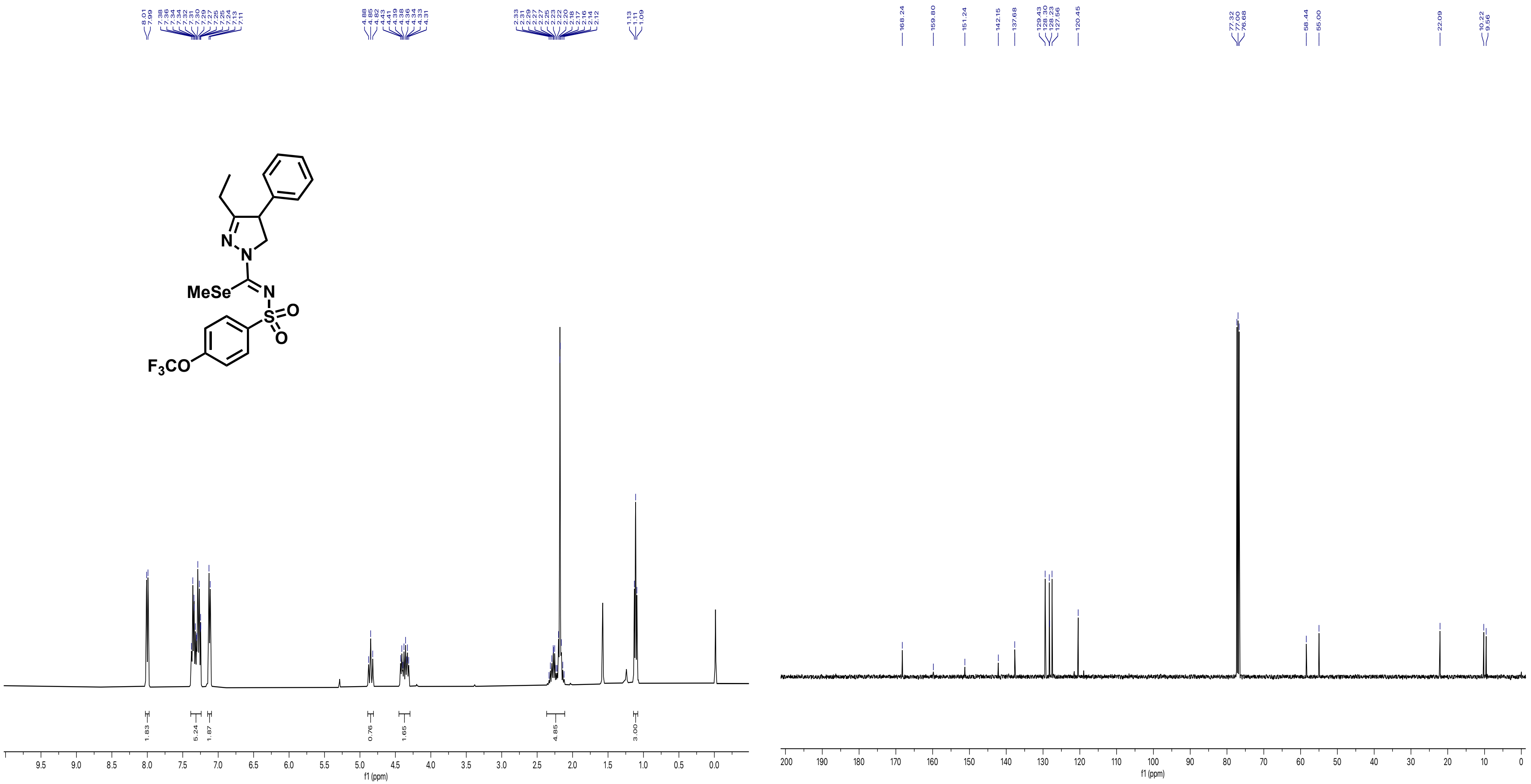

^{1}HNMR and ^{13}CNMR of **7b**

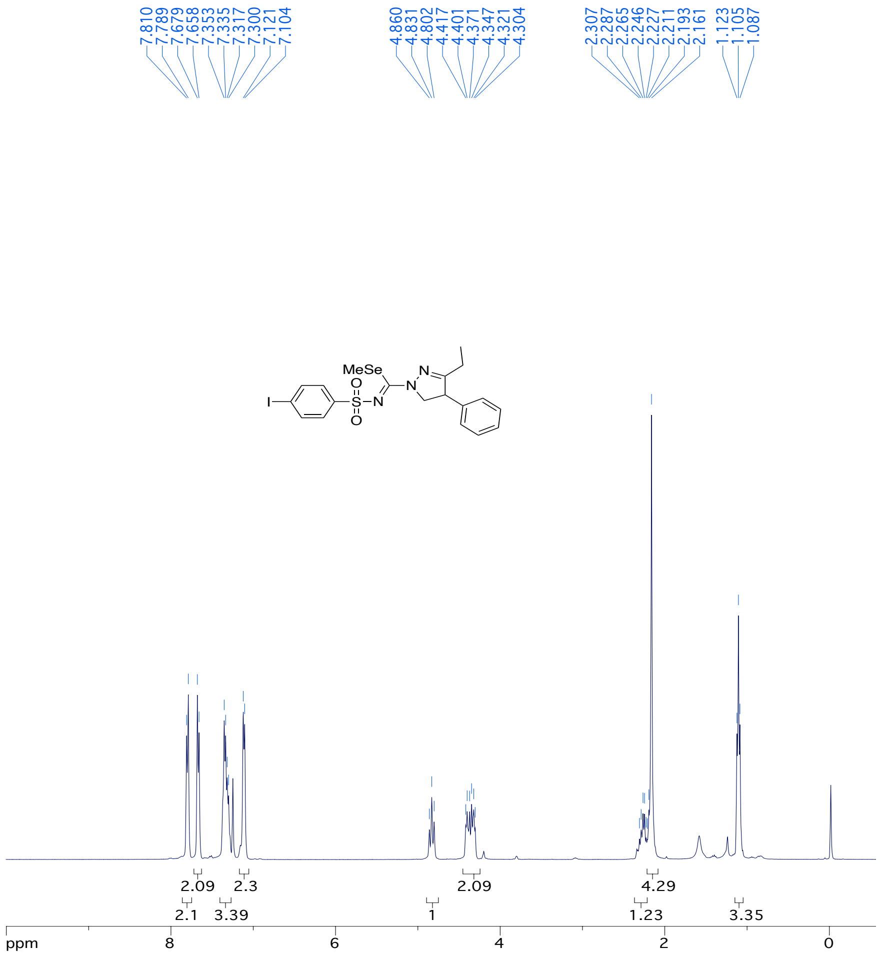

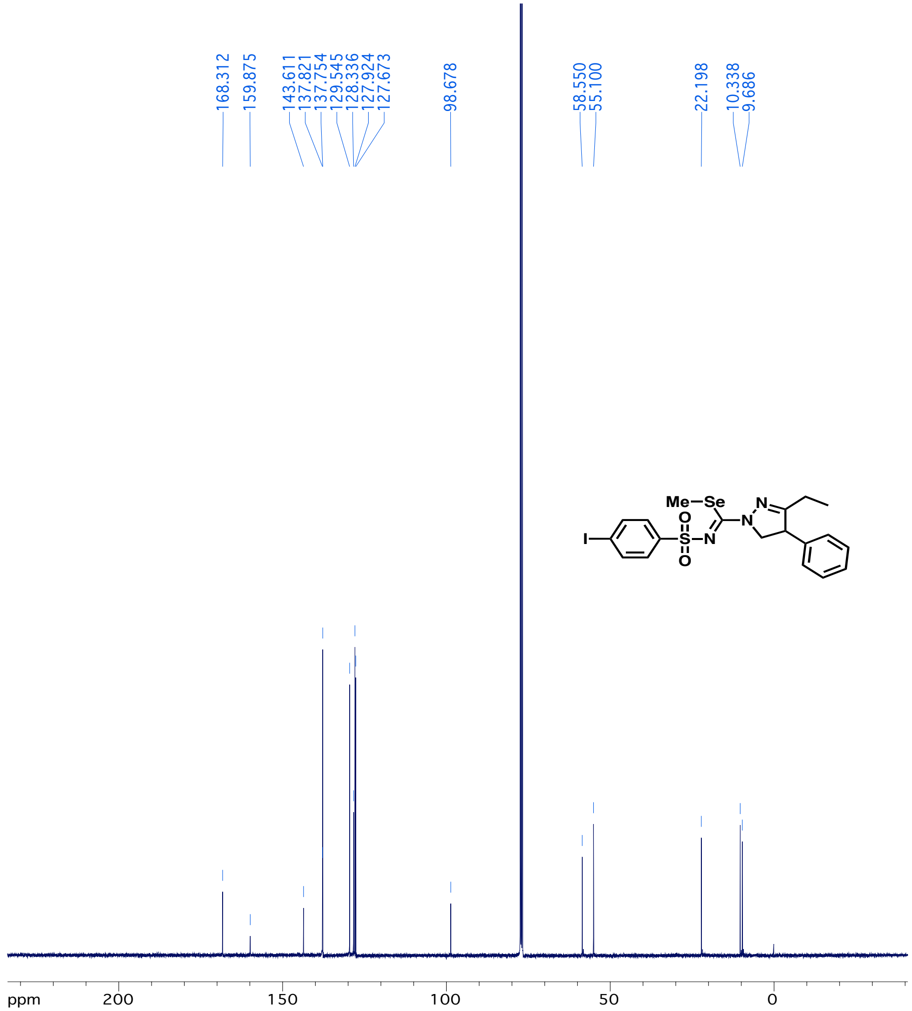

^{1}HNMR and ^{13}CNMR of **8c**

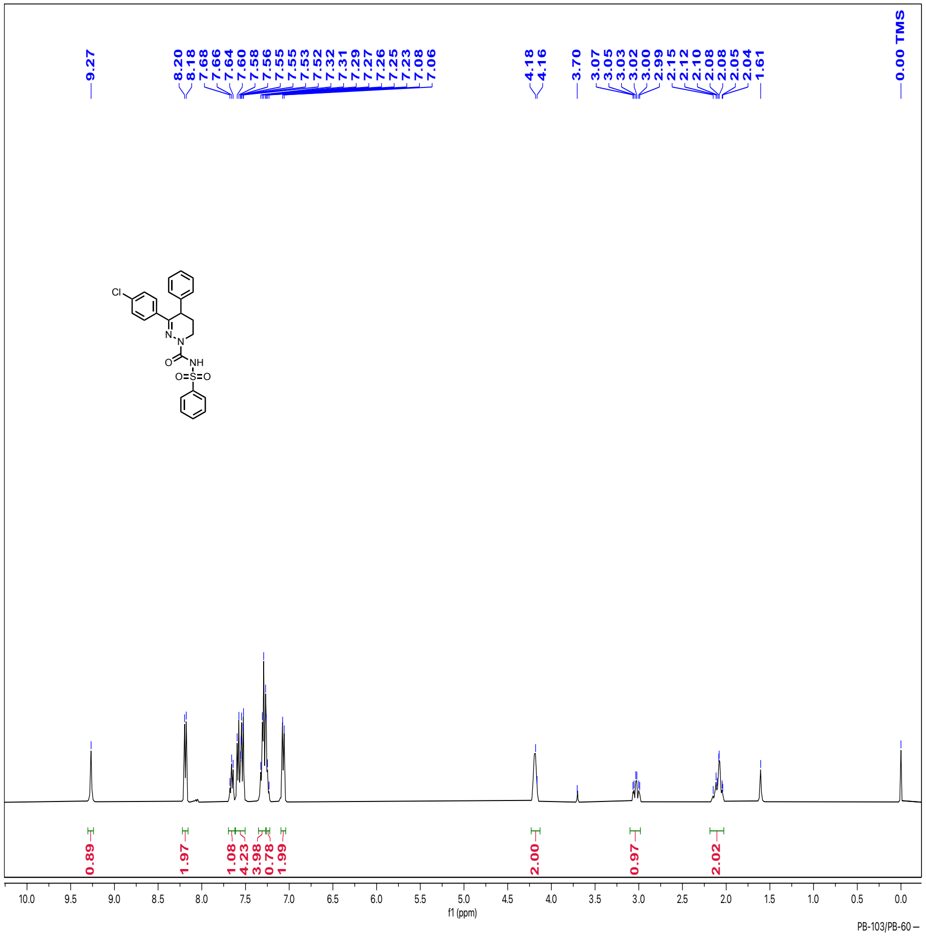

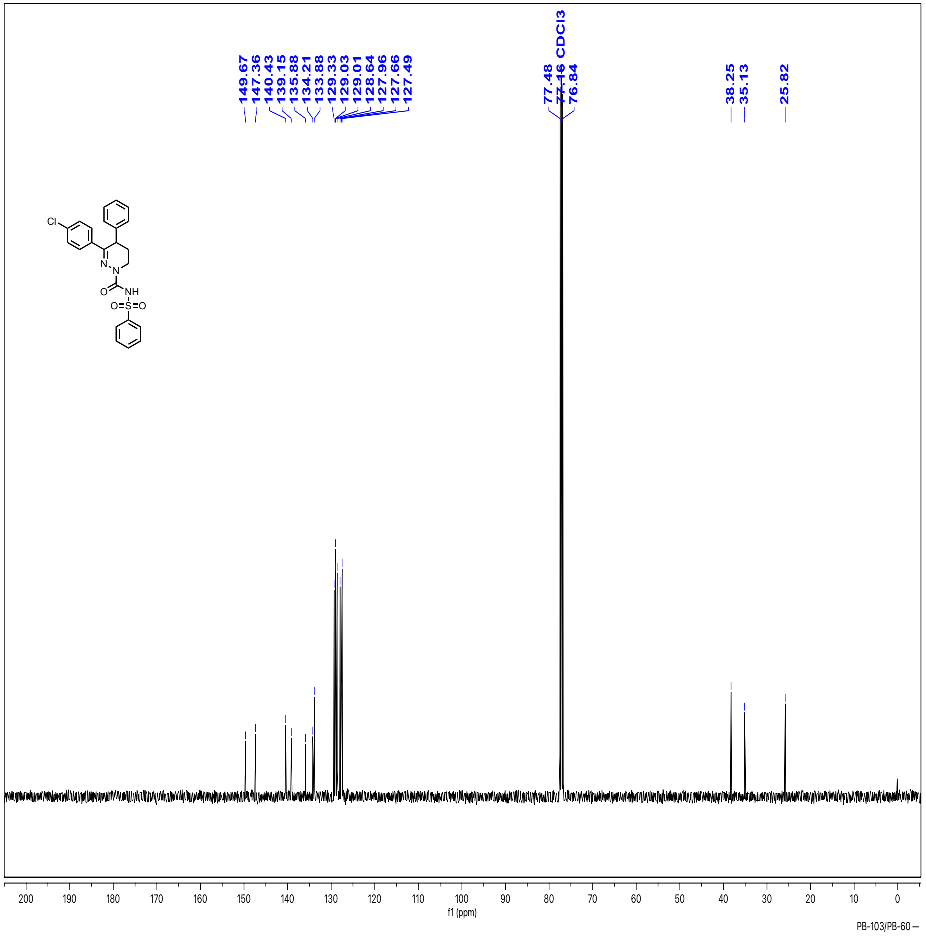

^{1}HNMR and ^{13}CNMR of **8d**

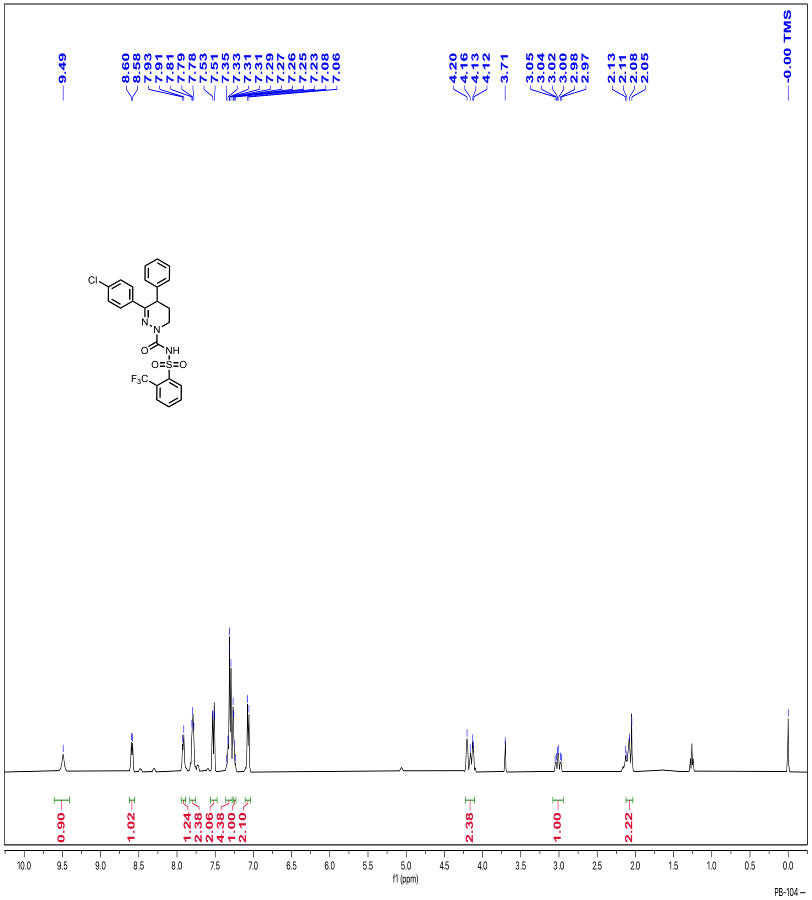

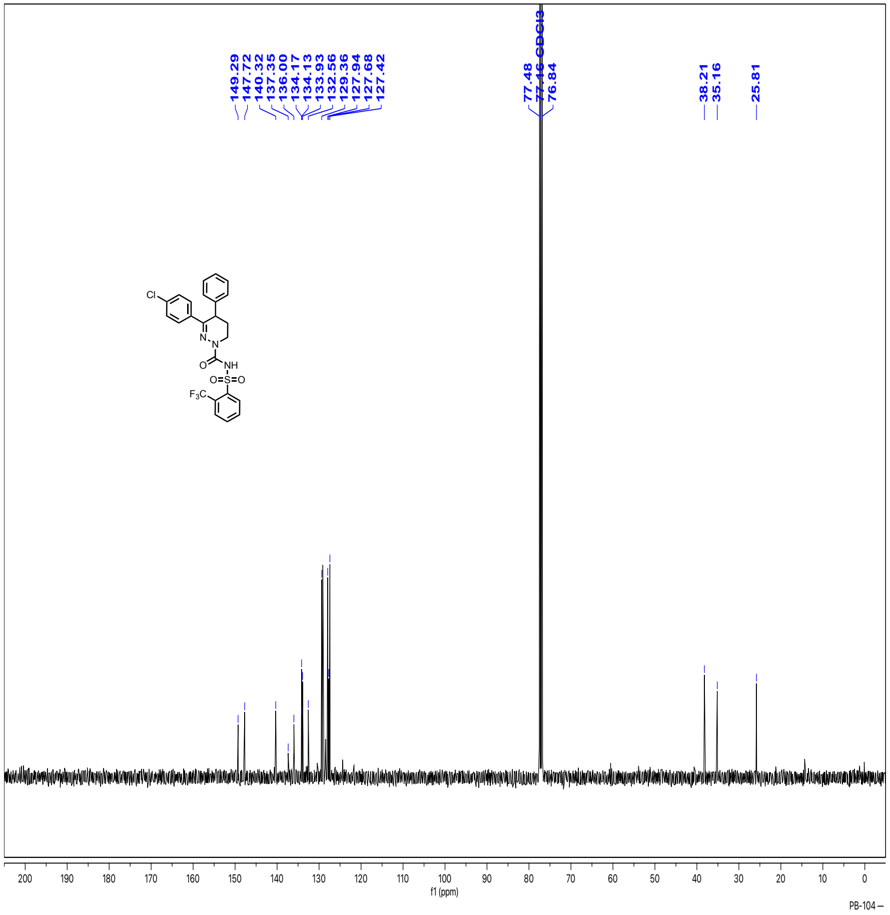

^{1}HNMR and ^{13}CNMR of **8e**

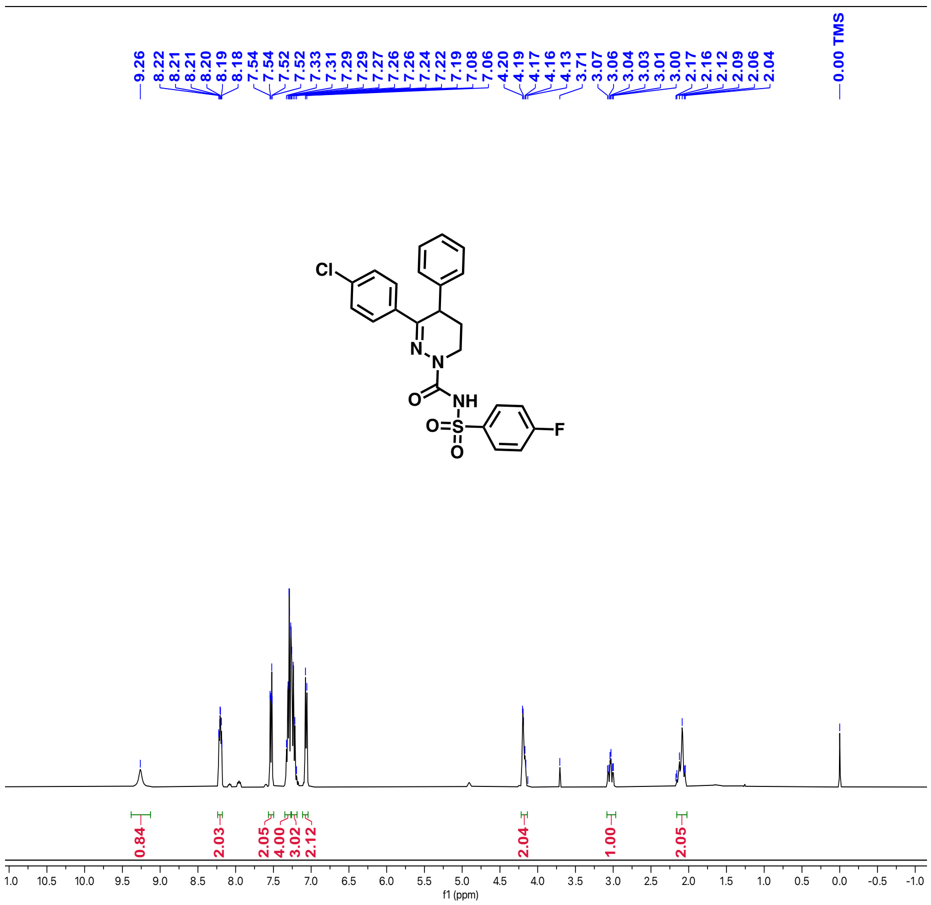

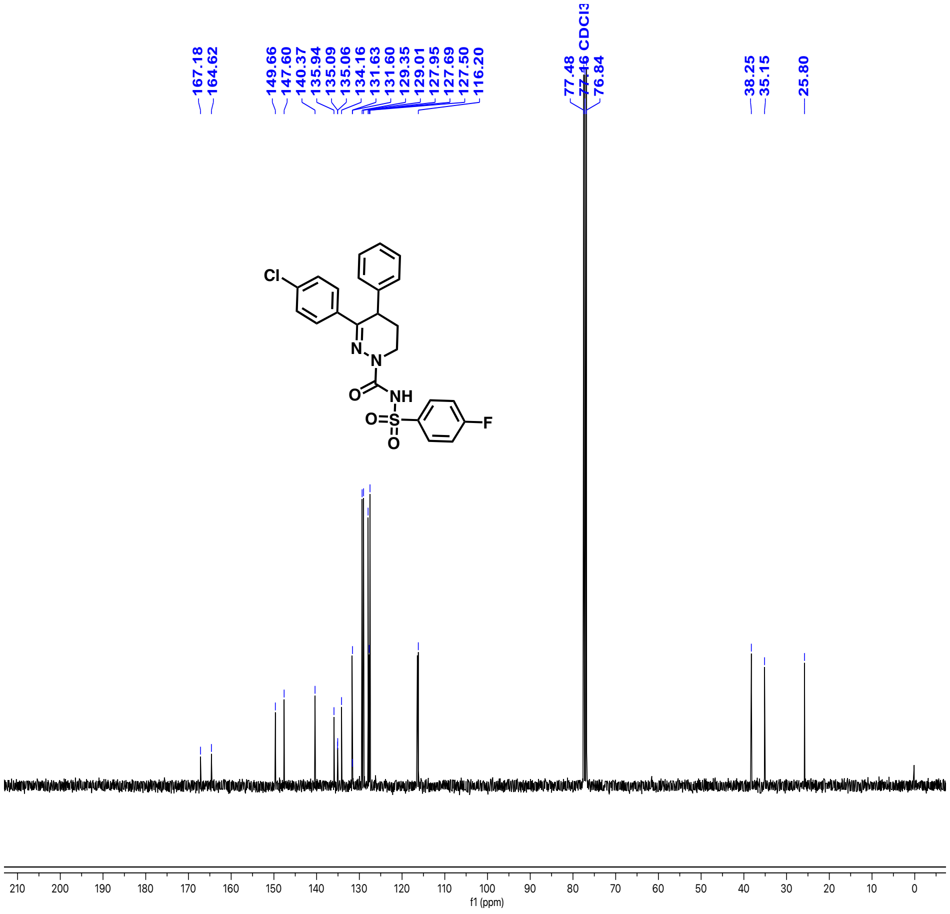

^{1}HNMR and ^{13}CNMR of **9a**

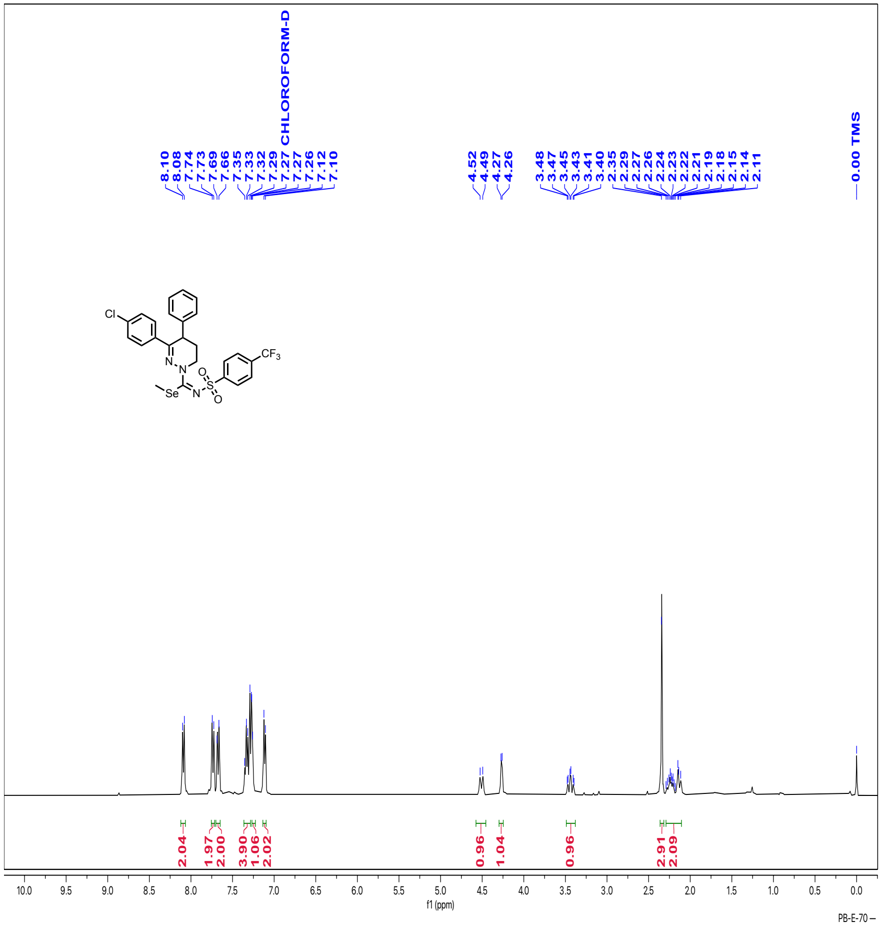

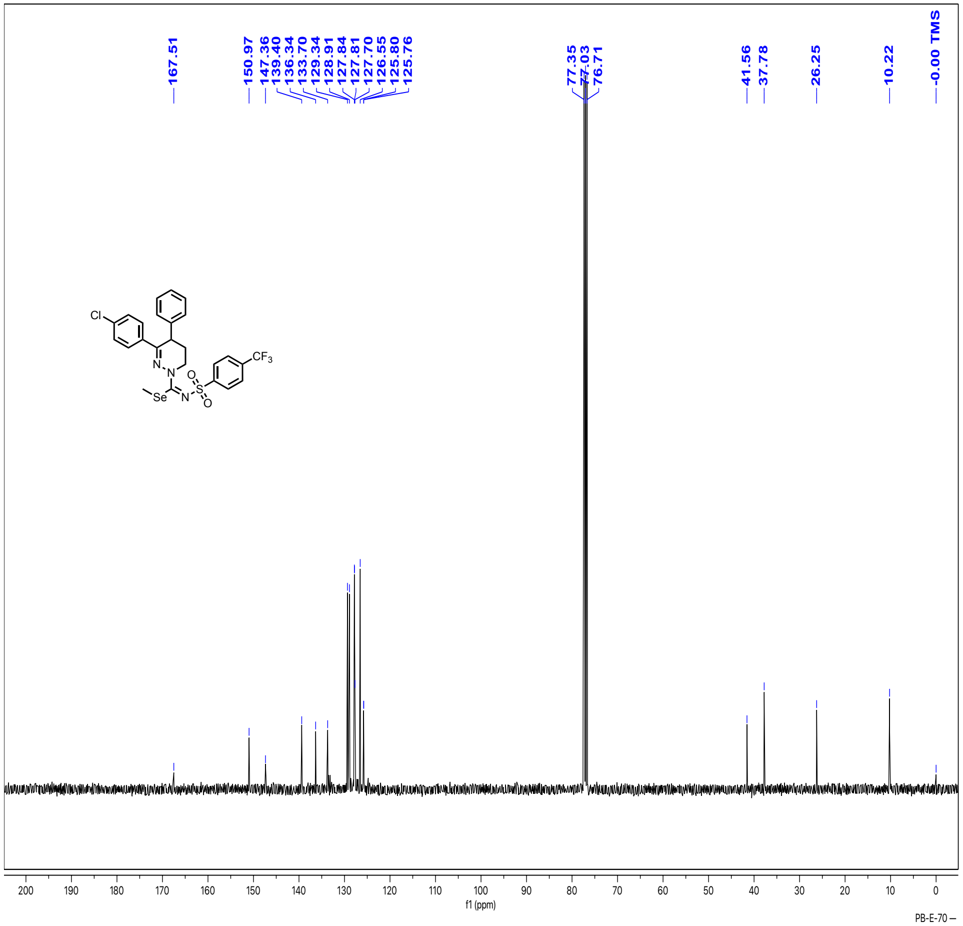

^{1}HNMR and ^{13}CNMR of **9b**

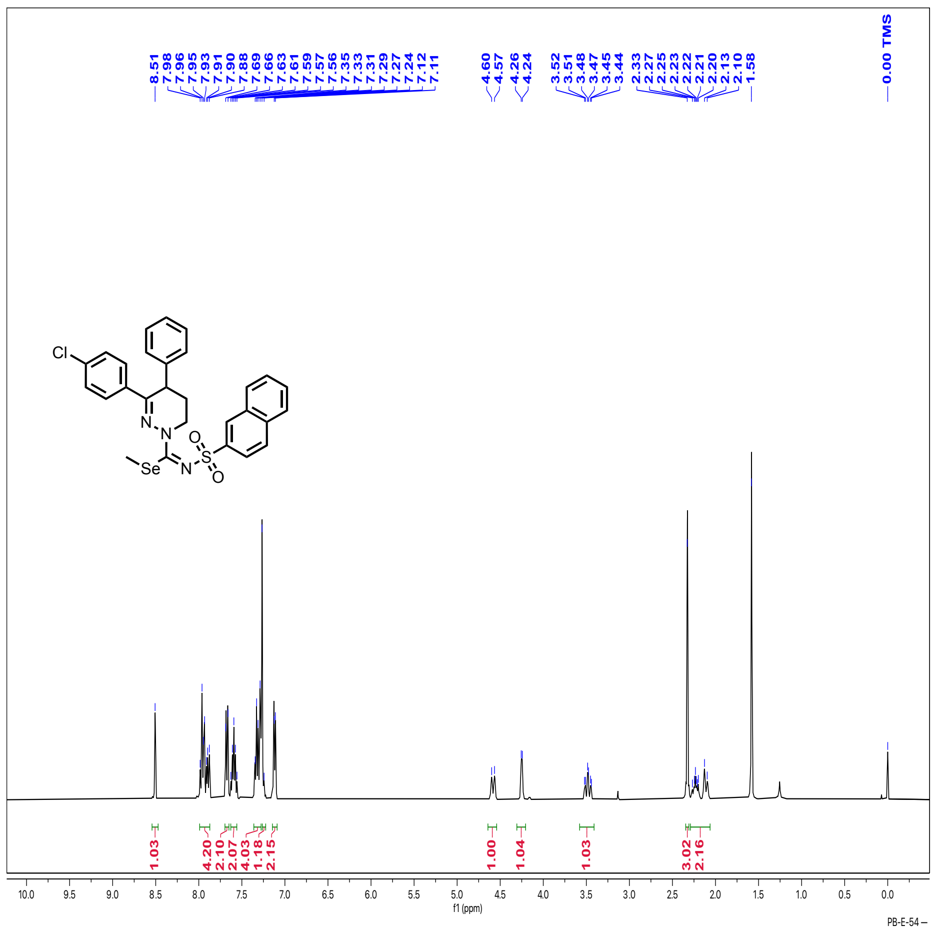

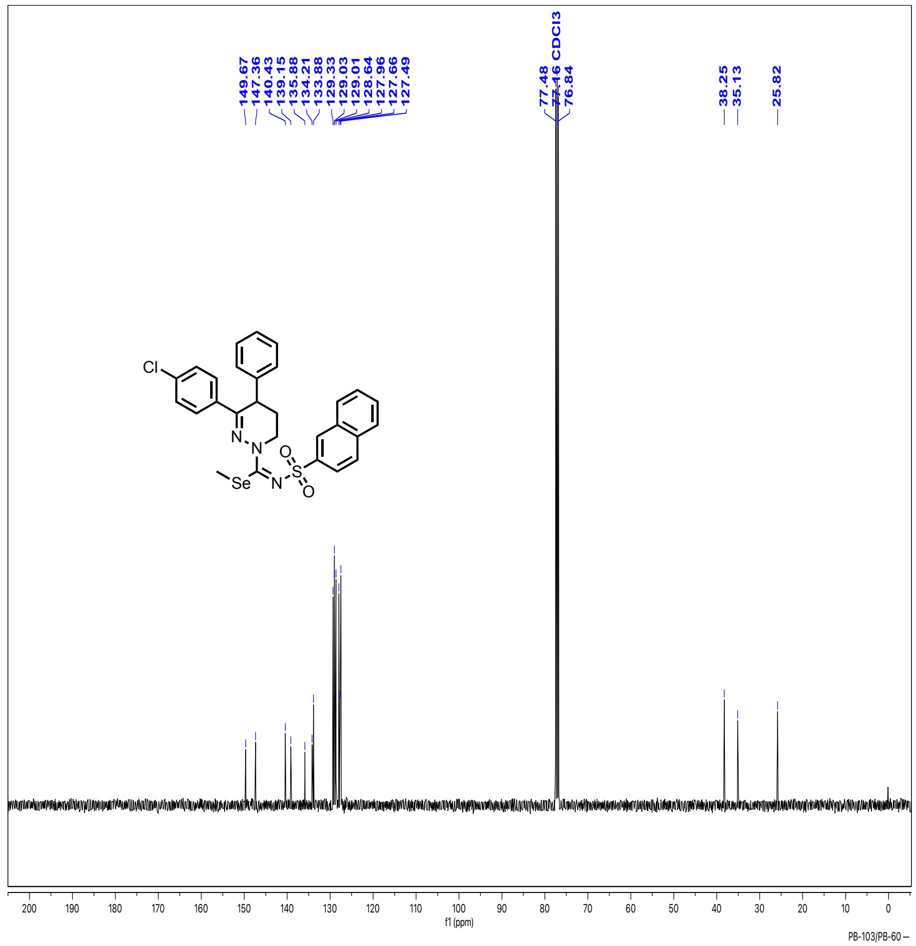

^{1}HNMR and ^{13}CNMR of **9c**

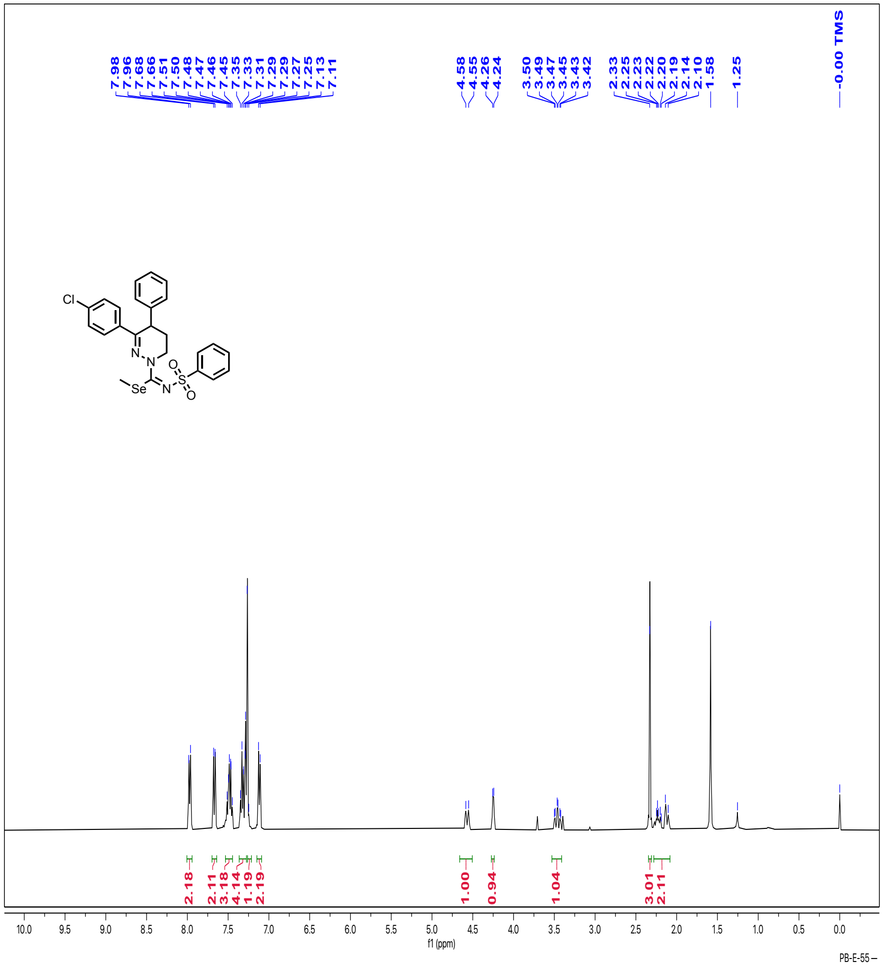

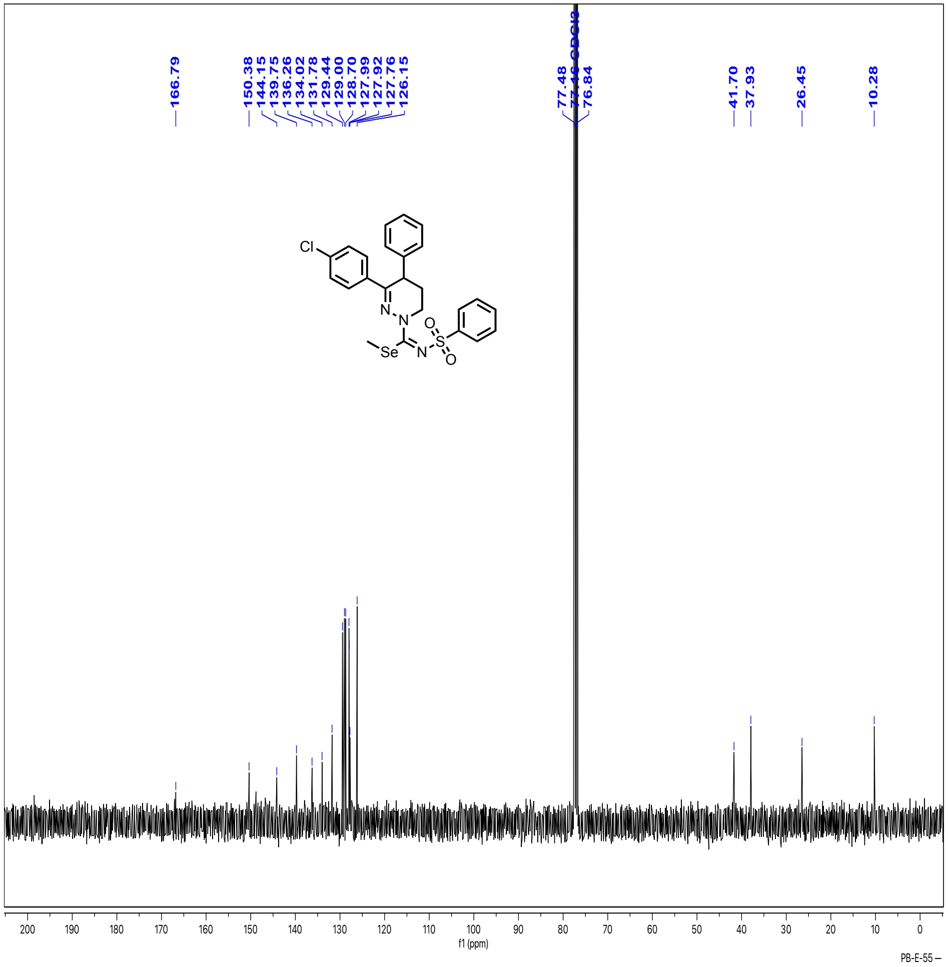

^{1}HNMR and ^{13}CNMR of **10**

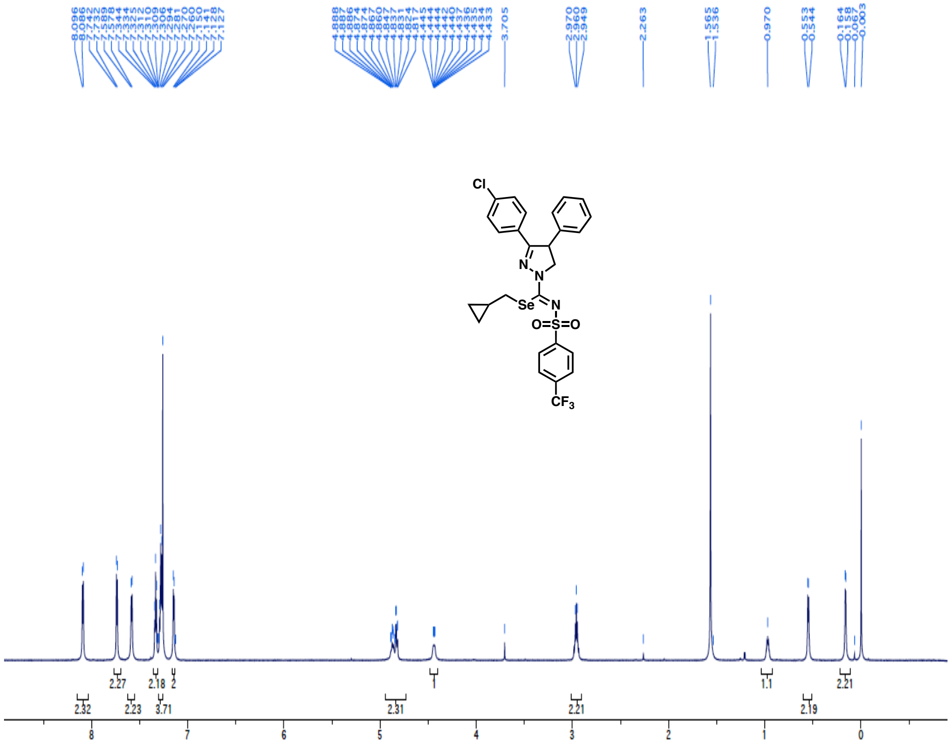

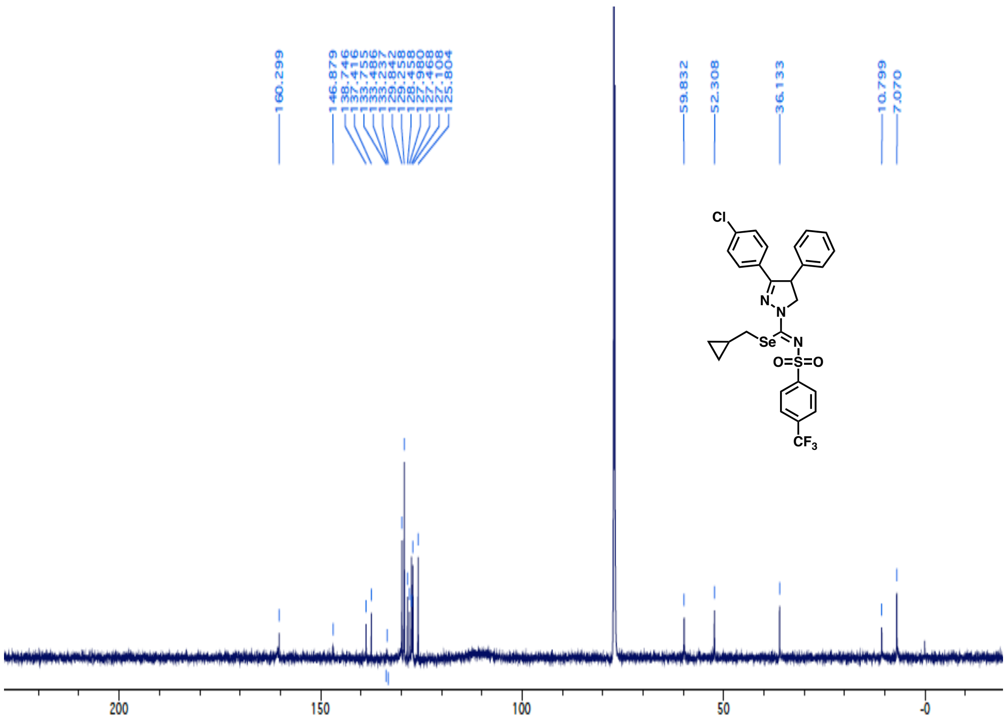

^{1}HNMR and ^{13}CNMR of **11**

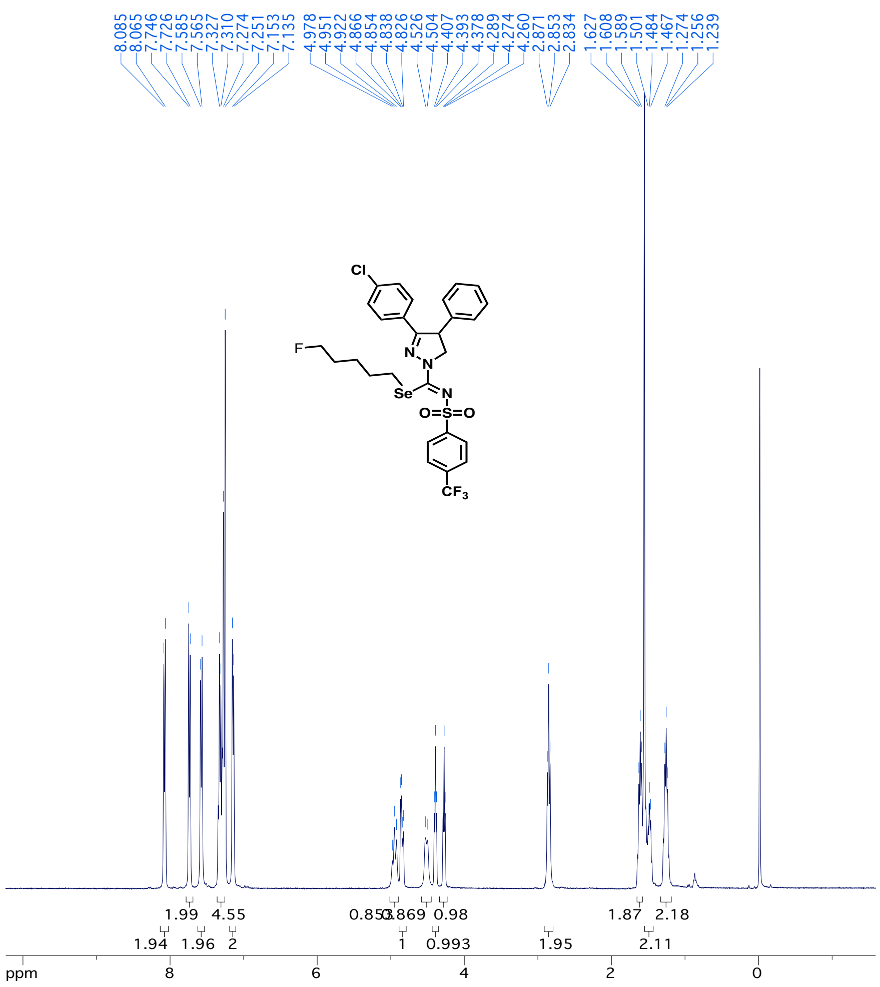

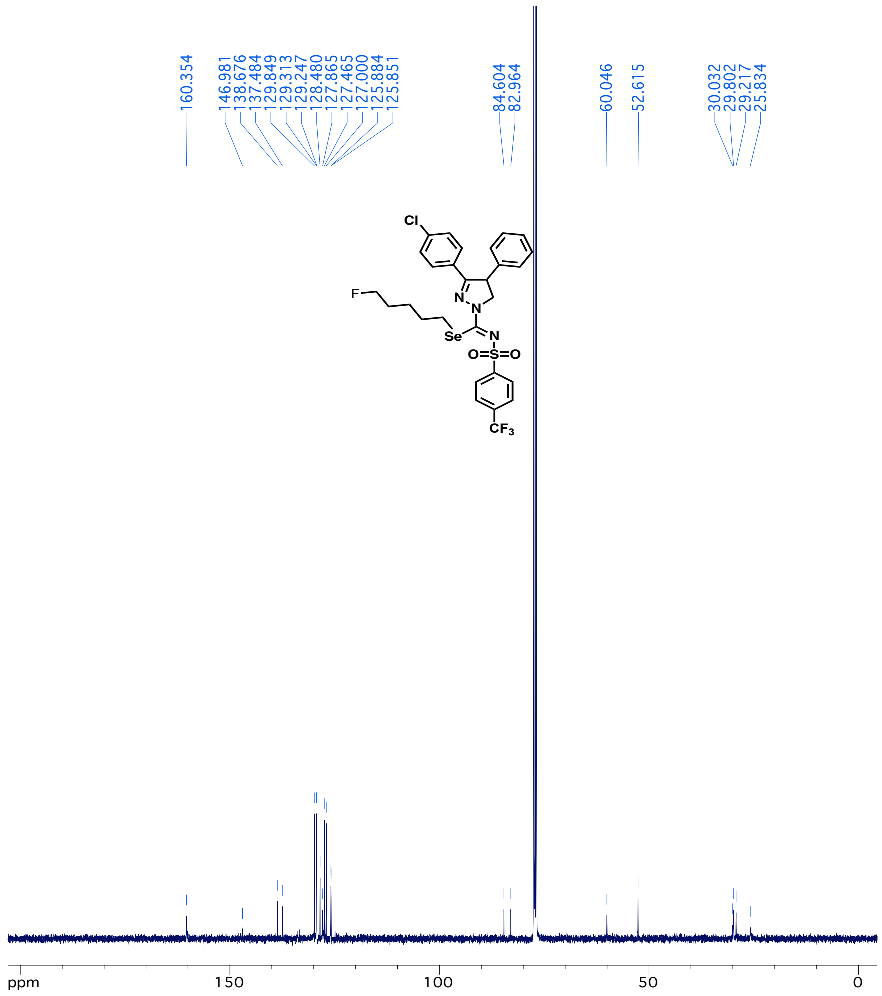

^{1}HNMR and ^{13}CNMR of **12**

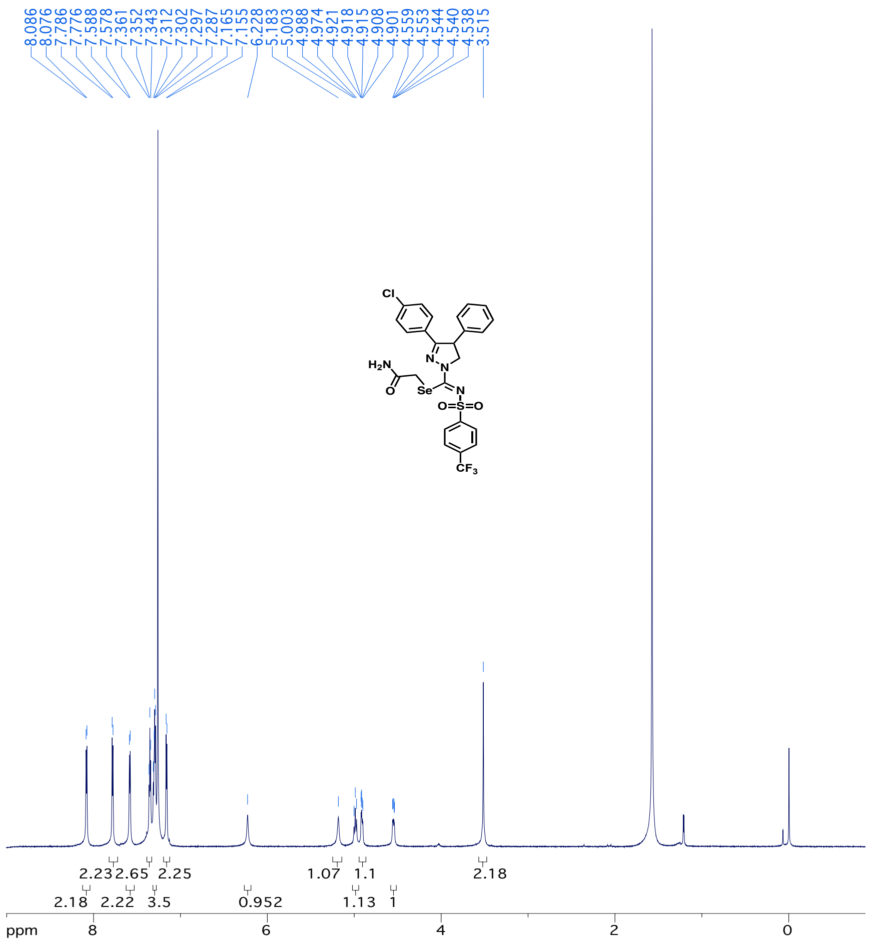

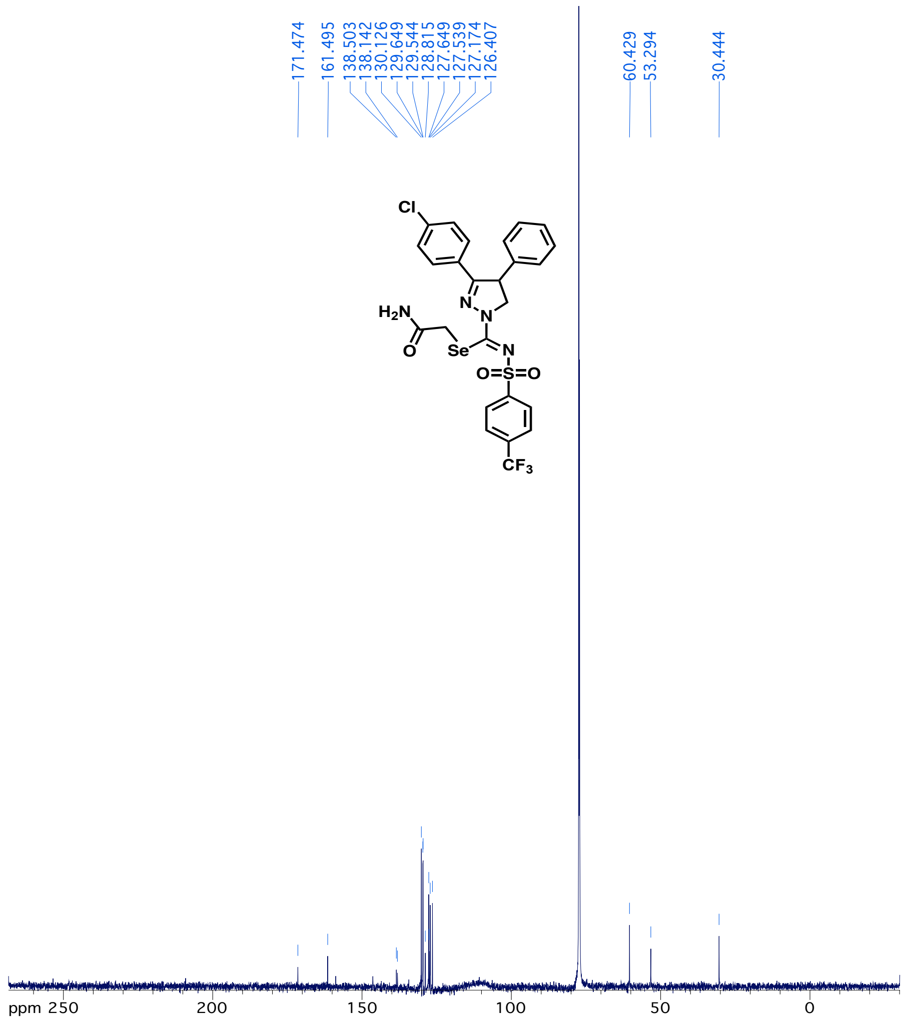

^{1}HNMR and ^{13}CNMR of **13**

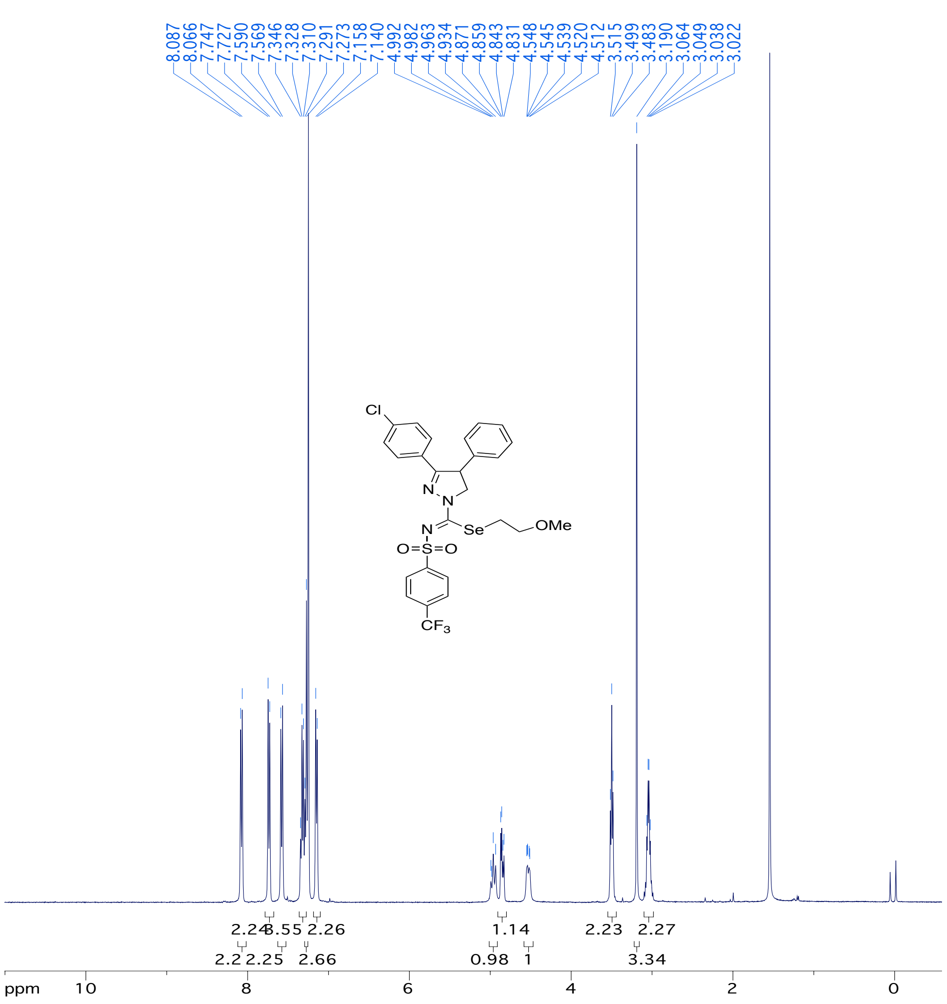

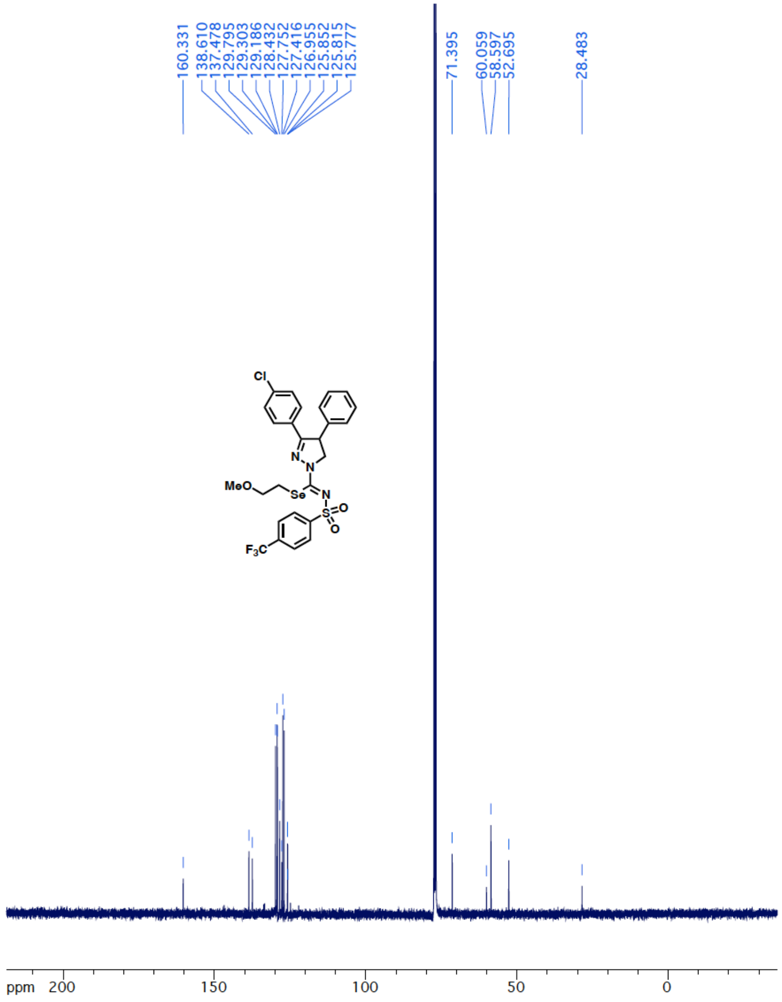

^{1}HNMR and ^{13}CNMR of **14**

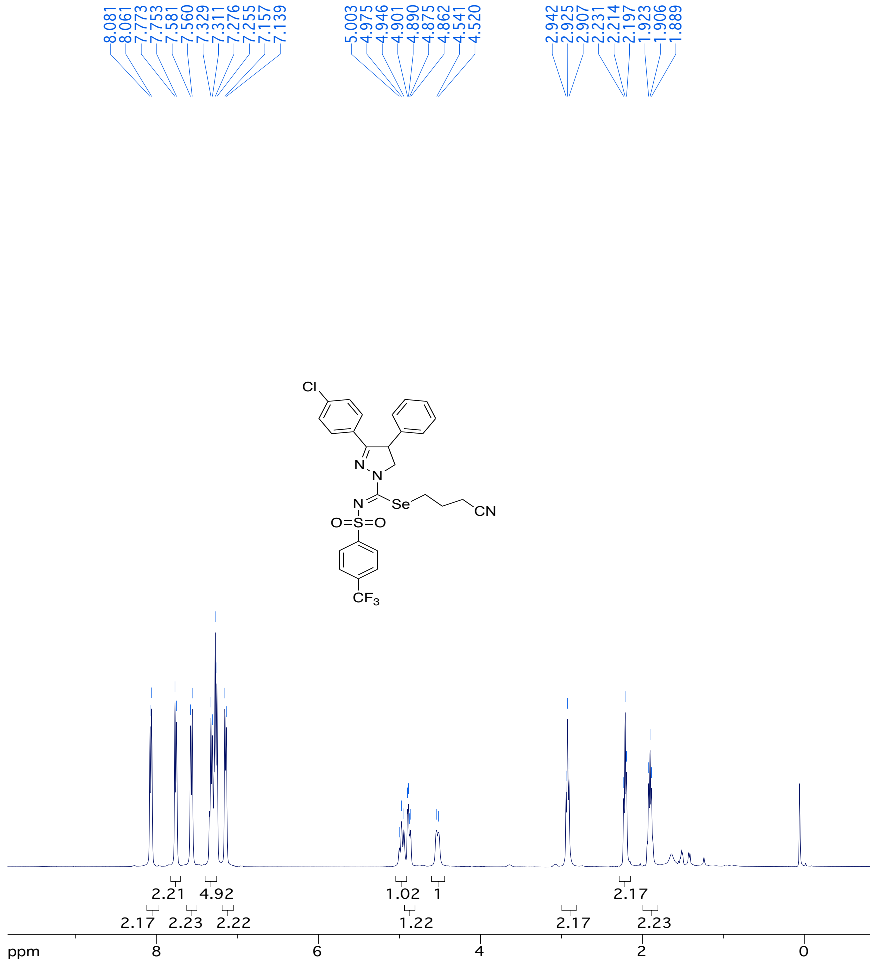

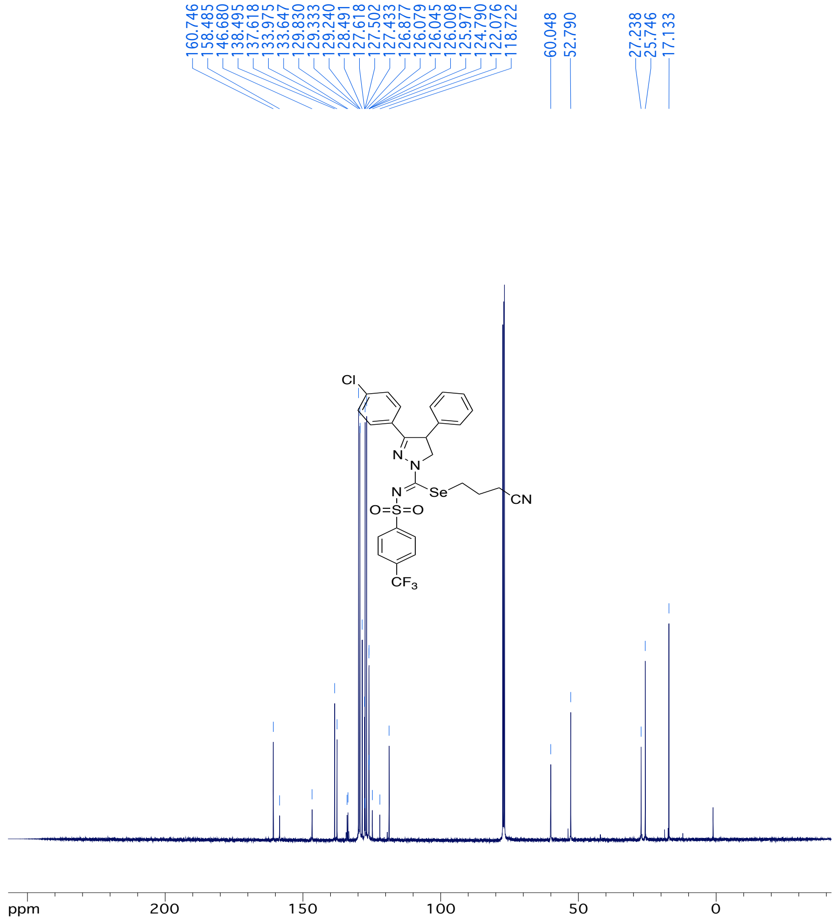

^{1}HNMR and ^{13}CNMR of **15**

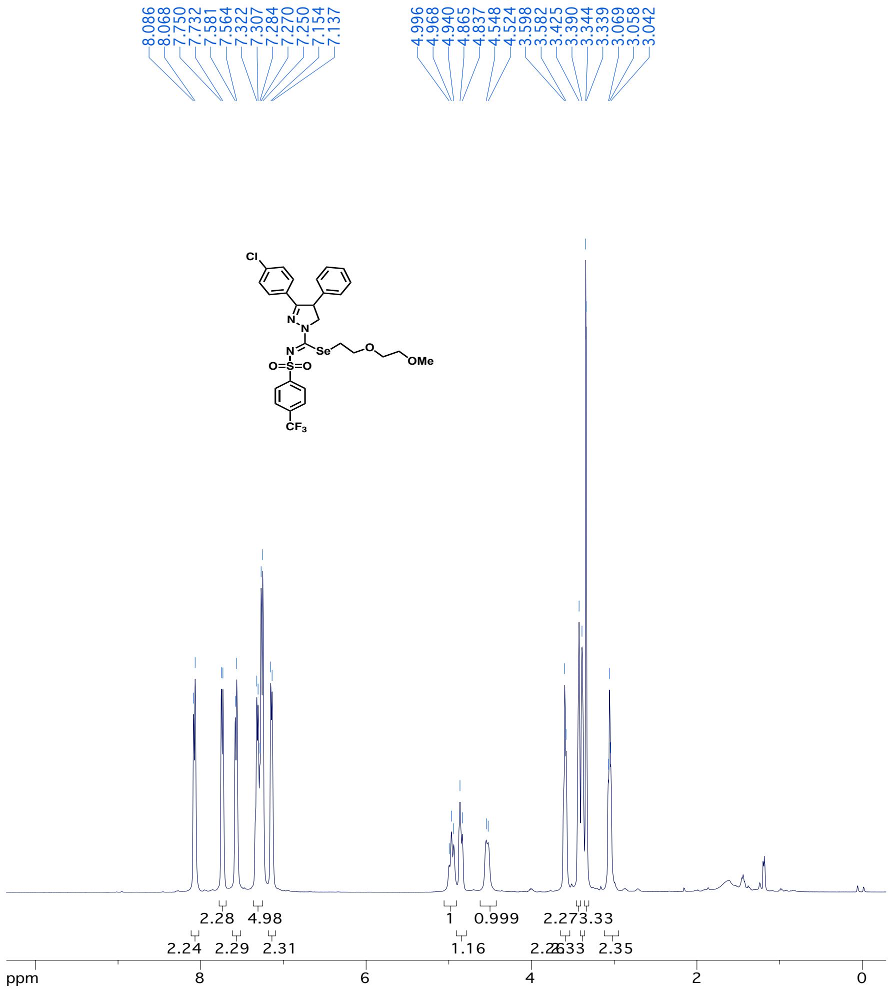

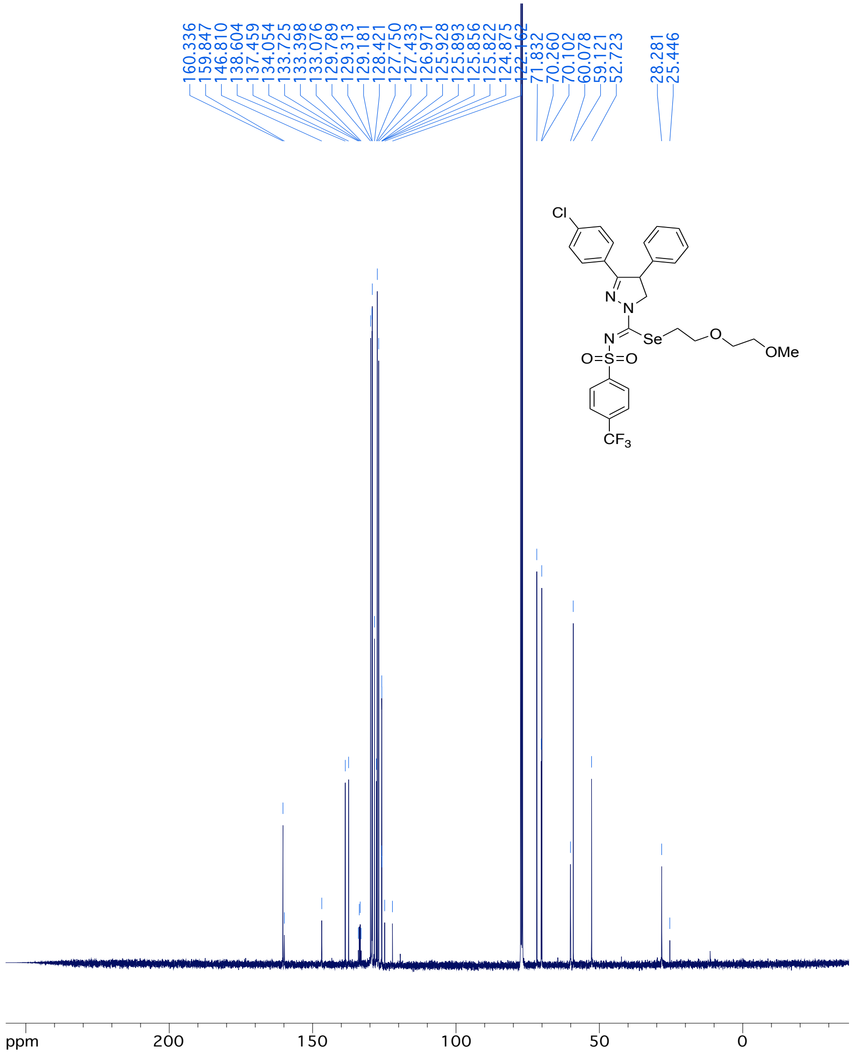

^{1}HNMR and ^{13}CNMR of **16**

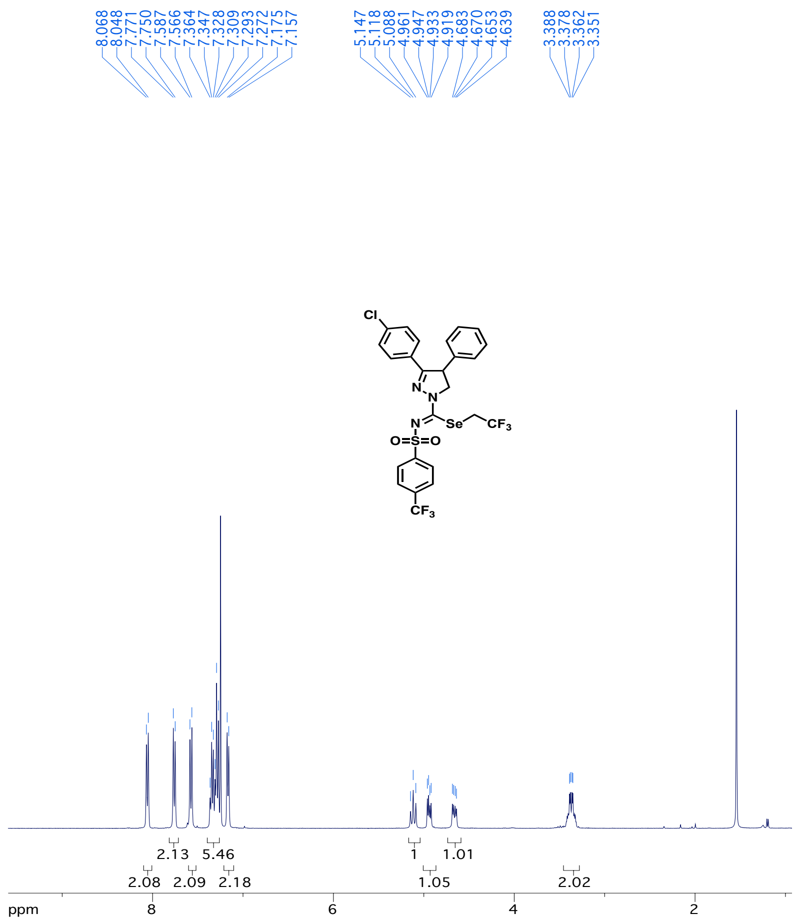

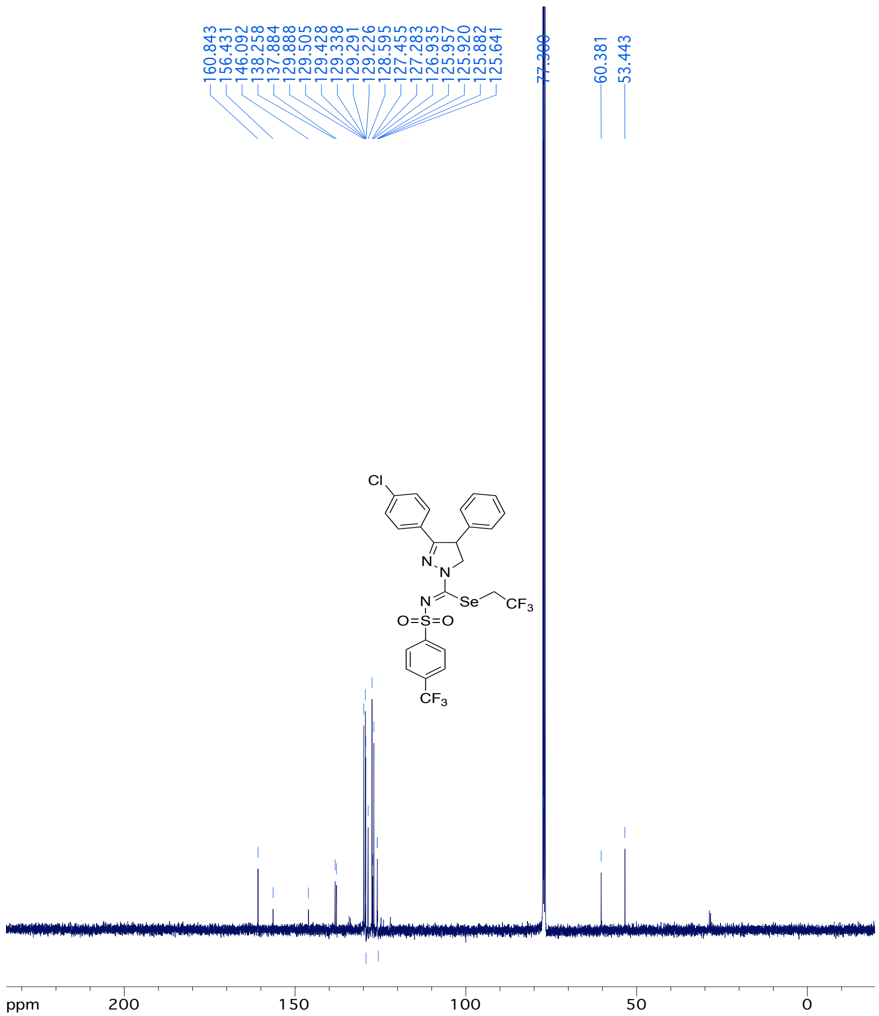

^{1}HNMR and ^{13}CNMR of **17**

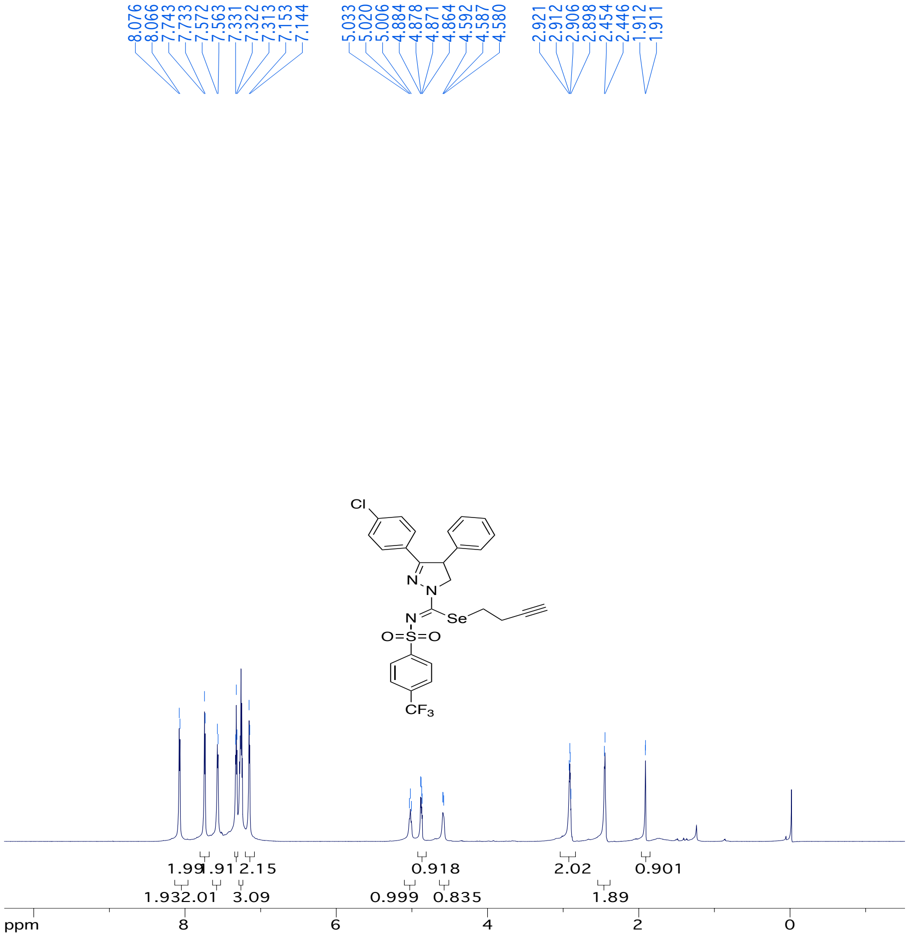

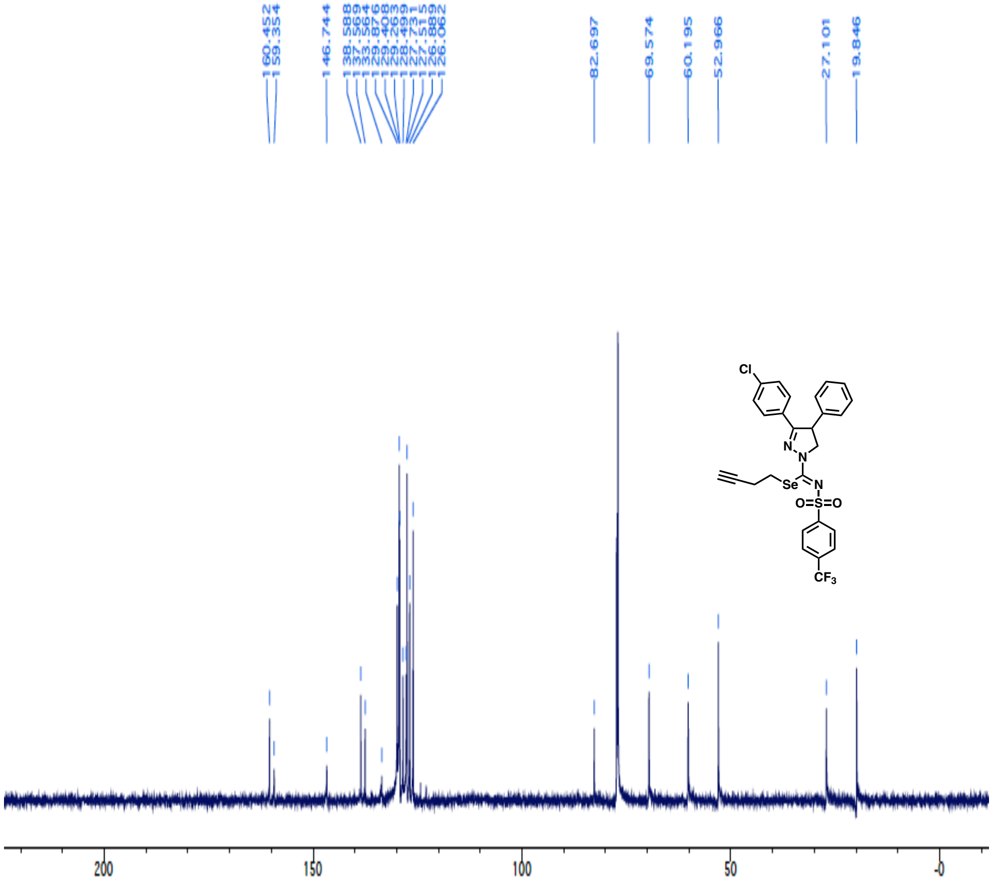

^{1}HNMR and ^{13}CNMR of **18**

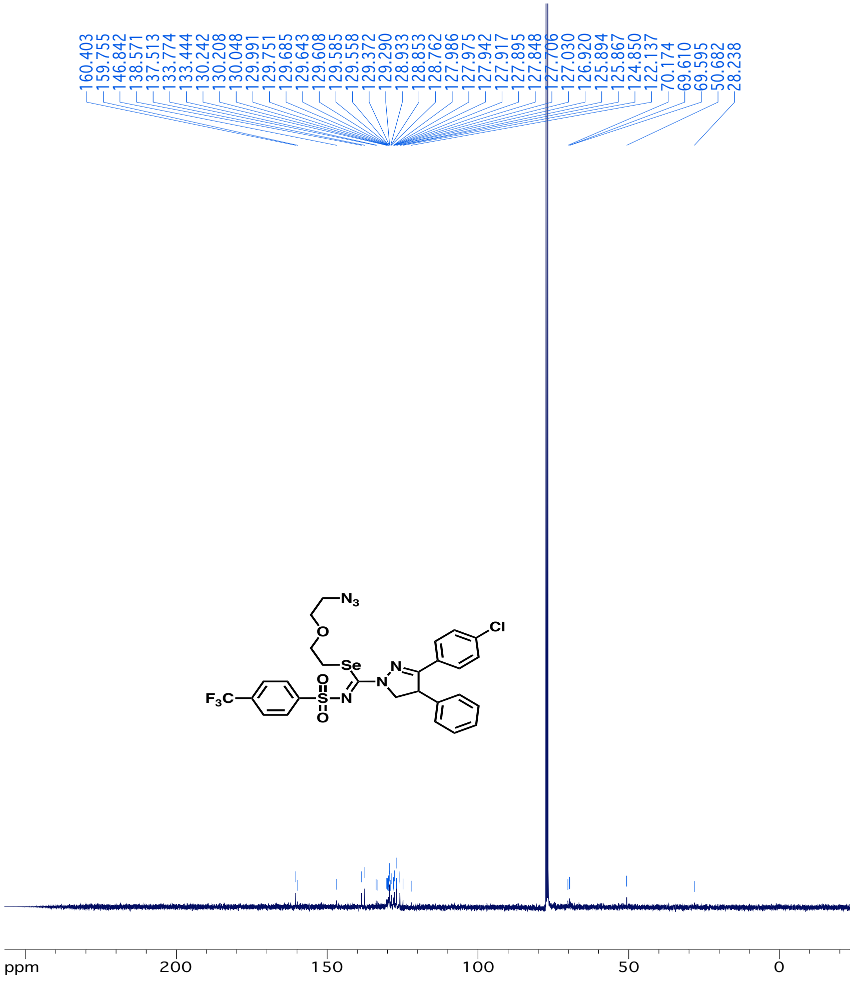

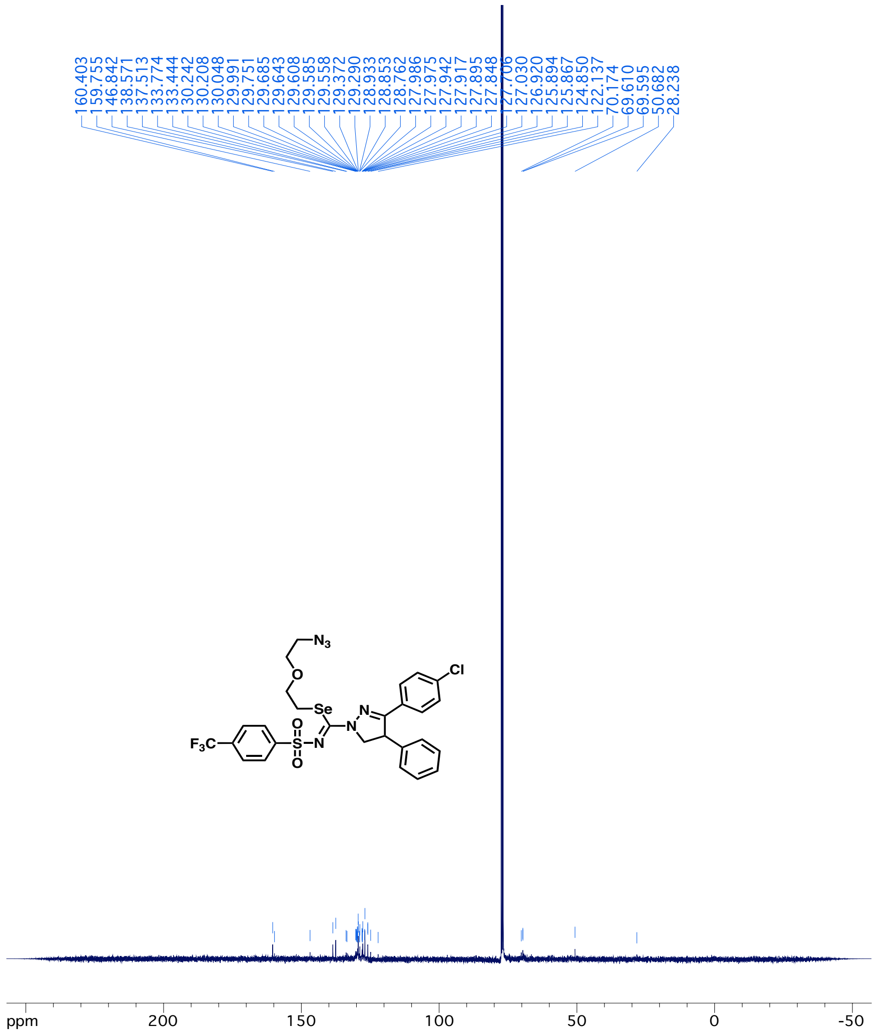

^{1}HNMR and ^{13}CNMR of **19**

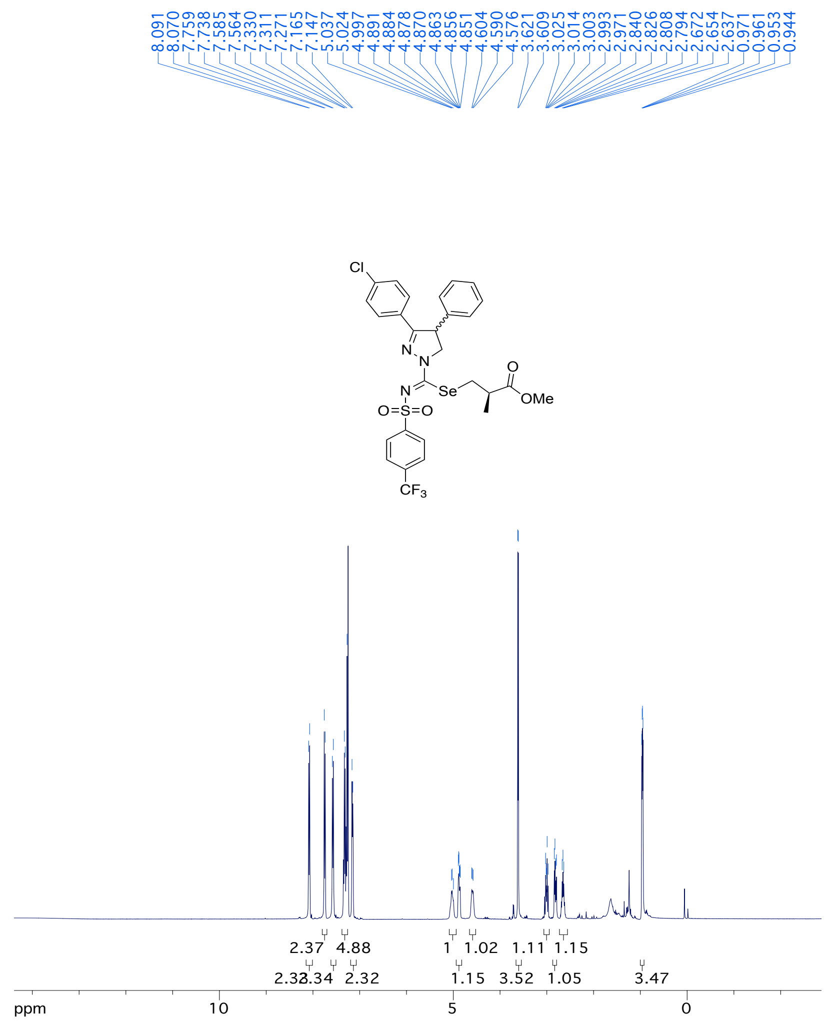

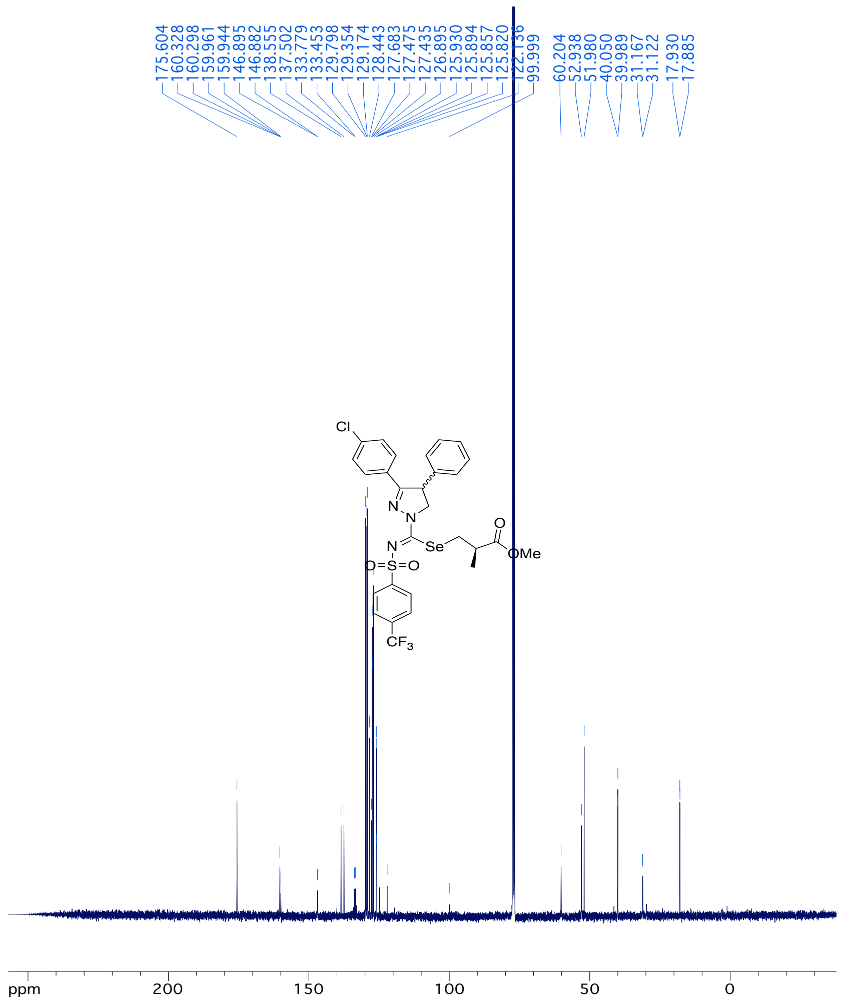

^{1}HNMR and ^{13}CNMR of **20**

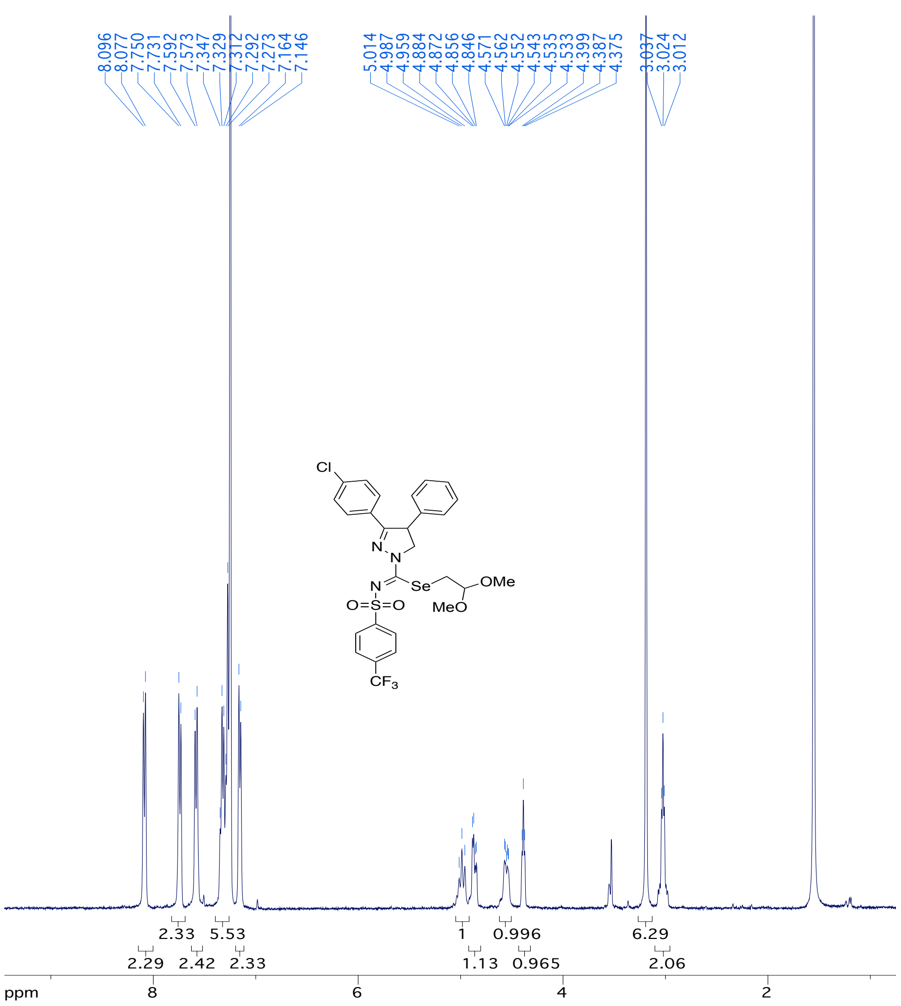

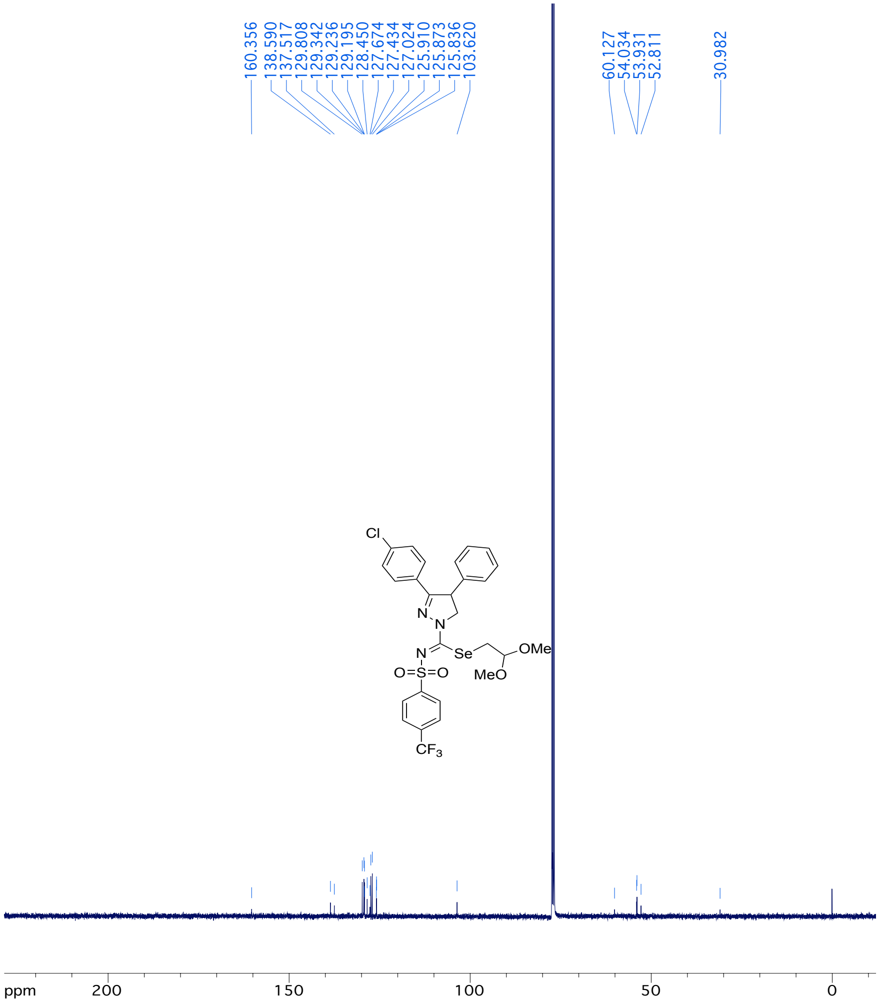

^{1}HNMR and ^{13}CNMR of **21**

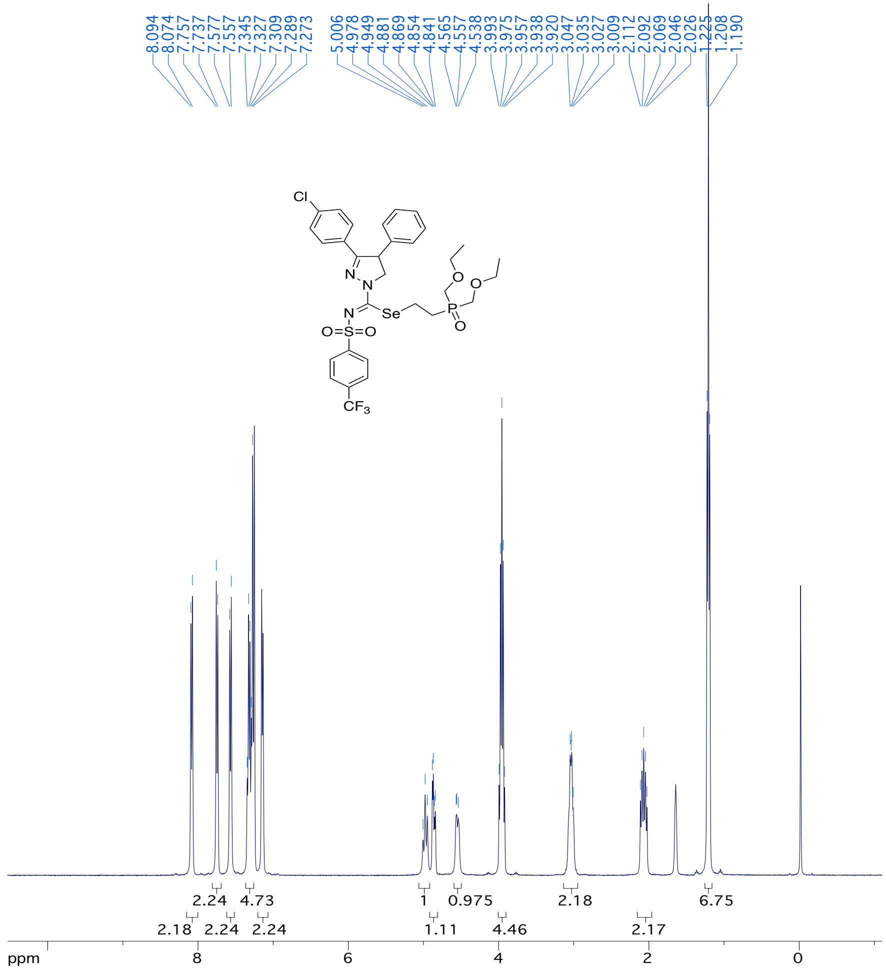

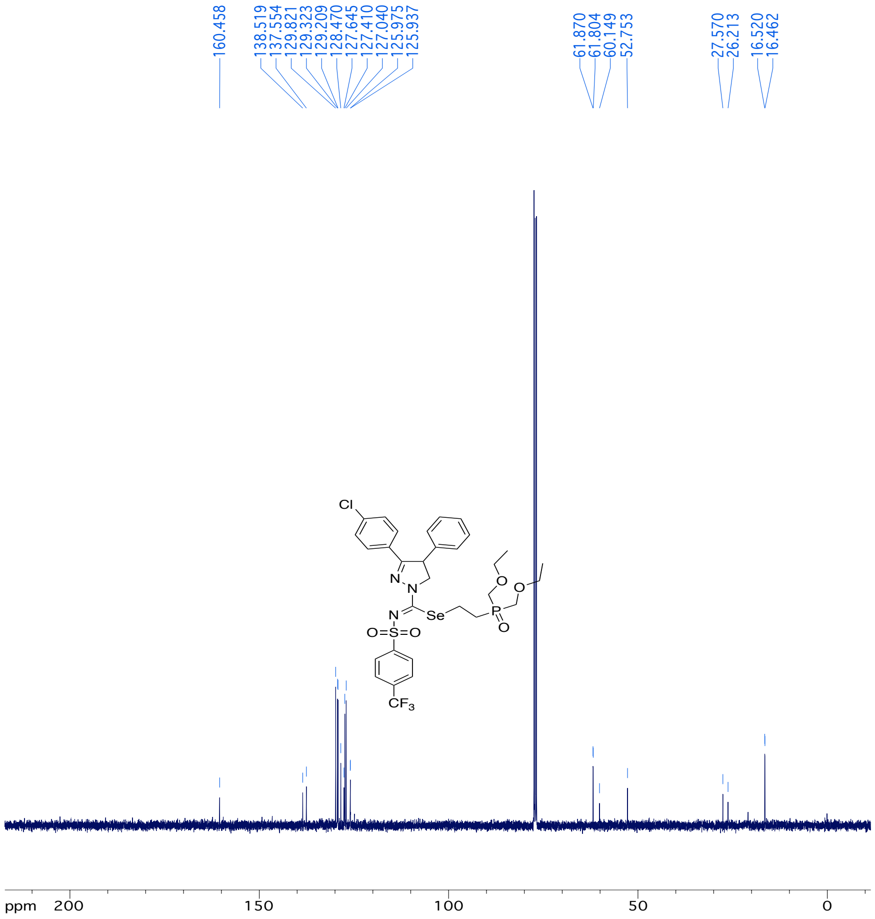

^{1}HNMR and ^{13}CNMR of **22**

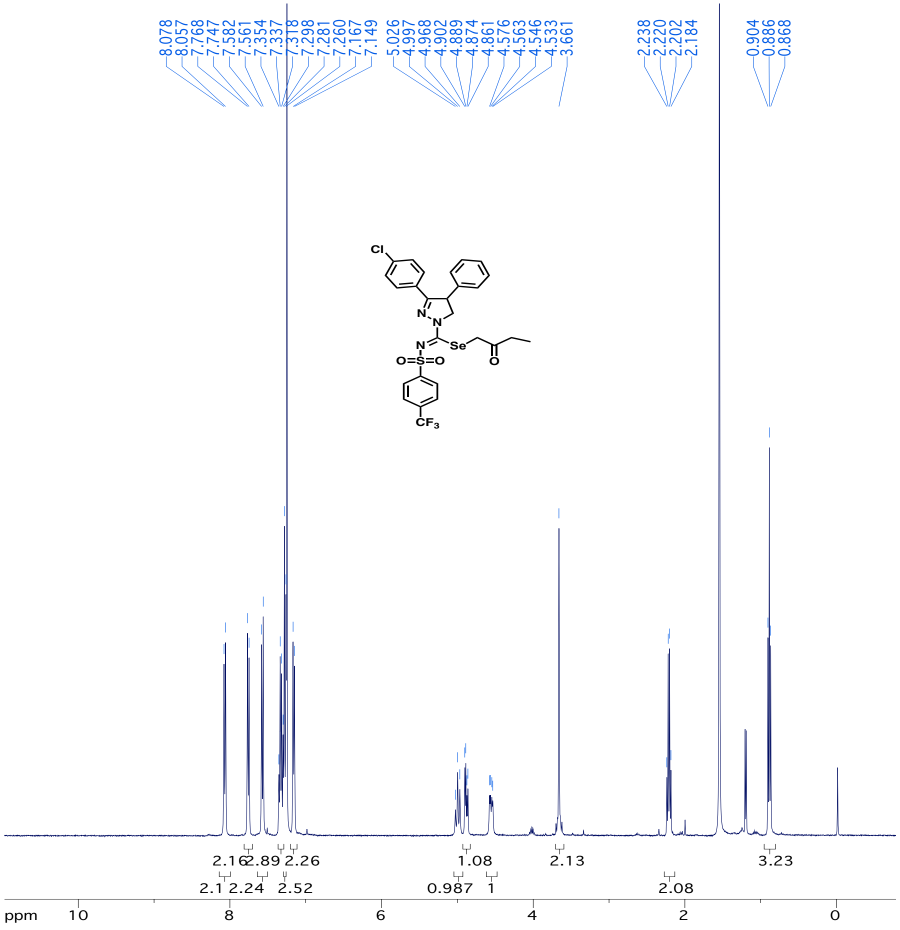

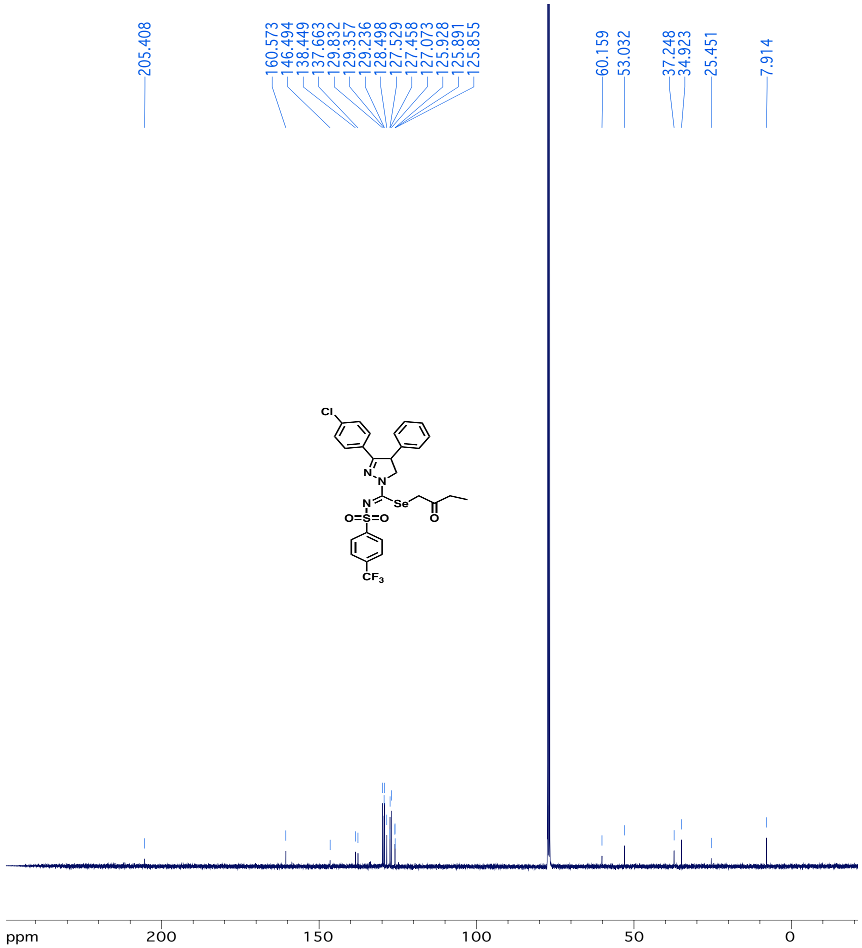

^{1}HNMR and ^{13}CNMR of **23**

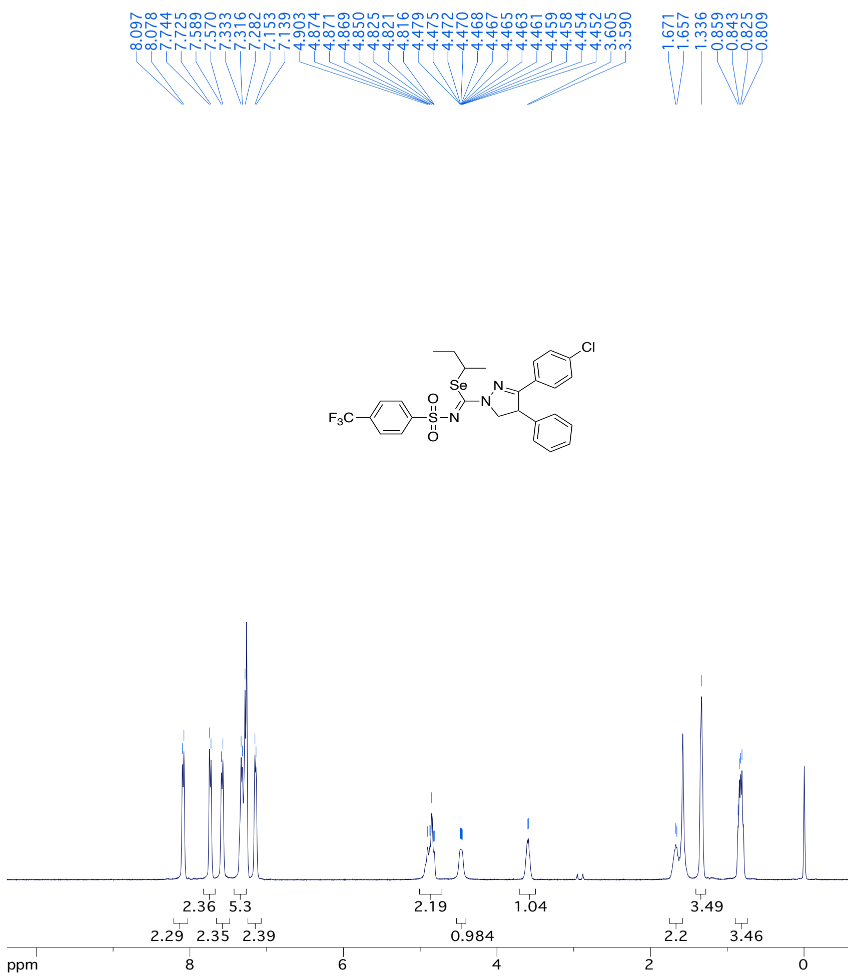

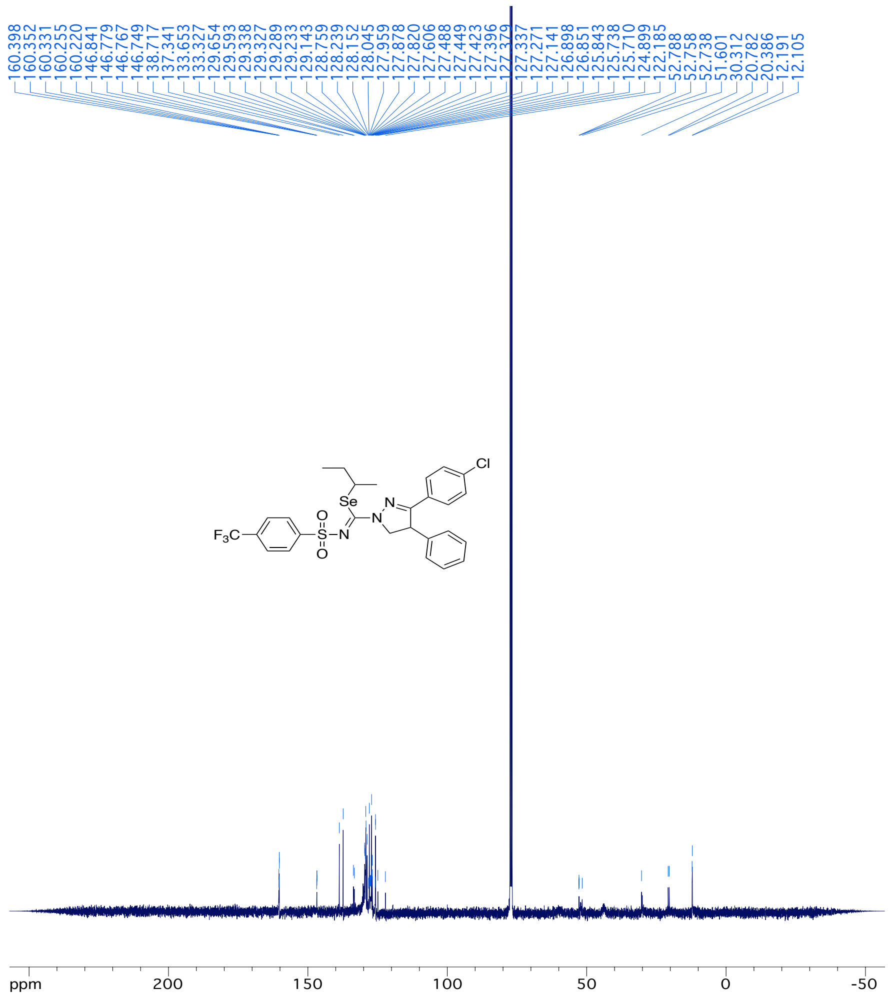

^{1}HNMR and ^{13}CNMR of **24**

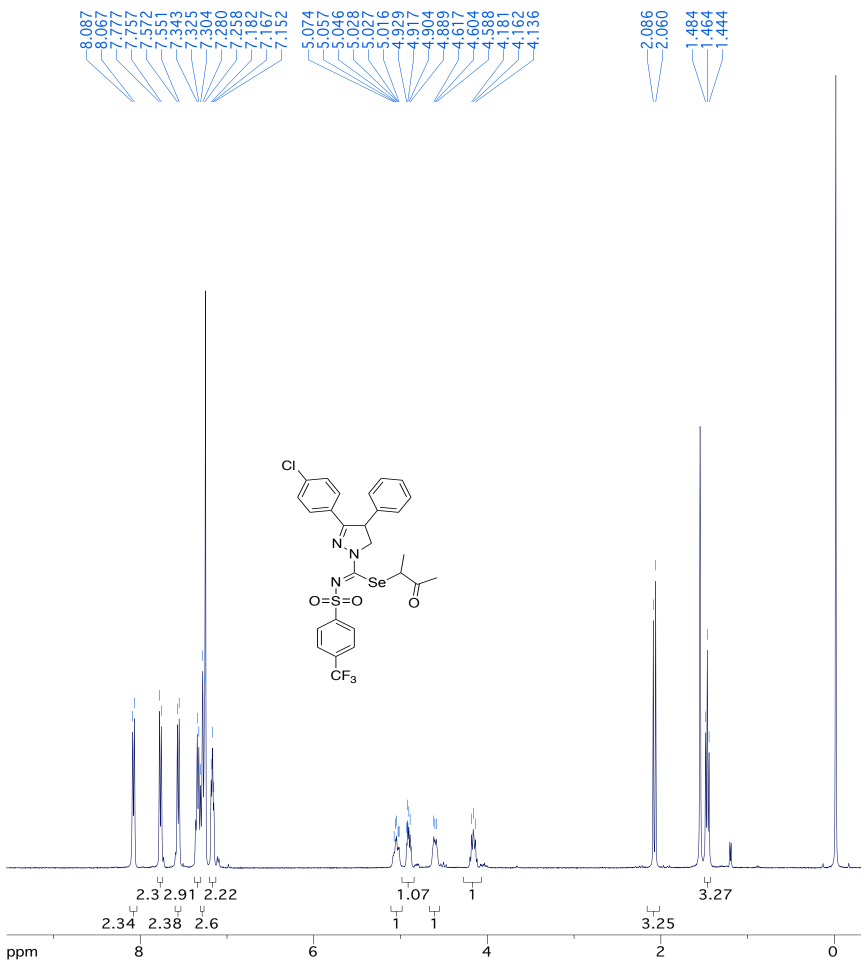

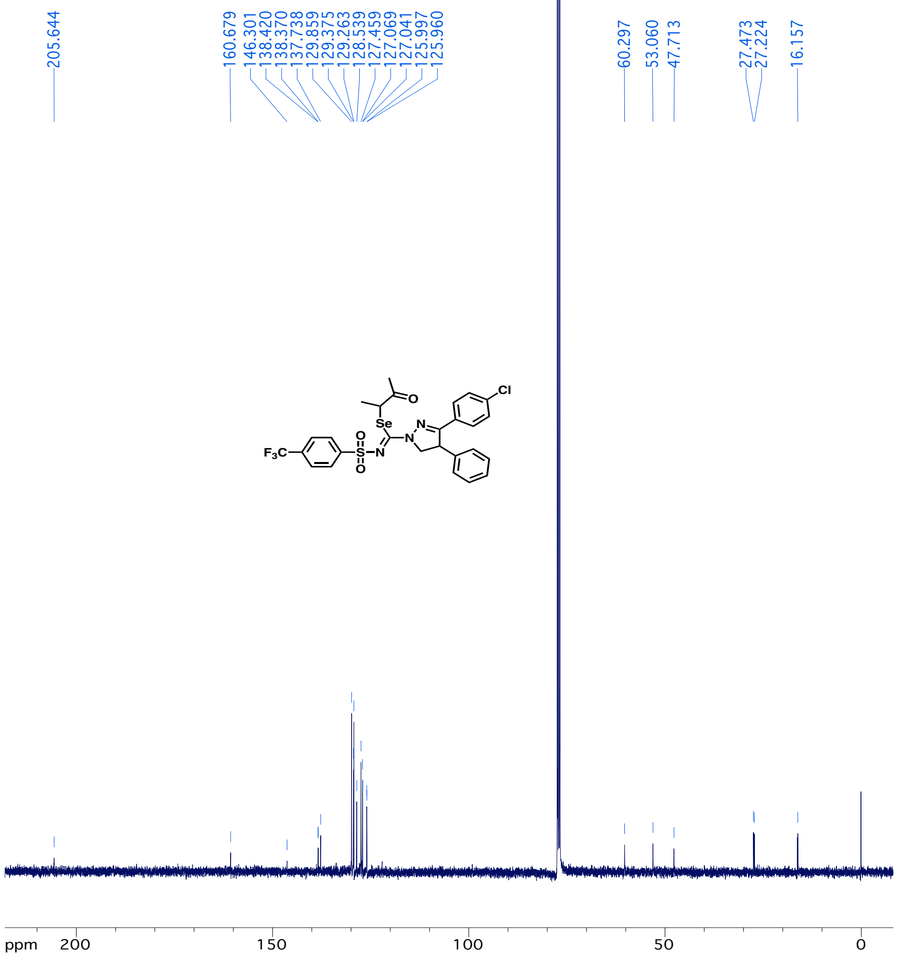

^{1}HNMR and ^{13}CNMR of **25**

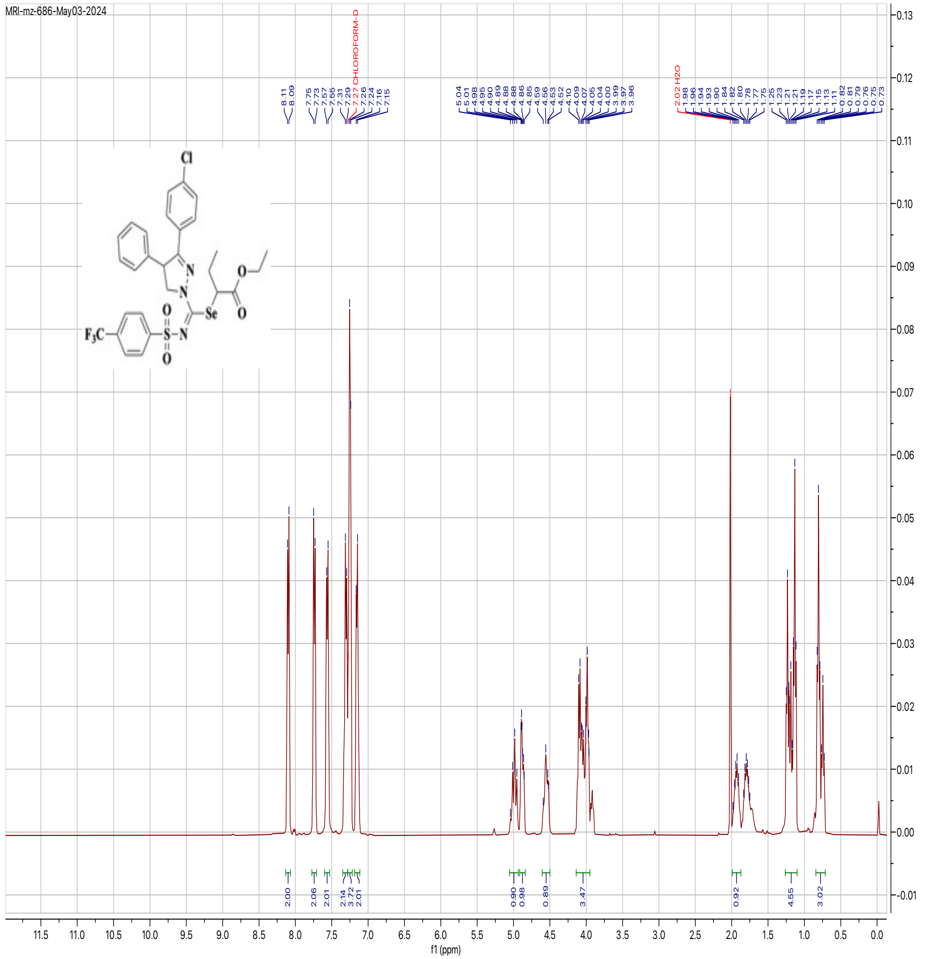

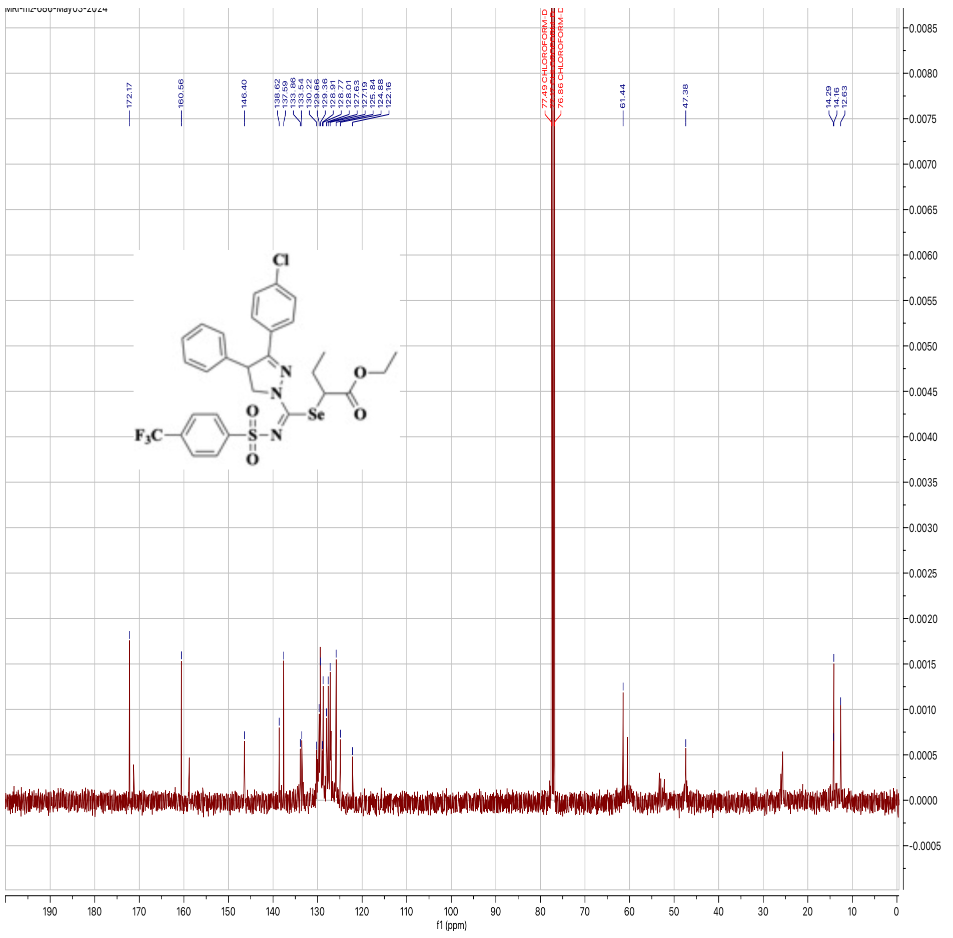

^{1}HNMR and ^{13}CNMR of **26**

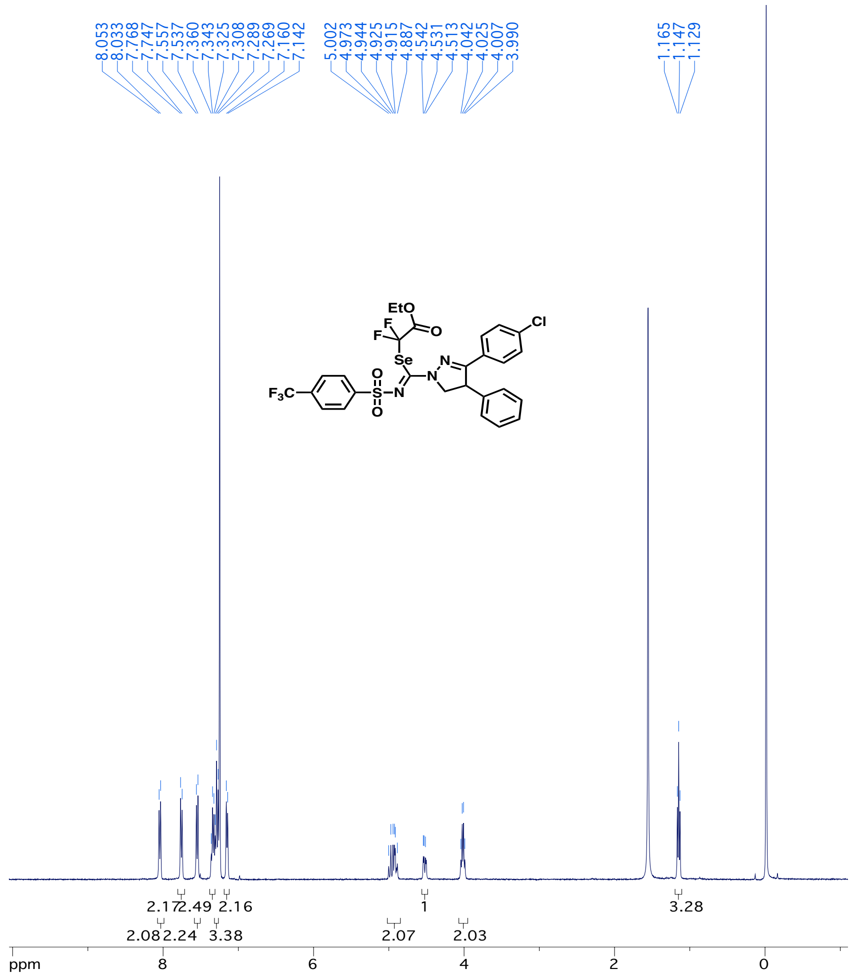

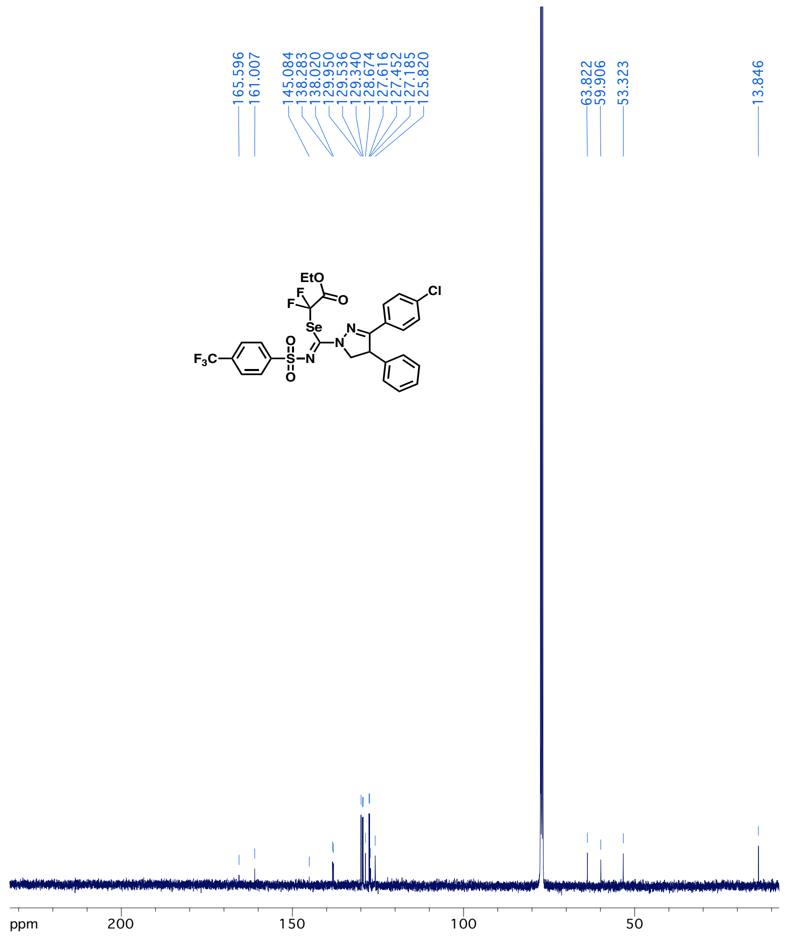

^{1}HNMR and ^{13}CNMR of **27**

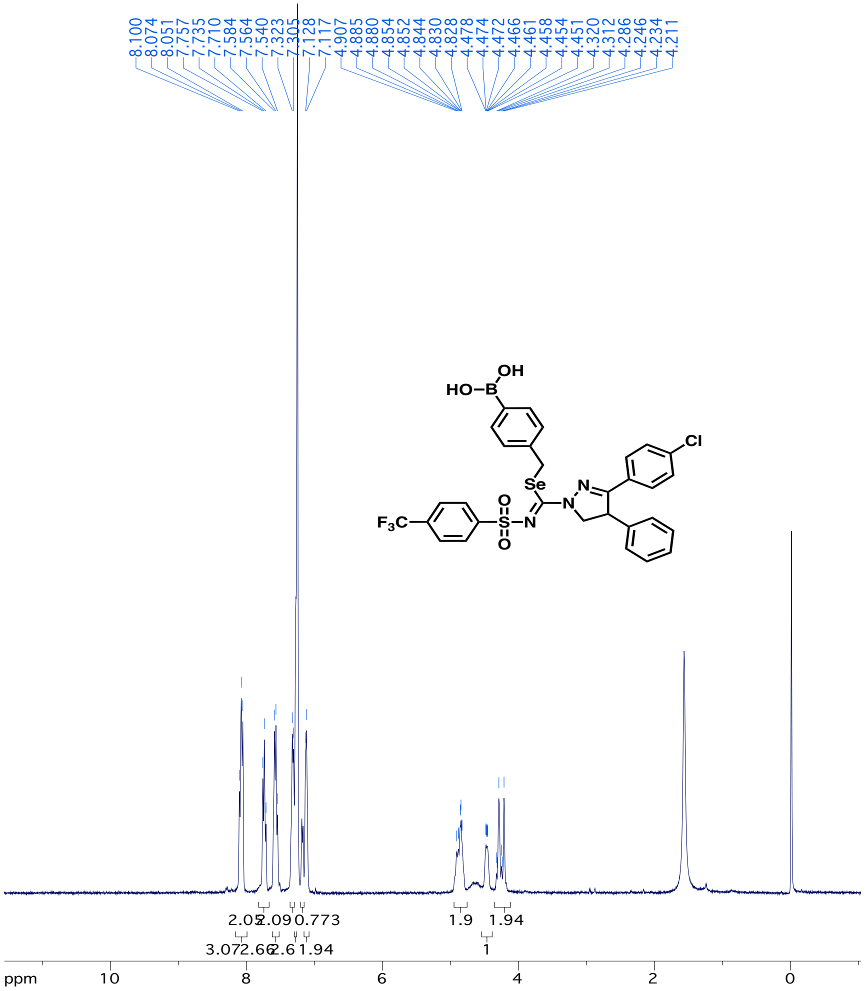

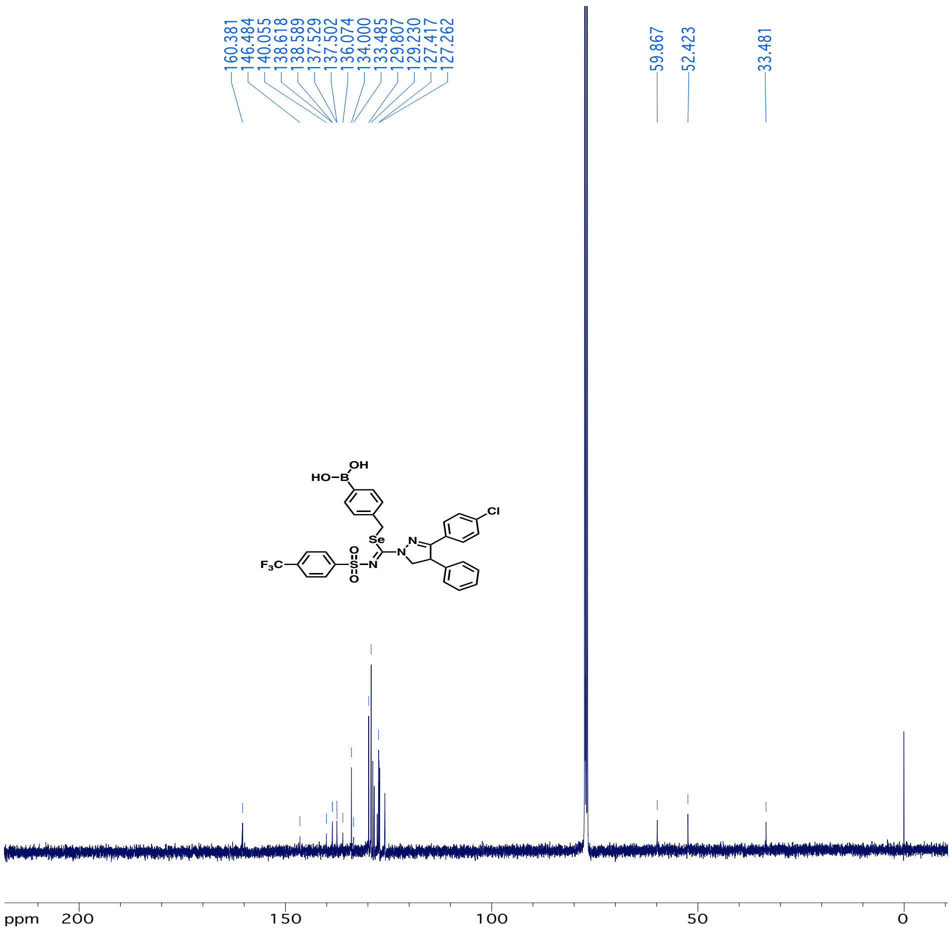

^{1}HNMR and ^{13}CNMR of **28**

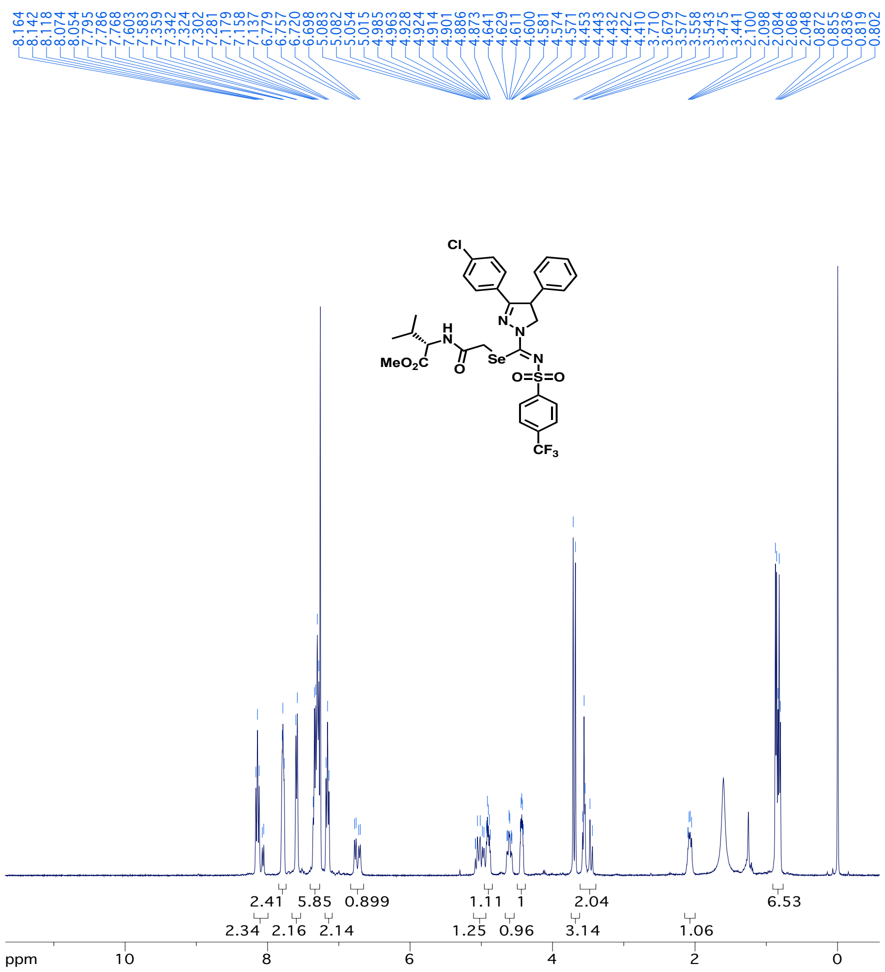

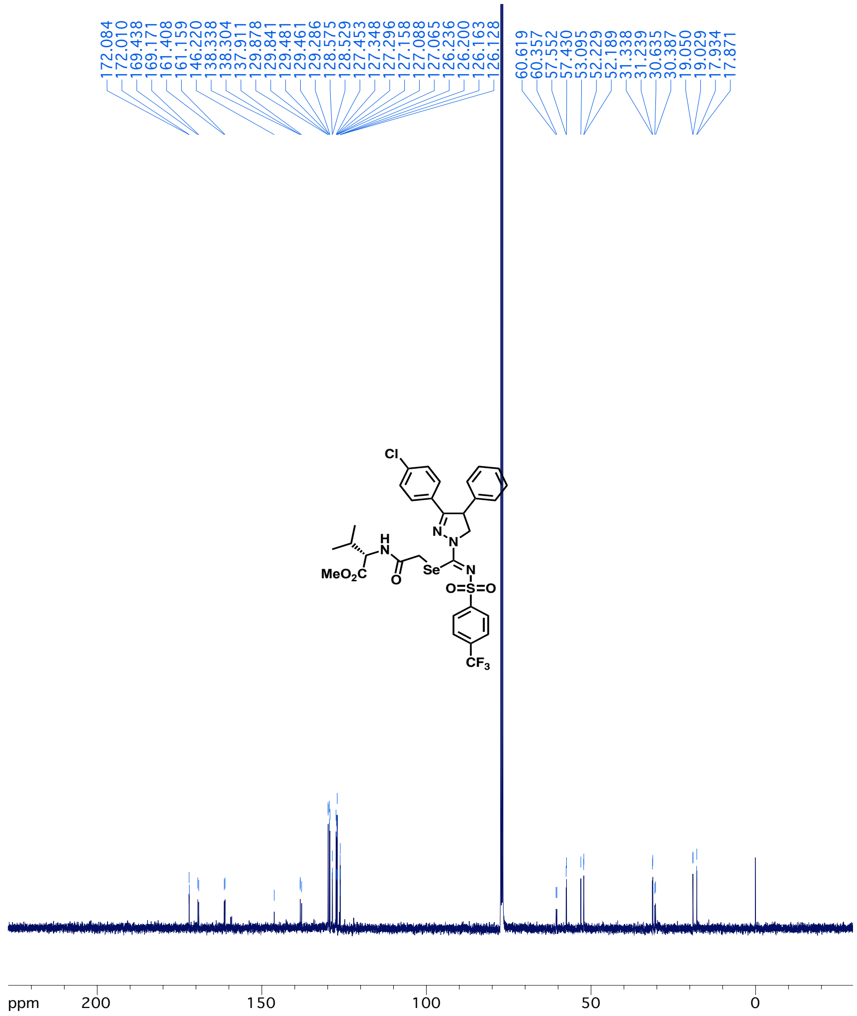

^{1}HNMR and ^{13}CNMR of **29**

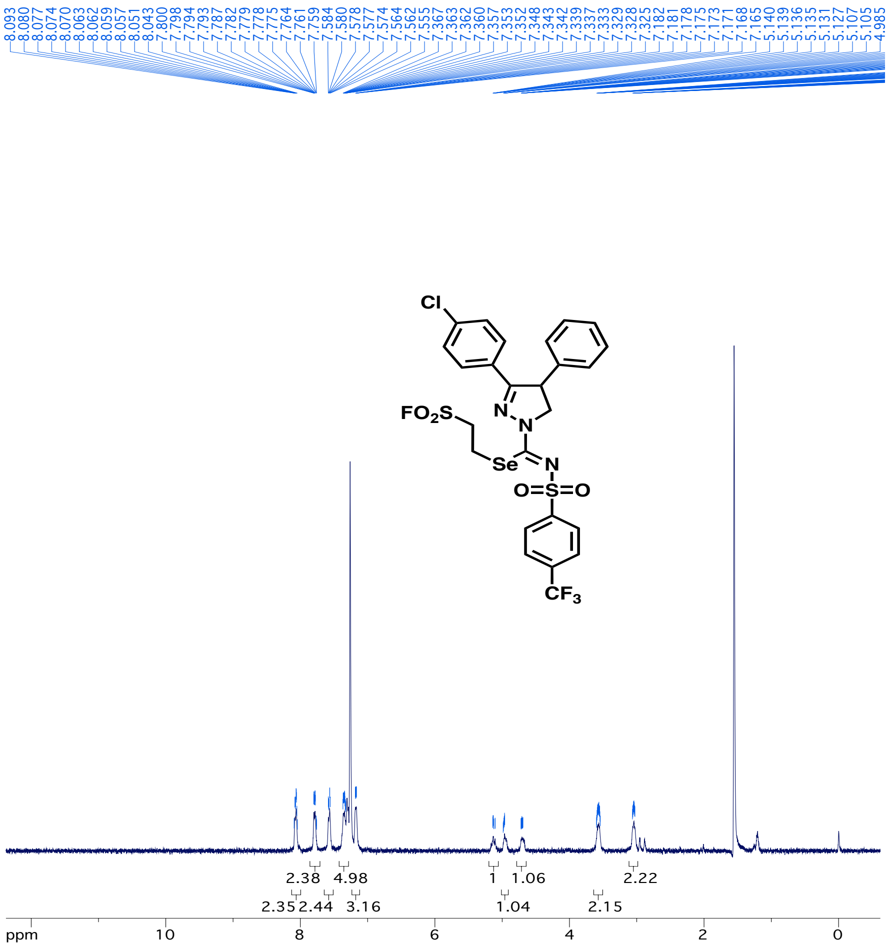

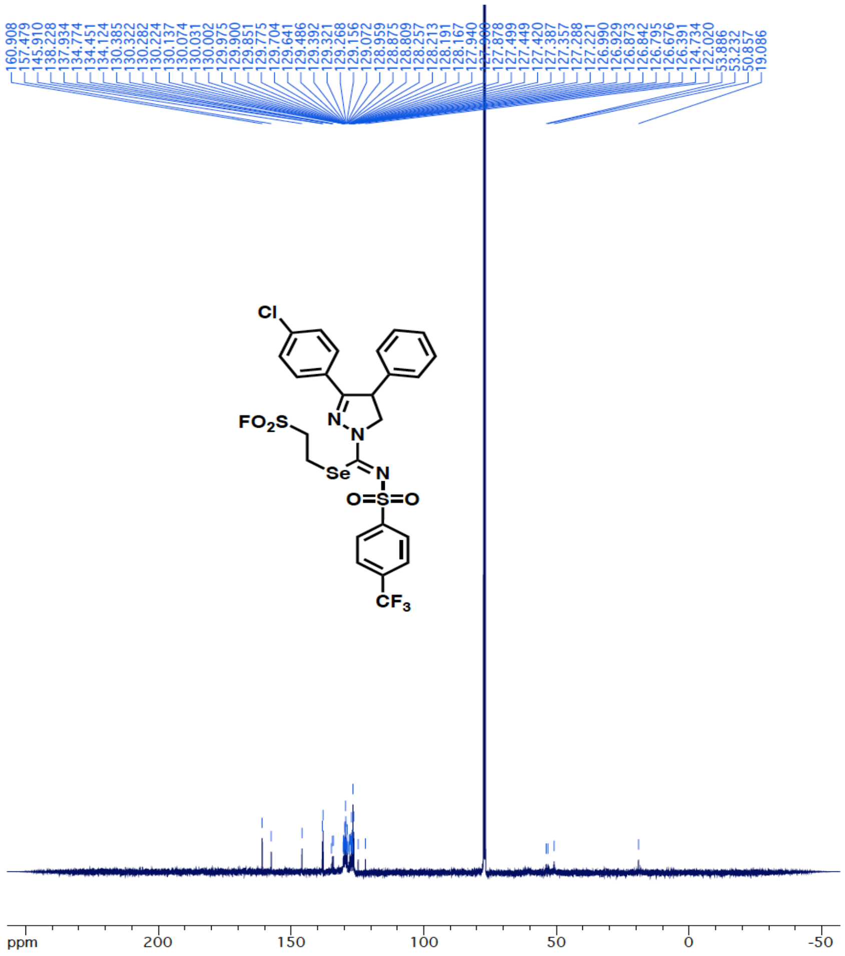

^{1}HNMR and ^{13}CNMR of **30**

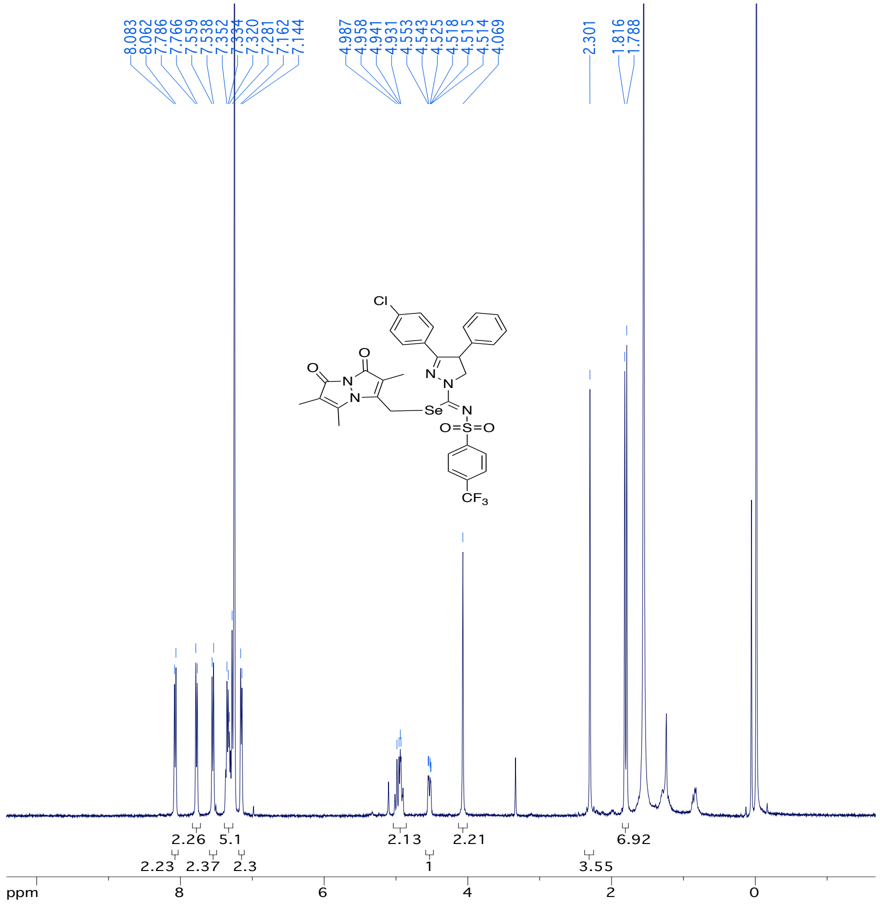

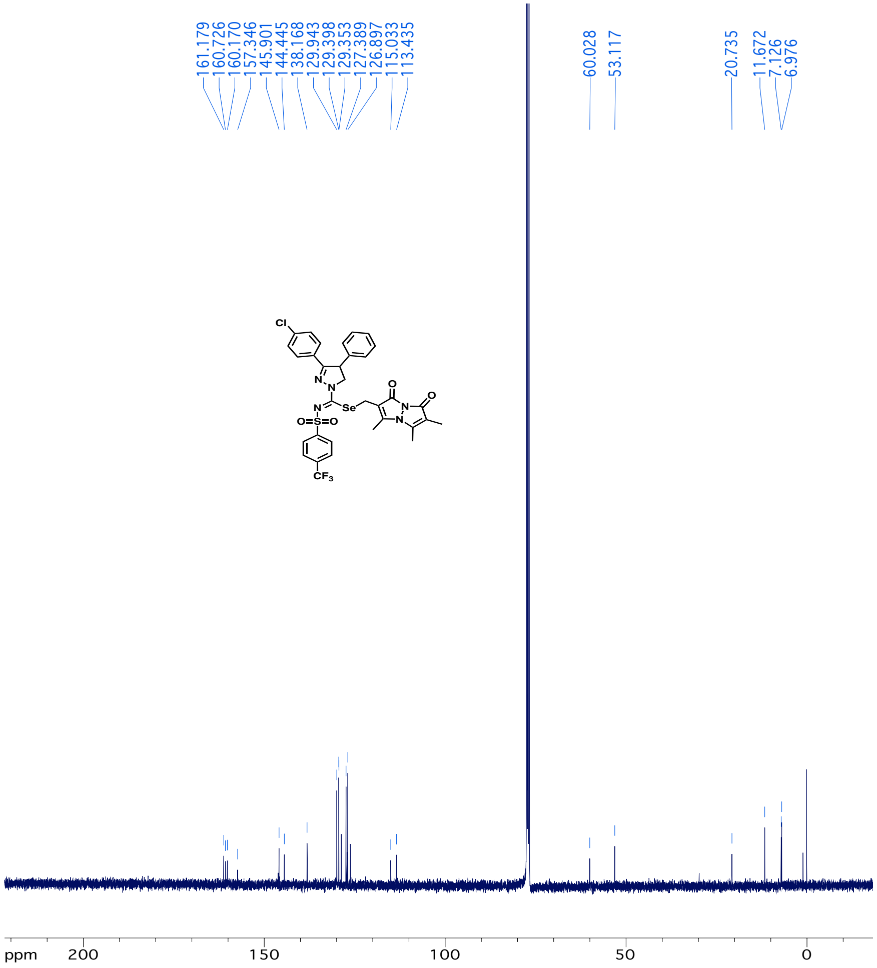

^{1}HNMR and ^{13}CNMR of **31**

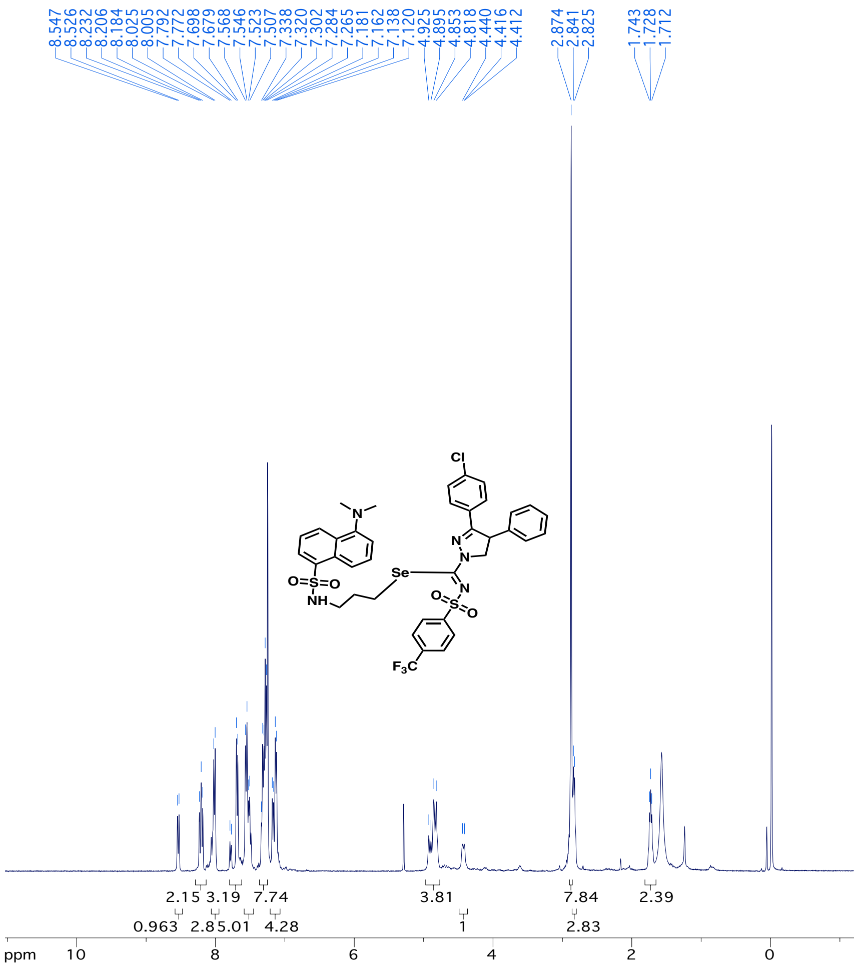

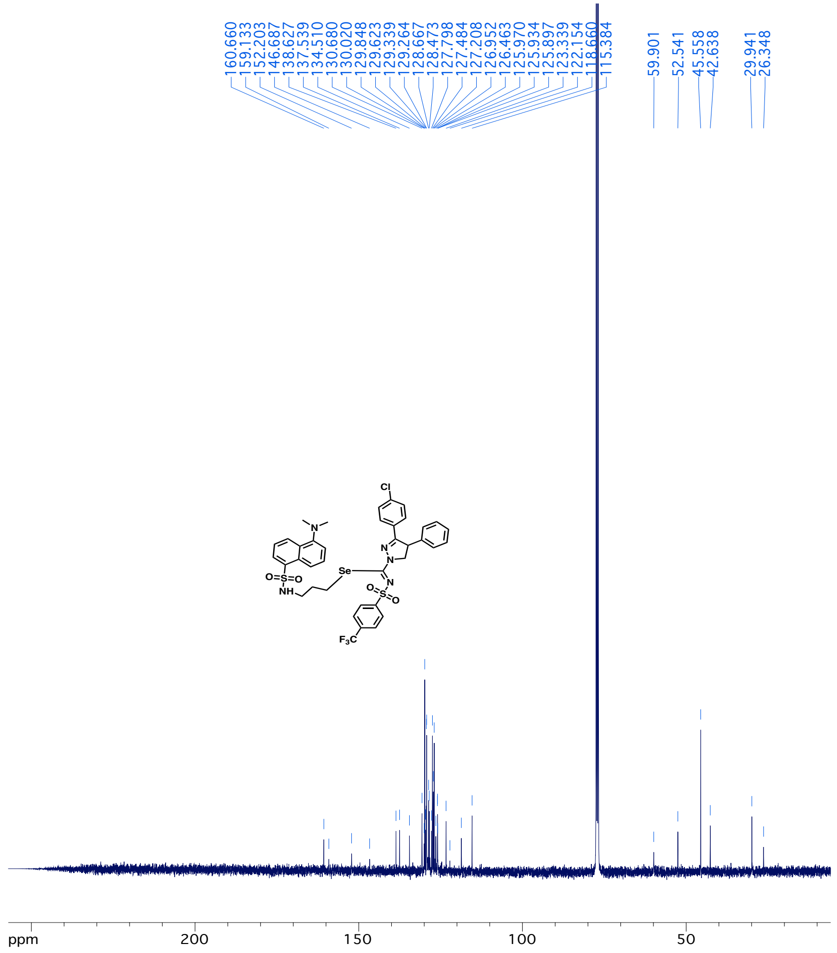

^{1}HNMR and ^{13}CNMR of **32**

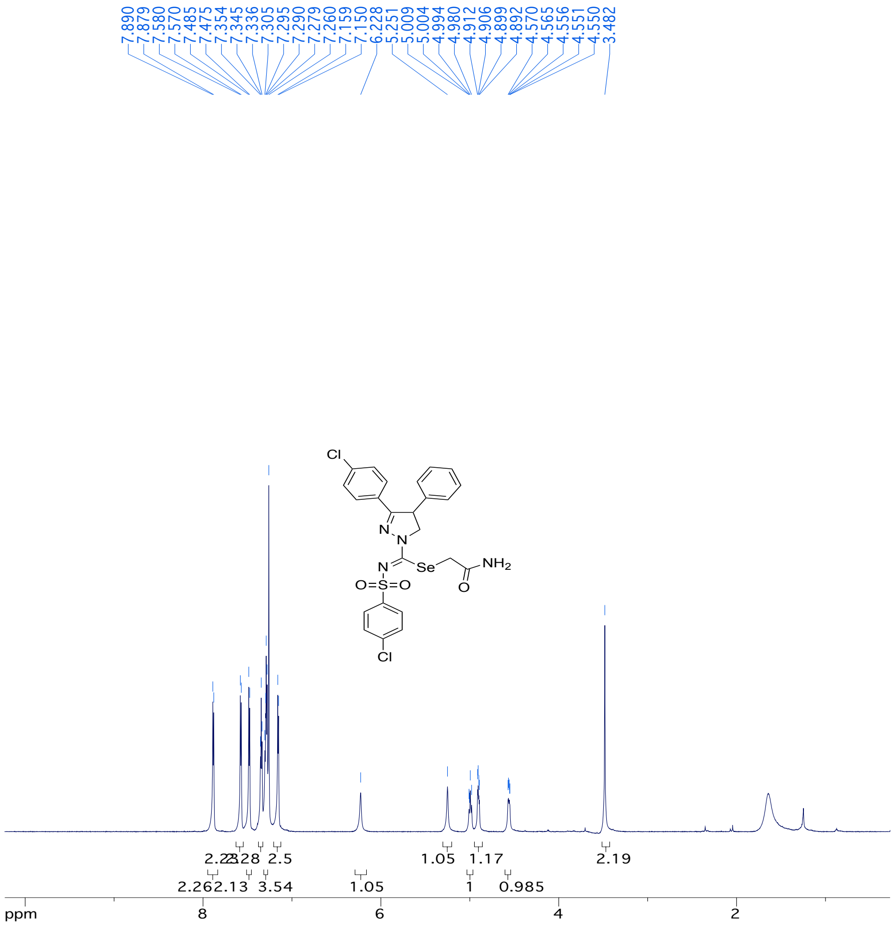

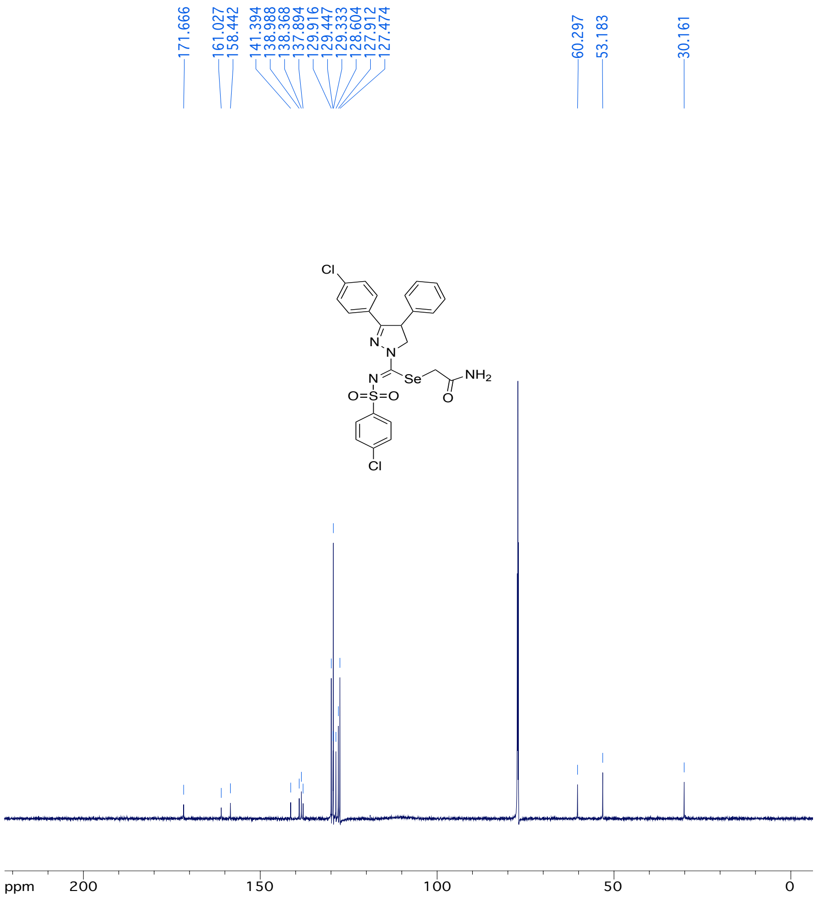

^{1}HNMR and ^{13}CNMR of **33**

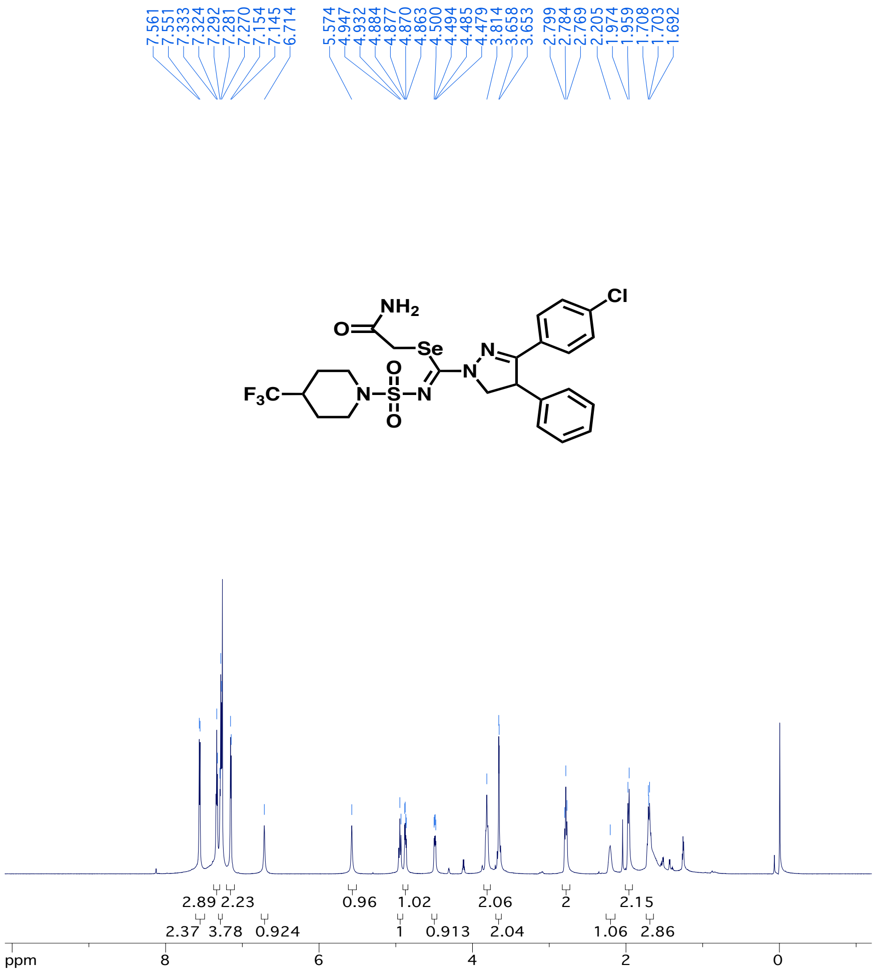

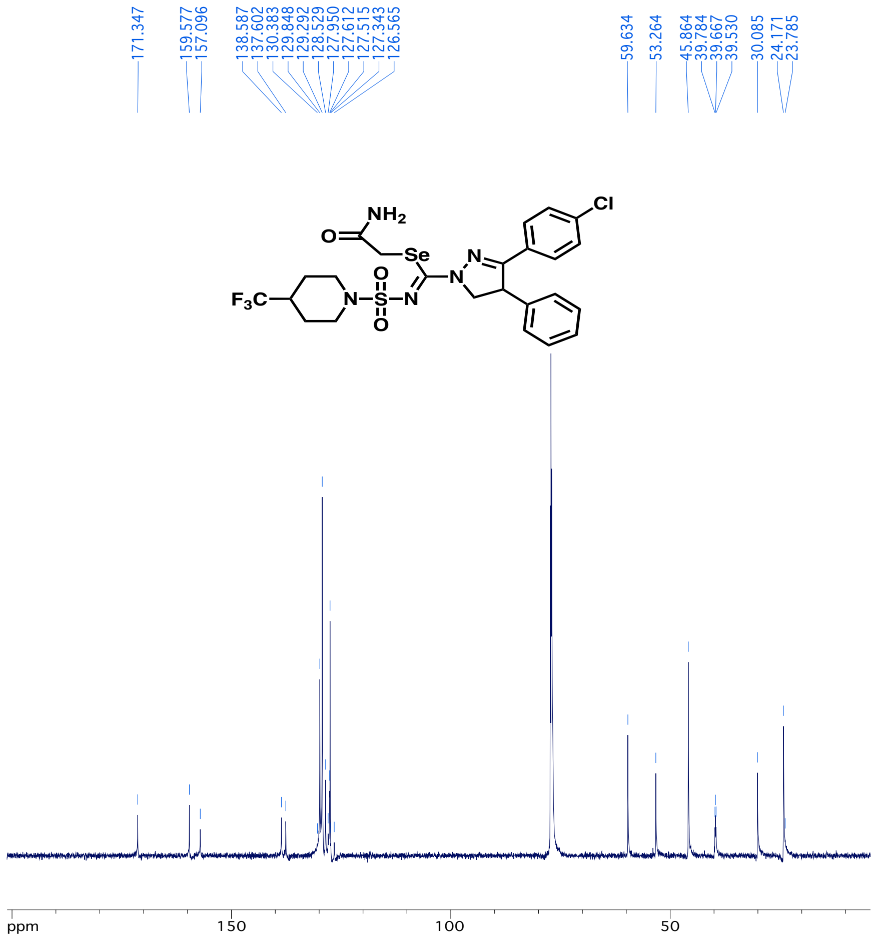

^{1}HNMR and ^{13}CNMR of **34**

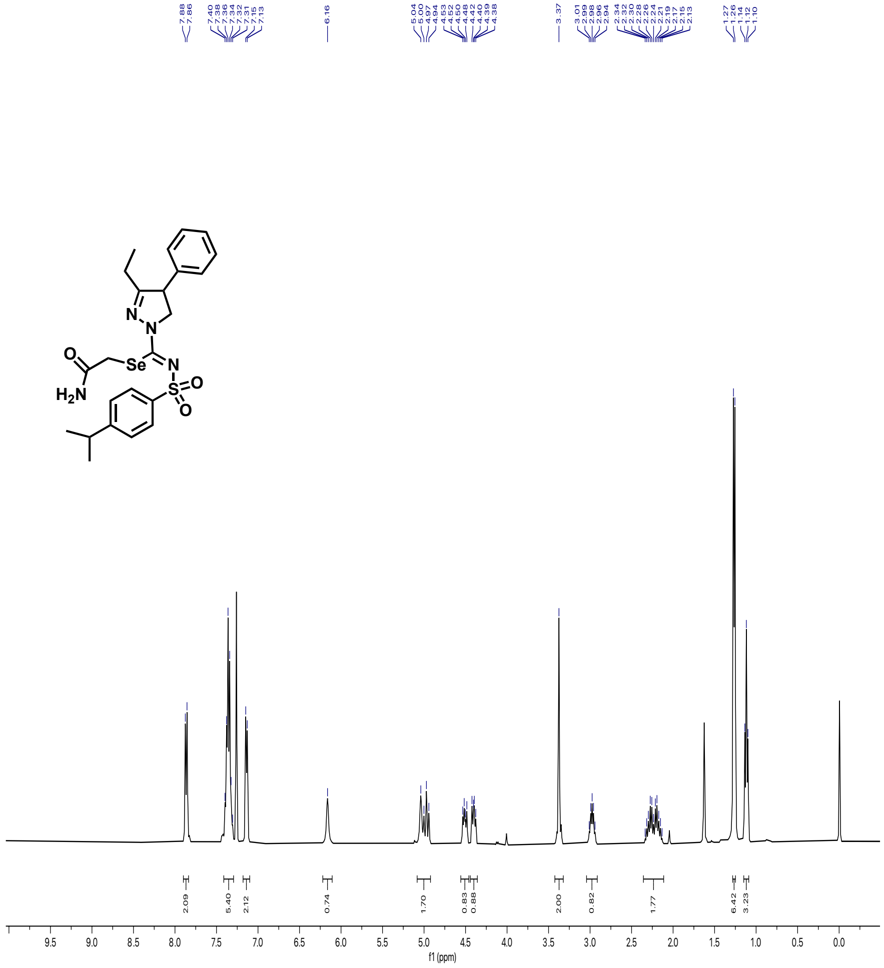

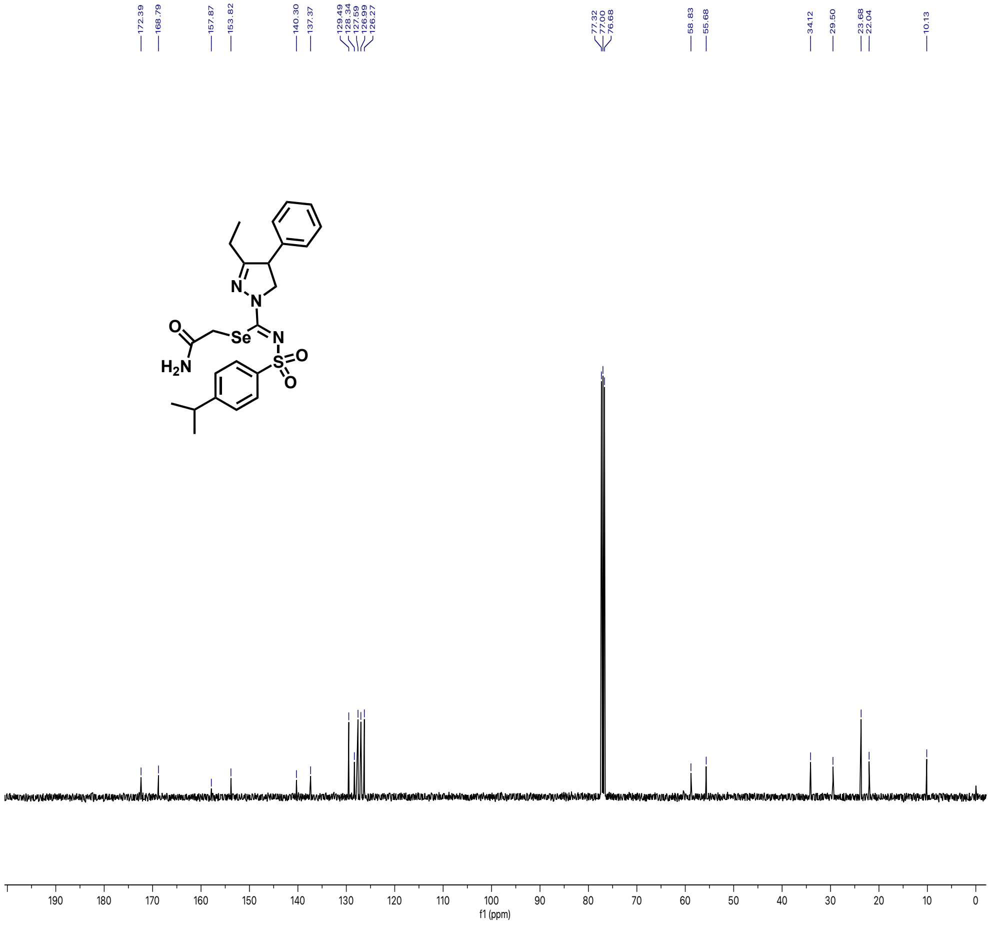

^{1}HNMR and ^{13}CNMR of **35**

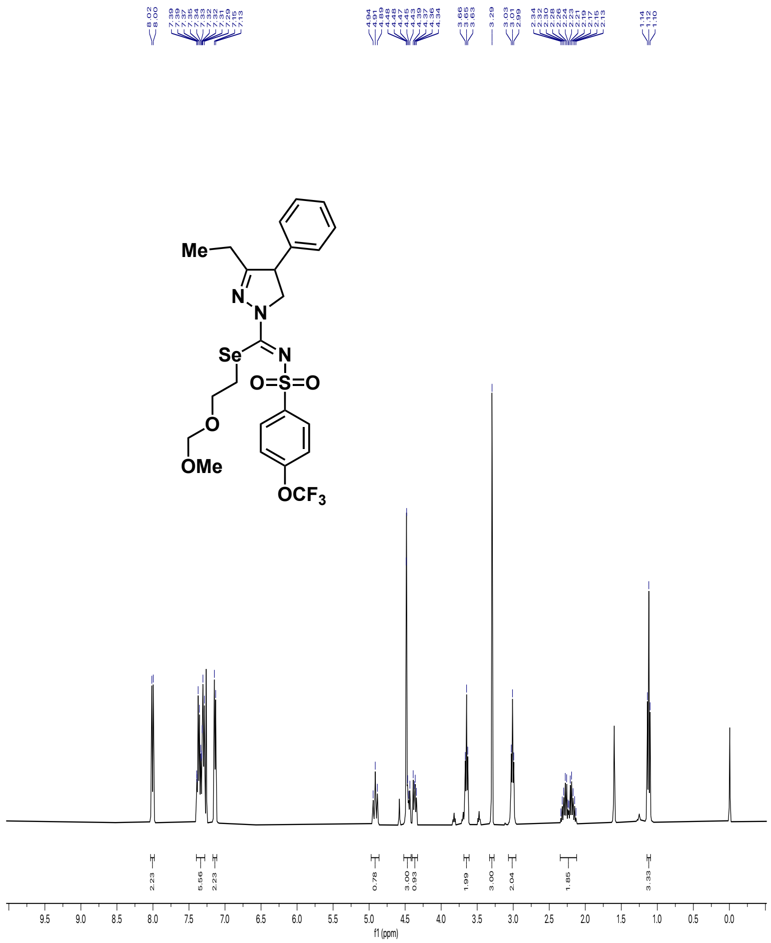

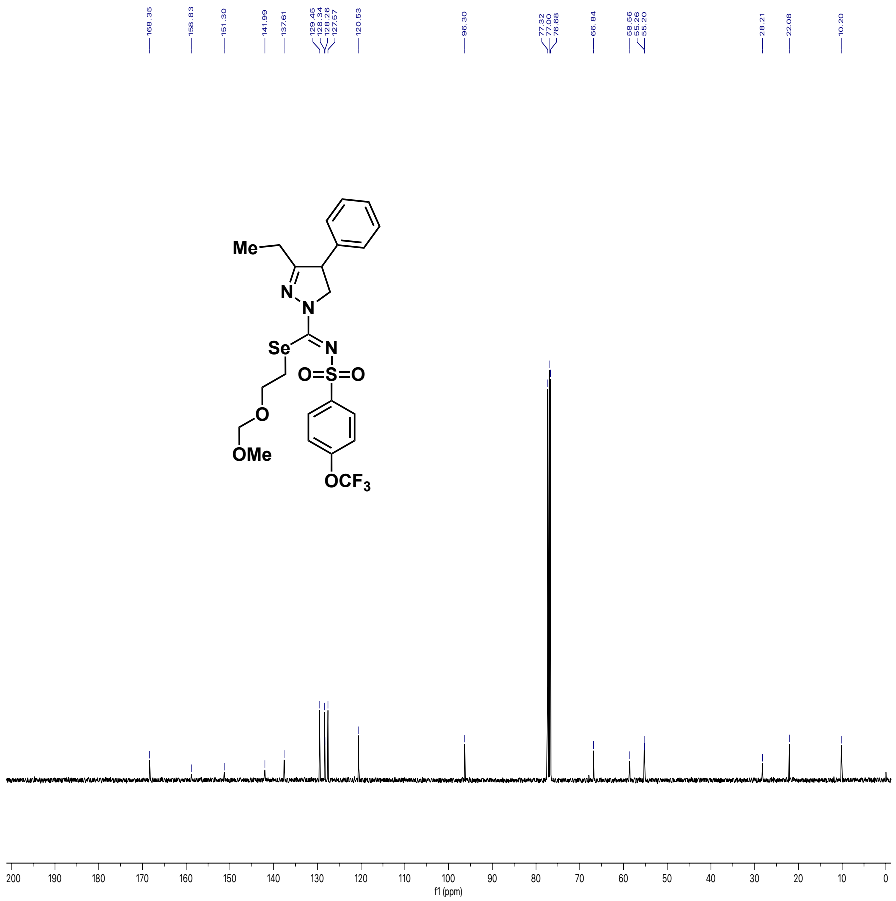

^{1}HNMR and ^{13}CNMR of **36**

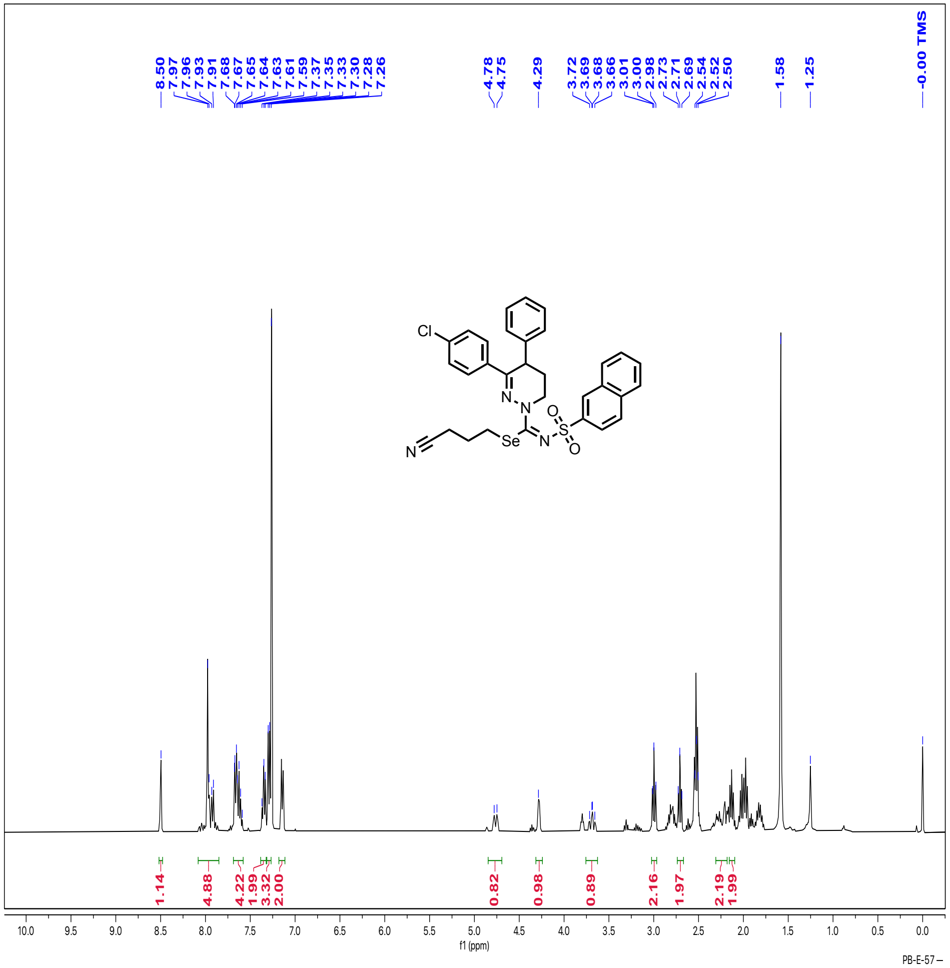

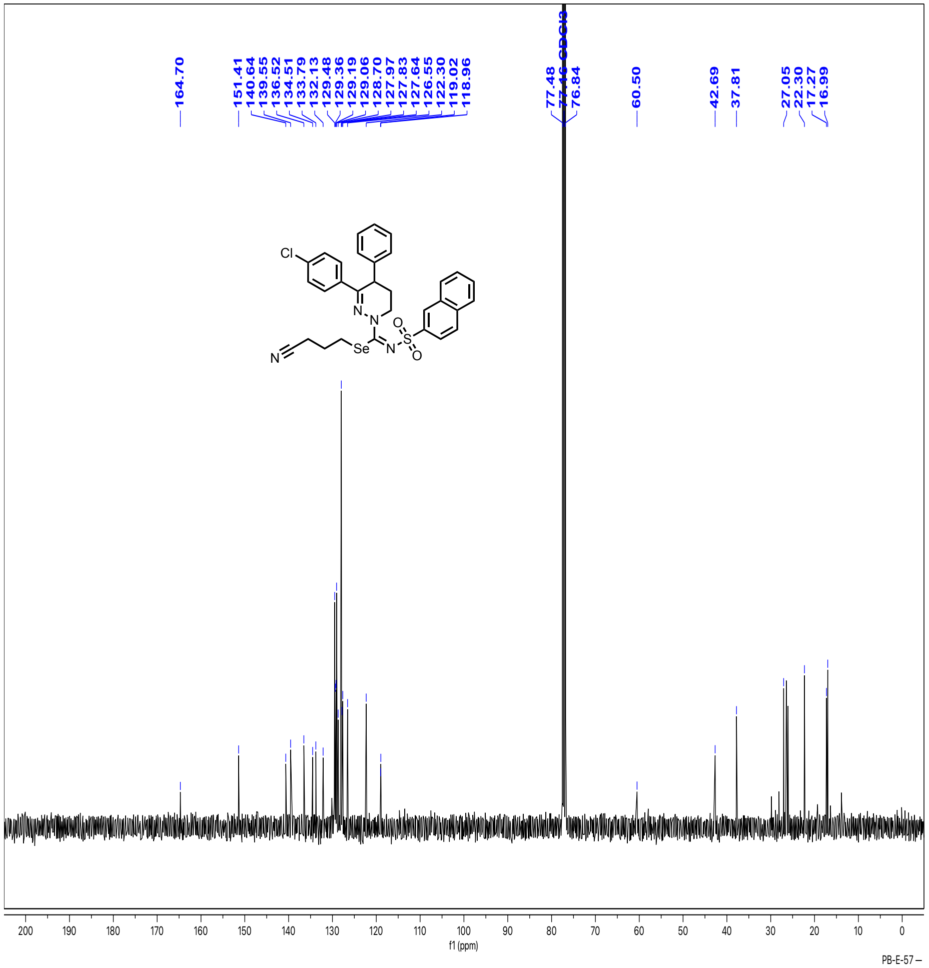

^{1}HNMR and ^{13}CNMR of **37**

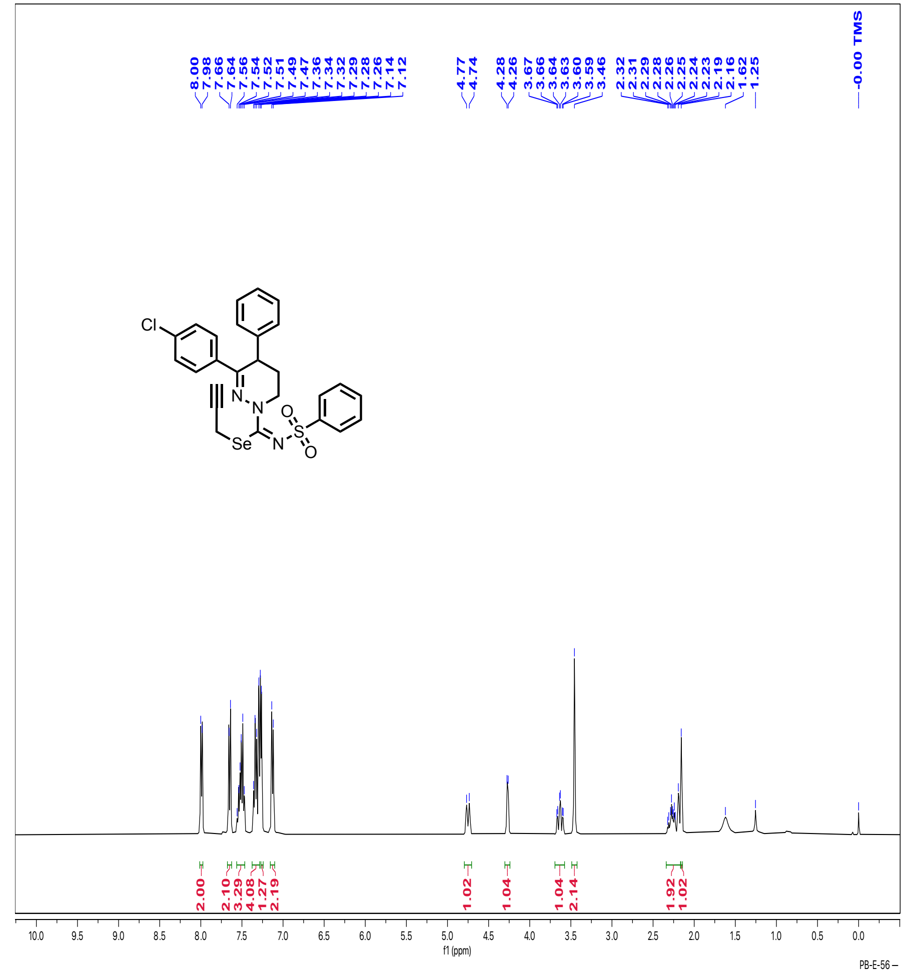

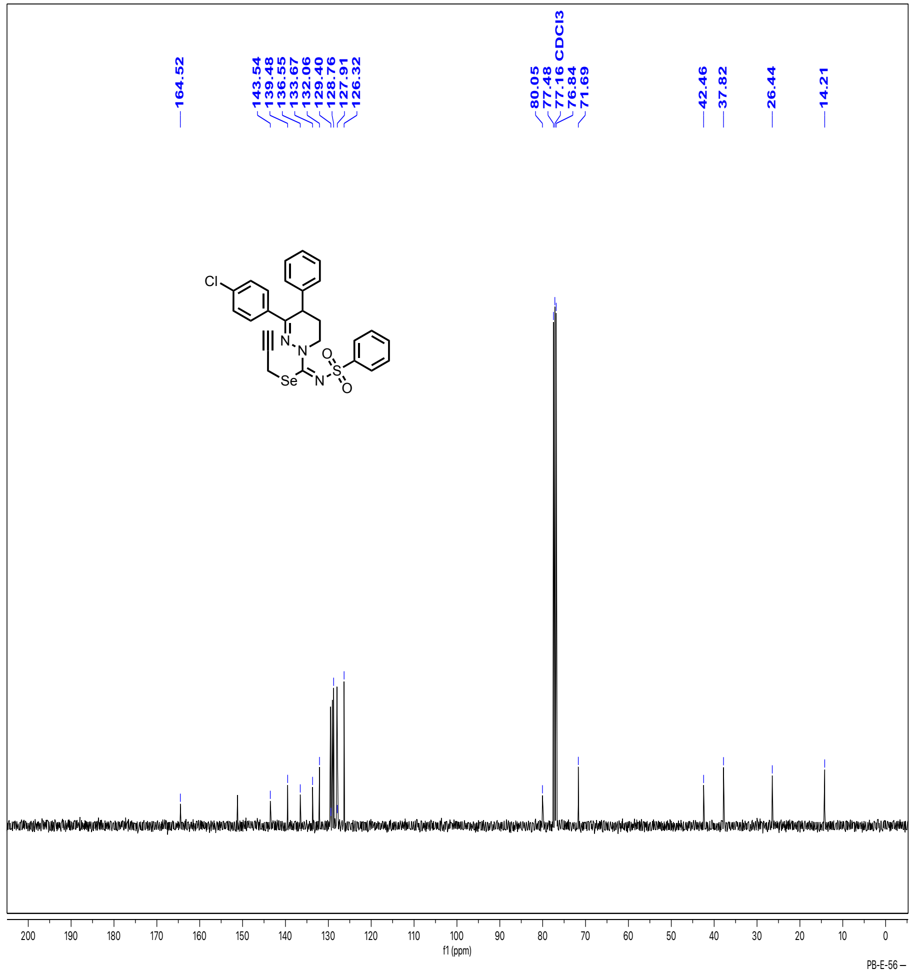

^{1}HNMR and ^{13}CNMR of **38**

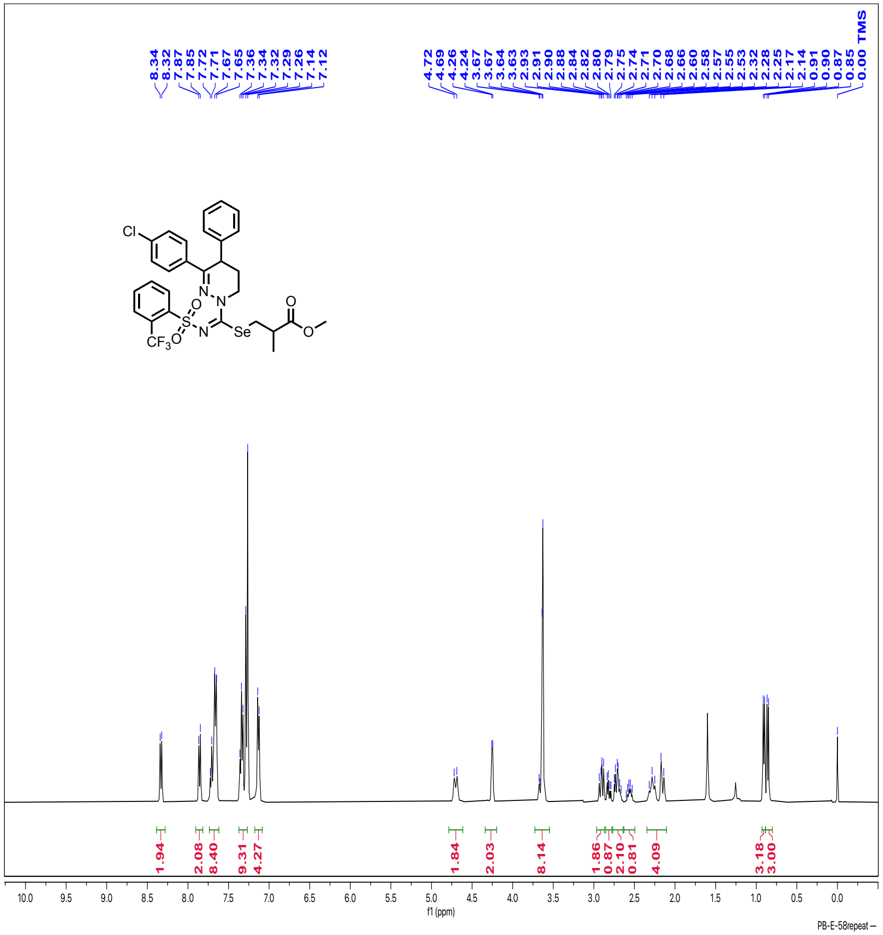

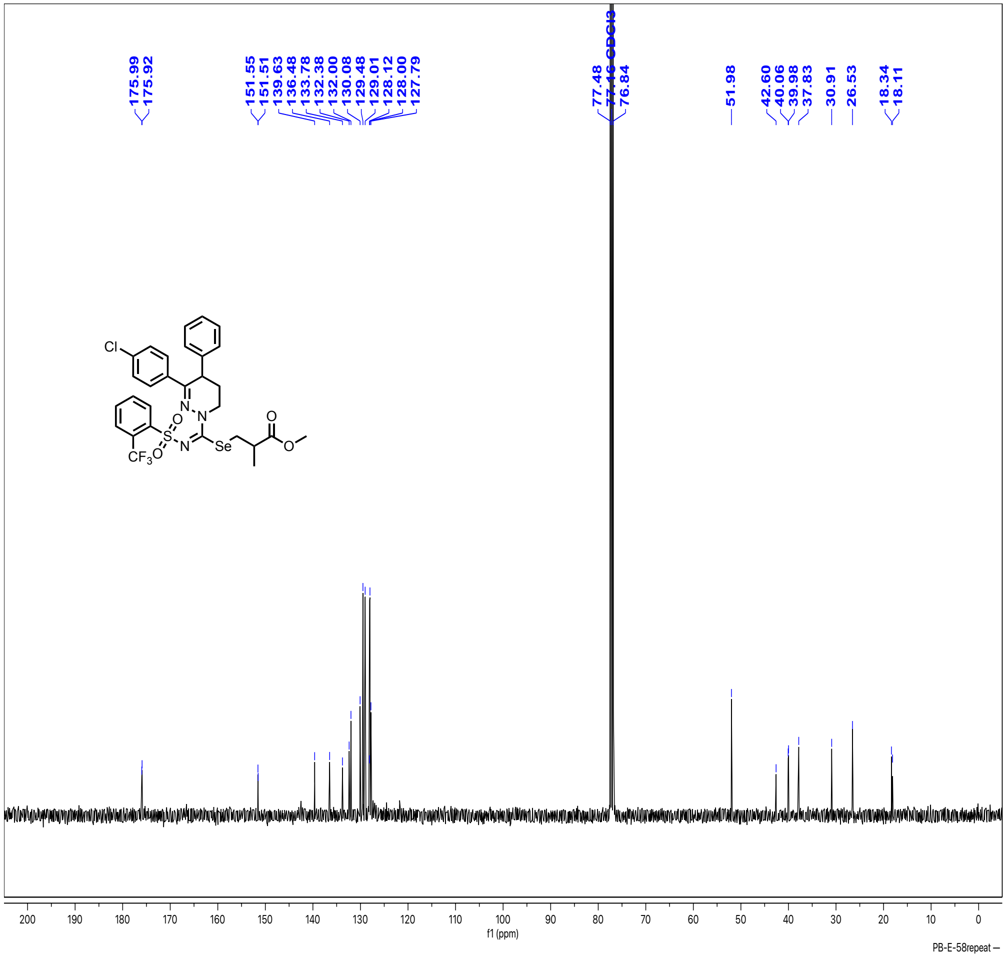

^{1}HNMR and ^{13}CNMR of **(-)-4a**

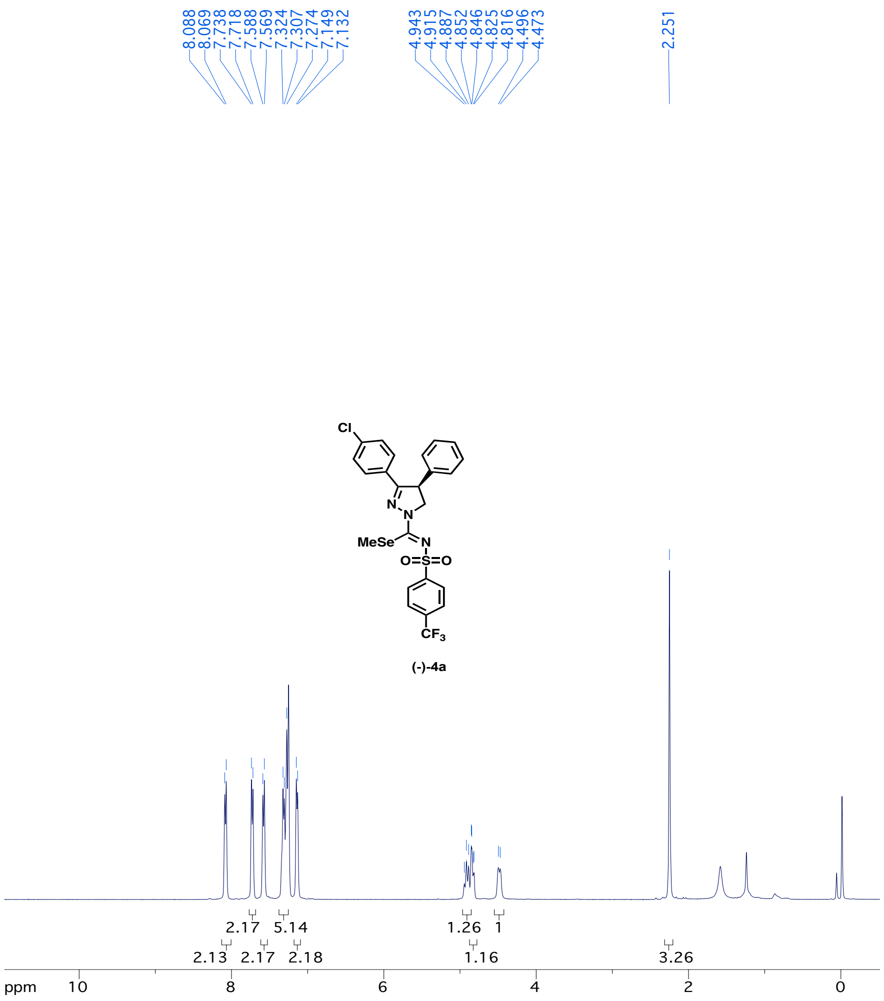

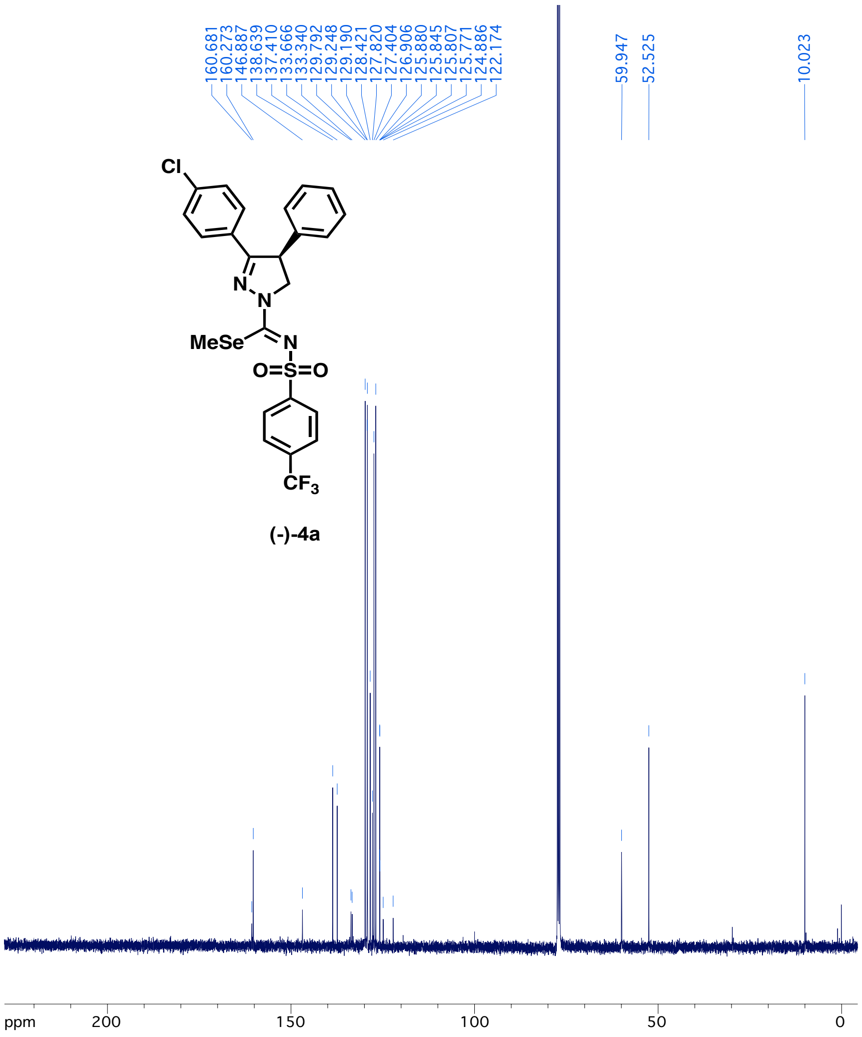

^{1}HNMR and ^{13}CNMR of **(-)-12**

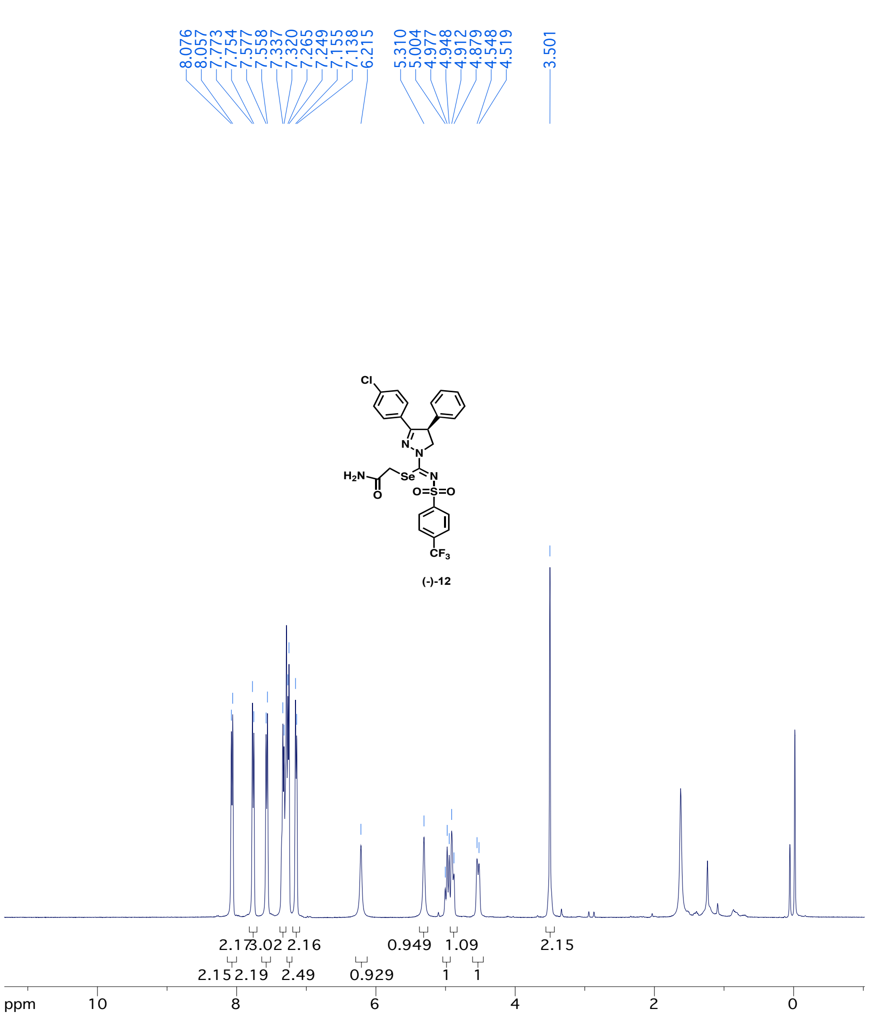

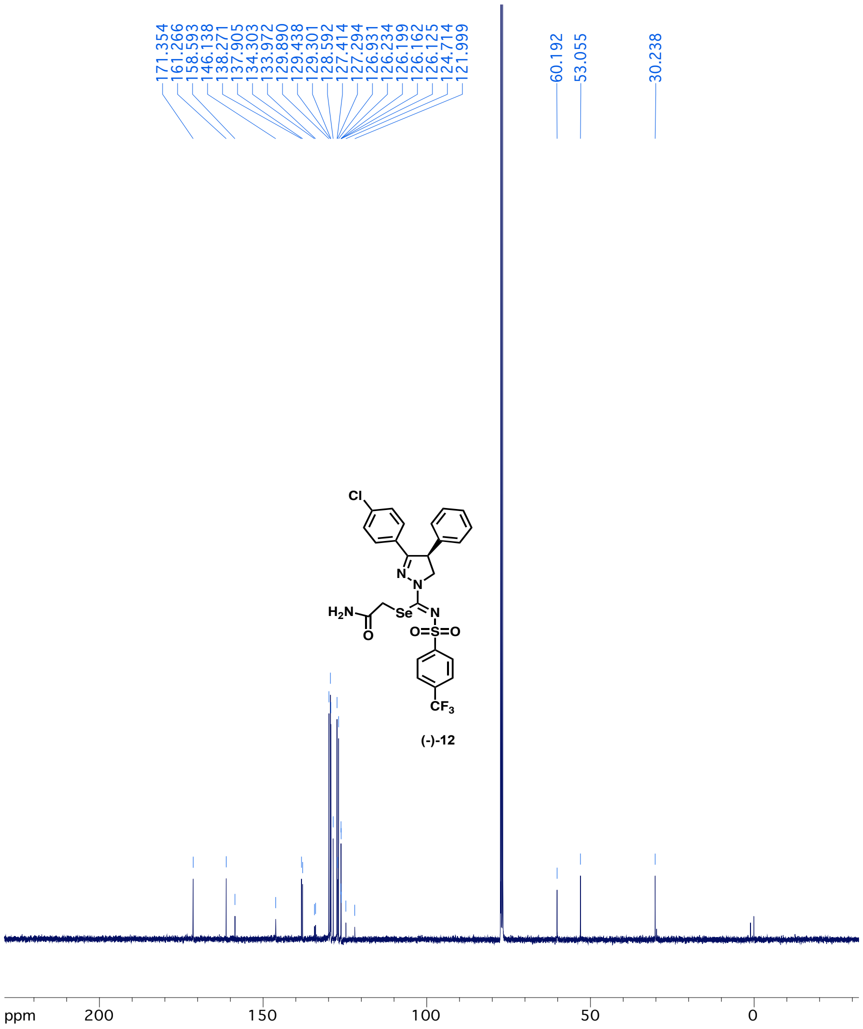

Supplement: Supplementary file 2 [file ja5c16359_si_005.pdf]
